# Methylene Insertion into Nitrogen‐Heteroatom Single Bonds of 1,2‐Azoles via a Zinc Carbenoid: An Alternative Tool for Skeletal Editing

**DOI:** 10.1002/advs.202307563

**Published:** 2023-12-26

**Authors:** Masato Tsuda, Taiki Morita, Yuto Morita, Jun Takaya, Hiroyuki Nakamura

**Affiliations:** ^1^ School of Life Science and Technology Tokyo Institute of Technology 4259 Nagatsuta‐cho Midori‐ku Yokohama 226–8501 Japan; ^2^ Laboratory for Chemistry and Life Science Institute of Innovative Research Tokyo Institute of Technology 4259 Nagatsuta‐cho Midori‐ku Yokohama 226–8501 Japan; ^3^ Department of Chemistry School of Science Tokyo Institute of Technology O‐okayama Meguro‐ku Tokyo 152–8551 Japan

**Keywords:** 1, 2‐azole, ring expansion, zinc carbenoid

## Abstract

The nitrogen‐heteroatom single bonds of 1,2‐azoles and isoxazolines underwent methylene insertion in the presence of CH_2_I_2_ (6 equiv.) and diethylzinc (3 equiv.) to produce a wide variety of the ring‐expanded six‐membered heterocycles. Density functional theory calculations suggest that the methylene insertion proceeds via cleavage of nitrogen‐heteroatom single bonds followed by ring closure.

## Graphical abstract

Methylene insertions of a zinc carbenoid into nitrogen‐heteroatom single bonds of 1,2‐azoles and cyclic oximes are demonstrated. The reaction produces a wide variety of six‐membered heterocycles from easily preparable substrates. Furthermore, density functional theory calculations reveal the reaction mechanism, where cleavage of nitrogen‐heteroatom single bonds and following ring‐closure are essential for the ring‐expansion.

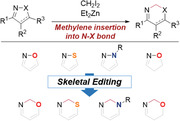

## 1 Introduction

Skeletal editing is the precise modification of molecular skeletons to facilitate the rapid diversification of complex molecular architectures.^[^ ^1^ ^]^ This strategy for modifying arenes and heterocycles involves three main transformations: ring expansion, ring contraction, and atom exchange. In particular, ring expansion with a single carbon insertion, one of the skeletal editing techniques, has been achieved through [2+1]‐cycloaddition of a carbon‐carbon double bond with a carbene or metal carbenoid followed by isomerization.^[^ ^2^ ^]^

**Scheme 1:**
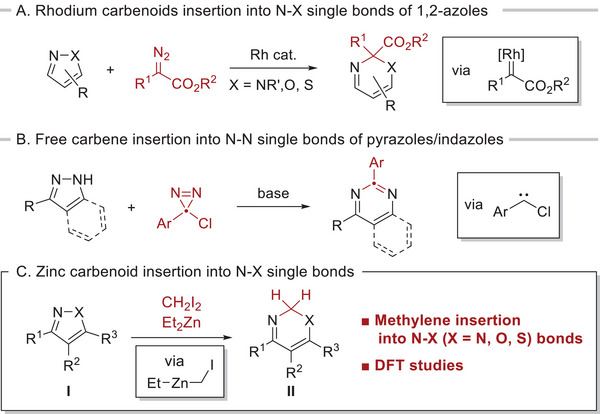
Methylene insertion into nitrogen‐heteroatom single bonds of 1,2‐azoles.

On the other hand, ring expansion by insertion of a carbon atom into a single bond is still less common. Diazo compounds are known as the most widely used carbene or transitional metal carbenoid precursors^[^ ^3^ ^]^ and the corresponding reactive intermediates insert into various types of single bonds, including C─C,^[^ ^4^ ^]^ C─O,^[^ ^5^ ^]^ C─Si,^[^ ^6^ ^]^ and C─S^[^ ^7^ ^]^ bonds in cyclic compounds. Nitrogen‐heteroatom single bonds in heteroarenes also undergo thering expansion with a single carbon insertion. Rhodium carbenoids insert into the N─X (X = N, O, S) single bonds of 1,2‐azoles^[^ ^8^ ^]^ to give the corresponding six‐membered products with a single carbon atom inserted (**Scheme** **1**‐A). Although these strategies using diazo precursors lead to various transformations of the compounds, the diazo compounds have structural limitations to stabilize themselves.

Diazirines are alternative practical carbene precursors. The stability of diazirines allows their application in photoaffinity probes.^[^ ^9^ ^]^ Although the free carbenes generated from diazirines by UV light have been reported to insert C─H, N─H, O─H, and Si─H single bonds,^[^ ^10^ ^]^ the reactivity of diazirines with other types of single bonds remains unknown. Recently, Levin et al. reported ring expansion from pyrazoles to pyrimidines via benzyl carbenes generated from chlorodiazirines (Scheme 1‐B).^[^ ^11^ ^]^

Zinc carbenoids are well‐known reagents for cyclopropanation of carbon–carbon double bonds, called Simmons–Smith cyclopropanation. Especially, Furukawa et al. reported the reliable and reproducible iodomethylzinc reagent (EtZnCH_2_I) prepared from diethylzinc (Et_2_Zn) and CH_2_I_2_.^[^ ^12^ ^]^ Further development of zinc carbenoid reagents has improved their reactivity with unreactive olefines^[^ ^13^ ^]^ and enabled their application in asymmetric synthesis.^[^ ^14^ ^]^ However, zinc carbenoid insertion into single bonds in cyclic compounds has not been reported.

We have developed direct functionalization of isoxazoles to access multi‐functionalized or heteroarene‐fused isoxazoles.^[^ ^15^ ^]^ During the way of investigation, we found an unexpected methylene insertion of a zinc carbenoid into the N─O single bond of isoxazole‐fused azaborines despite the presence of a vinyl group in a molecule.^[^ ^15f^ ^]^ In this study, we demonstrate methylene insertion into nitrogen‐heteroatom (N─X: X = N, O, S) single bonds of 1,2‐azoles **I** via a zinc carbenoid (EtZnCH_2_I) to afford the corresponding ring‐expanded products **II** (Scheme 1‐C). Furthermore, we examined the mechanism of this ring expansion by density functional theory (DFT) calculations to elucidate the unique reactivity of the iodomethylzinc reagent. Since the resulting 2*H*‐1,3‐oxazines are labile under acidic or heating conditions, their general synthetic methods have not been established.^[^ ^16^, ^17^ ^]^

## 2 Results and Discussion

### 2.1 Methylene Insertion into N─O Single Bonds of Isoxazoles

We first examined the methylene insertion into 3,5‐diphenylisoxazole (**1a**) (**Table** **1**). Treatment of **1a** with excess amount of CH_2_I_2_ (10 equiv.) and Et_2_Zn (5 equiv.) in CH_2_Cl_2_ gave the corresponding ring‐expanded product **2a** in 63% yield (entry 1). Although the reduction of the amount of CH_2_I_2_ and Et_2_Zn to one‐fifth resulted in the poor yield of **2a** (5%: entry 2), the use of CH_2_I_2_ (4 equiv.) and Et_2_Zn (2 equiv.) gave the product **2a** in moderate yield (56%, entry 3). Next, solvent effects on the methylene insertion were examined (entries 4–7). Although oxazine **2a** was not obtained in THF and ethyl acetate (entries 4 and 5, respectively), oxazine **2a** was generated in moderate yield in toluene (42%, entry 6) and in good yield in 1,2‐dichloroethane (62%, entry 7). Diluted condition (in 0.05 m) increased the yield of **2a** (68%, entry 8). When the reaction was carried out at 10 °C, oxazine **2a** was obtained in the better yield (86%, entry 9). However, longer reaction time did not improve the yield of **2a** but afforded several unidentified by‐products (entry 10). Finally, the use of CH_2_I_2_ (6 equiv.) and Et_2_Zn (3 equiv.) gave the best result: oxazine **2a** was obtained in 91% yield with 6% recovery of **1a** (entry 11). To clarify the importance of zinc species, control conditions were examined by using several types of zinc species. However, no other zinc species than Et_2_Zn gave oxazine **2a** (Table S1, Supporting Information). Needless to say, **1a** was not consumed in the absence of Et_2_Zn (entry 12).

**Table 1:** Optimization of methylene insertion into N─O single bond of isoxazole **1a**.

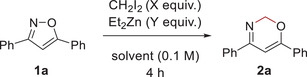						
Entry^a)^	X	Y	Solvent	Temp [°C]	2a [%]^b)^	Recovered **1a** [%]^b)^
1	10	5	CH_2_Cl_2_	0 to r.t.	63	9
2	2	1	CH_2_Cl_2_	0 to r.t.	5	22
3	4	2	CH_2_Cl_2_	0 to r.t.	56	0
4	4	2	THF	0 to r.t.	0	84
5	4	2	EtOAc	0 to r.t.	0	quant.
6	4	2	toluene	0 to r.t.	42	0
7	4	2	DCE	0 to r.t.	62	0
8^c)^	4	2	DCE	0 to r.t.	68	7
9^c)^	4	2	DCE	10	86	9
10^c^ ^,^ ^d)^	4	2	DCE	10	55	trace
11^c)^	6	3	DCE	10	91	6
12^c)^	6	0	DCE	10	0	quant.

^a)^ Reaction conditions: **1a** (0.20 mmol), CH_2_I_2_ (X equiv.), Et_2_Zn (Y equiv.), solvent (2 mL), 4 h;

^b)^ NMR yield using dibromomethane as an internal standard;

^c)^ DCE (4 mL, 0.05 m) was used;

^d)^ reaction time: 12 h;

Ph = phenyl, THF = tetrahydrofuran, EtOAc = ethyl acetate, DCE = 1,2‐dichloroethane.

With the optimized conditions (Table 1, entry 11), we examined the methylene insertion into various di‐ or tri‐substituted isoxazoles **1** (**Table** **2**). Although oxazine **2a** was labile on silica gel, its decomposition was suppressed by deactivation of silica gel using triethylamine (1% v/v in the eluent), and oxazine **2a** was isolated in 80% yield by preparative thin‐layer chromatography. The reaction was applicable to a 1.0 mmol scale reaction, and **2a** was obtained without a significant decrease in yield (76%). With the established procedures, isoxazoles having aryl groups gave oxazines **2b–d** in good yields. Isoxazole **1e** having a sterically hindered 2‐tolyl group at the C‐3 position (R^1^) gave oxazine **2e** in low yield (36%). Although alkyl groups such as cyclohexyl (**1f**) and *tert*‐butyl (**1** **g**) were tolerated to give the corresponding oxazines **2f** and **2** **g** in 26% and 62% yields, respectively, an electron withdrawing group such as an ethoxy carbonyl group gave oxazine **2** **h** in high yield (85%). Aryl groups at the C‐5 position gave the products **2i‐l** in moderate yields (42–52%). Although *tert*‐butyl group and ester group were also tolerated at the C‐5 position, oxazine **2n** having an ethyl ester group was obtained in lower yield (**2h**: 85% vs **2n**: 42%). We carefully examined the difference in yields between products **2** **h** and **2n**. A total of three experiments were performed for each, and the average yields were 81% and 41%, respectively (for each of yields, see Supporting Information). These results indicate that the difference in yields is due to the substituent effects. Furthermore, silyl substituents as R^3^ gave oxazines **2o** and **2p** in high yields. Trisubstituted isoxazoles **1q** and **1r** gave the corresponding oxazines **2q** and **2r** in 56% and 39% yields, respectively, without affecting the bromo and ester substituents in the molecule. Benzoisoxazoles **1s** and **1t** were also converted to the corresponding bicyclic oxazines in moderate to high yield (**2s**: 61%, **2t**: 88%). Furthermore, we applied the methylene insertion to the late‐stage skeletal editing of drug molecules. Zonisamide (**1u**),^[^ ^18^ ^]^ a drug approved by the FDA in 2000, afforded the product **2u** in 17% yield. Benzioxazole **1v**,^[^ ^19^ ^]^ a histone deacetylase (HDAC) inhibitor also underwent the methylene insertion selectively into the N─O bond of isoxazole in the presence of a 1,2,3‐triazole ring in the molecule, giving the corresponding product **2v** in 16% yield. As for unsuccessful experiments,^[^ ^20^ ^]^ isoxazoles with nonsubstituted (**1w**) and monosubstituted at the C‐3 (**1x**) or C‐5 (**1y**) position did not afford the corresponding oxazines **2w‐y**, suggesting that substituents R^1^ and R^3^ strongly affect this reaction (for further unsuccessful examples, see: Figure S1, Supporting Information). The DFT calculations suggest the possibility of oxazine formation, although the presence or absence of substituents affects the activation barrier to *N*‐alkylation. These results suggest that unsubstituted isoxazoles can lead to ring expansion products, but the products formed may be unstable. In fact, these oxazine derivatives have not been reported. Furthermore, complex mixtures were also obtained with mono 3‐ or mono 5‐substitutions. Substrates that were not successful are listed in Figure S1 (Supporting Information).

**Table 2:** Scope and limitations of isoxazoles.^a)^

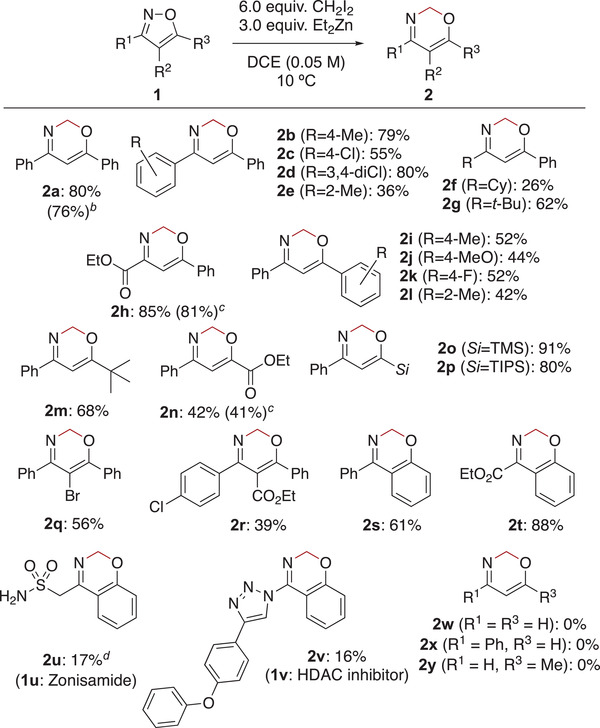

^a)^ Reaction conditions: substrate **1** (0.20 mmol, 1.0 equiv.), CH_2_I_2_ (6.0 equiv.), Et_2_Zn (3.0 equiv.), DCE (4.0 mL), 10 °C, 4 h;

^b)^ 1.0 mmol of **1a** was used;

^c)^ Average yield in three trials;

^d)^ CH_2_I_2_ (3.0 equiv.) and Et_2_Zn (1.5 equiv.) were used;

Me = methyl, Cy = cyclohexyl, *t*‐Bu = *tert*‐butyl, TMS = trimethylsilyl, TIPS = triisopropylsilyl.

### 2.2 Methylene Insertion into N─X Single Bonds of 1,2‐Azoles and Cyclic Oximes

We next investigated ring‐expansion of other 1,2‐azoles and related heterocycles (**Table** **3**). When the 3,5‐diphenylisothiazole **3a** was exposed to the optimized conditions, the ring‐expanded product **4a** was generated in 35% yield with 65% recovery of the starting material **3a**. We also examined methylene insertion of EtZnCH_2_I into N─N single bonds of pyrazoles. Although *N*‐tosylated pyrazole **3b** gave dihydropyrimidine **4b** in 19% yield, pyrazoles having other substituents (R = H, acetyl, *tert*‐butoxycarbonyl, methanesulfonyl) did not give the corresponding products. Indazoles having methoxy (**3c**), benzyloxy (**3d**), or 2,4,6‐trimethylbenzoyloxy (**3e**) groups afforded the corresponding bicyclic dihydropyrimidines **4c–e** in 31–39% yields. Increasing the reaction temperature or the amount of the iodomethylzinc reagent did not improve the reaction yield. On the other hand, unsubstituted indazole (R = H) gave *N*‐methyindazole in 74% yield via methylene insertion into N─H single bond (Scheme S1, Supporting Information). Furthermore, the current methylene insertion mediated by zinc carbenoid was applicable to five‐membered cyclic oximes. Indeed, diphenyl dihydrooxazine **4f** was produced in 80% yield from diphenylisoxazoline **3f**. The ring‐expanded products **4** **g** and **4** **h** were also obtained albeit in 30% and 57% yields, respectively. The low yields of **4** **g** and **4** **h** are probably due to the instability of dihydro oxazine structures which were readily converted to β‐keto alcohols via hydrolysis. In fact, the hydrolyzed products **5i** and **5j** were obtained from **3i** and **3j**, respectively, and the desired **4i** and **4j** were not observed. In the same manner, the methylene insertion into the six‐membered oxime **3k** was attempted, resulting in only γ‐keto alcohol **5k** in 74% yield.

**Table 3:** Investigation of methylene insertion to other 1,2‐azoles 4a‐e and cyclic oximes 4f‐k.^a)^

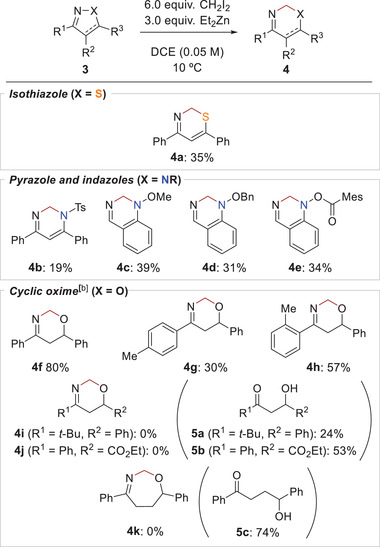

^a)^ Reaction conditions: substrate **3** (0.20 mmol, 1.0 equiv.), CH_2_I_2_ (1.20 mmol, 6.0 equiv.), Et_2_Zn (0.60 mmol, 3.0 equiv.), 1,2‐dichloroethane (4 mL), 10 °C, 4 h;

^b)^ Reaction conditions: substrate **3** (0.20 mmol, 1.0 equiv.), CH_2_I_2_ (0.60 mmol, 3.0 equiv.), Et_2_Zn (0.30 mmol, 1.5 equiv.), 1,2‐dichloroethane (4 mL), 10 °C, 4 h. Ts = tosyl, Bn = benzyl, Mes = mesityl.

### 2.3 Mechanistic Investigation

**Figure 1:**
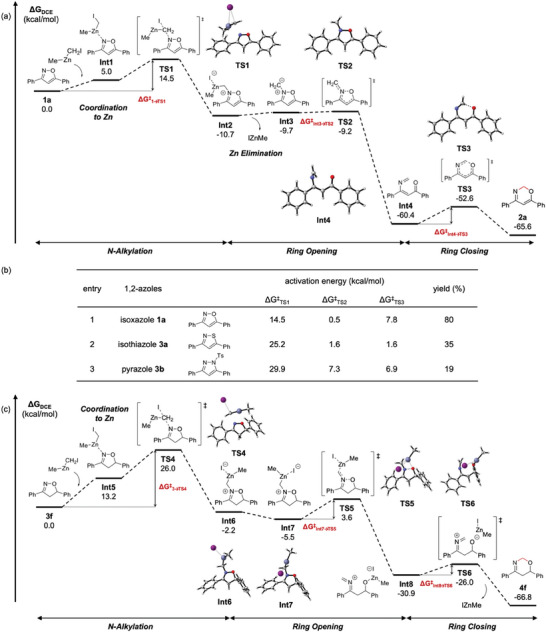
a) Gibbs free energy profile of methylene insertion into the N─O single bond of isoxazole **1a**; b) the summary of activation energies and product yield of each 1,2‐azole and c) Gibbs free energy profile of methylene insertion into the N─O single bond of cyclic oxime **3f** via MeZnCH_2_I. All calculations were conducted at the level of B3LYP‐D3/LANL2DZ for Zn, I, and 6–31G(d,p) for other elements in 1,2‐dichloroethane (PCM).

To clarify the mechanism of the methylene insertion, we conducted DFT calculations on the ring‐expansion of isoxazole **1a** and cyclic oxime **3f**. All calculations were performed by the Gaussian 16 program at the level of B3LYP‐D3/LANL2DZ for I and Zn and 6–31G(d,p) for other elements in 1,2‐dichloroethane (polarizable continuum model, PCM). MeZnCH_2_I^[^ ^21^ ^]^ was used as a model of zinc carbenoid for the calculations. We first examined the possibility of concerted insertion of zinc carbenoid into the N─O single bond, similar to the well‐known mechanism of Simmons–Smith reaction.^[^ ^22^ ^]^ However, no desired transition state structures were obtained, and the candidates converged to *N*‐ or *O*‐alkylated structures. Therefore, we focused on the stepwise path (**Figure** **1**–a). Isoxazole **1a** and MeZnCH_2_I formed the complex **Int1**, and S_N_2‐like *N*‐alkylation occurred between the nitrogen of isoxazole **1a** and MeZnCH_2_I, leading to the intermediate **Int2** via the transition state **TS1**. The energy barrier required for this *N*‐alkylation (Δ*G* ^‡^_1a→TS1_) is 14.5 kcal mol^−1^ (also see entry 1 in Figure 1–b). After the *N*‐alkylation, the zinc moiety (IZnMe) was eliminated from the intermediate **Int2** to afford the ylide intermediate **Int3**, followed by the N─O single bond cleavage to give the intermediate **Int4** through the transition state **TS2**. The energy barrier required for the N─O single bond cleavage (Δ*G* ^‡^_Int3→TS2_) is 0.5 kcal mol^−1^, and this small energy barrier agrees with the calculation by Khlebnikov's group.^[^ ^23^ ^]^ Finally, electrocyclization of the intermediate **Int4** occurred through the transition state **TS3** with an energy barrier (Δ*G* ^‡^_Int4→TS3_ = 7.8 kcal mol^−1^) to yield oxazine **2a**. These calculations suggest that the initial step from **1a** to **TS1** is a rate‐determining step of the entire reaction and that the coordination to zinc in **Int1** would promote this *N*‐alkylation step. This was supported by the control experiment where no reaction was observed in the absence of diethylzinc (Table 1, entry 12).

To compare the reactivities of 1,2‐azoles, we calculated the energy profiles of isothiazole **3a** and *N*‐tosyl‐3,5‐diphenylpyrazole **3b** (for the whole energy profiles, see Figures S2 and S3, Supporting Information). The activation energies in the steps **TS1**, **TS2**, and **TS3**, and product yields for 1,2‐azoles **1a**, **3a**, and **3b** are summarized in Figure 1b. The rate‐determining step of each substrate is the same *N*‐alkylation step. The activation energies required for isothiazole **3a** and pyrazole **3b** were higher than that of isoxazole **1a** (**1a**: Δ*G* ^‡^_TS1_ = 14.5 kcal mol^−1^ vs **3a**: Δ*G* ^‡^_TS1_ = 25.2 kcal mol^−1^ and **3b**: Δ*G* ^‡^_TS1_ = 29.9 kcal mol^−1^). These results are consistent with the lower yields of products **4a** (35%) and **4b** (19%) compared to **2a** (80%). The activation energies required for the following ring‐opening step (Δ*G* ^‡^_TS2_) were also higher than that of isoxazole **1a**. In contrast to the first and second steps, the less activation energies were needed for the final ring‐closing steps required less activation energy (**1a**: Δ*G* ^‡^_TS3_ = 7.1 kcal mol^−1^ vs **3a**: Δ*G* ^‡^_TS3_ = 1.6 kcal mol^−1^ and **3b**: Δ*G* ^‡^_TS3_ = 6.9 kcal mol^−1^).

We next demonstrated the DFT calculation on the insertion of five‐membered oxime **3f** (Figure 1c). Oxime **3f** has a chiral center, therefore (*R*)‐isoxazoline **3f** was employed in this calculation. As with the case of isoxazole **1a**, oxime **3f** underwent the *N*‐alkylation via S_N_2‐like transition state **TS4** with higher activation energy (**1a**: Δ*G* ^‡^_TS1_ = 14.5 kcal mol^−1^ vs **3f**: Δ*G* ^‡^_TS4_ = 26.0 kcal mol^−1^) after coordination to the zinc species (**Int5**) and afforded the intermediate Int6. After the conformational change from **Int6** to **Int7**, the intermediate **Int7** underwent N─O single bond cleavage via the transition state **TS5** with the activation energy (Δ*G* ^‡^_TS5_) of 9.1 kcal mol^−1^. Finally, the intermediate **Int8** cyclized to afford the product **4f** via the transition state **TS6** and subsequent elimination of the zinc moiety. The reaction barrier (Δ*G* ^‡^_TS6_) was 4.9 kcal mol^−1^, a suitable activation energy for the formation of the product **4f**. In contrast to the mechanisms for 1,2‐azoles, the zinc species (IZnMe) was involved in all steps after *N*‐alkylation. Therefore, the zinc species would be essential to sustain structures of the intermediates from **Int6** to **Int8**.

## 3 Conclusion

In conclusion, we have demonstrated the methylene insertion by a zinc carbenoid (EtZnCH_2_I) into nitrogen‐heteroatom single bonds in 1,2‐azoles and cyclic oximes. This transformation enables rapid access to synthetically challenging ring‐expanded heterocycles from the readily available 1,2‐azole scaffolds. The DFT calculations provided insight into the reaction mechanism of the methylene insertion by the zinc carbenoid and the difference in reactivity toward 1,2‐azoles and cyclic oximes. Since ring expansion with a single carbon insertion is an important skeletal editing technique, the current methylene insertion into nitrogen‐heteroatom single bonds in 1,2‐azoles and cyclic oximes provides an effective tool for scaffold hopping of heterocycles, especially in medicinal chemistry.^[^ ^1^ ^]^ Further extensions of methylene insertion with zinc carbenoids are currently in progress.

## Conflict of Interest

The authors declare no conflict of interest.

## Supporting information

Supporting Information

## Data Availability

The data that support the findings of this study are available in the supplementary material of this article.
