# Supplementary material for: Methylene Insertion into Nitrogen‐Heteroatom Single Bonds of 1,2‐Azoles via a Zinc Carbenoid: An Alternative Tool for Skeletal Editing
Source: Adv Sci (Weinh). 2023 Dec 26;11(10):2307563. doi: 10.1002/advs.202307563 (PMC10933618; doi:10.1002/advs.202307563)

ADVANCED SCIENCE
Open Access

Supporting Information

for *Adv. Sci.*, DOI 10.1002/advs.202307563

Methylene Insertion into Nitrogen-Heteroatom Single Bonds of 1,2-Azoles via a Zinc Carbenoid: An Alternative Tool for Skeletal Editing

*Masato Tsuda, Taiki Morita, Yuto Morita, Jun Takaya and Hiroyuki Nakamura**

Supporting Information (97 pages)

Methylene Insertion into Nitrogen-Heteroatom Single Bonds of 1,2-Azoles via a Zinc Carbenoid: An Alternative Tool for Skeletal Editing

Masato Tsuda,[1] Taiki Morita,[1,2] Yuto Morita,[3] Jun Takaya,[3] Hiroyuki Nakamura*[1,2]

1) *School of Life Science and Engineering, Tokyo Institute of Technology, 4259 Nagatsuta-cho Midori-ku, Yokohama, 226-8503, Japan*
2) *Laboratory for Chemistry and Life Science, Institute of Innovative Research, Tokyo Institute of Technology, 4259 Nagatsuta-cho Midori-ku, Yokohama, 226-8503, Japan*
3) *Department of Chemistry, Tokyo Institute of Technology, O-okayama, Meguro-ku, Tokyo 152-8551, Japan*

Corresponding E-mail: hiro@res.titech.ac.jp

Table of Contents

1.	General techniques	S2
2.	Screening of zinc species	S2
3.	Methylene insertion into N-H bond of indazole	S3
4.	Synthesis of starting materials	S3
5.	General procedure of methylene insertion	S3
6.	Computational methods	S15
7.	Reference	S65
8.	NMR spectra	S67

1. General techniques

NMR spectra were recorded on a Bruker biospin AVANCE II (400 MHz for ^{1}H, 100 MHz for ^{13}C) or a Bruker biospin AVANCE III (500 MHz for ^{1}H, 125 MHz for ^{13}C) instrument in the indicated solvent. Chemical shifts are reported in units parts per million (ppm) relative to the signal (0.00 ppm) for internal tetramethylsilane for solutions in $CDCl_3$ (7.26 ppm for ^{1}H, 77.16 ppm for ^{13}C). Multiplicities are reported using the following abbreviations: s; singlet, d; doublet, dd; doublet of doublets, t; triplet, dt; doublet of triplets q; quartet, quint; quintet, m; multiplet, br; broad, *J*; coupling constants in Hertz. IR spectra were recorded on a JASCO FT/IR-4200 spectrometer. Only the strongest and/or structurally important peaks are reported as IR data given in cm^{-1}. Mass spectra were measured using a JMS-700 Mstation and Bruker micrOTOF II. All reactions were monitored by thin-layer chromatography carried out on 0.2 mm E. Merck silica gel plates (60F-254) with UV light (254 nm), and were visualized using an aqueous alkaline $KMnO_4$ solution. Gel permeation chromatography (GPC) for purification was performed on Japan Analytical Industry Model LC-9225 NEXT (recycling preparative HPLC) and a Japan Analytical Industry Model UV-600 NEXT ultraviolet detector with a polystyrene gel column (JAIGEL-1H, 20 mm × 600 mm), using chloroform as solvent (3.5 mL/min). Column chromatography was performed on Silica Gel 60 N, purchased from Fuji Silysia Chemical Ltd. Preparative thin-layer chromatography (PTLC) was performed using Wakogel B5-F silica coated plates (1.0 mm) prepared in our laboratory. Preparative high performance liquid chromatography (HPLC) was performed with LC forte/R (YMC) using a normal phase column (Kanto, Mightysil Si60 250 × 20 mm, 5 μm). Isoxazoles **1a-1o**, **1q-r, 1v**, isothiazole **3a**, pyrazoles **3b-e**, cyclic oximes **3f-k** were synthesized according to the literatures.[1]-[19] Diethylzinc (*ca.* 1 mol/L in toluene) was purchased from TCI Research Chemicals.

2. Screening of zinc species

Table S1. Screening of zinc species for the methylene insertion

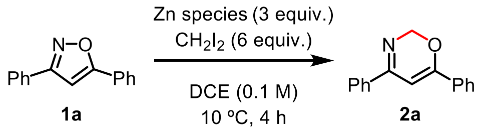

entry	Zinc species	Oxazine **2a** (%)^{a}
1	Zn powder	No Reaction
2	$ZnCl_2$	No Reaction
3	ZnI_2	No Reaction
4	$Zn(OAc)_2$	No Reaction
5	$Zn(OTf)_2$	No Reaction
6	$Zn(CN)_2$	No Reaction
7[20]	Zn(Cu)	No Reaction

^{a} ^{1}H NMR yield using dibromomethane as an internal standard.

3. Methylene insertion into N-H bond of indazole

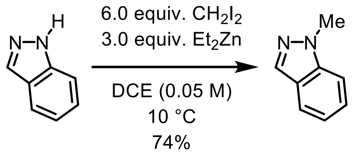

Scheme S1. Methylene insertion into N-H bond of indazole

4. Synthesis of starting materials

3-Phenyl-5-(triisopropylsilyl)isoxazole (1q)

To a mixture of (*Z*)-*N*-hydroxybenzimidoyl chloride (312 mg, 2.0 mmol) and ethynyltriisopropylsilane (445 μL, 2.0 mmol) in Et_2O (4 mL), triethylamine (372 μL, 4.0 mmol) was added dropwise at 0 °C under an argon atmosphere. After being stirred at room temperature for 2 h, the residue was poured into diethyl ether and water. The aqueous layer was extracted with diethyl ether. The combined extract was washed with brine, dried over $MgSO_4$ and concentrated *in vacuo*. The residue was purified by column chromatography on silica gel (hexane : ethyl acetate = 95 : 5) to afford isoxazole **1q** was obtained in 80% yield (483 mg, 1.60 mmol) as a pale yellow oil.^{1}H NMR (500 MHz, $CDCl_3$) δ 7.87 (dd, *J* = 8.0 Hz, *J* = 1.5 Hz, 2H), 7.47-7.41 (m, 3H), 6.80 (s, 1H), 1.41 (sep., *J* = 7.5 Hz, 3H), 1.16 (*d*, J = 7.5 Hz, 2H); ^{13}C NMR (125 MHz, $CDCl_3$) δ 176.2, 160.6, 129.8, 129.4, 129.0, 127.1, 112.2, 18.5, 11.0; FT-IR (neat) 2945, 2867, 1543, 1459, 1386, 913, 767, 749 cm^{-1}; HRMS (ESI-TOF): calcd for $[C_{18}H_{27}NOSi +H]^+$ 302.1940: found 302.1942.

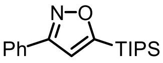

5. Methylene insertion into nitrogen-heteroatom bonds via a zinc carbenoid

General procedure of methylene insertion

To a mixture of 1,2-azole (0.20 mmol, 1.0 equiv.) in 1,2-dichloroethane (4.0 mL), diethylzinc (*ca.* 1 mol/L in toluene) (0.60 mL, 3.0 equiv.) was added at 10 ºC under an argon atmosphere. After stirred at the same temperature for 10 min, diiodomethane (96.5 μL, 6.0 equiv.) was added to the mixture. The reaction mixture was stirred at the same temperature for 4 h. The reaction was traced by TLC analysis. After completion of the reaction, saturated NH_4Cl aq. was added to the mixture. After being stirred for 30 min, the residue was poured into water. The aqueous layer was extracted with two portions of CH_2Cl_2. The combined extract was dried over Na_2SO_4 and concentrated *in vacuo*. The residue was purified by PTLC which was deactivated by 1% triethylamine to afford the desired product.

Notes:

Diethylzinc and diiodomethane should be added following the order mentioned above. The stirring time from the addition of the zinc reagent until that of diiodomethane was also crucial. The optimal time was 10 minutes.

Furthermore, deactivation of silica gel by triethylamine (1% v/v in eluent) was essential for purification. Without this deactivation, products readily decomposed.

4,6-Diphenyl-2*H*-1,3-oxazine (2a)

Following the general procedure using 3,5-diphenylisoxazole **1a** (66.4 mg, 0.30 mmol), purification by PTLC (hexane : acetone : triethylamine = 80 : 20 : 1) afforded the desired product **2a** (56.6 mg, 0.241 mmol, 80%) as a yellow solid. ^{1}H NMR (400 MHz, $CDCl_3$) δ 7.92-7.90 (m, 2H), 7.83-7.81 (m, 2H), 7.47-7.45 (m, 6H), 6.66 (s, 1H), 5.68 (s, 2H); ^{13}C NMR (100 MHz, $CDCl_3$) δ 163.6, 162.8, 137.1, 132.3, 131.1, 130.6, 128.7, 128.6, 126.7, 126.2, 95.9, 81.0; FT-IR (neat) 3085, 3029, 2940, 2834, 1642, 1415, 1088, 1064, 754, 693 cm^{-1}; HRMS (ESI-TOF): calcd for $[C_{16}H_{13}NO + H]^+$ 236.1075: found 236.1070.

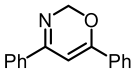

Procedure for 1 mmol-Scale Reaction

To a mixture of isoxazole **1a** (221 mg, 1.0 mmol, 1.0 equiv.) in 1,2-dichloroethane (20 mL), diethylzinc (ca. 1 mol/L in toluene) (3.0 mL, 3.0 equiv.) was added at 10 ºC under an argon atmosphere. After stirred at the same temperature for 10 min, diiodomethane (483 μL, 6.0 equiv.) was added to the mixture. The reaction mixture was stirred at the same temperature for 4 h. The reaction was traced by TLC analysis. After completion of the reaction, saturated NH_4Cl aq. was added to the mixture. After being stirred for 30 min, the residue was poured into water. The aqueous layer was extracted with two portions of CH_2Cl_2. The combined extract was dried over Na_2SO_4 and concentrated *in vacuo*. The residue was purified by column chromatography on silica gel (hexane : acetone : triethylamine = 80 : 20 : 1) to afford the desired product **2a** (164 mg, 0.70 mmol, 70%).

6-Phenyl-4-(*p*-tolyl)-2*H*-1,3-oxazine (2b)

Following the general procedure using 5-phenyl-3-(*p*-tolyl)isoxazole **1b** (47.1 mg, 0.20 mmol), purification by PTLC (hexane : acetone : triethylamine = 80 : 20 : 1) afforded the desired product **2b** (39.4 mg, 0.158 mmol, 79%) as a yellow oil. ^{1}H NMR (500 MHz, $CDCl_3$) δ 7.82-7.80 (m, 4H), 7.49-7.42 (m, 3H), 7.27-7.25 (m, 2H), 6.65 (s, 1H), 5.66 (s, 2H); ^{13}C NMR (125 MHz, $CDCl_3$) δ 163.4, 162.7, 140.9, 134.3, 132.4, 131.1, 129.3, 128.7, 126.7, 126.1, 95.9, 81.0, 21.5; FT-IR (neat) 3029, 2942, 2835, 1736, 1640, 1371, 1078, 817, 758, 699 cm^{-1}; HRMS (ESI-TOF): calcd for $[C_{17}H_{15}NO + H]^+$ 250.1226: found 250.1226.

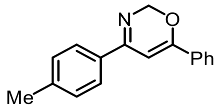

4-(4-Chlorophenyl)-6-phenyl-2*H*-1,3-oxazine (2c)

Following the general procedure using 3-(4-chlorophenyl)-5-phenylisoxazole **1c** (51.1 mg, 0.20 mmol), purification by PTLC (hexane : acetone : triethylamine = 80 : 20 : 1) afforded the desired product **2c** (29.7 mg, 0.110 mmol, 55%) as a yellow solid. ^{1}H NMR (400 MHz, $CDCl_3$) δ 7.84 (d, J = 8.8 Hz, 2H), 7.82-7.79 (m, 2H), 7.48-7.41 (m, 5H), 6.59 (s, 1H), 5.66 (s, 2H); ^{13}C NMR (100 MHz, $CDCl_3$) δ 163.6, 162.8, 137.1, 132.3, 131.1, 130.6, 128.7, 128.6, 126.7, 126.2, 95.9, 81.0; FT-IR (neat) 3060, 3027, 2938, 2839, 1653, 1619, 1371, 1072, 754, 670 cm^{-1}; HRMS (ESI-TOF): calcd for $[C_{16}H_{12}ClNO + H]^+$ 270.0680: found 270.0682.

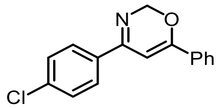

4-(3,4-Dichlorophenyl)-6-phenyl-2*H*-1,3-oxazine (2d)

Following the general procedure using 3-(3,4-dichlorophenyl)-5-phenylisoxazole **1d** (58.0 mg, 0.20 mmol), purification by PTLC (hexane : acetone : triethylamine = 80 : 20 : 1) afforded the desired product **2d** (48.7 mg, 0.160 mmol, 80%) as a yellow oil. ^{1}H NMR (500 MHz, $CDCl_3$) δ 7.99 (d, *J* = 1.5 Hz, 1H), 7.83-7.79 (m, 2H), 7.73-7.69 (m, 1H), 7.51-7.44 (m, 4H), 6.54 (s, 1H), 5.66 (s, 2H); ^{13}C NMR (125 MHz, $CDCl_3$) δ 163.5, 161.1, 137.0, 134.8, 133.1, 131.9, 131.5, 130.6, 128.8, 126.4, 95.0, 80.9; FT-IR (neat) 3062, 2944, 2836, 1653, 1494, 1371, 1073, 756, 699 cm^{-1}; HRMS (ESI-TOF): calcd for $[C_{16}H_{11}Cl_2NO + H]^+$ 304.0290: found 304.0287.

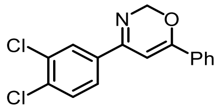

6-Phenyl-4-(*o*-tolyl)-2*H*-1,3-oxazine (2e)

Following the general procedure using 5-phenyl-3-(*o*-tolyl)isoxazole **1e** (47.1 mg, 0.20 mmol), purification by PTLC (hexane : acetone : triethylamine = 80 : 20 : 1) afforded the desired product **2e** (18.2 mg, 0.0729 mmol, 36%) as a yellow oil. ^{1}H NMR (400 MHz, $CDCl_3$) δ 7.90-7.88 (m, 2H), 7.76 (d, *J* = 8.8 Hz, 2H), 7.46-7.44 (m, 3H), 6.95 (d, *J* = 8.8 Hz, 2H), 6.55 (s, 1H), 5.63 (s, 2H); ^{13}C NMR (100 MHz, $CDCl_3$) δ 164.0, 162.9, 162.1, 137.4, 130.5, 128.6, 128.0, 126.8, 124.7, 114.2, 94.5, 81.0; FT-IR (neat) 3057, 3027, 2969, 2921, 2849, 1735, 1359, 1167, 763, 717 cm^{-1}; HRMS (ESI-TOF): calcd for $[C_{17}H_{15}NO + H]^+$ 250.1226: found 250.1228.

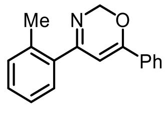

4-Cyclohexyl-6-phenyl-2*H*-1,3-oxazine (2f)

Following the general procedure using 3-cyclohexyl-5-phenylisoxazole **1f** (45.5 mg, 0.20 mmol), purification by PTLC (hexane : acetone : triethylamine = 80 : 20 : 1) afforded the desired product **2f** (12.7 mg, 0.0526 mmol, 26%) as a yellow oil. ^{1}H NMR (500 MHz, $CDCl_3$) δ 7.72-7.71 (m, 2H), 7.45-7.38 (m, 3H), 6.09 (s, 1H), 5.42 (s, 2H), 2.30-2.25 (m, 1H), 1.90-1.82 (m, 4H), 1.42-1.27 (m, 6H); ^{13}C NMR (125 MHz, $CDCl_3$) δ 172.2, 161.8, 132.1, 130.8, 128.5, 125.9, 96.9, 80.3, 45.7, 30.4, 26.1, 26.0; FT-IR (neat) 3056, 2923, 2818, 1736, 1530, 1338, 755, 690 cm^{-1}; HRMS (ESI-TOF): calcd for $[C_{16}H_{19}NO + H]^+$ 242.1539: found 242.1534.

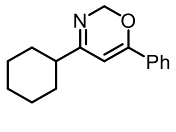

4-(*tert*-Butyl)-6-phenyl-2*H*-1,3-oxazine (2g)

Following the general procedure using 3-(*tert*-butyl)-5-phenylisoxazole **1g** (40.3 mg, 0.20 mmol), purification by PTLC (hexane : acetone : triethylamine = 80 : 20 : 1) afforded the desired product **2g** (26.9 mg, 0.125 mmol, 63%) as a pale yellow oil. ^{1}H NMR (500 MHz, $CDCl_3$) δ 7.72-7.71 (m, 2H), 7.43-7.39 (m, 3H), 6.25 (s, 1H), 5.43 (s, 2H), 1.21 (s, 9H); ^{13}C NMR (125 MHz, $CDCl_3$) δ 174.5, 161.7, 132.5, 130.8, 128.6, 125.9, 95.4, 80.6, 37.5, 28.0; FT-IR (neat) 3005, 2969, 2849, 1737, 1373, 1216, 764, 751 cm^{-1}; HRMS (ESI-TOF): calcd for $[C_{14}H_{17}NO + H]^+$ 216.1383: found 216.1383.

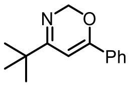

Ethyl 6-phenyl-2*H*-1,3-oxazine-4-carboxylate (2h)

Following the general procedure using ethyl 5-phenylisoxazole-3-carboxylate **1h** (43.4 mg, 0.20 mmol), purification by PTLC (hexane : acetone : triethylamine = 80 : 20 : 1) afforded the desired product **2h** (1st trial: 39.5 mg, 0.170 mmol, 85%, 2nd trial: 36.3 mg, 0.157 mmol, 78%, 3rd trial: 37.5 mg, 0.162 mmol, 81%) as a yellow solid. ^{1}H NMR (400 MHz, $CDCl_3$) δ 7.85-7.83 (m, 2H), 7.50-7.43 (m, 3H), 6.95 (s, 1H), 5.70 (s, 2H), 4.38 (q, J = 7.2 Hz, 2H), 1.39 (t, J = 7.2 Hz, 3H); ^{13}C NMR (125 MHz, $CDCl_3$) δ 163.7, 163.5, 157.7, 131.7, 131.3, 128.8, 126.3, 95.2, 80.5, 62.4, 14.3; FT-IR (neat) 3062, 2929, 2838, 1735, 1508, 1258, 762, 750 cm^{-1}; HRMS (ESI-TOF): calcd for $[C_{13}H_{13}NO_3 + Na]^+$ 254.0788: found 254.0789.

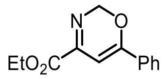

4-Phenyl-6-(*p*-tolyl)-2*H*-1,3-oxazine (2i)

Following the general procedure using 5-(4-methoxyphenyl)-3-phenylisoxazole **1i** (47.1 mg, 0.20 mmol), purification by PTLC (hexane : acetone : triethylamine = 80 : 20 : 1) afforded the desired product **2i** (25.9 mg, 0.103 mmol, 52%) as a yellow oil. ^{1}H NMR (500 MHz, $CDCl_3$) δ 7.90-7.89 (m, 2H), 7.71 (d, J = 8.0 Hz, 2H), 7.46-7.45 (m, 3H), 7.26-7.24 (m, 2H), 6.62 (s, 1H), 5.65 (s, 2H); ^{13}C NMR (125 MHz, $CDCl_3$) δ 163.9, 163.1, 141.7, 137.2, 130.6, 129.5, 128.6, 126.8, 126.2, 95.3, 81.0, 21.7; FT-IR (neat) 3060, 2992, 2927, 1701, 1569, 1330, 1052, 754, 691 cm^{-1}; HRMS (ESI-TOF): calcd for $[C_{17}H_{15}NO + H]^+$ 250.1226: found 250.1230.

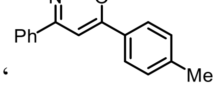

6-(4-Methoxyphenyl)-4-phenyl-2*H*-1,3-oxazine (2j)

Following the general procedure using 5-(4-methoxyphenyl)-3-phenylisoxazole **1j** (50.3 mg, 0.20 mmol), purification by PTLC (hexane : acetone : triethylamine = 80 : 20 : 1) afforded the desired product **2j** (23.3 mg, 0.0878 mmol, 44%) as a yellow oil. ^{1}H NMR (400 MHz, $CDCl_3$) δ 7.90-7.88 (m, 2H), 7.76 (d, J = 8.8 Hz, 2H), 7.46-7.44 (m, 3H), 6.95 (d, J = 8.8 Hz, 2H), 6.55 (s, 1H), 5.63 (s, 2H), 3.86 (s, 3H); ^{13}C NMR (100 MHz, $CDCl_3$) δ 164.0, 162.9, 162.1, 137.4, 130.5, 128.6, 128.0, 126.8, 124.7, 114.2, 94.5, 81.0, 55.6; FT-IR (neat) 3032, 2992, 2834, 2780, 1628, 1492, 1090, 761, 687 cm^{-1}; HRMS (ESI-TOF): calcd for $[C_{17}H_{15}NO_2 + H]^+$ 266.1176: found

266.1178.

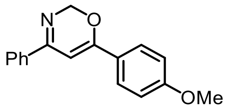

6-(4-Fluorophenyl)-4-phenyl-2*H*-1,3-oxazine (2k)

Following the general procedure using 5-(4-fluorophenyl)-3-phenylisoxazole **1k** (47.9 mg, 0.20 mmol), purification by PTLC (hexane : acetone : triethylamine = 80 : 20 : 1) afforded the desired product **2k** (27.2 mg, 0.107 mmol, 54%) as a yellow oil. ^{1}H NMR (500 MHz, $CDCl_3$) δ 7.89-7.88 (m, 2H), 7.82-7.79 (m, 2H), 7.49-7.43 (m, 3H), 7.13 (t, J = 8.5 Hz, 2H), 6.58 (s, 1H), 5.66 (s, 2H); ^{13}C NMR (125 MHz, $CDCl_3$) δ 165.6, 163.6, 161.9, 137.0, 130.7, 128.7, 128.53, 128.50, 128.4, 128.3, 126.7, 116.0, 115.8, 95.6, 81.1; FT-IR (neat) 2980, 2931, 2907, 2856, 1732, 1636, 1291, 1234, 1108, 761, 693 cm^{-1}; HRMS (ESI-TOF): calcd for $[C_{16}H_{12}FNO +H]^+$ 254.0976: found 254.0981.

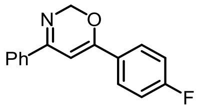

4-Phenyl-6-(*o*-tolyl)-2*H*-1,3-oxazine (2l)

Following the general procedure using 3-phenyl-5-(*o*-tolyl)isoxazole **1l** (47.1 mg, 0.20 mmol), purification by PTLC (hexane : acetone : triethylamine = 80 : 20 : 1) afforded the desired product **2l** (22.1 mg, 0.0886 mmol, 42%) as a yellow oil. ^{1}H NMR (500 MHz, $CDCl_3$) δ 7.90-7.88 (m, 2H), 7.52-7.43 (m, 4H), 7.37-7.34 (m, 1H), 7.28-7.24 (m, 2H), 6.30 (s, 1H), 5.67 (s, 2H), 2.51 (s, 3H); ^{13}C NMR (125 MHz, $CDCl_3$) δ 165.4, 163.5, 137.4, 137.0, 133.2, 131.2, 130.7, 130.5, 129.4, 128.7, 126.7, 126.0, 100.2, 80.6, 21.0; FT-IR (neat) 3060, 2990, 2922, 2856, 1700, 1604, 1570, 1053, 764, 699 cm^{-1}; HRMS (ESI-TOF): calcd for $[C_{17}H_{15}NO +H]^+$ 250.1226: found 250.1227.

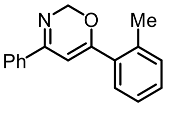

6-(*tert*-Butyl)-4-phenyl-2*H*-1,3-oxazine (2m)

Following the general procedure using 5-(*tert*-butyl)-3-phenylisoxazole **1m** (40.3 mg, 0.20 mmol), purification by PTLC (hexane : acetone : triethylamine = 80 : 20 : 1) afforded the desired product **2m** (29.3 mg, 0.136 mmol, 68%) as a pale yellow oil. ^{1}H NMR (500 MHz, $CDCl_3$) δ 7.82-7.80 (m, 2H), 7.45-7.39 (m, 3H), 5.97 (s, 1H), 5.45 (s, 2H), 1.21 (s, 9H); ^{13}C NMR (125 MHz, $CDCl_3$) δ 176.4, 163.6, 137.3, 130.4, 128.5, 126.6, 94.0, 80.7, 35.8, 27.7; FT-IR (neat) 3060, 2966, 2869, 2787, 1633, 1559, 1349, 1101, 765, 692 cm^{-1}; HRMS (ESI-TOF): calcd for $[C_{14}H_{17}NO +H]^+$ 216.1383: found 216.1380.

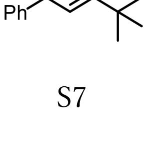

Ethyl 4-phenyl-2*H*-1,3-oxazine-6-carboxylate (2n)

Following the general procedure using ethyl 3-phenylisoxazole-5-carboxylate **1n** (43.4 mg, 0.20 mmol), purification by PTLC (hexane : acetone : triethylamine = 80 : 20 : 1) afforded the desired product **2n** (1st trial: 19.3 mg, 0.0834 mmol, 42%, 2nd trial: 19.4 mg, 0.0840 mmol, 42%, 3rd trial: 18.1 mg, 0.0783 mmol, 39%) as a yellow oil. ^{1}H NMR (500 MHz, $CDCl_3$) δ 7.77-7.76 (m, 2H), 7.49-7.41 (m, 3H), 6.68 (s, 1H), 5.67 (s, 2H), 4.39 (q, *J* = 7.0 Hz, 2H), 1.40 (t, *J* = 7.0 Hz, 3H); ^{13}C NMR (125 MHz, $CDCl_3$) δ 163.7, 163.5, 157.7, 131.7, 131.3, 128.8, 126.3, 95.2, 80.5, 62.4, 14.3; FT-IR (neat) 3059, 2925, 2820, 1704, 1570, 1278, 1049, 765, 691 cm^{-1}; HRMS (ESI-TOF): calcd for $[C_{13}H_{13}NO_3+H]^+$ 232.0968: found 232.0969.

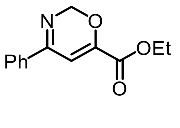

4-Phenyl-6-(trimethylsilyl)-2*H*-1,3-oxazine (2o)

Following the general procedure using 3-phenyl-5-(trimethylsilyl)isoxazole **1o** (43.5 mg, 0.20 mmol), purification by PTLC (hexane : acetone : triethylamine = 80 : 20 : 1) afforded the desired product **2o** (43.5 mg, 0.188 mmol, 91%) as a yellow oil. ^{1}H NMR (400 MHz, $CDCl_3$) δ 7.82-7.80 (m, 2H), 7.44-7.39 (m, 3H), 6.32 (s, 1H), 5.36 (s, 2H), 0.24 (s, 9H); ^{13}C NMR (100 MHz, $CDCl_3$) δ 175.4, 160.8, 137.0, 130.5, 128.6, 126.6, 109.2, 79.1, -2.74; FT-IR (neat) 2965, 2905, 2781, 1639, 1560, 1494, 1402, 1127, 1056, 763, 689 cm^{-1}; HRMS (ESI-TOF): calcd for $[C_{13}H_{17}NOSi+Na]^+$ 254.0972: found 254.0971.

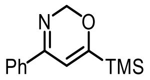

4-Phenyl-6-(triisopropylsilyl)-2*H*-1,3-oxazine (2p)

Following the general procedure using 3-phenyl-5-(triisopropylsilyl)isoxazole **1p** (60.3 mg, 0.20 mmol), purification by PTLC (hexane : acetone : triethylamine = 80 : 20 : 1) afforded the desired product **2p** (50.2 mg, 0.159 mmol, 80%) as a yellow oil. ^{1}H NMR (500 MHz, $CDCl_3$) δ 7.82-7.80 (m, 2H), 7.46-7.40 (m, 3H), 6.40 (s, 1H), 5.33 (s, 2H), 1.31-1.24 (m, 3H), 1.14 (d, *J* = 7.5 Hz, 18H); ^{13}C NMR (125 MHz, $CDCl_3$) δ 172.8, 160.4, 137.1, 130.4, 128.6, 126.7, 112.1, 78.4, 18.6, 10.4; FT-IR (neat) 3060, 2927, 2826, 1629, 1557, 1398, 1117, 760, 691 cm^{-1}; HRMS (ESI-TOF): calcd for $[C_{19}H_{29}NOSi+H]^+$ 316.2091: found 316.2090.

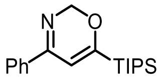

5-Bromo-4,6-diphenyl-2*H*-1,3-oxazine (2q)

Following the general procedure using 4-bromo-3,5-diphenylisoxazole **1q** (60.0 mg, 0.20 mmol), purification by PTLC (hexane : acetone : triethylamine = 80 : 20 : 1) afforded the desired product **2q** (35.2 mg, 0.112 mmol, 56%) as a yellow oil. ^{1}H NMR (400 MHz, $CDCl_3$) δ 7.91 (dd, *J* = 7.6, *J* = 1.2 Hz, 2H), 7.68 (dd, *J* = 7.2 Hz, *J* = 1.6 Hz, 2H), 7.51-7.44 (m, 6H), 5.54 (s, 2H); ^{13}C NMR (100 MHz, $CDCl_3$) δ 165.6, 161.5, 137.1, 132.0, 131.4,

130.4, 130.1, 129.1, 128.3, 128.1, 94.5, 80.1; FT-IR (neat) 2957, 2930, 2871, 1683, 1558, 1524, 1046, 766, 689 cm^{-1}; HRMS (ESI-TOF): calcd for $[C_{16}H_{12}BrNO +H]^+$ 314.0175: found 314.0166.

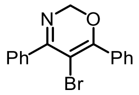

Ethyl 4-(4-chlorophenyl)-6-phenyl-2*H*-1,3-oxazine-5-carboxylate (2r)

Following the general procedure using ethyl 3-(4-chlorophenyl)-5-phenylisoxazole-4-carboxylate **1r** (65.6 mg, 0.20 mmol), purification by PTLC (hexane : acetone : triethylamine = 80 : 20 : 1) afforded the desired product **2r** (26.7 mg, 0.078 mmol, 39%) as a yellow oil. ^{1}H NMR (400 MHz, $CDCl_3$) δ 7.75-7.73 (m, 2H), 7.56-7.52 (m, 3H), 7.47-7.43 (m, 2H), 7.39-7.37 (m, 2H), 5.57 (s, 2H), 3.86 (q, *J* = 7.2 Hz, 2H), 0.81 (t, *J* = 7.2, 3H); ^{13}C NMR (100 MHz, $CDCl_3$) δ 171.1, 165.16, 164.87, 137.0, 135.9, 132.2, 131.9, 130.7, 129.3, 128.4, 128.1, 108.1, 80.9, 60.8, 13.5; FT-IR (neat) 3062, 2980, 1715, 1598, 1489, 1396, 1251, 1158, 1015, 840, 694 cm-1; HRMS (ESI-TOF): calcd for $[C_{19}H_{16}ClNO_3 +H]$+ 342.0891: found 342.0896.

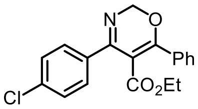

4-Phenyl-2*H*-benzo[*e*][1,3]oxazine (2s)

Following the general procedure using 3-phenylbenzo[*d*]isoxazole **1s** (39.0 mg, 0.20 mmol), purification by PTLC (hexane : acetone : triethylamine = 80 : 20 : 1) afforded the desired product **2s** (25.4 mg, 0.121 mmol, 61%) as a yellow solid. ^{1}H NMR (500 MHz, $CDCl_3$) δ 7.61-7.59 (m, 2H), 7.51-7.49 (m, 3H), 7.48-7.40 (m, 1H), 7.39-7.25 (m, 1H), 7.00-6.96 (m, 2H), 5.61 (s, 2H); ^{13}C NMR (125 MHz, $CDCl_3$) δ 164.8, 157.0, 136.8, 133.6, 130.1, 129.0, 128.5, 128.0, 121.5, 119.0, 116.7, 79.5; FT-IR (neat) 3060, 2900, 2821, 1606, 1336, 1250, 1077, 843, 768, 695 cm^{-1}; HRMS (ESI-TOF): calcd for $[C_{14}H_{11}NO +H]^+$ 210.0913: found 210.0913.

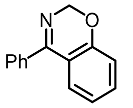

Ethyl 2*H*-benzo[*e*][1,3]oxazine-4-carboxylate (2t)

Following the general procedure using 3-phenylbenzo[*d*]isoxazole **1t** (38.2 mg, 0.20 mmol), purification by PTLC (hexane : acetone : triethylamine = 80 : 20 : 1) afforded the desired product **2t** (36.1 mg, 0.176 mmol, 88%) as a yellow solid. ^{1}H NMR (400 MHz, $CDCl_3$) δ 7.77 (dd, *J* = 7.8 Hz, 1.4 Hz, 1H), 7.38 (dt, *J* = 8.0 Hz, 1.2 Hz, 1H), 7.00 (dt, *J* = 7.6 Hz, 0.8 Hz, 1H), 6.88 (d, *J* = 8.0 Hz, 1H), 5.63 (s, 2H), 4.42 (q, *J* = 7.2 Hz, 2H), 1.41 (t, *J* = 7.2 Hz, 3H); ^{13}C NMR (100 MHz, $CDCl_3$) δ 163.3, 156.2, 156.1, 134.6, 127.3, 122.1, 116.5, 116.2 79.1, 62.4 14.2; FT-IR (neat) 3072, 2982, 2848, 1731, 1624, 1486, 1373, 1206, 1154, 1130, 1022, 756 cm^{-1}; HRMS (ESI-TOF): calcd for $[C_{11}H_{11}NO_3 +H]^+$ 206.0812: found 206.0813.

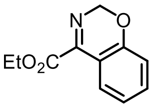

(2*H*-benzo[*e*][1,3]oxazin-4-yl)methanesulfonamide (2u)

Following the general procedure using benzo[*d*]isoxazol-3-ylmethanesulfonamide **1u** (42.4 mg, 0.20 mmol), purification by column chromatography on silica gel (hexane : AcOEt = 7 : 3), then preparative HPLC (hexane : AcOEt = 70 : 30 to 50 : 50) afforded the desired product **2u** (7.9 mg, 0.035 mmol, 17%) as a white solid; Mp 143-144 ºC; ^{1}H NMR (500 MHz, CD_3CN) δ 7.54 (dd, *J* = 8.0 Hz, 1.5 Hz, 1H), 7.43 (td, *J* = 7.4 Hz, 1.5 Hz, 1H), 7.04 (td, *J* = 7.8 Hz, 1,0 Hz), 6.90 (dd, *J* = 8.0 Hz, 0.5 Hz,1H), 5.57 (s, 2H), 5.56 (brs, 2H), 5,56 (s, 2H); ^{13}C NMR (125 MHz, CD_3CN) δ 158.0, 156.6, 135.2, 127.3, 122.7, 119.1, 117.1, 79.9, 58.5; FT-IR (neat) 2922, 2851, 1606, 1541, 1457, 1308, 1276, 1139, 1019, 955, 765 cm^{-1}; HRMS (ESI-TOF): calcd for $[C_9H_{10}N_2O_3S +Na]^+$ 249.0304: found 249.0305.

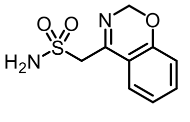

4-(4-(4-phenoxyphenyl)-1*H*-1,2,3-triazol-1-yl)-2*H*-benzo[*e*][1,3]oxazine (2v)

Following the general procedure using 3-(4-(4-phenoxyphenyl)-1*H*-1,2,3-triazol-1-yl)benzo[*d*]isoxazole[19] **1v** (70.9 mg, 0.20 mmol), purification by GPC afforded the desired product **2v** (11.9 mg, 0.032 mmol, 16%) as a colorless oil. ^{1}H NMR (500 MHz, $CDCl_3$) δ 8.53 (s, 1H), 8.19 (dd, J = 8.0 Hz, 1.5 Hz, 1H), 7.89 (d, *J* = 8.5 Hz, 1H), 7.53-7.50 (m, 1H), 7.39-7.36 (m, 2H), 7.16-7.03 (m, 7H), 5.68 (s, 2H); ^{13}C NMR (125 MHz, $CDCl_3$) δ 158.1, 157.8, 156.9, 150.7, 146.8, 135.2, 130.0, 129.0, 127.7, 124.8, 123.8, 122.4, 119.4, 119.2, 118.2, 116.8, 113.9, 78.5; FT-IR (neat) 2919, 2054, 1645, 1488, 1277, 1238, 1006, 752 cm^{-1}; HRMS (ESI-TOF): calcd for $[C_{22}H_{16}N_4O_2 +Na]^+$ 391.1165: found 391.1164.

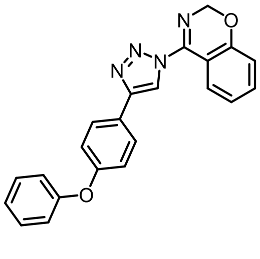

4,6-Diphenyl-2*H*-1,3-thiazine (4a)

Following the general procedure using 3,5-diphenylisothiazole (25.3 mg, 0.10 mmol), purification by PTLC (hexane : acetone : triethylamine = 90 : 10 : 1) afforded the desired product **4a** (17.6 mg, 0.035 mmol, 35%) as a colorless oil. ^{1}H NMR (500 MHz, $CDCl_3$) δ 7.89-7.87 (m, 2H), 7.77-7.75 (m, 2H), 7.49-7.44 (m, 6H), 7.25 (s, 1H), 4.84 (s, 2H); ^{13}C NMR (125 MHz, $CDCl_3$) δ 165.9, 152.6, 138.6, 136.9, 130.9, 130.4, 129.0, 128.7, 128.1, 127.2, 114.8, 50.5; FT-IR (neat) 3057, 2869, 2818, 1602, 1571, 1530, 1487, 1066, 755, 725 cm^{-1}; HRMS (ESI-TOF): calcd for $[C_{16}H_{13}NS +H]^+$ 252.0841: found 252.0846.

4,6-Diphenyl-1-tosyl-1,2-dihydropyrimidine (4b)

Following the general procedure using 3,5-diphenyl-1-tosyl-1*H*-pyrazole **3b** (74.9 mg, 0.20 mmol), purification by PTLC (hexane : acetone : triethylamine = 90 : 10 : 1) afforded the desired product **4b** (14.8 mg, 0.038 mmol, 19%) as a colorless oil. ^{1}H NMR (500 MHz, $CDCl_3$) δ 7.72 (dd, *J* = 7.5 Hz, *J* = 1.5 Hz, 2H), 7.51-7.45 (m, 5H), 7.40-7.36 (m, 3H), 7.33-7.30 (m, 2H), 7.01 (d, *J* = 8.5 Hz, 2H), 6.62 (s, 1H), 5.53 (s, 2H), 2.14 (s, 3H); ^{13}C NMR (125 MHz, $CDCl_3$) δ 162.9, 148.8, 144.1, 136.3, 135.6, 135.3, 130.8, 130.3, 129.1, 128.5, 128.3, 128.2, 127.9, 126.5, 113.5, 65.4, 21.3; FT-IR (neat) 3058, 2884, 1622, 1358, 1167, 764, 750, 717 cm^{-1}; HRMS (ESI-TOF): calcd for $[C_{23}H_{20}N_2O_2S + Na]^+$ 411.1138: found 411.1138.

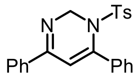

1-Methoxy-1,2-dihydroquinazoline (4c)

Following the general procedure using indazole **3c** (29.6 mg, 0.20 mmol), purification by PTLC (hexane : acetone : triethylamine = 90 : 10 : 1) afforded the desired product **4c** (12.5 mg, 0.0770 mmol, 39%) as a yellow oil. ^{1}H NMR (500 MHz, $CDCl_3$) δ 8.12 (s, 1H), 7.38 (dt, *J* = 7.8 Hz, *J* = 1.3 Hz, 1H), 7.18 (dd, *J* = 7.5 Hz, *J* = 1.1 Hz, 1H), 7.09 (d, *J* = 8.2 Hz, 1H), 6.98 (dt, *J* = 10 Hz, *J* = 3.7 Hz, 1H), 4.88 (d, *J* = 2.1 Hz, 2H), 3.73 (s, 2H); ^{13}C NMR (125 MHz, $CDCl_3$) δ 159.2, 148.1, 136.3, 133.3, 129.4, 128.70, 128.66, 127.2, 122.3, 119.3, 114.1, 76.4, 67.6; FT-IR (neat) 3006, 2866, 1623, 1358, 1167, 750, 717 cm^{-1}; HRMS (ESI-TOF): calcd for $[C_9H_{10}N_2O + H]^+$ 163.0866: found 163.0866.

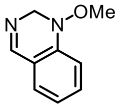

1-(Benzyloxy)-1,2-dihydroquinazoline (4d)

Following the general procedure using indazole **3d** (44.9 mg, 0.20 mmol), purification by PTLC (hexane : acetone : triethylamine = 90 : 10 : 1) afforded the desired product **4d** (14.8 mg, 0.062 mmol, 31%) as a yellow oil. ^{1}H NMR (500 MHz, $CDCl_3$) δ 8.10 (s, 1H), 7.45 (dd, *J* = 8.2 Hz, *J* = 1.7 Hz, 2H), 7.40-7.32 (m, 4H), 7.16 (dd, *J* = 7.5 Hz, *J* = 1.3 Hz, 1H), 7.03 (d, *J* = 8.1 Hz, 1H), 6.97 (dt, *J* = 10.3 Hz, *J* = 3.8 Hz, 1H), 4.87 (s, 2H), 4.83 (d, *J* = 2.1 Hz, 2H); ^{13}C NMR (125 MHz, $CDCl_3$) δ 159.2, 148.1, 136.3, 133.3, 129.4, 128.70, 128.66, 127.2, 122.3, 119.2, 114.1, 76.4, 67.6; FT-IR (neat) 3006, 2969, 1733, 1258, 1065, 913, 748 cm^{-1}; HRMS (ESI-TOF): calcd for $[C_{15}H_{14}N_2O + H]^+$ 239.1179: found 239.1179.

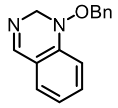

Quinazolin-1(2*H*)-yl 2,4,6-trimethylbenzoate (4e)

Following the general procedure using indazole **3e** (56.0 mg, 0.20 mmol), purification by PTLC (hexane : acetone : triethylamine = 90 : 10 : 1) afforded the desired product **4e** (20.2 mg, 0.0686 mmol, 34%) as a yellow

oil. ^{1}H NMR (500 MHz, $CDCl_3$) δ 8.3 (s, 1H), 7.73 (d, *J* = 8.9 Hz, 1H), 7.67 (d, *J* = 8.6 Hz, 1H), 7.33-7.30 (m, 1H), 7.12-7.09 (m, 1H), 6.80 (s, 2H), 6.53 (s, 2H), 2.24 (s, 3H), 2.15 (s, 6H); ^{13}C NMR (125 MHz, $CDCl_3$) δ169.2, 149.7, 140.3, 136.0, 128.9, 128.8, 127.2, 125.1, 122.7, 122.2, 120.8, 118.2, 73.5, 21.3, 19.9, ; FT-IR (neat) 3060, 2943, 1955, 1604, 1462, 1362, 1007, 957, 765, 695 cm^{-1}; HRMS (ESI-TOF): calcd for $[C_{18}H_{18}N_2O_2 +H]^+$ 295.3615: found 295.3610.

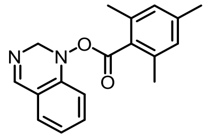

1-Methyl-1*H*-indazole (4l)

Following the general procedure using 1*H*-indazole (23.6 mg, 0.20 mmol), purification by PTLC (hexane : acetone = 8 : 2) afforded the desired product **4l** (19.6 mg, 0.148 mmol, 74%) as a white solid. Spectroscopic data are in agreement with the previous report.[21]

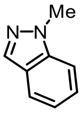

To a mixture of 1,2-azole (0.20 mmol, 1.0 equiv.) in 1,2-dichloroethane (4.0 mL), diethylzinc (*ca.* 1 mol/L in toluene) (0.60 mL, 3.0 equiv.) was added at 10 ºC under an argon atmosphere. After stirred at the same temperature for 10 min, diiodomethane (96.5 μL, 6.0 equiv.) was added to the mixture. The reaction mixture was stirred at the same temperature for 4 h. The reaction was traced by TLC analysis. After completion of the reaction, saturated NH_4Cl aq. was added to the mixture. After being stirred for 30 min, the residue was poured into water. The aqueous layer was extracted with two portions of CH_2Cl_2. The combined extract was dried over Na_2SO_4 and concentrated *in vacuo*. The residue was purified by PTLC which was deactivated by 1% triethylamine to afford the desired product.

4,6-Diphenyl-5,6-dihydro-2*H*-1,3-oxazine (4f)

Following the general procedure using 3,5-diphenyl-4,5-dihydroisoxazole **3f** (44.7 mg, 0.20 mmol), diethylzinc (0.30 mL, 1.5 equiv.), and diiodomethane (48.3 μL, 3.0 equiv.), purification by PTLC (hexane : acetone : triethylamine = 80 : 20 : 1) afforded the desired product **4f** (38.0 mg, 0.160 mmol, 80%) as a yellow oil. ^{1}H NMR (400 MHz, $CDCl_3$) δ 7.84-7.81 (m, 2H), 7.44-7.34 (m, 8H), 5.66 (dd, *J* = 16.0 Hz, *J* = 2.4 Hz, 1H), 5.44 (dt, *J* = 16.0 Hz, *J* = 3.4 Hz, 1H), 4.72 (dd, *J* = 10.4 Hz, *J* = 3.6 Hz, 1H), 2.99 (dt, *J* = 17.2 Hz, *J* = 3.2 Hz, 1H), 2.83-2.75 (s, 1H); ^{13}C NMR (100 MHz, $CDCl_3$) δ 161.9, 141.4, 138.4, 130.5, 128.8, 128.6, 128.2, 126.0, 125.9, 81.9, 73.2, 34.6; FT-IR (neat) 3060, 2943, 2834, 1736, 1642, 1371, 1089, 1065, 753, 693 cm^{-1}; HRMS (ESI-TOF): calcd for $[C_{16}H_{15}NO +Na]^+$ 260.1046: found 260.1049.

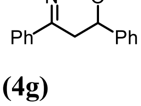

6-Phenyl-4-(*p*-tolyl)-5,6-dihydro-2*H*-1,3-oxazine (4g)

Following the general procedure using 5-phenyl-3-(*p*-tolyl)-4,5-dihydroisoxazole **3g** (47.5 mg, 0.20 mmol), diethylzinc (0.30 mL, 1.5 equiv.), and diiodomethane (48.3 μL, 3.0 equiv.), purification by PTLC (hexane :

acetone : triethylamine = 80 : 20 : 1) afforded the desired product **4g** (15.2 mg, 0.0604 mmol, 30%) as a yellow oil. ^{1}H NMR (400 MHz, $CDCl_3$) δ 7.71 (d, *J* = 7.7, 2H), 7.44-7.32 (m, 5H), 7.22 (d, *J* = 8.0 Hz, 2H), 5.64 (dd, *J* = 16.0 Hz, *J* = 2.4 Hz, 1H), 5.43 (dt, *J* = 16.0 Hz, *J* = 3.2 Hz, 1H), 4.71 (dd, *J* = 10.4 Hz, *J* = 3.6 Hz, 1H), 2.97 (dt, *J* = 17.5 Hz, *J* = 3.0 Hz, 1H), 2.81-2.72 (m, 1H), 2.39 (s, 3H); ^{13}C NMR (100 MHz, $CDCl_3$) δ 161.7, 141.5, 140.7, 135.7, 129.3, 128.8, 128.1, 125.99, 125.95, 81.9, 73.3, 34.5, 21.5; FT-IR (neat) 3030, 2943, 2836, 1642, 1372, 1080, 1021, 758, 699 cm^{-1}; HRMS (ESI-TOF): calcd for $[C_{17}H_{17}NO + Na]^+$ 274.1202: found 274.1204.

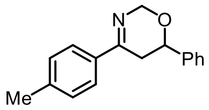

6-Phenyl-4-(*o*-tolyl)-5,6-dihydro-2*H*-1,3-oxazine (4h)

Following the general procedure using 3,5-diphenylisoxazole **3h** (47.5 mg, 0.20 mmol), diethylzinc (0.30 mL, 1.5 equiv.), and diiodomethane (48.3 μL, 3.0 equiv.), purification by PTLC (hexane : acetone : triethylamine = 80 : 20 : 1) afforded the desired product **4h** (28.7 mg, 0.114 mmol, 57%) as a yellow oil. ^{1}H NMR (400 MHz, $CDCl_3$) δ 7.41-7.22 (m, 9H), 5.63 (d, *J* = 16 Hz, 2H), 5.42 (dt, *J* = 15.9 Hz, *J* = 3.4 Hz, 1H), 4.77 (t, *J* = 7.0 Hz, 1H), 2.76-2.74 (m, 2H), 2.45 (s, 3H); ^{13}C NMR (100 MHz, $CDCl_3$) δ 166.0, 141.2, 140.2, 135.2, 131.2, 128.8, 128.1, 127.0, 126.0, 125.9, 81.7, 73.1, 37.9, 20.3; FT-IR (neat) 3062, 3030, 2923, 1587, 1494, 1338, 1030, 895, 757, 699 cm^{-1}; HRMS (ESI-TOF): calcd for $[C_{17}H_{17}NO + Na]^+$ 274.1202: found 274.1202.

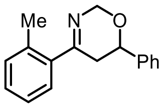

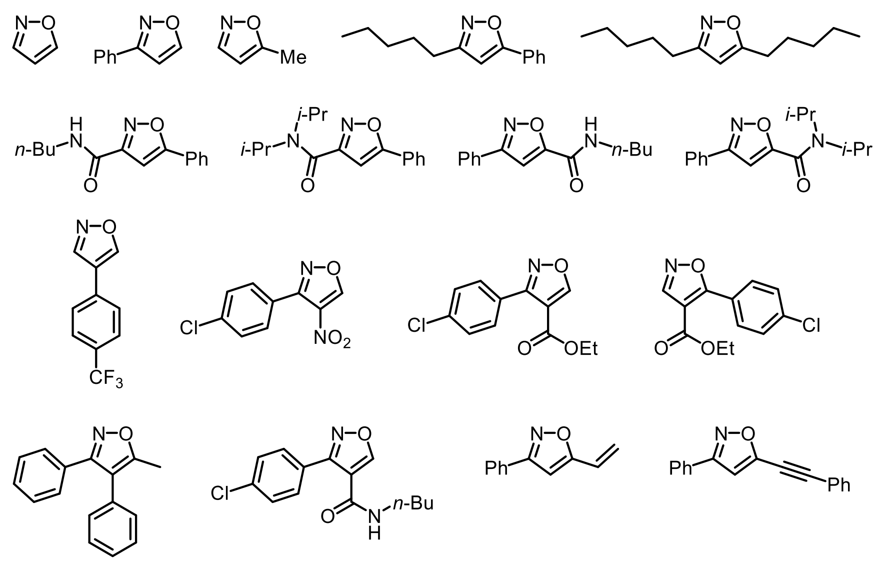

Figure S1. Examples of unsccessful substrate for methylene insertion

6. Computational Methods

All calculations were performed with the Gaussian 16 program package (revision C.01).[20] The DFT calculations were carried out using the B3LYP functional[21] with tight SCF convergence and ultrafine integration grids. Grimmes's DFT-D3 dispersion correction was used to express the van der waals interaction.[22] A standard 6-31G(d,p)[23] basis set was used for carbon, hydrogen, oxygen, and nitrogen and LANL2DZ[24] basis set was used for iodine and zinc. Each of the stationary points was adequately characterized by normal coordinate analysis (no imaginary frequency for an equilibrium structure and one imaginary frequency for a transition-state structure). Intrinsic reaction coordinates (IRC)[25] were calculated to verify the relevance of transition-state structures. The solvent effect of 1,2-dichloroethane was taken into account by the polarizable continuum model (PCM)[26] for DFT calculations. In all calculations, the temperature was set to 298.15 K. All the optimized geometries are available as Supporting Information in .xyz format.

Table S2. Calculated electronic energy, enthalpy, Gibbs free energy (Hartree), and imaginary frequency (cm^{-1})

Compound	Electronic Energy	Enthalpy	Free energy	Imaginary Frequency
$MeZnCH_2I$	-156.214362	-156.145241	-156.187691	–
$EtZnCH_2I$	-195.525967	-195.426530	-195.471422	–
$IZnCH_2I$	-127.761206	-127.726916	-127.769823	–
ICH_2ZnCH_2I	-167.001494	-166.939879	-166.987109	–
IZnMe	-116.977728	-116.935664	-116.973846	–
**1a**	-708.189847	-707.955678	-708.011345	–
**Int1**	-864.416566	-864.111131	-864.191022	–
**TS1**	-864.401633	-864.097065	-864.175991	-311.78
**Int2**	-864.447054	-864.139685	-864.216112	–
**Int3**	-864.447345	-864.139862	-864.217652	–
**TS2**	-864.435687	-864.130809	-864.207425	-495.93
**Int4**	-864.506519	-864.199303	-864.278470	–
**Int5**	-747.506653	-747.244010	-747.304813	–
**TS3**	-747.498251	-747.236306	-747.293554	-319.78
**2a**	-747.521979	-747.257413	-747.314283	–
**Int1-$EtZnCH_2I$**	-903.729678	-903.393895	-903.475749	–
**TS1-$EtZnCH_2I$**	-903.714621	-903.379612	-903.459448	-327.35
**Int2-$EtZnCH_2I$**	-903.759848	-903.421974	-903.500714	–
**Int1-$IZnCH_2I$**	-835.972810	-835.701668	-835.780826	–
**TS1-$IZnCH_2I$**	-835.945957	-835.676129	-835.752669	-351.76
**Int2- $IZnCH_2I$**	-836.000151	-835.727361	-835.803686	–
**Int1-ICH_2ZnCH_2I**	-875.205527	-874.907616	-874.993643	–
**TS1-ICH_2ZnCH_2I**	-875.189872	-874.892846	-874.974563	-322.41

**TS2-ICH$_2$ZnCH$_2$I**	-875.237979	-874.937977	-875.018127	–
**1w**	-246.989268	-245.984746	-246.015475	–
**Int1-w**	-402.269061	-402.135309	-402.194583	–
**TS1-w**	-402.247364	-402.114272	-402.171376	-328.71
**Int2-w**	-402.293992	-402.158599	-402.213627	–
**TS2-w**	-402.213627	-402.157329	-402.213303	-363.22
**Int3-w**	-285.365818	-285.275210	-285.312978	–
**TS3-w**	-285.354638	-285.264400	-285.298254	-385.43
**2w**	-285.377312	-285.284306	-285.317987	–
**1z**	-324.704265	-324.583051	-324.621912	–
**Int1-z**	-480.923149	-480.730399	-480.792639	–
**TS1-z**	-480.906723	-480.715136	-480.780054	-314.19
**Int2-z**	-480.954695	-480.760036	-480.823295	–
**TS2-z**	-480.943745	-480.751884	-480.814972	-470.74
**Int3-z**	-364.023737	-363.874318	-363.919657	–
**TS3-z**	-364.012573	-363.863738	-363.906210	-349.28
**2z**	-364.036559	-363.885145	-363.926217	–
**3a**	-1031.207388	-1030.975755	-1031.031664	–
**Int1-S**	-1187.421808	-1187.118817	-1187.199149	–
**TS1-S**	-1187.403507	-1187.101316	-1187.179144	-319.40
**Int2-S**	-1187.445759	-1187.140795	-1187.218681	–
**Int3-S**	-1187.446635	-1187.141715	-1187.217868	–
**TS2-S**	-1187.420472	-1187.117726	-1187.194910	-343.33
**Int4-S**	-1187.455266	-1187.150589	-1187.231115	–
**Int5-S**	-1070.480192	-1070.219587	-1070.281321	–
**TS3-S**	-1070.479932	-1070.220337	-1070.279128	-84.76
**4a**	-1070.508811	-1070.247285	-1070.305467	–
**3b**	-1507.329353	-1506.954642	-1507.034972	–
**Int1-N**	-1663.543671	-1663.098418	-1663.199750	–
**TS-N**	-1663.522214	-1663.076395	-1663.174965	-336.96
**Int2-N**	-1663.559298	-1663.111048	-1663.210162	–
**Int3-N**	-1663.561764	-1663.113480	-1663.211549	–
**TS2-N**	-1663.536169	-1663.090831	-1663.190033	-490.87
**Int4-N**	-1663.592810	-1663.145578	-1663.244863	–
**Int5-N**	-1546.619010	-1546.216268	-1546.300904	–
**TS3-N**	-1546.616697	-1546.616702	-1546.295625	-143.27
**4b**	-1546.657636	-1546.252648	-1546.331383	–
**3f**	-709.402311	-709.144996	-709.202510	–

**Int6**	-865.616690	-865.287977	-865.369093	–
**TS4**	-865.597413	-865.269364	-865.348711	-333.32
**Int7**	-865.645883	-865.314841	-865.393653	–
**Int8**	-865.651082	-865.320049	-865.398900	–
**TS5**	-865.633439	-865.305289	-865.384454	-392.38
**Int9**	-865.688933	-865.359204	-865.439485	–
**TS6**	-865.681941	-865.353326	-865.431636	-171.56
**4f**	-748.753702	-748.465288	-748.522860	–
**3c**	-494.34184	-494.182292	-494.226469	–
**Int1-OMe**	-650.561873	-650.330971	-650.399942	–
**TS1-OMe**	-650.541591	-650.331578	-650.380648	-320.86
**Int2-OMe**	-650.586444	-650.353308	-650.419220	–
**Int3-OMe**	-533.586331	-533.399736	-533.446028	–
**TS2-OMe**	-533.574593	-533.389973	-533.436026	-340.62
**Int4-OMe**	-533.614315	-533.427564	-533.477974	–
**TS3-OMe**	-533.610812	-533.424687	-533.472082	-183.13
**4c**	-533.661876	-533.472653	-533.519595	–
**3m**	-1198.834416	-1198.581262	-1198.644485	–
**Int1-Ts**	-1355.048913	-1354.724454	-1354.810522	–
**TS1-Ts**	-1355.025437	-1354.701507	-1354.784391	-335.25
**Int2-Ts**	-1355.064883	-1354.738236	-1354.819992	–
**Int3-Ts**	-1238.077538	-1237.797218	-1237.861678	–
**TS2-Ts**	-1238.065560	-1237.786970	-1237.850800	-502.10
**Int4-Ts**	-1238.108556	-1237.827897	-1237.897304	–
**TS3-Ts**	-1238.105719	-1237.825962	-1237.892994	-182.29
**4m**	-1238.164653	-1237.881129	-1237.945237	–

6-1. Examination of zinc carbenoids

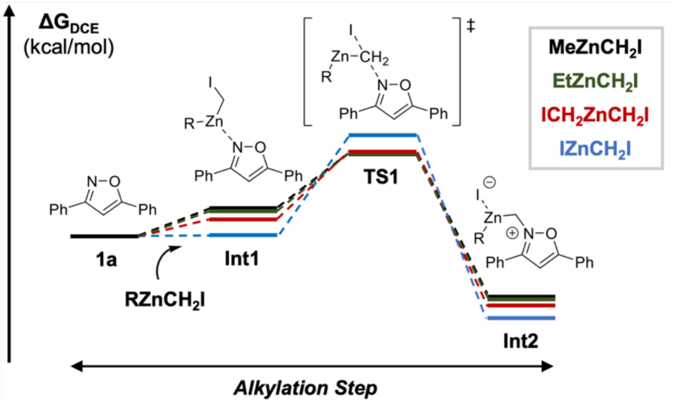

entry	Halomethylzinc spieces	$\Delta G^{\ddagger}$ (kcal/mol)
1	$MeZnCH_2I$	14.5
2	$EtZnCH_2I$	14.6
3	ICH_2ZnCH_2I	15.0
4	$IZnCH_2I$	17.9

Figure S2. Comparison of activation energies in the first step of the methylene insertion.

The combination of diiodomethane and diethyl zinc yields Furukawa reagent ($EtZnCH_2I$). The calculated activation energy using model zinc carbenoid ($MeZnCH_2I$) was similar to the energy using Furukawa reagent (entry 1: 14.5 kcal/mol vs. entry 2: 14.6 kcal/mol). On the other hand, the activation energies using other zinc carbenoid species such as Wittig reagent (ICH_2ZnCH_2I: entry 3) and Simmons-Smith reagent ($IZnCH_2I$: entry 4) were higher than Furukawa reagent. Although Simmons-Smith reagent can be prepared by using zinc–copper couple (Zn(Cu)) and diiodomethane, the combination did not give the product (entry 7, Table S1). The calculated higher activation energy supports this result.

6-2. Energy profiles of isoxazoles having different substituents

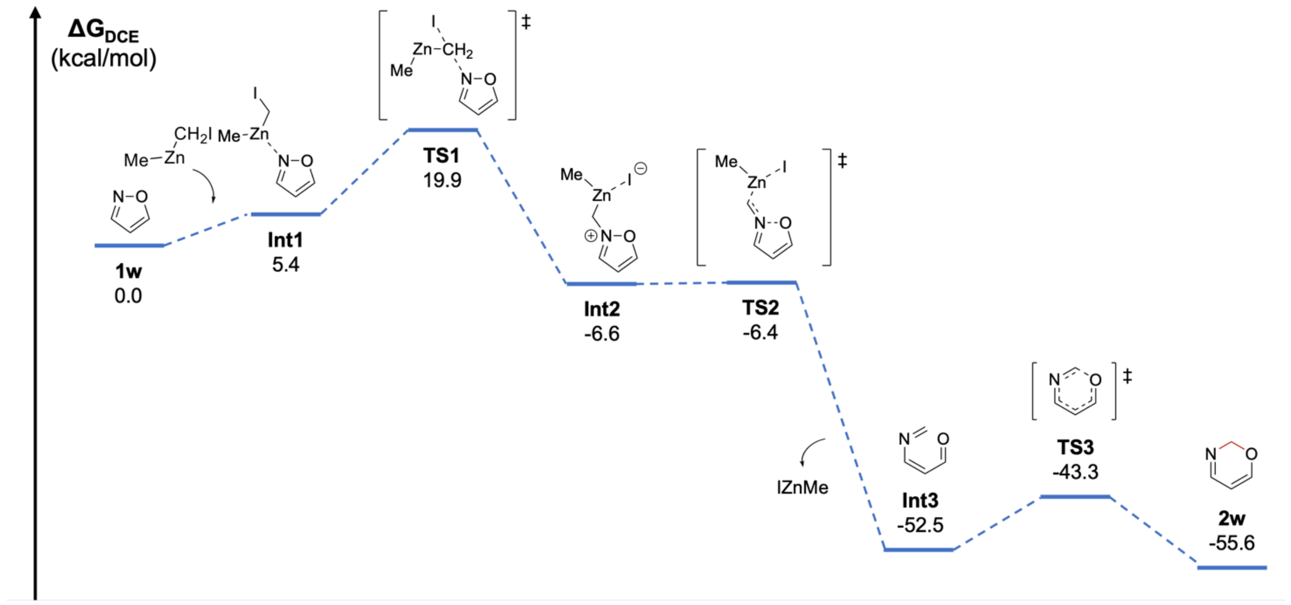

Figure S3. Gibbs free energy profile of the ring expansion of isoxazole **1w**. DFT calculation by B3LYP-3D/LANL2DZ for Zn, I and 6-31G(d,p) for other elements in 1,2-dichloromethane (PCM).

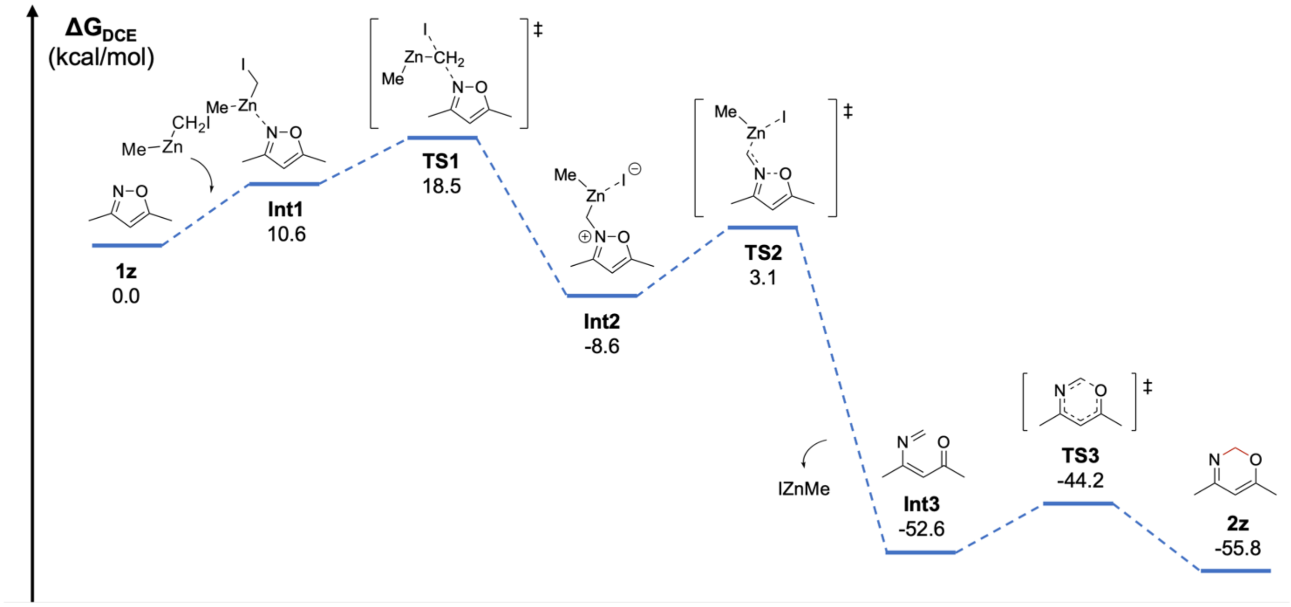

Figure S4. Gibbs free energy profile of the ring expansion of isothiazole **1z**. DFT calculation by B3LYP-3D/LANL2DZ for Zn, I and 6-31G(d,p) for other elements in 1,2-dichloromethane (PCM).

6-3. Energy profiles of other 1,2-azoles

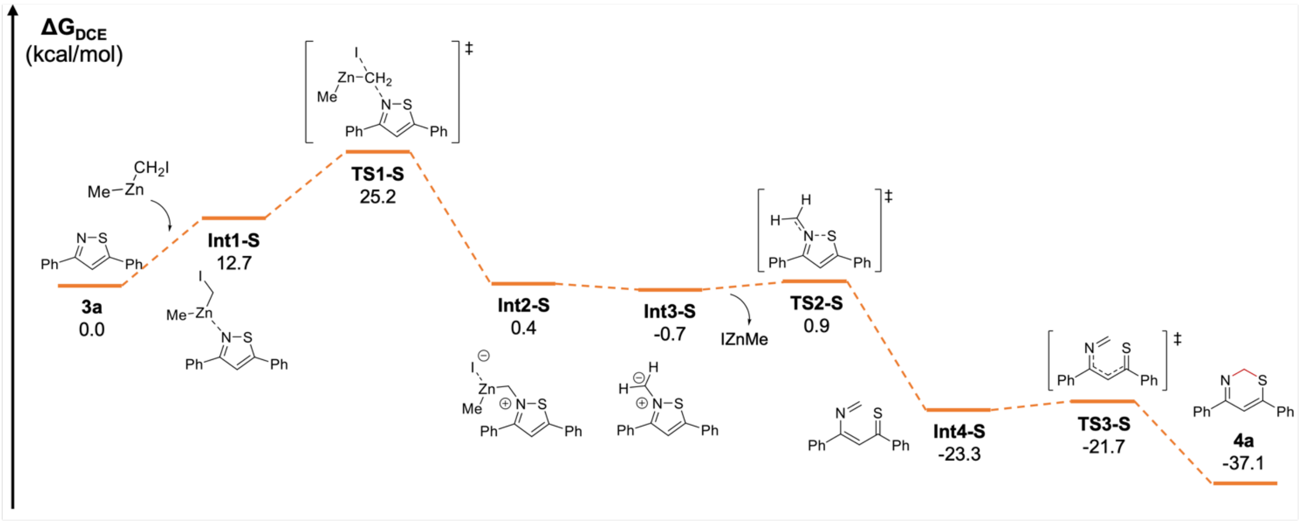

Figure S5. Gibbs free energy profile of the ring expansion of isothiazole **3a**. DFT calculation by B3LYP-3D/LANL2DZ for Zn, I and 6-31G(d,p) for other elements in 1,2-dichloromethane (PCM).

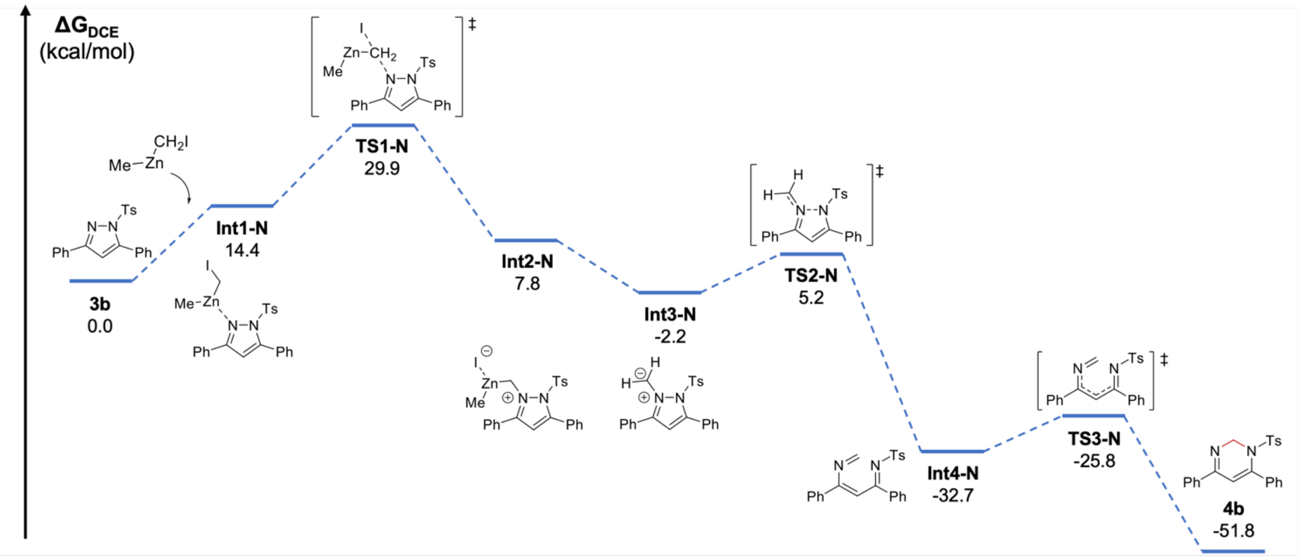

Figure S6. Gibbs free energy profile of the ring expansion of pyrazole **3b**. DFT calculation by B3LYP-3D/LANL2DZ for Zn, I and 6-31G(d,p) for other elements in 1,2-dichloromethane (PCM).

6-4. Energy profiles of indazoles

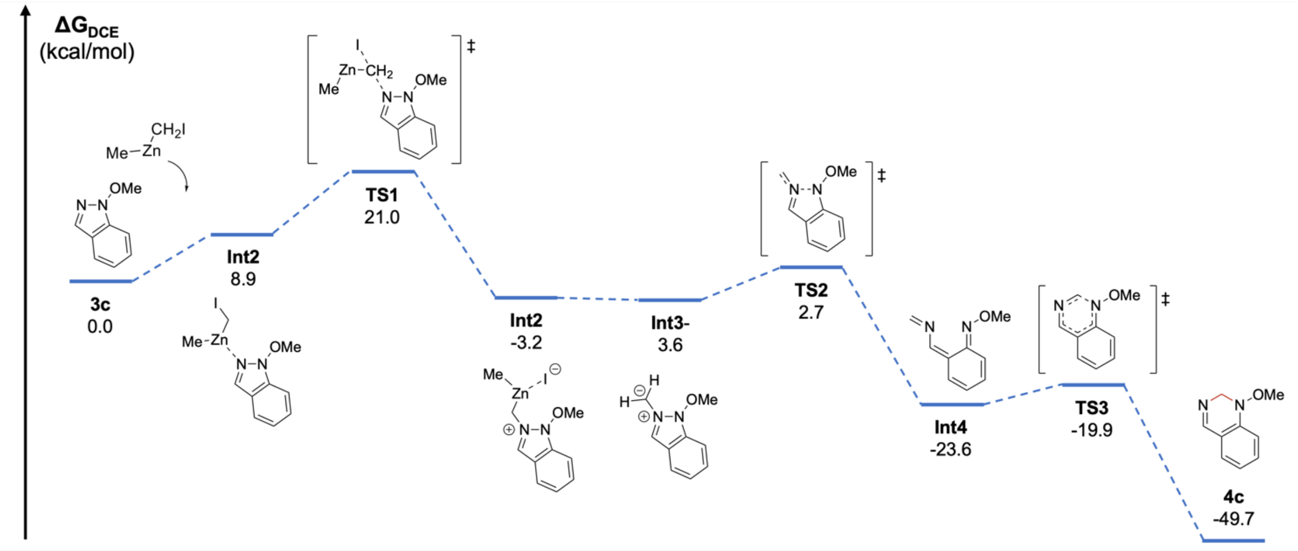

Figure S7. Gibbs free energy profile of the ring expansion of pyrazole **3c**. DFT calculation by B3LYP-3D/LANL2DZ for Zn, I and 6-31G(d,p) for other elements in 1,2-dichloromethane (PCM).

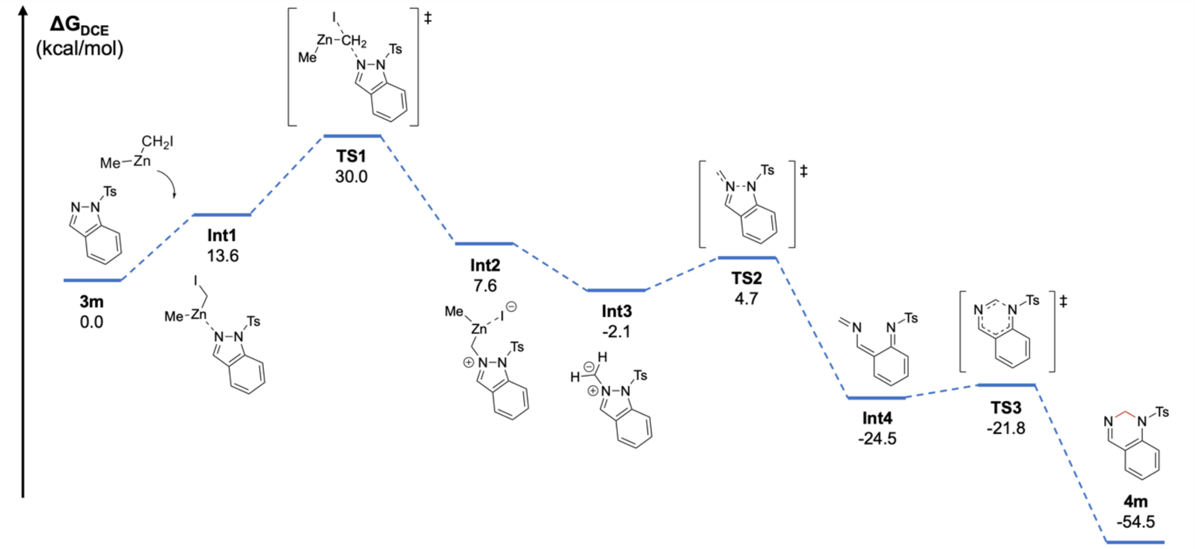

Figure S8. Gibbs free energy profile of the ring expansion of pyrazole **3m**. DFT calculation by B3LYP-3D/LANL2DZ for Zn, I and 6-31G(d,p) for other elements in 1,2-dichloromethane (PCM).

Table S2. Comparison of substituents at the N-1 position of indazole[a)]

indazole 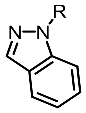	activation energy: $\Delta G^{\ddagger}_{TS1}$ (kcal/mol)	yield (%)
R = OMe	21.0	31
R = Ts	30.0	0

[a] *N*-Alkoxy pyrazoles have not reported and there is no established method to synthesize them. We tentatively calculated the energy of 1-methoxy-3,5-diphenyl-1*H*-pyrazole. The calculated energy barrier was $\Delta G^{\ddagger}_{TS1}$ = 27.2 kcal/mol (*N*-Ts pyrazole: $\Delta G^{\ddagger}_{TS1}$ = 29.9 kcal/mol). The compound has potential to be applicable to this methylene insertion.

Cartesian coordinates and geometry

All coordinates were calculated at the level of B3LYP-D3/LANL2DZ for I, Zn and 6-31G(d,p) for other elements.

MeZnCH$_2$I

Zn	-1.871043	0.308619	0.005369
C	-0.043300	1.298696	0.005289
H	0.196009	1.882220	0.896781
H	0.179908	1.913712	-0.869022
C	-3.409198	-1.034723	0.002115
H	-3.016412	-2.058526	0.012351
H	-4.039961	-0.930600	-0.888821
H	-4.055741	-0.918786	0.880147
I	1.652135	-0.204459	-0.036987

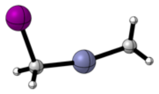

EtZnCH$_2$I

Zn	-1.504707	-0.705629	0.000002
C	0.430002	-1.442205	-0.000007
H	0.749735	-1.996631	-0.884771
H	0.749740	-1.996642	0.884749
C	-3.377117	0.156857	-0.000000
H	-3.933993	-0.203151	-0.876220
H	-3.933999	-0.203148	0.876217
I	1.871684	0.316951	0.000000
C	-3.339122	1.694455	-0.000003
H	-4.343831	2.143645	-0.000010
H	-2.814148	2.085883	0.879526
H	-2.814138	2.085880	-0.879526

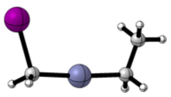

IZnCH$_2$I

Zn	-0.426404	0.710594	0.000001
C	1.502493	1.372963	-0.000002
H	1.782913	1.940427	0.887746
H	1.782909	1.940420	-0.887757
I	2.937781	-0.348022	0.000000
I	-2.933793	-0.282855	-0.000000

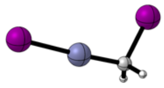

ICH$_2$ZnCH$_2$I

Zn	0.000000	0.000000	0.029957
C	1.675429	1.163501	-0.204307
H	1.835822	1.955761	0.528429
H	1.802600	1.587311	-1.202147
C	-1.675429	-1.163500	-0.204307
H	-1.802599	-1.587310	-1.202147
H	-1.835821	-1.955760	0.528429
I	3.535946	-0.096838	0.027362
I	-3.535947	0.096838	0.027362

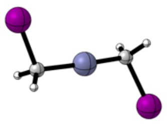

IZnMe

Zn	-1.405460	-0.004625	0.011062
I	1.382480	-0.017909	0.037056
C	-3.421946	0.142489	-0.247909
H	-3.945927	0.045966	0.707513
H	-3.771996	-0.643985	-0.923530
H	-3.665790	1.115198	-0.686545

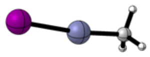

1a

C	1.112369	-0.358750	-0.039427
C	0.008779	0.541892	0.035229
C	-1.096842	-0.260488	-0.029901

N	0.697973	-1.611313	-0.144971
H	0.035635	1.614098	0.141494
O	-0.696041	-1.552433	-0.137664
C	-2.533398	0.002958	-0.001145
C	-3.457549	-1.056338	0.001554
C	-3.006242	1.326846	0.025504
C	-4.825750	-0.792754	0.031585
H	-3.099089	-2.079708	-0.018160
C	-4.374786	1.583130	0.056807
H	-2.303618	2.154063	0.017185
C	-5.289223	0.525253	0.060042
H	-5.530996	-1.618321	0.033935
H	-4.728506	2.609446	0.076720
H	-6.355723	0.727668	0.083807
C	2.551798	-0.042210	-0.006746
C	3.505502	-1.065867	0.131424
C	2.991344	1.286300	-0.115744
C	4.864960	-0.763871	0.158075
H	3.171588	-2.094053	0.221182
C	4.353776	1.585460	-0.086511
H	2.271866	2.090311	-0.232930
C	5.294699	0.562630	0.050233
H	5.590960	-1.564162	0.266808
H	4.678823	2.617929	-0.173130
H	6.354964	0.796235	0.073319

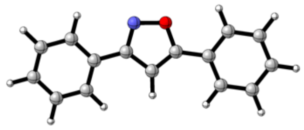

Int1

O	2.206622	-0.798263	-0.848560
N	1.105745	0.050906	-0.974826
C	1.522981	1.243558	-0.577597
C	2.891193	1.203114	-0.182410
H	3.500831	2.022925	0.161468
C	3.268668	-0.098255	-0.375455
Zn	-1.399509	-0.144976	1.061210
C	-2.201951	-1.219914	-0.505259
H	-1.808490	-2.227778	-0.653771
H	-2.197720	-0.710892	-1.471069
C	-0.634124	0.936416	2.614470
H	0.428327	0.703374	2.757161
H	-1.150837	0.741017	3.561473
H	-0.706871	2.008985	2.400980
I	-4.414971	-1.617134	-0.187522
C	4.523918	-0.820316	-0.186356
C	5.628112	-0.172781	0.395400
C	4.645022	-2.163653	-0.582807
C	6.828278	-0.856458	0.573439
H	5.545759	0.861991	0.712397
C	5.848546	-2.842014	-0.400399
H	3.797285	-2.667622	-1.033901
C	6.943072	-2.192356	0.176518
H	7.674490	-0.347149	1.024275
H	5.932102	-3.879019	-0.710984
H	7.879788	-2.723186	0.316862
C	0.601748	2.392415	-0.564921
C	0.867245	3.503008	0.250068
C	-0.572622	2.374168	-1.336844
C	-0.028323	4.571397	0.299371
H	1.761735	3.522897	0.864427
C	-1.467830	3.440846	-1.280974
H	-0.775522	1.520746	-1.974907
C	-1.199233	4.542328	-0.462242
H	0.185483	5.422775	0.938412
H	-2.372264	3.415588	-1.881397
H	-1.896769	5.373490	-0.420803

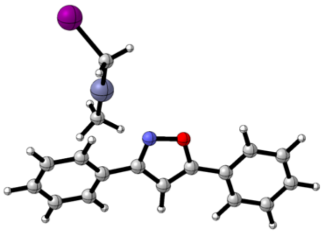

TS1

O	1.768295	-0.627321	-0.861135
N	0.725190	0.274943	-1.029196

C	1.182508	1.462352	-0.644075
C	2.537922	1.357240	-0.231661
H	3.181199	2.154008	0.104748
C	2.854376	0.031847	-0.383895
Zn	-1.566584	0.236561	1.174342
C	-1.164365	-0.725237	-0.607997
H	-0.595925	-1.649940	-0.669239
H	-1.538134	-0.429923	-1.583716
C	-1.607760	1.158686	3.000366
H	-1.071290	0.562034	3.747305
H	-2.634046	1.300939	3.357456
H	-1.129555	2.143947	2.948793
I	-3.467157	-2.289510	-0.284664
C	4.064310	-0.746843	-0.145884
C	5.220857	-0.113006	0.342194
C	4.086312	-2.131163	-0.391377
C	6.375898	-0.852927	0.580600
H	5.216180	0.954770	0.536014
C	5.246039	-2.864422	-0.150234
H	3.197222	-2.624872	-0.767991
C	6.392361	-2.229603	0.335808
H	7.263860	-0.355493	0.958009
H	5.254456	-3.932934	-0.341531
H	7.294308	-2.804110	0.523339
C	0.310321	2.644649	-0.651487
C	0.603097	3.739398	0.177791
C	-0.848972	2.673190	-1.446671
C	-0.251839	4.840138	0.214945
H	1.486560	3.721850	0.807848
C	-1.701336	3.774869	-1.403570
H	-1.067057	1.840844	-2.105944
C	-1.406459	4.859555	-0.572197
H	-0.019159	5.679659	0.862568
H	-2.591667	3.789669	-2.024543
H	-2.071349	5.717283	-0.540949

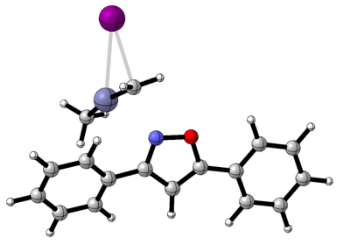

Int2

O	2.105836	-0.881022	-0.374929
N	0.966506	-0.081052	-0.467845
C	1.342044	1.199511	-0.416710
C	2.748191	1.245691	-0.272162
H	3.344808	2.141232	-0.215180
C	3.176210	-0.057335	-0.230793
Zn	-1.825124	-0.049811	0.823048
C	-0.296686	-0.771459	-0.503992
H	-0.090014	-1.795514	-0.188322
H	-0.668539	-0.797281	-1.531498
C	-2.380211	1.288961	2.282524
H	-2.171369	0.908894	3.290106
H	-3.453014	1.507649	2.225304
H	-1.836377	2.233763	2.160328
I	-3.921327	-1.901344	-0.258761
C	4.477630	-0.686513	-0.065372
C	5.617508	0.112800	0.135817
C	4.607437	-2.086785	-0.102726
C	6.865089	-0.482686	0.296504
H	5.528342	1.193649	0.169753
C	5.859581	-2.673272	0.061100
H	3.732083	-2.707421	-0.259577
C	6.989699	-1.875189	0.260290
H	7.740753	0.139203	0.452261
H	5.953373	-3.754129	0.032036
H	7.964358	-2.336092	0.387100
C	0.404264	2.323756	-0.470583
C	0.630264	3.426119	0.371898
C	-0.700349	2.325551	-1.338896
C	-0.254618	4.501471	0.364312

H	1.480564	3.424105	1.046518
C	-1.578690	3.408437	-1.343730
H	-0.863960	1.493747	-2.013686
C	-1.361548	4.492542	-0.490089
H	-0.083816	5.343209	1.027689
H	-2.429516	3.405272	-2.017261
H	-2.050771	5.331206	-0.492710

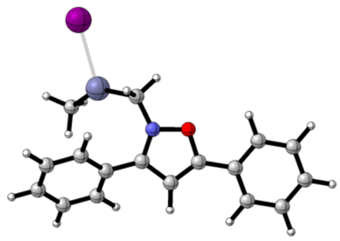

Int3

O	0.800452	1.457106	-0.190561
N	-0.589217	1.496060	-0.205681
C	-1.074338	0.212012	-0.103925
C	0.054873	-0.646844	-0.052408
H	0.008100	-1.718659	0.058509
C	1.182737	0.112022	-0.084393
C	-1.119156	2.711050	-0.348411
H	-0.434711	3.536163	-0.450878
H	-2.171851	2.776853	-0.558849
C	2.600818	-0.147974	0.005292
C	3.075793	-1.476069	0.064593
C	3.538611	0.904920	0.032129
C	4.438877	-1.735253	0.151519
H	2.372242	-2.302552	0.039503
C	4.902369	0.633118	0.119415
H	3.193401	1.932022	-0.013867
C	5.363031	-0.684204	0.180005
H	4.784075	-2.764076	0.196325
H	5.609078	1.457689	0.140065
H	6.426439	-0.891492	0.247070
C	-2.484514	-0.139260	-0.021141
C	-2.891470	-1.420413	-0.455886
C	-3.466615	0.715896	0.521810

C	-4.219142	-1.823211	-0.354651
H	-2.160059	-2.094418	-0.890784
C	-4.795335	0.304995	0.618071
H	-3.186769	1.689302	0.907287
C	-5.183797	-0.961729	0.177896
H	-4.504680	-2.811531	-0.703224
H	-5.529602	0.980893	1.046939
H	-6.221231	-1.273797	0.246665

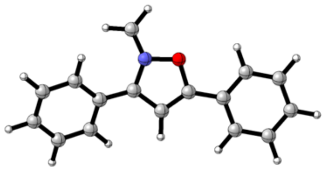

TS2

O	0.838326	1.524776	-0.344581
N	-0.644826	1.563809	-0.286352
C	-1.073014	0.275255	-0.301954
C	0.064168	-0.574593	-0.391029
H	0.036032	-1.650653	-0.331995
C	1.185851	0.200072	-0.310518
C	-1.201038	2.762375	-0.397243
H	-0.628345	3.614246	-0.065968
H	-2.105549	2.853741	-0.980946
C	2.582987	-0.119961	-0.066696
C	3.043944	-1.444266	-0.208044
C	3.502045	0.882624	0.298529
C	4.380608	-1.753469	0.022752
H	2.354817	-2.227751	-0.507536
C	4.837580	0.562402	0.532808
H	3.160628	1.906807	0.402427
C	5.285400	-0.754014	0.396778
H	4.719298	-2.778975	-0.091121
H	5.532102	1.346286	0.820238
H	6.327837	-0.999135	0.574797
C	-2.460555	-0.132433	-0.075261
C	-2.835448	-1.456342	-0.389340
C	-3.438082	0.717192	0.481694
C	-4.127931	-1.912114	-0.145378

H	-2.114066	-2.129731	-0.840255
C	-4.723862	0.247749	0.740156
H	-3.182728	1.741418	0.726394
C	-5.082132	-1.064993	0.424070
H	-4.390581	-2.933483	-0.404885
H	-5.453922	0.918912	1.183314
H	-6.091472	-1.419465	0.608705

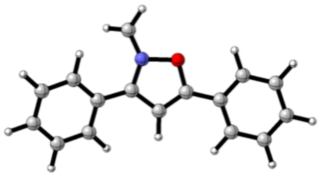

Int4

O	1.424870	2.118873	0.181124
N	-1.424045	2.113497	-0.077673
C	-1.196389	0.741071	0.007989
C	0.041120	0.178379	0.125493
H	0.069840	-0.886605	0.316124
C	1.326537	0.888539	0.124437
C	-1.214179	2.777963	-1.135982
H	-1.407466	3.850271	-1.135592
H	-0.834840	2.331127	-2.061319
C	2.578218	0.051252	0.089153
C	2.602313	-1.278313	-0.361571
C	3.781231	0.652198	0.493466
C	3.801613	-1.990493	-0.399688
H	1.693179	-1.756962	-0.709451
C	4.975735	-0.062277	0.468594
H	3.755067	1.684006	0.826976
C	4.988699	-1.387410	0.021296
H	3.808728	-3.014822	-0.759745
H	5.897152	0.410699	0.794980
H	5.920129	-1.945296	-0.002099
C	-2.435259	-0.074720	0.069166
C	-2.452839	-1.400660	-0.399436
C	-3.622786	0.477210	0.582432
C	-3.620197	-2.158338	-0.335857
H	-1.559776	-1.828937	-0.842300
C	-4.787042	-0.285834	0.648193
H	-3.620305	1.499953	0.942029
C	-4.790386	-1.605953	0.191758
H	-3.618961	-3.177736	-0.709473
H	-5.692442	0.151103	1.058373
H	-5.699287	-2.197944	0.239570

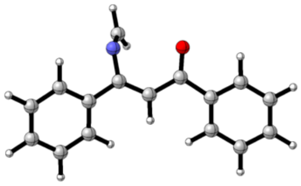

TS3

O	1.391134	2.056710	-0.305988
N	-1.272234	2.073329	0.017104
C	-1.214439	0.747537	-0.235775
C	0.004863	0.122398	-0.524840
H	0.028612	-0.957403	-0.522348
C	1.255172	0.794406	-0.343097
C	-0.417320	2.841897	-0.616027
H	-0.226525	3.839260	-0.226139
H	-0.233012	2.701153	-1.684412
C	2.484904	-0.015130	-0.076044
C	2.629677	-1.332522	-0.540462
C	3.541562	0.575646	0.636248
C	3.802245	-2.043842	-0.289022
H	1.842512	-1.798491	-1.123454
C	4.703626	-0.143858	0.903368
H	3.433010	1.598642	0.978715
C	4.838311	-1.455978	0.440454
H	3.906406	-3.057319	-0.664120
H	5.506938	0.319440	1.468177
H	5.747369	-2.014630	0.641808
C	-2.457316	-0.034170	-0.024355
C	-2.629453	-1.308304	-0.593009
C	-3.505663	0.512961	0.735888
C	-3.813392	-2.017298	-0.398910
H	-1.849425	-1.737965	-1.212686
C	-4.684270	-0.201741	0.936853

H	-3.377042	1.497769	1.170277
C	-4.842660	-1.469787	0.371224
H	-3.934182	-2.995893	-0.853418
H	-5.480364	0.230548	1.535593
H	-5.762818	-2.025380	0.525225

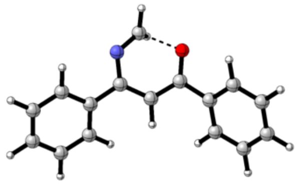

2a

O	1.190748	1.887819	0.214666
N	-1.222307	2.011735	-0.078885
C	-1.232652	0.722121	-0.225752
C	-0.001782	-0.027756	-0.448389
H	-0.013663	-1.076810	-0.702272
C	1.171991	0.570382	-0.105667
C	0.056864	2.622644	-0.324619
H	0.115946	3.601833	0.151964
H	0.219948	2.730157	-1.411089
C	2.482328	-0.089428	0.015839
C	2.585203	-1.480147	0.197721
C	3.658356	0.677653	-0.052456
C	3.834396	-2.088349	0.287313
H	1.687850	-2.082185	0.293163
C	4.906536	0.065282	0.043547
H	3.582713	1.750069	-0.190397
C	4.999033	-1.318456	0.209792
H	3.900139	-3.162399	0.431016
H	5.807464	0.668253	-0.015641
H	5.971920	-1.794678	0.284574
C	-2.530426	0.002939	-0.081999
C	-2.721934	-1.289777	-0.594927
C	-3.604970	0.635014	0.567473
C	-3.954382	-1.931724	-0.464319
H	-1.920570	-1.798354	-1.119662
C	-4.831004	-0.009554	0.705378
H	-3.457887	1.634211	0.961739

C	-5.011201	-1.297041	0.189674
H	-4.086452	-2.928217	-0.874929
H	-5.647747	0.490514	1.217547
H	-5.967642	-1.799907	0.297204

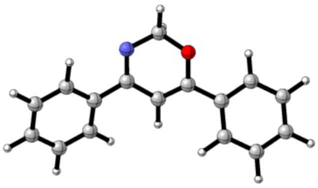

Int1-EtZnCH$_2$I

O	2.109997	-0.831317	-0.979517
N	1.096178	0.107247	-1.172216
C	1.578170	1.259018	-0.729271
C	2.908953	1.101388	-0.243378
H	3.558357	1.859439	0.162715
C	3.190425	-0.225404	-0.425242
Zn	-1.368860	-0.063939	0.885597
C	-2.148233	-1.158441	-0.684076
H	-1.605519	-2.068705	-0.947611
H	-2.333600	-0.599624	-1.603925
C	-0.670011	1.126366	2.404576
H	-0.776196	0.619861	3.373618
H	0.408356	1.269421	2.248891
I	-4.215007	-1.961936	-0.193015
C	4.363352	-1.052253	-0.154342
C	5.509226	-0.481403	0.427214
C	4.362983	-2.422061	-0.470924
C	6.630309	-1.266485	0.684677
H	5.522838	0.574587	0.677817
C	5.488026	-3.201776	-0.209744
H	3.482612	-2.867994	-0.920604
C	6.624133	-2.628370	0.367532
H	7.510012	-0.815372	1.133374
H	5.477468	-4.258666	-0.458157
H	7.499579	-3.238126	0.569246
C	0.755958	2.481253	-0.766258
C	1.185195	3.646775	-0.114111
C	-0.476517	2.493557	-1.442919

C	0.400236	4.799853	-0.137140
H	2.127213	3.654572	0.424287
C	-1.261355	3.644375	-1.457931
H	-0.812256	1.595863	-1.951050
C	-0.824990	4.802887	-0.807083
H	0.744271	5.693510	0.374545
H	-2.212089	3.639141	-1.982584
H	-1.436602	5.699919	-0.821571
C	-1.374630	2.493418	2.439685
H	-0.978661	3.157292	3.223599
H	-1.264680	3.022352	1.487572
H	-2.450555	2.386947	2.625758

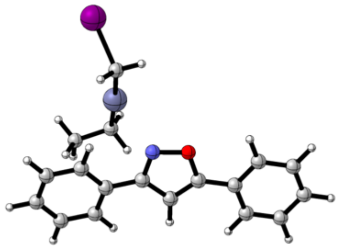

TS1-EtZnCH$_2$I

O	1.974238	-0.628685	-0.751572
N	0.870862	0.180925	-0.994307
C	1.229866	1.420048	-0.674520
C	2.578758	1.441240	-0.228326
H	3.155572	2.304340	0.061575
C	2.997026	0.137573	-0.295797
Zn	-1.686158	0.151514	0.938995
C	-0.919435	-0.979238	-0.610605
H	-0.241810	-1.818607	-0.479178
H	-1.213248	-0.871582	-1.651206
C	-2.253289	1.498218	2.372244
H	-2.664119	0.962719	3.238794
H	-1.353050	2.014443	2.733862
I	-3.023796	-2.813627	-0.277684
C	4.259727	-0.529859	-0.001092
C	5.318897	0.196892	0.571104
C	4.431063	-1.896818	-0.281899
C	6.526675	-0.434931	0.855100
H	5.195515	1.250934	0.798441
C	5.642577	-2.521637	0.005514
H	3.617880	-2.460673	-0.725615
C	6.692425	-1.794601	0.573453
H	7.338693	0.132931	1.298374
H	5.767507	-3.577059	-0.215516
H	7.635318	-2.284558	0.796238
C	0.290150	2.543467	-0.794614
C	0.469217	3.694858	-0.010954
C	-0.808878	2.468687	-1.668249
C	-0.435442	4.751832	-0.099674
H	1.303648	3.756351	0.680385
C	-1.711006	3.527746	-1.751825
H	-0.941150	1.587475	-2.286590
C	-1.526158	4.671673	-0.969574
H	-0.291675	5.635151	0.514634
H	-2.554218	3.463026	-2.432391
H	-2.229539	5.495906	-1.036662
C	-3.272212	2.529390	1.856220
H	-3.560599	3.260536	2.626289
H	-2.873930	3.095223	1.007253
H	-4.194570	2.046021	1.512381

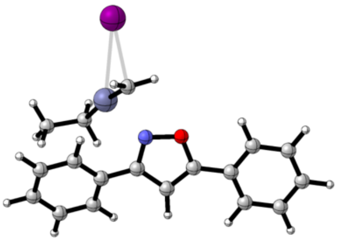

Int2-EtZnCH$_2$I

O	2.280569	-0.869629	-0.315208
N	1.124173	-0.091351	-0.434073
C	1.478719	1.196236	-0.416503
C	2.883024	1.270551	-0.266418
H	3.464194	2.176613	-0.219888
C	3.333195	-0.023633	-0.188196
Zn	-1.772089	-0.026932	0.631504
C	-0.121159	-0.807170	-0.495259

H	0.072223	-1.792468	-0.067034
H	-0.414496	-0.938292	-1.540887
C	-2.706517	1.308498	1.889582
H	-2.988486	0.794639	2.819143
H	-1.989677	2.090866	2.176915
I	-3.615795	-2.271469	-0.295962
C	4.644761	-0.623087	0.006470
C	5.790671	0.192560	0.009501
C	4.777007	-2.011014	0.193932
C	7.047089	-0.374833	0.200826
H	5.700381	1.262952	-0.143982
C	6.038080	-2.568895	0.386196
H	3.895998	-2.643386	0.193397
C	7.174217	-1.754602	0.390430
H	7.928121	0.258832	0.200373
H	6.133886	-3.639867	0.533596
H	8.155845	-2.193396	0.539897
C	0.525168	2.304199	-0.507148
C	0.754039	3.454883	0.267368
C	-0.600031	2.244648	-1.347634
C	-0.145163	4.517433	0.219157
H	1.618723	3.503170	0.921547
C	-1.491663	3.315417	-1.394637
H	-0.765596	1.376893	-1.974600
C	-1.270010	4.448292	-0.608337
H	0.029508	5.397230	0.829969
H	-2.356531	3.263886	-2.047831
H	-1.969790	5.277326	-0.642652
C	-3.946166	1.950779	1.246699
H	-4.458888	2.659605	1.915131
H	-3.678632	2.503210	0.338238
H	-4.682297	1.192943	0.953547

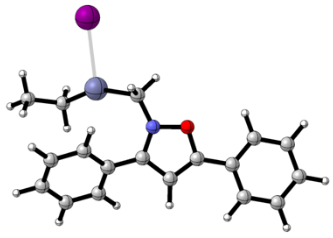

Int1-IZnCH$_2$I

O	1.718388	-0.802970	-0.222137
N	1.003169	0.359864	-0.498560
C	1.883405	1.351350	-0.560114
C	3.189065	0.857108	-0.311541
H	4.105364	1.424359	-0.320027
C	3.032052	-0.490456	-0.110349
Zn	-1.159603	0.041551	-0.300846
C	-1.801976	-1.483382	-1.523542
H	-0.998378	-2.144783	-1.853170
H	-2.374012	-1.173393	-2.399266
I	-3.212565	-2.887763	-0.480234
C	3.959720	-1.579840	0.170293
C	5.331552	-1.308169	0.316934
C	3.497634	-2.901751	0.296840
C	6.223378	-2.343193	0.584233
H	5.699192	-0.291445	0.224301
C	4.396566	-3.931384	0.565281
H	2.440619	-3.116694	0.184345
C	5.759325	-3.656328	0.709082
H	7.280859	-2.125473	0.695950
H	4.032798	-4.949481	0.662000
H	6.457256	-4.461273	0.917943
C	1.475122	2.728647	-0.866129
C	2.179072	3.804874	-0.303923
C	0.391186	2.982557	-1.721690
C	1.791291	5.114511	-0.582190
H	3.012655	3.615363	0.365172
C	0.007092	4.293016	-1.996932
H	-0.127814	2.151607	-2.187607
C	0.704683	5.360981	-1.425926
H	2.334696	5.942263	-0.137437
H	-0.830257	4.480412	-2.661733
H	0.405292	6.382098	-1.641475
I	-1.872991	1.833676	1.668941

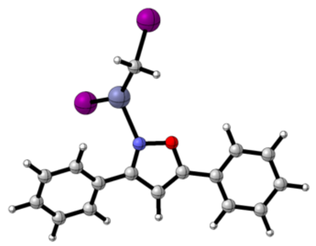

TS1-IZnCH$_2$I

O	2.560089	0.749082	0.682643
N	1.423465	0.145259	1.204642
C	1.620955	-1.167375	1.139718
C	2.900371	-1.442537	0.587914
H	3.351348	-2.410845	0.444284
C	3.445839	-0.213406	0.319788
Zn	-1.319461	0.173076	-0.416814
C	-0.298963	1.400283	0.839574
H	0.410261	2.147589	0.500234
H	-0.480461	1.439286	1.906852
I	-2.304745	3.333327	0.559556
C	4.722433	0.219227	-0.236338
C	5.668476	-0.738708	-0.642674
C	5.018908	1.586582	-0.375402
C	6.887688	-0.333182	-1.178601
H	5.450554	-1.796937	-0.541233
C	6.241214	1.984052	-0.912729
H	4.293384	2.329243	-0.062117
C	7.177732	1.027919	-1.315352
H	7.612060	-1.079191	-1.490088
H	6.462952	3.041547	-1.016850
H	8.129312	1.341106	-1.733822
C	0.590989	-2.116488	1.583537
C	0.547647	-3.409523	1.037271
C	-0.384353	-1.730458	2.519516
C	-0.459774	-4.296181	1.414488
H	1.286303	-3.713218	0.302269
C	-1.390705	-2.620154	2.890944
H	-0.339250	-0.742016	2.963298
C	-1.432227	-3.903250	2.338076
H	-0.489928	-5.290927	0.981293
H	-2.137621	-2.313955	3.616706
H	-2.217421	-4.594712	2.627729
I	-2.739217	-1.560487	-1.879295

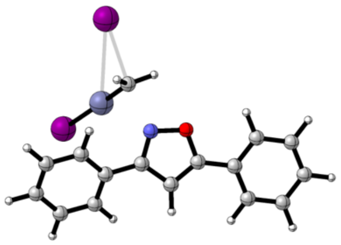

Int2-IZnCH$_2$I

O	2.608854	1.094795	0.507838
N	1.444546	0.428610	0.870528
C	1.708414	-0.876446	0.977792
C	3.073142	-1.074622	0.663942
H	3.594264	-2.017337	0.685082
C	3.586941	0.162947	0.368314
Zn	-1.450497	0.535494	-0.086337
C	0.255862	1.243378	0.941771
H	0.512646	2.189828	0.462670
H	0.014952	1.446333	1.988613
I	-3.560646	2.271141	0.484545
C	4.900535	0.640114	-0.033667
C	5.906921	-0.286979	-0.359155
C	5.173784	2.018540	-0.098601
C	7.167407	0.162286	-0.741547
H	5.701254	-1.351785	-0.323090
C	6.437541	2.457861	-0.483357
H	4.401699	2.736310	0.155994
C	7.435702	1.533393	-0.804504
H	7.939812	-0.556656	-0.994797
H	6.644266	3.522225	-0.530142
H	8.419901	1.880094	-1.103890
C	0.715086	-1.885042	1.350313
C	0.773027	-3.142964	0.725444
C	-0.265193	-1.637375	2.327318
C	-0.155625	-4.126477	1.052397

H	1.523575	-3.334277	-0.034559
C	-1.189622	-2.629769	2.650945
H	-0.292462	-0.685713	2.844494
C	-1.140276	-3.870492	2.011375
H	-0.117103	-5.089012	0.552897
H	-1.942891	-2.434573	3.407238
H	-1.865035	-4.638754	2.261936
I	-1.879062	-1.358315	-1.998345

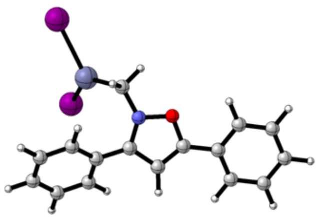

Int1-ICH$_2$ZnCH$_2$I

O	3.029997	0.372098	0.696720
N	1.813727	-0.212219	1.053124
C	1.932818	-1.498646	0.763147
C	3.213741	-1.784111	0.208943
H	3.599377	-2.744105	-0.094294
C	3.860415	-0.577735	0.195685
Zn	-1.037931	0.467796	-0.342957
C	-0.386997	2.157436	0.620848
H	0.576115	2.537708	0.277474
H	-0.353568	2.083783	1.708733
C	-1.754416	-1.197486	-1.309330
H	-1.761677	-1.169474	-2.400209
H	-1.318645	-2.141063	-0.980716
I	-1.766644	3.923205	0.289401
C	5.196885	-0.158980	-0.217535
C	6.046722	-1.066695	-0.873911
C	5.650773	1.146512	0.038991
C	7.325666	-0.674886	-1.261567
H	5.704614	-2.074851	-1.085504
C	6.931306	1.532054	-0.352902
H	5.000347	1.849598	0.547727
C	7.772471	0.624572	-1.002414
H	7.973698	-1.383237	-1.768509
H	7.273613	2.541866	-0.148222
H	8.770107	0.927409	-1.305602
C	0.814391	-2.423632	1.009007
C	0.696697	-3.602596	0.257699
C	-0.167474	-2.119342	1.967693
C	-0.393959	-4.452416	0.447312
H	1.442742	-3.842064	-0.493715
C	-1.257107	-2.968067	2.150139
H	-0.069042	-1.216020	2.560588
C	-1.375704	-4.134720	1.388862
H	-0.481101	-5.356483	-0.147533
H	-2.014689	-2.720589	2.887290
H	-2.228668	-4.791484	1.528743
I	-3.941823	-1.486105	-0.816328

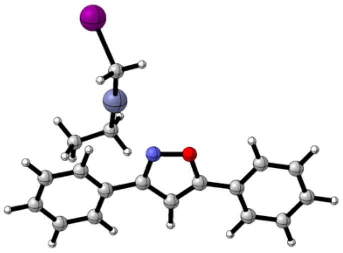

TS1-ICH$_2$ZnCH$_2$I

O	3.005374	0.357056	0.642857
N	1.773113	-0.108065	1.085879
C	1.775138	-1.423683	0.904209
C	3.013461	-1.843003	0.348307
H	3.312472	-2.851544	0.113101
C	3.745609	-0.692614	0.204399
Zn	-1.019861	0.431507	-0.459749
C	0.272623	1.451904	0.760746
H	1.111607	2.035559	0.394572
H	0.157022	1.542035	1.836148
C	-1.986473	-0.905086	-1.672509
H	-2.090630	-0.623320	-2.721087
H	-1.579173	-1.916596	-1.618444
I	-1.277462	3.764449	0.459122
C	5.085241	-0.407332	-0.296828
C	5.945976	-1.464985	-0.639975

C	5.525282	0.919489	-0.448906
C	7.221577	-1.197063	-1.129673
H	5.620604	-2.493168	-0.518540
C	6.803153	1.179089	-0.939081
H	4.864878	1.738688	-0.186410
C	7.653697	0.124164	-1.281266
H	7.880176	-2.019450	-1.390913
H	7.134595	2.206222	-1.055484
H	8.648895	0.329850	-1.663379
C	0.594862	-2.230867	1.241435
C	0.331856	-3.418180	0.539722
C	-0.307676	-1.797070	2.227980
C	-0.821886	-4.152248	0.812822
H	1.016659	-3.753897	-0.232746
C	-1.460328	-2.533881	2.495008
H	-0.096502	-0.889240	2.782984
C	-1.721425	-3.710394	1.786816
H	-1.021869	-5.064966	0.260389
H	-2.152351	-2.191872	3.258205
H	-2.621400	-4.280813	1.994105
I	-4.120048	-1.202226	-1.019184

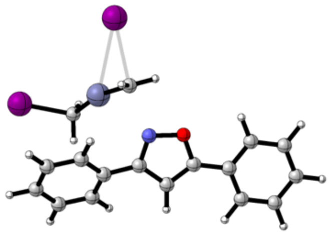

Int2-ICH$_2$IZnCH$_2$I

O	3.228055	0.769540	0.363959
N	1.966454	0.200676	0.578260
C	2.090473	-1.128769	0.527209
C	3.442231	-1.441590	0.258003
H	3.852203	-2.433340	0.162428
C	4.102394	-0.243677	0.146645
Zn	-0.998572	0.641219	-0.167876
C	0.877044	1.125971	0.755312
H	1.190730	2.056858	0.278780
H	0.737311	1.316867	1.823791
C	-1.896392	-0.838430	-1.305247
H	-2.050520	-0.605104	-2.360839
H	-1.405995	-1.811481	-1.234450
I	-2.427511	3.098471	0.263293
C	5.480039	0.115067	-0.153543
C	6.454531	-0.894850	-0.250124
C	5.846325	1.458609	-0.350950
C	7.773900	-0.561917	-0.541539
H	6.183334	-1.933375	-0.091008
C	7.168622	1.781344	-0.644445
H	5.097356	2.239705	-0.279661
C	8.133898	0.774811	-0.740185
H	8.522253	-1.344681	-0.612198
H	7.445852	2.819075	-0.799527
H	9.164000	1.030605	-0.968072
C	0.976182	-2.061771	0.700680
C	0.939018	-3.224604	-0.087650
C	-0.045005	-1.822359	1.635533
C	-0.121024	-4.119328	0.042358
H	1.721769	-3.410456	-0.815913
C	-1.099968	-2.723703	1.761256
H	-0.004147	-0.947360	2.273297
C	-1.143099	-3.868232	0.962508
H	-0.152893	-5.008325	-0.579084
H	-1.888863	-2.529367	2.479745
H	-1.971896	-4.562473	1.055722
I	-3.986601	-1.317985	-0.613315

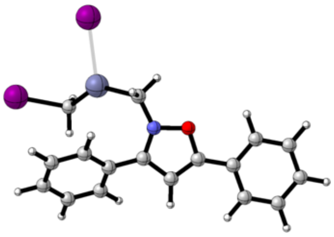

1w

O	0.551152	-1.008991	-0.000001
N	-0.845350	-0.873295	-0.000001
C	-1.062753	0.422247	0.000001
C	0.143942	1.176903	0.000000

H	0.264936	2.248495	-0.000009
C	1.108465	0.218133	0.000002
H	2.188430	0.248654	-0.000003
H	-2.083059	0.784141	0.000000

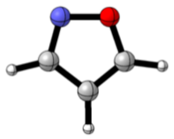

Int1-w

O	3.199517	-0.686307	-0.143973
N	2.675318	0.602591	-0.193558
C	3.696662	1.410125	-0.373873
C	4.919116	0.693325	-0.448811
H	5.914094	1.083685	-0.591264
C	4.534540	-0.604724	-0.297839
Zn	0.366281	1.067720	-0.048074
C	0.469298	3.112162	-0.175627
H	-0.512380	3.599715	-0.143457
H	0.950425	3.416766	-1.114956
H	1.067097	3.533430	0.643610
I	-2.641190	-0.898148	0.199197
H	3.515643	2.474458	-0.443332
H	5.069108	-1.543283	-0.277987
C	-0.378432	-0.869563	-0.017590
H	-0.238495	-1.450845	-0.932486
H	-0.070127	-1.498146	0.821547

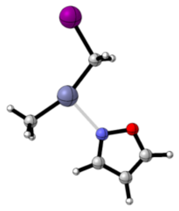

TS1-w

O	3.465854	-1.700689	-0.109590
N	2.715983	-0.548863	0.087117
C	3.550957	0.461322	0.011330
C	4.877396	0.015935	-0.231986
H	5.770106	0.610968	-0.340099
C	4.751150	-1.338313	-0.296420
Zn	0.107560	1.289584	0.149363
C	-0.075594	3.312649	0.010287
H	-0.842815	3.588686	-0.722374
H	0.867270	3.776016	-0.303813
H	-0.360057	3.752840	0.972996
I	-2.159449	-1.332116	0.450832
H	3.177695	1.469366	0.133152
H	5.449067	-2.146433	-0.460212
C	0.552019	-0.724131	0.249203
H	0.601303	-1.380156	-0.615475
H	0.712485	-1.270930	1.174005

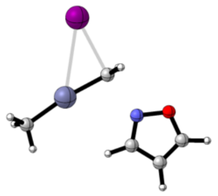

Int2-w

O	-3.510021	-1.634773	0.014765
N	-2.564563	-0.576530	-0.053268
C	-3.238928	0.557014	-0.035626
C	-4.621856	0.302962	0.042466
H	-5.419817	1.026573	0.077851
C	-4.716849	-1.059904	0.077617
Zn	0.111024	0.847106	0.013092
C	-1.170972	-0.895546	-0.141703
H	-0.991346	-1.398445	-1.096021
H	-0.930240	-1.580001	0.675600
C	-0.248537	2.875605	-0.013788
H	-0.750925	3.169432	-0.945177
H	0.679850	3.454603	0.051111
H	-0.890062	3.192420	0.818766
I	2.702669	-0.501898	0.011770
H	-2.698793	1.493484	-0.070123
H	-5.555878	-1.737570	0.152369

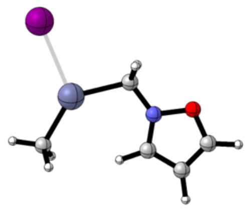

TS2-w

O	-3.627360	-1.729215	0.005532
N	-2.451949	-0.566892	0.014123
C	-3.170834	0.532680	-0.010382
C	-4.556213	0.325794	-0.033909
H	-5.317187	1.088581	-0.057828
C	-4.733636	-1.041076	-0.024979
Zn	0.185391	0.904289	0.017619
C	-1.145854	-0.947326	0.052938
H	-0.821456	-1.485102	-0.839145
H	-0.859352	-1.436532	0.985090
C	-0.355689	2.892836	-0.021344
H	-0.938308	3.136722	-0.917586
H	0.540111	3.523528	-0.023725
H	-0.952523	3.165459	0.857345
I	2.663371	-0.521014	0.022866
H	-2.642444	1.479489	-0.012569
H	-5.665761	-1.600378	-0.042594

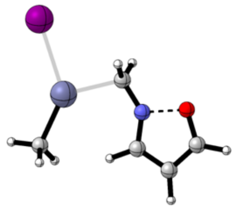

Int3-w

O	-1.208124	-1.304599	-0.171946
N	1.350871	0.138006	-0.460544
C	0.493252	1.142909	-0.069158
C	-0.843122	1.047561	0.144353
H	-1.387070	1.964395	0.349540
C	-1.633064	-0.179713	0.070186
C	1.848633	-0.733244	0.313862
H	2.510437	-1.492962	-0.100711
H	1.646509	-0.768232	1.389343
H	0.963865	2.125874	-0.042368
H	-2.719048	-0.023399	0.248117

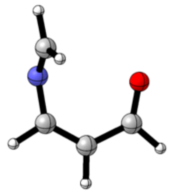

TS3-w

O	-1.042003	-1.123914	-0.129265
N	1.431323	-0.019350	-0.300473
C	0.796076	1.128926	-0.031808
C	-0.572341	1.203544	0.220698
H	-1.049246	2.174762	0.282024
C	-1.401245	0.082919	-0.061148
C	0.940606	-1.114478	0.246943
H	1.190686	-2.075829	-0.195372
H	0.675746	-1.124301	1.306496
H	1.366645	2.047285	-0.170891
H	-2.445640	0.299370	-0.332938

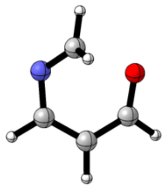

2w

O	0.180807	-1.322921	-0.226213
N	1.053222	0.968772	-0.142515
C	-0.171854	1.364293	-0.078271
C	-1.296148	0.476145	0.168934
H	-2.302281	0.851378	0.300895
C	-1.059296	-0.841462	-0.018124
C	1.235245	-0.420775	0.225549
H	2.146285	-0.820808	-0.220132
H	1.291721	-0.506591	1.323638
H	-0.375547	2.415050	-0.292779
H	-1.826876	-1.606271	-0.092845

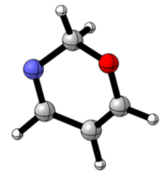

1z

O	0.687273	1.128863	0.000001
N	-0.721285	1.178520	-0.000002
C	-1.116934	-0.076849	-0.000003
C	-0.007875	-0.975215	-0.000001
H	-0.029309	-2.054130	-0.000002
C	1.086281	-0.164492	0.000002
C	2.552843	-0.416279	0.000000
H	3.022857	0.026762	-0.883874
H	3.022858	0.026755	0.883877
H	2.747764	-1.489914	-0.000004
C	-2.575434	-0.412793	0.000002
H	-2.833394	-1.006429	0.882689
H	-3.179868	0.496617	-0.000006
H	-2.833379	-1.006441	-0.882682

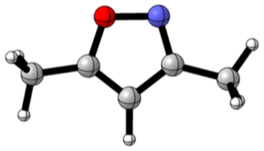

Int1-z

O	-3.203353	-0.185692	-1.138210
N	-2.375405	-1.327855	-1.128851
C	-2.415766	-1.780840	0.107424
C	-3.250172	-0.969625	0.933080
H	-3.474159	-1.102865	1.980368
C	-3.714223	0.004072	0.100062
Zn	-0.184645	1.066930	-0.041644
C	0.996259	-0.600519	-0.339076
H	1.021513	-0.989280	-1.358918
H	0.826797	-1.440568	0.336245
C	-1.299011	2.746885	0.275251
H	-1.925715	2.967404	-0.597055
H	-0.669965	3.625466	0.462502
H	-1.961741	2.623210	1.139652
I	3.183885	-0.125123	0.045173
C	-1.651828	-3.008320	0.493555
H	-2.336881	-3.805428	0.800169
H	-1.048766	-3.366208	-0.343258
H	-0.991139	-2.798990	1.340297
C	-4.631029	1.161228	0.282627
H	-4.111607	2.100744	0.073538
H	-5.485514	1.087675	-0.397644
H	-5.000080	1.185713	1.309387

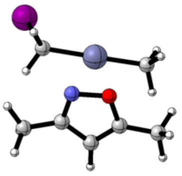

TS1-z

O	-2.566589	0.125824	-0.823225
N	-1.855395	-1.024480	-0.466364
C	-2.669220	-1.762687	0.262418
C	-3.938403	-1.127946	0.386146
H	-4.808255	-1.495534	0.908137
C	-3.815948	0.040794	-0.305359
Zn	0.088232	1.359045	0.212065
C	0.292103	-0.627765	-0.288781
H	0.407441	-0.995920	-1.303628
H	0.334514	-1.438110	0.434352
C	-0.330757	3.287715	0.723998
H	0.058603	3.989154	-0.023007
H	0.112400	3.550550	1.691500
H	-1.413434	3.446369	0.798015
I	3.055746	-0.439948	-0.114753
C	-2.221946	-3.069683	0.832442
H	-2.920595	-3.863510	0.553025
H	-1.226377	-3.333384	0.471099
H	-2.198730	-3.018277	1.925583
C	-4.735639	1.172514	-0.591732
H	-4.319800	2.111480	-0.212512

H	-4.886105	1.284060	-1.670187
H	-5.701853	0.996150	-0.116875

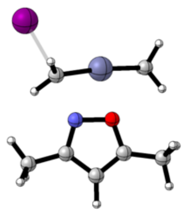

Int2-z

O	-2.689837	0.466688	-0.735335
N	-2.110043	-0.712412	-0.298066
C	-3.018372	-1.436631	0.342473
C	-4.241370	-0.720148	0.326754
H	-5.177178	-1.041049	0.755797
C	-3.996186	0.432477	-0.360297
Zn	0.433433	0.838201	0.262815
C	-0.687611	-0.819354	-0.495543
H	-0.495967	-0.938527	-1.565975
H	-0.369441	-1.722680	0.025126
C	0.400913	2.698566	1.154867
H	0.927256	3.438271	0.539427
H	0.894182	2.672646	2.134054
H	-0.623122	3.062115	1.307314
I	3.123048	-0.368961	-0.198294
C	-2.697824	-2.763942	0.938973
H	-3.619085	-3.275952	1.218403
H	-2.142116	-3.384722	0.230637
H	-2.081711	-2.642214	1.836239
C	-4.829973	1.585351	-0.778329
H	-4.415691	2.521824	-0.392992
H	-4.866157	1.655058	-1.870113
H	-5.843972	1.459609	-0.397031

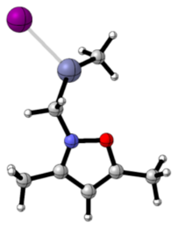

TS2-z

O	2.433772	-0.642424	-0.549272
N	1.983550	0.934770	-0.231299
C	3.139449	1.456978	0.161325
C	4.213901	0.562309	0.099010
H	5.245794	0.802341	0.301667
C	3.727598	-0.643966	-0.377733
Zn	-0.519765	-0.745083	0.315530
C	0.671152	1.177875	-0.029658
H	0.139038	1.587823	-0.886753
H	0.440164	1.612875	0.944569
C	0.136136	-2.566086	1.000423
H	0.506033	-3.164139	0.159338
H	-0.651321	-3.133632	1.507889
H	0.967482	-2.424323	1.700764
I	-3.118455	0.309100	-0.201488
C	3.176018	2.892896	0.582758
H	4.205462	3.250970	0.619856
H	2.592810	3.511649	-0.103476
H	2.731724	2.992507	1.579337
C	4.489155	-1.868539	-0.766817
H	4.082825	-2.744139	-0.252752
H	4.390427	-2.037972	-1.843813
H	5.545134	-1.756558	-0.516261

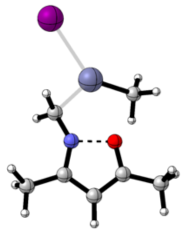

Int3-z

O	-1.360518	1.297806	-0.266965
N	1.455475	0.735610	-0.445542
C	0.981049	-0.524476	-0.089844
C	-0.331528	-0.834009	0.069698
H	-0.573213	-1.875953	0.256859
C	-1.468119	0.093477	-0.039038
C	1.636246	1.663215	0.399352

H	2.022110	2.623683	0.058064
H	1.416169	1.558587	1.467554
C	2.067600	-1.564345	-0.004212
H	1.658917	-2.557220	0.190902
H	2.641848	-1.588620	-0.936368
H	2.769570	-1.302860	0.796552
C	-2.838261	-0.543127	0.141044
H	-3.614111	0.219870	0.065221
H	-3.003978	-1.312726	-0.621689
H	-2.903419	-1.040896	1.115431

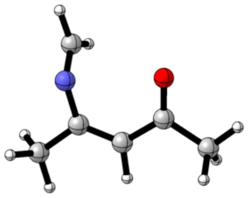

TS3-z

O	1.368718	1.070560	0.087970
N	-1.308497	0.974048	0.342772
C	-1.194633	-0.315006	-0.037902
C	0.043213	-0.863391	-0.377755
H	0.107420	-1.932319	-0.546231
C	1.264468	-0.179496	-0.083026
C	-0.455887	1.822560	-0.192676
H	-0.305137	2.784278	0.293019
H	-0.246044	1.791404	-1.265536
C	-2.425092	-1.171358	0.087232
H	-2.241793	-2.201519	-0.223769
H	-2.772142	-1.171714	1.126047
H	-3.235293	-0.752643	-0.519438
C	2.509463	-1.004568	0.158622
H	3.401787	-0.425182	-0.086120
H	2.559473	-1.279999	1.218426
H	2.492267	-1.927560	-0.426533

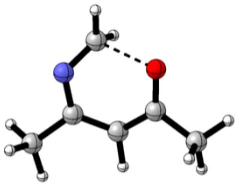

2z

O	-1.186017	0.972723	-0.269859
N	1.255151	1.074140	-0.089496
C	1.249047	-0.211878	0.042531
C	0.023429	-0.963014	0.293616
H	0.052595	-2.026246	0.496499
C	-1.154235	-0.348341	0.026575
C	-0.013996	1.702137	0.195716
H	-0.082954	2.671691	-0.299586
H	-0.125377	1.839235	1.285236
C	-2.495845	-0.996488	-0.064490
H	-3.188237	-0.543227	0.652735
H	-2.914887	-0.836223	-1.064178
H	-2.428278	-2.068815	0.125352
C	2.531056	-0.979461	-0.138926
H	2.741798	-1.574961	0.757102
H	2.447414	-1.682204	-0.975670
H	3.363273	-0.297747	-0.322278

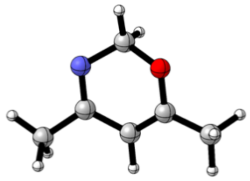

3a

C	1.123516	-0.355626	-0.067541
C	-1.201493	-0.443027	-0.092240
C	-0.035184	0.374247	0.052573
H	-0.051936	1.430871	0.283086
N	-0.976551	-1.733296	-0.314594
S	0.674402	-2.015127	-0.364825
C	2.514273	0.106378	0.018389
C	3.544000	-0.753634	0.439670
C	2.838369	1.431695	-0.324384
C	4.859365	-0.300172	0.517230
H	3.311254	-1.774830	0.727514
C	4.153393	1.883312	-0.238615
H	2.059350	2.100347	-0.676165

C	5.168891	1.019777	0.181032
H	5.641399	-0.976673	0.847885
H	4.387017	2.908658	-0.508556
H	6.193626	1.372658	0.243751
C	-2.594391	0.046802	0.006643
C	-3.647950	-0.854077	0.238037
C	-2.892204	1.412115	-0.133556
C	-4.961287	-0.399786	0.329998
H	-3.422510	-1.909002	0.349884
C	-4.208232	1.864805	-0.040141
H	-2.099261	2.125111	-0.334327
C	-5.247346	0.961831	0.193004
H	-5.763459	-1.108455	0.513441
H	-4.420920	2.923464	-0.154563
H	-6.271443	1.315122	0.266804

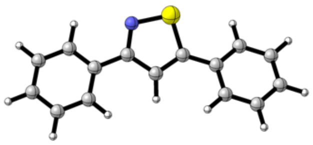

Int1-S

C	3.007585	-0.469682	-0.108506
C	1.458471	1.262372	-0.193720
C	2.835833	0.894136	-0.189340
H	3.650183	1.604364	-0.243575
N	0.599281	0.247964	-0.117094
S	1.434721	-1.213202	-0.060573
C	4.256473	-1.240765	-0.094105
C	4.333110	-2.487092	0.553351
C	5.403764	-0.733941	-0.731300
C	5.526494	-3.206630	0.563255
H	3.463673	-2.883689	1.070029
C	6.596228	-1.454349	-0.713761
H	5.352841	0.214446	-1.256461
C	6.661946	-2.692432	-0.068079
H	5.571013	-4.165153	1.071055
H	7.473061	-1.052578	-1.212303
H	7.591664	-3.253053	-0.058050
C	0.956621	2.648001	-0.283845
C	-0.236877	2.931706	-0.969037
C	1.671782	3.701227	0.308604
C	-0.710941	4.240212	-1.047123
H	-0.779600	2.123655	-1.450176
C	1.194645	5.009292	0.228393
H	2.588440	3.493090	0.851891
C	0.002607	5.282238	-0.447858
H	-1.632075	4.447656	-1.583326
H	1.751253	5.814386	0.698421
H	-0.366515	6.301484	-0.510509
Zn	-1.616931	0.211821	0.802522
C	-2.415769	-0.899636	-0.762223
H	-1.895477	-1.833116	-0.993433
H	-2.559412	-0.364590	-1.704700
C	-1.407934	1.193913	2.592751
H	-0.868208	0.574956	3.321309
H	-2.366714	1.472584	3.046516
H	-0.826680	2.113828	2.451184
I	-4.520646	-1.644507	-0.332254

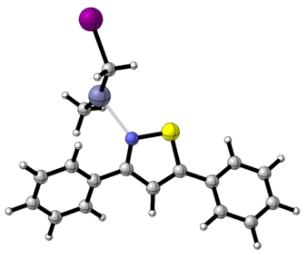

TS1-S

C	3.042247	-0.262143	-0.348632
C	1.124115	1.029829	-0.607684
C	2.515943	1.009811	-0.304310
H	3.090611	1.895110	-0.066586
N	0.592706	-0.161827	-0.883322
S	1.766169	-1.364412	-0.786403
C	4.427974	-0.688374	-0.124536
C	4.725897	-1.978812	0.349159
C	5.485899	0.203151	-0.381758
C	6.047604	-2.365785	0.561720

H	3.921456	-2.673552	0.573672
C	6.805163	-0.186555	-0.162214
H	5.273096	1.192818	-0.772893
C	7.090865	-1.471243	0.309042
H	6.261769	-3.363519	0.932028
H	7.611605	0.510645	-0.367820
H	8.120090	-1.773393	0.476704
C	0.280003	2.240112	-0.607139
C	-0.778602	2.377360	-1.519730
C	0.509654	3.256573	0.335070
C	-1.599545	3.503344	-1.481034
H	-0.939251	1.608862	-2.267558
C	-0.313782	4.381351	0.370971
H	1.314028	3.152492	1.056472
C	-1.372034	4.505662	-0.533519
H	-2.412537	3.600663	-2.193922
H	-0.133726	5.156192	1.109744
H	-2.013673	5.381027	-0.503037
Zn	-1.590543	0.072494	1.348488
C	-1.472674	-0.799473	-0.517994
H	-1.164861	-1.827939	-0.696202
H	-1.770329	-0.315656	-1.441487
C	-1.331272	1.158090	3.051139
H	-0.461067	0.805774	3.617834
H	-2.203364	1.112241	3.713221
H	-1.157173	2.208305	2.789491
I	-4.134958	-1.723753	-0.339243

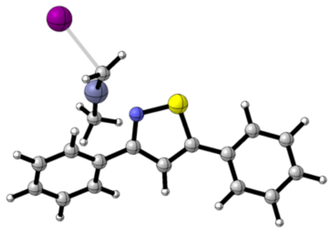

Int2-S

C	3.251111	-0.273598	-0.217514
C	1.228176	0.882527	-0.423909
C	2.634259	0.956246	-0.273403
H	3.158488	1.897789	-0.186155
N	0.774755	-0.382633	-0.488421
S	2.058949	-1.520285	-0.415259
C	4.674066	-0.579444	-0.052914
C	5.098968	-1.786702	0.531733
C	5.639480	0.346879	-0.489494
C	6.457330	-2.058786	0.676350
H	4.368600	-2.503095	0.897668
C	6.995882	0.070522	-0.337783
H	5.326393	1.269230	-0.967597
C	7.409268	-1.131739	0.243680
H	6.771708	-2.991500	1.133773
H	7.731242	0.790526	-0.682867
H	8.467458	-1.345545	0.357359
C	0.333975	2.050071	-0.443299
C	-0.721032	2.162089	-1.364323
C	0.550528	3.080683	0.488851
C	-1.557167	3.277739	-1.336789
H	-0.875300	1.393375	-2.111470
C	-0.292578	4.189310	0.514962
H	1.360063	2.996824	1.207037
C	-1.350044	4.287789	-0.394302
H	-2.368155	3.357143	-2.053573
H	-0.127518	4.972988	1.247456
H	-2.007465	5.151350	-0.370863
Zn	-1.973585	-0.115111	0.945218
C	-0.605361	-0.873080	-0.522855
H	-0.565231	-1.954728	-0.374463
H	-1.022629	-0.695800	-1.515335
C	-2.277264	1.037318	2.619776
H	-3.309834	0.967233	2.981141
H	-2.069844	2.090493	2.392152
H	-1.614002	0.740667	3.442112
I	-4.286802	-1.500143	-0.358537

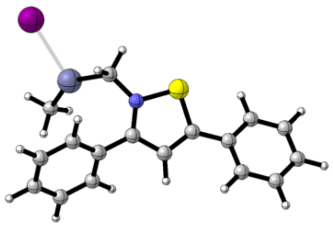

Int3-S

C	-1.224225	0.240196	0.087815
C	1.145589	0.326384	0.095011
C	-0.036272	-0.444658	0.095756
H	0.014450	-1.525092	0.050820
N	0.882725	1.688801	0.123059
S	-0.857189	1.983146	0.120301
C	-2.591895	-0.237088	-0.008294
C	-3.692923	0.640303	0.097969
C	-2.866609	-1.609560	-0.209792
C	-5.001473	0.171696	0.000776
H	-3.530560	1.701960	0.269789
C	-4.174915	-2.071993	-0.295405
H	-2.048307	-2.315300	-0.308455
C	-5.254884	-1.187316	-0.193934
H	-5.825905	0.873852	0.084914
H	-4.354182	-3.132149	-0.450510
H	-6.274374	-1.552923	-0.265517
C	2.504372	-0.224152	0.036359
C	3.524856	0.337142	-0.757831
C	2.794063	-1.408749	0.744311
C	4.780924	-0.261535	-0.834573
H	3.326871	1.231703	-1.338557
C	4.049437	-2.006939	0.659250
H	2.029209	-1.851299	1.375182
C	5.053350	-1.434707	-0.126190
H	5.547920	0.187530	-1.459004
H	4.247108	-2.916832	1.218539
H	6.034785	-1.895225	-0.183953
C	1.709911	2.732432	0.211258
H	1.292152	3.725961	0.247652
H	2.760936	2.547131	0.357958

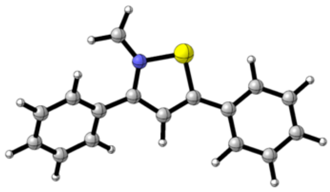

TS2-S

C	1.232853	0.348923	-0.295780
C	-1.144792	0.425668	-0.235050
C	0.036669	-0.321118	-0.433819
H	-0.009701	-1.399795	-0.514778
N	-0.923787	1.760861	-0.000499
S	0.935643	2.070668	-0.142824
C	2.558677	-0.224270	-0.074333
C	3.722870	0.558002	-0.217490
C	2.715820	-1.581122	0.283119
C	4.986952	0.015389	0.007934
H	3.639339	1.595664	-0.529950
C	3.980860	-2.125143	0.481355
H	1.840610	-2.205157	0.430460
C	5.125169	-1.330404	0.351755
H	5.866103	0.643112	-0.103852
H	4.074343	-3.171478	0.757362
H	6.110016	-1.756885	0.515223
C	-2.475656	-0.192004	-0.098355
C	-3.439441	0.254156	0.827485
C	-2.781983	-1.323313	-0.880343
C	-4.649699	-0.418229	0.974624
H	-3.233404	1.124749	1.440348
C	-3.998963	-1.987809	-0.734739
H	-2.067070	-1.672489	-1.618816
C	-4.940709	-1.541045	0.193667
H	-5.371068	-0.061646	1.704204
H	-4.212399	-2.852699	-1.356052
H	-5.891326	-2.053959	0.302620
C	-1.773068	2.770779	0.053081
H	-1.434239	3.717987	0.446463
H	-2.748335	2.665138	-0.403221

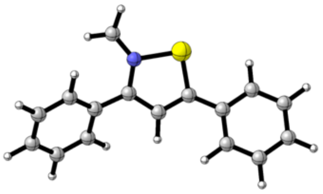

Int4-S

C	1.311695	0.791723	-0.052125
C	-1.221371	0.667536	0.065552
C	0.038063	0.135292	-0.112682
H	0.068896	-0.936101	-0.271419
N	-1.433730	1.976338	0.457880
S	1.541824	2.458434	-0.038316
C	2.492416	-0.117593	0.021754
C	3.690852	0.198807	-0.643169
C	2.436766	-1.308580	0.770858
C	4.791421	-0.651019	-0.571208
H	3.741772	1.111163	-1.227390
C	3.546709	-2.147769	0.859377
H	1.533381	-1.560292	1.316918
C	4.725453	-1.825978	0.183448
H	5.701984	-0.397677	-1.105751
H	3.490600	-3.051140	1.459142
H	5.586607	-2.484588	0.244404
C	-2.421226	-0.203301	-0.013926
C	-3.552831	0.059287	0.781017
C	-2.454104	-1.302328	-0.893330
C	-4.674634	-0.763250	0.710168
H	-3.542844	0.897338	1.468786
C	-3.583063	-2.114304	-0.969177
H	-1.609948	-1.498716	-1.545364
C	-4.695052	-1.852027	-0.164775
H	-5.532988	-0.554475	1.341070
H	-3.597715	-2.947143	-1.665298
H	-5.572726	-2.488206	-0.223773
C	-2.113341	2.819532	-0.205483
H	-2.304241	3.799676	0.228869
H	-2.502371	2.624701	-1.210097

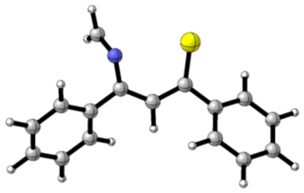

TS3-S

C	-1.267234	0.766939	0.121585

C	1.285199	0.737277	0.039188
C	0.023865	0.196172	0.306170
H	0.016262	-0.873119	0.480133
N	1.465740	2.003963	-0.413794
S	-1.668914	2.414013	-0.036909
C	-2.384082	-0.213732	0.001813
C	-3.629743	0.036150	0.606637
C	-2.220406	-1.406472	-0.729051
C	-4.673450	-0.879056	0.491636
H	-3.763057	0.944154	1.184685
C	-3.272282	-2.311352	-0.859180
H	-1.277409	-1.605664	-1.227481
C	-4.500207	-2.053924	-0.244787
H	-5.621911	-0.676757	0.979818
H	-3.133862	-3.215394	-1.444297
H	-5.316271	-2.763985	-0.338866
C	2.491888	-0.120899	0.059836
C	3.602074	0.211987	-0.738596
C	2.571565	-1.253383	0.891291
C	4.747727	-0.579175	-0.722626
H	3.547157	1.085992	-1.377453
C	3.723763	-2.035690	0.912307
H	1.746077	-1.506110	1.548136
C	4.813144	-1.705958	0.101551
H	5.590808	-0.317020	-1.354437
H	3.773767	-2.899652	1.567678
H	5.708575	-2.319777	0.116376
C	0.943165	3.025584	0.171620
H	0.957931	3.988936	-0.333018
H	0.684464	3.019622	1.229471

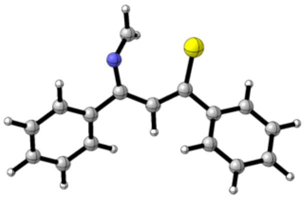

4a

C	-1.159242	0.547906	0.097440

C	1.309835	0.728056	0.297207
C	0.036153	0.029967	0.488356
H	0.052329	-1.006007	0.804195
N	1.429255	2.012132	0.180722
S	-1.270282	2.235181	-0.428143
C	-2.392294	-0.262050	0.000218
C	-3.651082	0.284453	0.309126
C	-2.323329	-1.607506	-0.408669
C	-4.803028	-0.496654	0.227687
H	-3.720344	1.316629	0.636158
C	-3.476076	-2.385003	-0.487758
H	-1.365251	-2.031318	-0.691245
C	-4.720004	-1.832977	-0.168852
H	-5.765275	-0.061497	0.479561
H	-3.405560	-3.418780	-0.811951
H	-5.618308	-2.439195	-0.234920
C	2.546547	-0.093482	0.136259
C	3.606335	0.399290	-0.644384
C	2.694579	-1.343184	0.759016
C	4.775295	-0.339927	-0.804153
H	3.493070	1.364749	-1.124498
C	3.871569	-2.077926	0.607605
H	1.906279	-1.741342	1.389060
C	4.913272	-1.582618	-0.177936
H	5.579670	0.051433	-1.419980
H	3.972560	-3.037341	1.105962
H	5.825256	-2.158857	-0.301937
C	0.258009	2.809482	0.452675
H	0.413590	3.838484	0.123906
H	0.032461	2.808271	1.527902

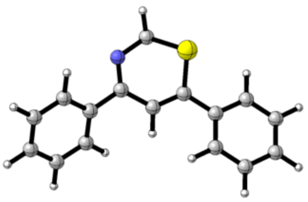

3b

C	1.679026	0.843865	0.049754
C	-0.327670	1.838258	-0.111866
C	1.039558	2.057602	0.210230
H	1.520846	2.980378	0.495764
N	-0.533562	0.564669	-0.451703
N	0.681230	-0.031509	-0.346305
C	3.106678	0.522347	0.177186
C	3.776018	-0.244999	-0.790131
C	3.833075	1.052137	1.255181
C	5.142342	-0.488702	-0.668881
H	3.229774	-0.637218	-1.641679
C	5.201219	0.809344	1.369662
H	3.316811	1.640664	2.007164
C	5.858726	0.035999	0.410136
H	5.649634	-1.083194	-1.422433
H	5.751494	1.218879	2.211220
H	6.923572	-0.155368	0.501189
C	-1.444200	2.798333	-0.102312
C	-2.761854	2.358898	-0.320242
C	-1.215855	4.164158	0.126816
C	-3.819650	3.264561	-0.309005
H	-2.944664	1.303695	-0.494085
C	-2.277836	5.068468	0.139020
H	-0.206051	4.526395	0.291425
C	-3.583197	4.623385	-0.078987
H	-4.832099	2.909679	-0.477884
H	-2.083429	6.121919	0.316906
H	-4.409288	5.328015	-0.069729
S	0.742792	-1.767542	-0.600552
O	0.940011	-1.984086	-2.036351
O	1.716395	-2.273857	0.366979
C	-0.895840	-2.263619	-0.136569
C	-1.154599	-2.543405	1.206081
C	-1.885422	-2.363729	-1.116204
C	-2.441431	-2.931845	1.567696
H	-0.367065	-2.460312	1.946471
C	-3.163623	-2.753945	-0.731124
H	-1.655297	-2.137556	-2.150609
C	-3.461640	-3.041931	0.610687

H	-2.657243	-3.152433	2.608655
H	-3.944140	-2.833988	-1.481830
C	-4.845306	-3.489745	1.007542
H	-4.951978	-4.571858	0.865275
H	-5.050235	-3.274117	2.059312
H	-5.611358	-3.004100	0.396691

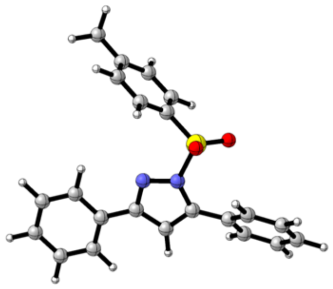

Int1-N

C	-1.710816	-0.210336	1.592550
C	-0.422399	1.611476	1.347339
C	-1.500897	1.054236	2.090171
H	-2.047655	1.511372	2.900716
N	0.010850	0.742948	0.433938
N	-0.734097	-0.397541	0.616612
C	-2.723878	-1.200318	1.985092
C	-2.378992	-2.522103	2.307748
C	-4.063623	-0.790122	2.082798
C	-3.364321	-3.420613	2.711265
H	-1.344612	-2.837169	2.236263
C	-5.046046	-1.694490	2.485229
H	-4.328419	0.230385	1.824300
C	-4.698240	-3.010952	2.798387
H	-3.090445	-4.441388	2.959416
H	-6.080900	-1.372508	2.548851
H	-5.463287	-3.715757	3.109940
C	0.194209	2.935174	1.519490
C	0.861497	3.566546	0.456227
C	0.113518	3.588865	2.760028
C	1.443283	4.820092	0.635933
H	0.911562	3.080303	-0.511114
C	0.693635	4.844953	2.934524
H	-0.386375	3.106821	3.594250
C	1.361395	5.463392	1.874545
H	1.953320	5.298561	-0.194732
H	0.628869	5.337247	3.900081
H	1.813541	6.440996	2.011942
Zn	2.041267	0.543642	-0.866719
C	2.956866	-0.510694	0.666520
H	2.291035	-1.199564	1.192773
H	3.514611	0.055306	1.416805
C	1.865913	1.595183	-2.623934
H	1.035844	2.314077	-2.616252
H	1.689219	0.910006	-3.463460
H	2.773060	2.165956	-2.860651
I	4.555523	-1.928686	-0.115801
S	-0.889638	-1.391668	-0.822051
O	0.416877	-1.261846	-1.476079
O	-1.385180	-2.690511	-0.387043
C	-2.127341	-0.528281	-1.744799
C	-3.467201	-0.890496	-1.579767
C	-1.738724	0.548644	-2.546109
C	-4.436954	-0.152210	-2.252068
H	-3.738127	-1.724116	-0.942060
C	-2.729219	1.269010	-3.206745
H	-0.691799	0.809721	-2.648097
C	-4.086212	0.932324	-3.072277
H	-5.482677	-0.421107	-2.137040
H	-2.444707	2.107074	-3.836065
C	-5.144371	1.704683	-3.818194
H	-6.105025	1.673402	-3.297318
H	-4.854359	2.750233	-3.953515
H	-5.296514	1.275156	-4.815778

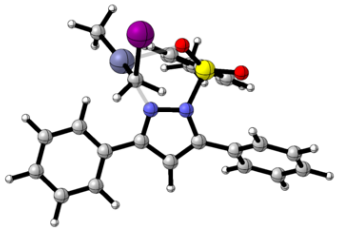

TS1-N

C	-1.908928	-1.209383	1.080290
C	-0.286787	0.159918	1.804156
C	-1.472662	-0.519083	2.189072
H	-1.938533	-0.517695	3.162860
N	0.002332	-0.101098	0.527345
N	-0.960212	-0.981882	0.076227
C	-3.094569	-2.058960	0.940916
C	-3.037263	-3.297877	0.281949
C	-4.300536	-1.641928	1.530678
C	-4.172425	-4.101877	0.211326
H	-2.105629	-3.621711	-0.166924
C	-5.433789	-2.449363	1.452754
H	-4.346944	-0.678020	2.027896
C	-5.371611	-3.680068	0.793183
H	-4.120781	-5.059638	-0.296907
H	-6.364219	-2.116297	1.901970
H	-6.254825	-4.308725	0.732817
C	0.563107	1.041801	2.609242
C	1.238069	2.113942	1.999721
C	0.701980	0.832418	3.990308
C	2.051649	2.951271	2.758947
H	1.094938	2.296436	0.939863
C	1.517504	1.673723	4.746039
H	0.190099	0.000527	4.464163
C	2.195634	2.730539	4.132475
H	2.566500	3.779926	2.282690
H	1.628603	1.500801	5.811919
H	2.831031	3.383348	4.723182
Zn	2.095028	0.814144	-1.513720
C	1.998742	-0.574002	-0.014385
H	1.689681	-1.603457	-0.179382
H	2.306561	-0.406692	1.014301
C	2.026536	2.303336	-2.902856
H	1.651941	3.245654	-2.484172
H	1.363378	2.018991	-3.728500
H	3.019123	2.503123	-3.323314
I	4.702974	-1.528288	-0.399281
S	-1.393932	-0.652610	-1.613768
O	-0.113257	-0.465244	-2.297440
O	-2.308885	-1.714636	-2.011559
C	-2.254341	0.891865	-1.475922
C	-3.612458	0.869411	-1.142299
C	-1.548742	2.087977	-1.625947
C	-4.269448	2.082028	-0.958103
H	-4.137906	-0.072008	-1.028173
C	-2.231039	3.287088	-1.439297
H	-0.499446	2.084767	-1.894760
C	-3.593295	3.304766	-1.101498
H	-5.323798	2.079655	-0.698258
H	-1.695519	4.224281	-1.557829
C	-4.325145	4.610817	-0.925749
H	-5.134217	4.519795	-0.195899
H	-3.650245	5.407369	-0.601381
H	-4.774135	4.926016	-1.875390

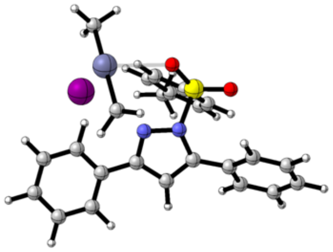

Int2-N

C	2.229323	0.152335	-1.387868
C	0.549176	1.614487	-1.054163
C	1.933886	1.488195	-1.326575
H	2.615782	2.303728	-1.509574
N	0.024613	0.378450	-0.955727
N	1.040562	-0.567110	-1.140991
C	3.509316	-0.486778	-1.687336
C	3.575361	-1.647783	-2.476744
C	4.697345	0.107629	-1.225703
C	4.811310	-2.206949	-2.789803
H	2.659402	-2.100838	-2.837664
C	5.930197	-0.457581	-1.542502
H	4.645454	0.990809	-0.596987
C	5.989411	-1.616053	-2.323136

H	4.855715	-3.103293	-3.400280
H	6.842908	-0.000356	-1.173635
H	6.950949	-2.057563	-2.566682
C	-0.222048	2.857112	-0.994784
C	-1.283459	3.033830	-0.088600
C	0.150991	3.924356	-1.833512
C	-1.957929	4.251205	-0.029891
H	-1.566519	2.233704	0.584331
C	-0.534284	5.135086	-1.776177
H	0.961935	3.795182	-2.542946
C	-1.590001	5.301305	-0.875279
H	-2.770601	4.379084	0.677986
H	-0.246774	5.947066	-2.436467
H	-2.123011	6.246016	-0.831460
Zn	-2.374275	-0.553130	0.849802
C	-1.375266	-0.032500	-0.962607
H	-1.435207	-0.912332	-1.602979
H	-1.929032	0.783265	-1.431015
C	-2.346340	-0.587110	2.906230
H	-2.022811	0.368533	3.338667
H	-1.662338	-1.363470	3.269694
H	-3.344908	-0.804906	3.302992
I	-4.950524	-1.274945	-0.537109
S	1.169855	-1.816750	0.213759
O	-0.205561	-2.166170	0.551313
O	2.094695	-2.811941	-0.306059
C	1.920612	-0.864441	1.502179
C	3.302247	-0.964739	1.692536
C	1.130288	0.024602	2.236775
C	3.895416	-0.151381	2.653157
H	3.890510	-1.656405	1.102132
C	1.751061	0.829337	3.186289
H	0.059488	0.075830	2.085967
C	3.136015	0.756603	3.408511
H	4.966706	-0.220326	2.815453
H	1.149462	1.523443	3.765186
C	3.789260	1.615681	4.460141
H	4.835537	1.819208	4.217350
H	3.265653	2.567975	4.579446
H	3.769436	1.106463	5.431201

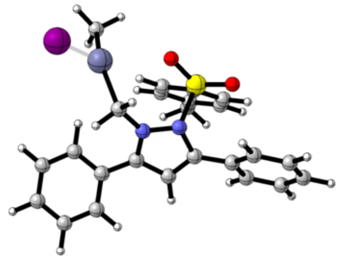

Int3-N

C	1.328667	-1.006941	-0.060766
C	-0.918542	-1.325043	0.223315
C	0.154515	-1.329065	-0.693088
H	0.057074	-1.595789	-1.735268
N	-0.361330	-1.089319	1.474430
N	1.020255	-0.732405	1.317186
C	2.694560	-0.973817	-0.557852
C	3.798863	-1.156298	0.298460
C	2.939304	-0.787146	-1.933599
C	5.095384	-1.159950	-0.207058
H	3.627340	-1.291418	1.360463
C	4.239138	-0.802956	-2.434493
H	2.104600	-0.613273	-2.606261
C	5.326311	-0.987911	-1.575922
H	5.932059	-1.302716	0.470958
H	4.403857	-0.656276	-3.498182
H	6.339359	-0.991729	-1.966350
C	-2.330548	-1.569418	-0.021742
C	-3.348020	-0.946095	0.731107
C	-2.718560	-2.399776	-1.095938
C	-4.692184	-1.154305	0.427407
H	-3.087936	-0.262306	1.531218
C	-4.062314	-2.597735	-1.398244
H	-1.956978	-2.900851	-1.685290
C	-5.060067	-1.981953	-0.635500
H	-5.454805	-0.657491	1.020222
H	-4.333177	-3.245449	-2.227033
H	-6.107871	-2.145673	-0.867241

C	-0.823441	-1.220189	2.713857
H	-0.125409	-1.113486	3.524739
H	-1.870082	-1.427673	2.854407
S	1.149020	1.082310	1.691637
O	0.607344	1.249620	3.043205
O	2.548241	1.386251	1.392556
C	0.072889	1.842666	0.512019
C	0.526206	2.019125	-0.797830
C	-1.252975	2.108780	0.876367
C	-0.376342	2.476712	-1.756652
H	1.556652	1.804819	-1.056445
C	-2.131518	2.564020	-0.097601
H	-1.575914	1.960108	1.899938
C	-1.711535	2.745432	-1.427459
H	-0.037053	2.619808	-2.778057
H	-3.161829	2.776291	0.171696
C	-2.695025	3.210020	-2.471153
H	-2.207469	3.400606	-3.429979
H	-3.472217	2.453760	-2.629736
H	-3.200275	4.127933	-2.153190

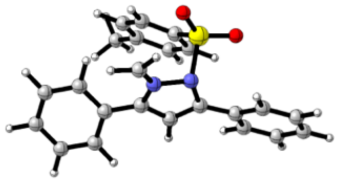

TS2-N

C	1.407236	0.717209	-0.064352
C	-0.833880	1.130406	-0.437020
C	0.238187	1.208481	0.477763
H	0.165769	1.782985	1.389950
N	-0.466666	0.536398	-1.595906
N	1.142726	0.085539	-1.286668
C	2.779836	1.005218	0.355605
C	3.865213	0.797589	-0.515996
C	3.043126	1.482372	1.654715
C	5.165738	1.082263	-0.108151
H	3.676249	0.410273	-1.509930
C	4.347181	1.753274	2.060704
H	2.227097	1.627226	2.355756
C	5.416385	1.560253	1.181045
H	5.988747	0.923152	-0.798787
H	4.529284	2.114786	3.068535
H	6.432424	1.772463	1.499436
C	-2.128021	1.810733	-0.275236
C	-3.330780	1.191850	-0.654224
C	-2.178887	3.092170	0.305111
C	-4.550981	1.844389	-0.474661
H	-3.319694	0.179540	-1.040917
C	-3.398353	3.739694	0.484207
H	-1.256459	3.586858	0.592355
C	-4.588656	3.120002	0.090065
H	-5.472067	1.350134	-0.768062
H	-3.420398	4.732707	0.922646
H	-5.538380	3.627873	0.227307
C	-1.055536	0.152663	-2.701767
H	-0.460217	0.108681	-3.601307
H	-2.052781	-0.262771	-2.681130
S	1.132474	-1.649036	-1.256013
O	0.727425	-2.081325	-2.596305
O	2.453980	-2.006235	-0.729862
C	-0.116258	-2.125976	-0.083896
C	0.168168	-2.046166	1.282024
C	-1.392101	-2.469056	-0.543322
C	-0.850460	-2.305752	2.195959
H	1.166954	-1.793210	1.617729
C	-2.394467	-2.722192	0.387792
H	-1.582633	-2.539871	-1.606989
C	-2.144473	-2.634172	1.766875
H	-0.636888	-2.249649	3.259192
H	-3.386570	-2.995471	0.040213
C	-3.254274	-2.866941	2.760853
H	-3.850167	-1.955024	2.887733
H	-3.933917	-3.654237	2.422311
H	-2.863276	-3.144832	3.742954

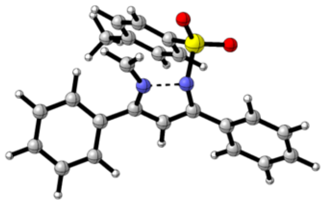

Int4-N

C	-0.510808	1.239496	-0.311098
C	-1.885933	-0.884665	-0.232439
C	-1.746070	0.480240	-0.239289
H	-2.651369	1.066979	-0.151486
N	-0.823295	-1.772379	-0.323701
N	0.550986	0.646583	-0.791200
C	-0.591208	2.665379	0.111068
C	0.029079	3.665603	-0.654619
C	-1.328422	3.028094	1.249287
C	-0.081315	5.003025	-0.282780
H	0.582903	3.392371	-1.546935
C	-1.419949	4.365638	1.630209
H	-1.806962	2.258102	1.846419
C	-0.800484	5.355287	0.862539
H	0.392777	5.770399	-0.886578
H	-1.975604	4.635552	2.522852
H	-0.880710	6.398148	1.153961
C	-3.230110	-1.517835	-0.191008
C	-3.396268	-2.787758	0.388661
C	-4.358116	-0.867039	-0.723873
C	-4.658208	-3.374255	0.463210
H	-2.537270	-3.313434	0.790100
C	-5.616575	-1.459105	-0.654514
H	-4.247198	0.091693	-1.218741
C	-5.772754	-2.712330	-0.056151
H	-4.769738	-4.350712	0.924326
H	-6.474527	-0.945923	-1.077619
H	-6.754421	-3.173075	-0.004168
C	-0.036750	-2.034792	0.636974
H	0.791123	-2.722644	0.472995
H	-0.132646	-1.583204	1.630698
S	2.125926	1.163320	-0.604459
O	2.635283	1.587458	-1.924590
O	2.356274	2.049543	0.554170
C	2.825203	-0.435904	-0.203709
C	3.112417	-0.743042	1.125760
C	3.001358	-1.380725	-1.217476
C	3.582180	-2.018406	1.440240
H	2.965315	0.005156	1.896345
C	3.471192	-2.647812	-0.885933
H	2.767323	-1.125995	-2.245155
C	3.767091	-2.987967	0.444987
H	3.805571	-2.263606	2.474580
H	3.607690	-3.388080	-1.669128
C	4.299846	-4.357628	0.785613
H	3.854885	-5.127803	0.148961
H	5.385212	-4.397538	0.633910
H	4.104156	-4.615924	1.829723

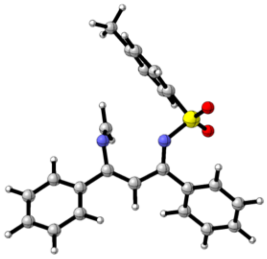

TS3-N

C	0.702357	0.914460	-0.253365
C	2.046953	-1.178139	-0.054957
C	1.863622	0.135877	-0.513692
H	2.733290	0.698778	-0.823321
N	1.030164	-1.882408	0.481677
N	-0.467851	0.329432	-0.011779
C	0.908271	2.395950	-0.224048
C	0.682961	3.142750	0.940909
C	1.391493	3.034462	-1.374689
C	0.931423	4.514399	0.946192
H	0.314257	2.651719	1.834249
C	1.624375	4.409708	-1.366818
H	1.568247	2.455669	-2.276078

C	1.397475	5.152169	-0.206099
H	0.760754	5.085176	1.853791
H	1.983460	4.898852	-2.267092
H	1.584101	6.221682	-0.198777
C	3.395220	-1.794831	0.009017
C	3.494448	-3.195322	0.089181
C	4.578569	-1.036122	-0.016765
C	4.738643	-3.819990	0.124113
H	2.583822	-3.783833	0.116282
C	5.822855	-1.662398	0.021508
H	4.533675	0.047133	-0.037784
C	5.907773	-3.055376	0.088437
H	4.797054	-4.902842	0.178014
H	6.726957	-1.061512	0.008440
H	6.878465	-3.541079	0.118075
C	-0.179370	-1.708636	-0.006273
H	-1.028364	-2.034460	0.589546
H	-0.344610	-1.670104	-1.084923
S	-1.868159	1.123755	0.503098
O	-1.841061	1.344864	1.961839
O	-2.157146	2.262610	-0.385739
C	-3.044781	-0.175022	0.160367
C	-3.389519	-0.443642	-1.166955
C	-3.587177	-0.909438	1.213612
C	-4.286727	-1.472881	-1.433471
H	-2.961462	0.142467	-1.973022
C	-4.487706	-1.935197	0.926191
H	-3.303675	-0.681162	2.234725
C	-4.849542	-2.232778	-0.394611
H	-4.556926	-1.691733	-2.462513
H	-4.913111	-2.513126	1.741332
C	-5.842905	-3.326004	-0.699581
H	-5.913621	-4.044709	0.120843
H	-6.842536	-2.903734	-0.857217
H	-5.572033	-3.866203	-1.611451

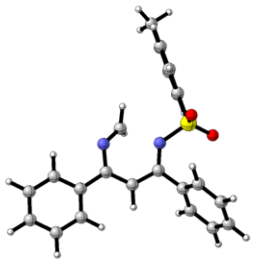

4b

C	-0.914466	-0.530419	-1.547581
C	0.271166	-1.240298	-1.059680
C	1.434607	-0.562910	-0.886021
N	-0.818262	0.560004	-2.239874
H	0.208376	-2.289963	-0.802626
C	0.545656	0.979847	-2.508311
H	0.963142	0.404676	-3.347393
H	0.566823	2.036894	-2.769816
C	2.636040	-1.112558	-0.237844
C	3.915215	-0.689704	-0.636752
C	2.520559	-2.081313	0.773984
C	5.051811	-1.241751	-0.052574
H	4.002841	0.068606	-1.406318
C	3.660493	-2.629561	1.358396
H	1.536527	-2.381466	1.120164
C	4.928769	-2.214034	0.944493
H	6.035534	-0.913173	-0.373515
H	3.559113	-3.370870	2.145022
H	5.816675	-2.638785	1.402866
C	-2.265556	-1.032175	-1.183828
C	-2.453752	-1.863314	-0.068430
C	-3.388354	-0.626519	-1.924041
C	-3.734145	-2.281904	0.296331
H	-1.605752	-2.157611	0.540399
C	-4.664948	-1.047090	-1.561426
H	-3.240895	0.022307	-2.780300
C	-4.842837	-1.877002	-0.449474
H	-3.864383	-2.918464	1.166239
H	-5.523526	-0.730157	-2.145877
H	-5.838921	-2.204322	-0.166973

N	1.480704	0.778270	-1.377070
S	1.451265	2.010052	-0.144116
O	2.645443	1.800397	0.679021
O	1.250411	3.275202	-0.861660
C	0.013585	1.658228	0.854372
C	-1.211023	2.240038	0.518626
C	0.114488	0.713716	1.877375
C	-2.348070	1.864489	1.224744
H	-1.267088	2.961814	-0.287672
C	-1.038799	0.347422	2.569256
H	1.074888	0.274352	2.122704
C	-2.283986	0.904710	2.247653
H	-3.304627	2.310680	0.969531
H	-0.970164	-0.389649	3.363834
C	-3.540055	0.453865	2.947585
H	-3.320462	-0.011765	3.911798
H	-4.227601	1.288521	3.112659
H	-4.063054	-0.285746	2.329733

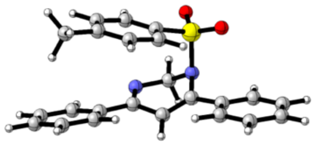

3f

O	0.756444	-2.071631	0.089995
C	-0.944359	-0.713633	-0.261989
N	-0.535145	-1.691514	0.469050
C	1.288808	-1.121242	-0.888972
H	1.678491	-1.714836	-1.720191
C	0.059223	-0.289869	-1.309150
H	-0.302271	-0.542538	-2.311595
H	0.256724	0.784732	-1.285247
C	-2.272432	-0.113997	-0.080844
C	-2.711795	0.898234	-0.949597
C	-3.126358	-0.538224	0.955505
C	-3.974251	1.471128	-0.789376
H	-2.070595	1.240070	-1.755453
C	-4.383294	0.035964	1.111478
H	-2.791408	-1.316939	1.632065
C	-4.813358	1.043312	0.239874
H	-4.299567	2.252005	-1.469955
H	-5.031133	-0.299944	1.915567
H	-5.794848	1.490256	0.365416
C	2.397842	-0.298307	-0.269032
C	2.358344	0.056809	1.084697
C	3.452181	0.160668	-1.066755
C	3.356378	0.866108	1.628958
H	1.550794	-0.311920	1.708542
C	4.449719	0.972311	-0.522996
H	3.493929	-0.118480	-2.116715
C	4.403417	1.327774	0.826856
H	3.318339	1.133957	2.680855
H	5.264734	1.319892	-1.151047
H	5.180882	1.955179	1.252508

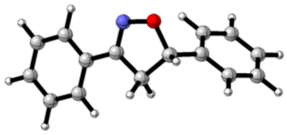

Int6

O	0.804652	0.295801	0.426862
N	1.700858	-0.777485	0.257984
C	2.901240	-0.317814	0.187585
Zn	-1.459399	0.065496	1.019237
C	-1.967596	-1.367838	-0.387037
H	-1.592817	-2.379492	-0.215046
H	-1.760046	-1.109399	-1.428249
C	-1.497320	1.503071	2.483185
H	-0.663436	1.369307	3.184941
H	-2.423887	1.492992	3.069644
H	-1.392933	2.501054	2.041572
I	-4.212901	-1.705109	-0.429937
C	2.974709	1.190265	0.274517
H	3.239388	1.611347	-0.702023
H	3.713362	1.534714	1.001037
C	1.539791	1.542076	0.695128

H	1.482052	1.714114	1.774469
C	4.055955	-1.202771	-0.014552
C	5.339980	-0.655805	-0.172044
C	3.899328	-2.601736	-0.055668
C	6.443261	-1.487641	-0.368890
H	5.482876	0.419524	-0.144851
C	5.002354	-3.426327	-0.250524
H	2.910397	-3.029851	0.068225
C	6.278779	-2.872943	-0.407924
H	7.429936	-1.051118	-0.490139
H	4.869559	-4.503642	-0.279071
H	7.137814	-3.519822	-0.558540
C	0.878703	2.669186	-0.053114
C	0.481389	2.495679	-1.385812
C	0.673135	3.903655	0.571228
C	-0.114573	3.546566	-2.083215
H	0.626999	1.532153	-1.864798
C	0.084638	4.958822	-0.130000
H	0.966879	4.037066	1.608788
C	-0.311912	4.781606	-1.457296
H	-0.426212	3.402232	-3.113509
H	-0.071832	5.913409	0.363495
H	-0.776755	5.598958	-2.000592

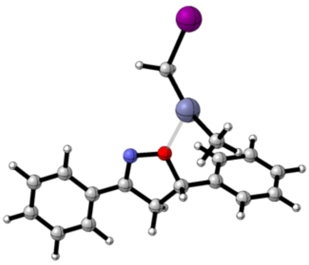

TS4

O	1.108340	-0.898614	-0.920119
N	0.698036	0.380180	-0.527374
C	1.638203	1.243661	-0.701277
Zn	-1.476665	-1.746999	-0.993926
C	-1.385899	0.114029	-0.076290
H	-1.613479	1.062123	-0.556139
H	-1.153499	0.244239	0.977507
C	-1.502905	-3.586460	-1.893138
H	-1.186814	-3.516726	-2.940606
H	-2.505520	-4.028482	-1.873823
H	-0.821648	-4.277828	-1.383277
I	-4.102339	0.017891	0.708347
C	2.872361	0.609231	-1.303921
H	3.786732	0.905175	-0.786383
H	2.961914	0.901054	-2.355969
C	2.554280	-0.888874	-1.130203
H	2.732041	-1.453681	-2.047686
C	1.501892	2.665765	-0.375649
C	2.459016	3.574554	-0.860308
C	0.438950	3.146310	0.412677
C	2.345250	4.935385	-0.579307
H	3.289178	3.223460	-1.464163
C	0.332548	4.505308	0.691891
H	-0.291106	2.454413	0.814556
C	1.282307	5.404514	0.195125
H	3.087992	5.627129	-0.964184
H	-0.489607	4.863795	1.303363
H	1.196209	6.463927	0.416514
C	3.262869	-1.534370	0.042849
C	2.667923	-1.606227	1.307275
C	4.561334	-2.027938	-0.136313
C	3.367873	-2.161128	2.381126
H	1.657586	-1.237192	1.447867
C	5.260385	-2.580182	0.937822
H	5.025444	-1.981482	-1.118498
C	4.664691	-2.647234	2.200417
H	2.897492	-2.214663	3.358607
H	6.265359	-2.963469	0.787542
H	5.206196	-3.080481	3.036104

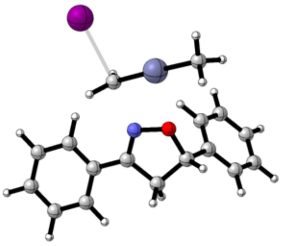

Int7

O	1.228226	-1.380573	-0.601104
N	0.780686	-0.105687	-0.208984
C	1.535407	0.852071	-0.673713
Zn	-1.960229	-1.119719	-0.856767
C	-0.491788	-0.162715	0.417389
H	-0.832959	0.844317	0.634114
H	-0.424298	-0.769625	1.322550
C	-2.311301	-2.263061	-2.528323
H	-1.386788	-2.465646	-3.082591
H	-3.008987	-1.761698	-3.209548
H	-2.752752	-3.227723	-2.250632
I	-4.258877	-0.211681	0.811749
C	2.571522	0.263906	-1.592305
H	3.543197	0.746482	-1.483301
H	2.246898	0.360929	-2.635602
C	2.576142	-1.202977	-1.123971
H	2.650879	-1.898202	-1.962111
C	1.396355	2.271554	-0.360336
C	1.764680	3.212333	-1.340198
C	0.976554	2.724304	0.905505
C	1.678480	4.576435	-1.070921
H	2.105110	2.876277	-2.314371
C	0.906140	4.088678	1.170361
H	0.735424	2.011622	1.686000
C	1.247155	5.017925	0.182380
H	1.951864	5.293543	-1.838313
H	0.590712	4.427362	2.152048
H	1.185251	6.081127	0.392509
C	3.623477	-1.504688	-0.069885
C	3.296151	-1.637550	1.282353
C	4.960603	-1.623669	-0.471522
C	4.298797	-1.877852	2.225665
H	2.261028	-1.566223	1.597818
C	5.960089	-1.863849	0.470951
H	5.219351	-1.529689	-1.523426
C	5.630894	-1.989628	1.824030
H	4.035307	-1.979757	3.274238
H	6.993161	-1.956970	0.149523
H	6.408218	-2.178365	2.558364

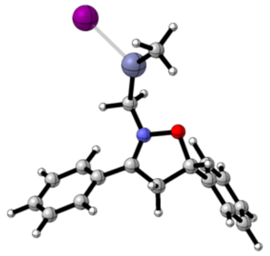

Int8

O	0.914677	-1.416423	0.195606
N	0.439985	-0.215000	0.744836
C	0.671304	0.813911	-0.019853
Zn	-2.094133	-1.653271	1.195168
C	-0.406813	-0.449695	1.859101
H	-0.768476	0.501777	2.238446
H	0.146484	-1.003242	2.619943
C	-3.253989	-3.296536	1.609130
H	-3.015718	-3.721438	2.591903
H	-3.099022	-4.081196	0.858957
H	-4.319653	-3.038046	1.604907
I	-2.612595	-0.110972	-1.327532
C	1.283021	0.353213	-1.311347
H	2.093378	1.004078	-1.642083
H	0.504484	0.323987	-2.082202
C	1.768722	-1.059280	-0.931306
H	1.535028	-1.788240	-1.709173
C	0.389686	2.205011	0.327230
C	-0.002356	3.086342	-0.695975
C	0.570175	2.697782	1.632589
C	-0.240924	4.428478	-0.409773
H	-0.142923	2.713401	-1.704651
C	0.340587	4.043357	1.908027
H	0.914873	2.035983	2.419818
C	-0.072010	4.909835	0.891207
H	-0.557956	5.098114	-1.202950
H	0.491376	4.417566	2.915703

H	-0.253889	5.957329	1.110966
C	3.233252	-1.122085	-0.551479
C	3.654595	-1.046701	0.779764
C	4.187472	-1.213510	-1.572968
C	5.017904	-1.055053	1.084863
H	2.921172	-0.995245	1.577437
C	5.548519	-1.220199	-1.267059
H	3.864417	-1.280417	-2.609082
C	5.967087	-1.139481	0.064234
H	5.336655	-0.998136	2.121496
H	6.280359	-1.294647	-2.065915
H	7.026236	-1.147954	0.303640

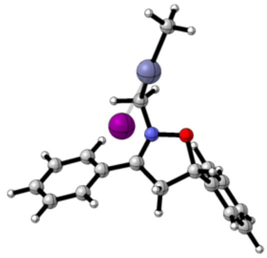

TS5

O	0.277343	-1.440017	0.288154
N	0.243145	0.087005	0.934365
C	0.735724	0.868778	0.015843
Zn	-1.994690	-1.559583	0.835431
C	-0.560796	0.143030	1.992276
H	-1.214121	1.005211	2.053673
H	-0.261599	-0.402481	2.880819
C	-2.714397	-3.335614	1.564019
H	-2.065983	-3.723433	2.357888
H	-2.762568	-4.085187	0.765779
H	-3.721805	-3.208135	1.975309
I	-2.960232	0.443396	-0.953087
C	0.924332	0.163521	-1.294266
H	1.784683	0.535525	-1.851116
H	0.020936	0.283404	-1.901728
C	1.085833	-1.323632	-0.869099
H	0.650330	-1.966587	-1.641006
C	1.112127	2.260300	0.268851
C	1.366645	3.101780	-0.831994
C	1.242384	2.784794	1.570895
C	1.737483	4.430092	-0.634954
H	1.258042	2.725384	-1.842898
C	1.631863	4.106161	1.758943
H	1.047675	2.154599	2.431012
C	1.875370	4.936213	0.658872
H	1.918390	5.068124	-1.494206
H	1.740418	4.493343	2.767217
H	2.166494	5.970900	0.811281
C	2.531776	-1.708287	-0.608174
C	3.036706	-1.808066	0.691602
C	3.387170	-1.929618	-1.695420
C	4.383543	-2.116007	0.901760
H	2.371615	-1.652707	1.534133
C	4.731524	-2.238316	-1.486252
H	2.998809	-1.861388	-2.709075
C	5.234239	-2.329615	-0.184704
H	4.766549	-2.190147	1.915575
H	5.384766	-2.410722	-2.336722
H	6.280469	-2.570182	-0.020234

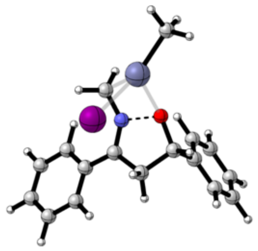

Int9

O	-0.336013	0.939659	-0.503077
N	0.105193	-1.891670	-0.404136
C	1.008949	-1.370785	0.326422
Zn	-2.166795	1.501828	-0.653525
C	-0.942399	-2.160374	-1.039184
H	-1.469643	-3.095252	-0.862226
H	-1.275316	-1.498913	-1.836842
C	-3.559646	2.881793	-1.220897
H	-3.091402	3.795996	-1.604305

H	-4.193041	3.155682	-0.369157
H	-4.212594	2.481043	-2.005042
I	-3.575213	-1.037153	0.514307
C	0.527043	-0.383201	1.345775
H	1.183823	-0.345515	2.215354
H	-0.477995	-0.668972	1.662866
C	0.393038	1.049505	0.656861
H	-0.106144	1.650232	1.441472
C	2.426704	-1.672700	0.065338
C	3.436978	-1.150609	0.891231
C	2.786160	-2.470865	-1.038875
C	4.776096	-1.409705	0.609232
H	3.190099	-0.522386	1.736320
C	4.123661	-2.727238	-1.314801
H	2.014600	-2.880745	-1.682392
C	5.122959	-2.194970	-0.491758
H	5.547296	-0.991543	1.247722
H	4.390065	-3.338344	-2.171133
H	6.167864	-2.392735	-0.710505
C	1.774344	1.660815	0.434270
C	2.339760	1.684095	-0.843868
C	2.513028	2.165463	1.512536
C	3.628941	2.184060	-1.040566
H	1.752705	1.300684	-1.671402
C	3.802637	2.664602	1.320885
H	2.077649	2.160800	2.510066
C	4.366746	2.671127	0.041270
H	4.059277	2.191250	-2.038451
H	4.364807	3.051907	2.166219
H	5.369838	3.059422	-0.110740

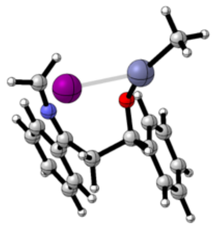

TS6

O	0.681773	1.417217	0.386523
N	-0.646739	-0.853284	1.288336
C	-1.266719	-0.901797	0.172974
Zn	2.604343	1.385873	0.110594
C	0.308541	-0.523598	2.036028
H	1.194820	-1.158979	2.097860
H	0.212193	0.340138	2.690226
C	4.266846	2.545564	0.374460
H	4.580064	2.535739	1.425439
H	4.070997	3.587178	0.093091
H	5.106475	2.182297	-0.228421
I	3.085875	-1.639197	-0.359991
C	-0.600646	-0.188821	-0.970229
H	-1.224413	-0.242156	-1.860973
H	0.336467	-0.719069	-1.173382
C	-0.228779	1.313384	-0.651157
H	0.179221	1.689187	-1.607630
C	-2.547931	-1.616504	0.079325
C	-3.469432	-1.306832	-0.935478
C	-2.873108	-2.597434	1.035964
C	-4.695854	-1.966566	-0.987382
H	-3.251316	-0.529836	-1.658525
C	-4.091889	-3.262465	0.969205
H	-2.159711	-2.842335	1.816674
C	-5.006207	-2.947313	-0.042622
H	-5.408446	-1.713854	-1.765752
H	-4.330902	-4.026379	1.702045
H	-5.958500	-3.466280	-0.092632
C	-1.523098	2.079472	-0.373655
C	-1.835893	2.510038	0.918199
C	-2.442109	2.312010	-1.407290
C	-3.051688	3.149410	1.178532
H	-1.108448	2.348241	1.706264
C	-3.656724	2.949189	-1.151306
H	-2.202921	1.996493	-2.421053
C	-3.967333	3.367081	0.146869
H	-3.282467	3.479785	2.187749
H	-4.357649	3.123541	-1.962974
H	-4.912015	3.863862	0.348534

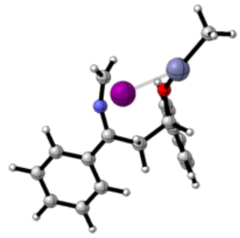

4f

C	-0.861741	0.860216	-0.065872
N	-0.411606	1.268482	-1.194700
C	0.879755	1.905628	-1.269934
H	1.541164	1.222688	-1.827701
H	0.780064	2.828457	-1.850605
O	1.486966	2.292982	-0.042267
C	-0.089940	1.022464	1.235362
H	-0.478398	1.893689	1.774884
H	-0.234451	0.159085	1.889702
C	1.393859	1.249832	0.944472
H	1.878098	1.670427	1.831416
C	-2.191039	0.186534	-0.031805
C	-2.875973	-0.021555	1.176080
C	-2.788463	-0.249907	-1.227661
C	-4.124977	-0.645452	1.188113
H	-2.447931	0.313673	2.114687
C	-4.029466	-0.878945	-1.214108
H	-2.258222	-0.089535	-2.159737
C	-4.704217	-1.079026	-0.004773
H	-4.643145	-0.791813	2.131068
H	-4.472599	-1.217190	-2.146193
H	-5.672698	-1.570158	0.005438
C	2.182925	0.017887	0.509809
C	3.551429	0.184755	0.247349
C	1.615800	-1.248606	0.331567
C	4.336084	-0.887220	-0.172144
H	3.994465	1.169459	0.367631
C	2.400672	-2.327212	-0.087925
H	0.558244	-1.410370	0.511579
C	3.760889	-2.150948	-0.339863
H	5.394209	-0.739549	-0.367621

H	1.943534	-3.303406	-0.220311
H	4.369596	-2.989358	-0.665215

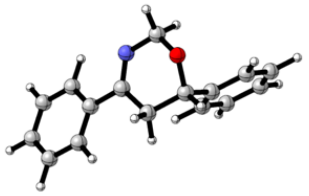

3c

C	0.872660	0.970911	0.048275
C	0.126732	-0.217166	-0.166678
C	0.712245	-1.491510	-0.217399
C	2.085328	-1.543237	-0.040513
C	2.856859	-0.372101	0.176434
C	2.268471	0.880952	0.221513
C	-0.101592	2.014476	0.025744
H	0.114133	-2.379119	-0.388468
H	2.587421	-2.505507	-0.069754
H	3.930164	-0.467402	0.307492
H	2.863604	1.773975	0.385656
H	0.047396	3.078448	0.149715
N	-1.165036	0.201892	-0.301877
N	-1.322751	1.536662	-0.184401
O	-2.242706	-0.607989	-0.541010
C	-2.950860	-0.891191	0.689952
H	-3.774066	-1.543494	0.396513
H	-2.294961	-1.403617	1.400428
H	-3.336592	0.033939	1.126474

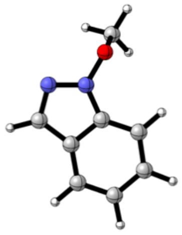

Int1-OMe

C	-3.329198	-0.989021	0.780492
C	-3.373979	-0.168288	-0.377592
C	-4.471946	-0.133675	-1.251712

C	-5.539380	-0.952146	-0.923033
C	-5.524586	-1.780587	0.230737
C	-4.435475	-1.810263	1.083806
C	-2.055403	-0.731591	1.363384
H	-4.472202	0.496859	-2.133183
H	-6.414045	-0.964189	-1.566130
H	-6.388845	-2.402824	0.440096
H	-4.426149	-2.446840	1.962815
H	-1.611024	-1.153825	2.253494
N	-2.180863	0.491640	-0.391294
N	-1.381434	0.159662	0.643887
Zn	0.855014	0.926688	0.624586
C	1.625879	-0.859968	-0.107782
H	1.313732	-1.133490	-1.119165
H	1.495025	-1.741684	0.525143
C	0.924846	2.889380	1.225827
H	0.077846	3.149605	1.874218
H	0.899065	3.573757	0.367913
H	1.841305	3.113684	1.786050
I	3.891399	-0.792755	-0.331926
O	-1.734871	1.352113	-1.351366
C	-2.016751	2.730148	-0.981913
H	-1.644467	3.320429	-1.819428
H	-3.093751	2.874904	-0.860847
H	-1.480025	2.989031	-0.066997

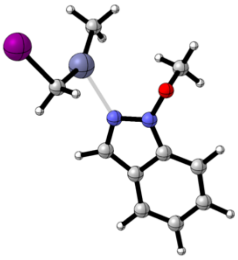

TS1-OMe

C	-3.367169	-0.990532	0.829805
C	-3.302741	-0.212484	-0.356392
C	-4.406748	-0.006861	-1.198716
C	-5.590130	-0.613465	-0.811987
C	-5.683635	-1.397344	0.368813
C	-4.589610	-1.594126	1.192481
C	-2.050925	-0.948385	1.370654
H	-4.325707	0.584535	-2.103293
H	-6.473600	-0.490614	-1.430834
H	-6.636284	-1.851073	0.622585
H	-4.662207	-2.196576	2.092217
H	-1.655437	-1.431773	2.252752
N	-2.009062	0.218058	-0.432657
N	-1.258692	-0.204901	0.607536
Zn	1.370170	1.529868	0.570311
C	0.859315	-0.437177	0.267814
H	0.594512	-0.846764	-0.703274
H	0.852942	-1.202628	1.039597
C	1.542040	3.551877	0.768672
H	0.676762	3.970916	1.296395
H	1.594266	4.032830	-0.215394
H	2.442226	3.833945	1.326507
I	3.516685	-1.238965	-0.309609
O	-1.466587	1.033163	-1.381902
C	-1.733112	2.431361	-1.087284
H	-1.214788	2.980827	-1.873048
H	-2.807612	2.629115	-1.128731
H	-1.331196	2.701760	-0.107646

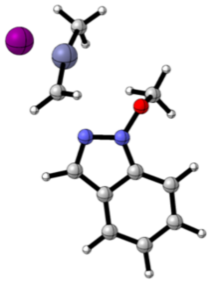

Int2-OMe

C	3.388563	-1.240854	-0.373492
C	3.478990	-0.056646	0.399259
C	4.682647	0.619479	0.627192
C	5.814295	0.062811	0.048577
C	5.755683	-1.127652	-0.718134
C	4.559273	-1.790667	-0.933336
C	2.021348	-1.614048	-0.361573

H	4.722079	1.519672	1.228690
H	6.773393	0.549736	0.192453
H	6.672359	-1.525701	-1.140495
H	4.515376	-2.705634	-1.514146
H	1.523242	-2.474356	-0.781409
N	2.211197	0.204579	0.853771
N	1.329500	-0.720275	0.346547
Zn	-1.311572	0.751556	-0.497214
C	-0.087097	-0.633629	0.612881
H	-0.213439	-0.383073	1.667816
H	-0.493058	-1.628123	0.429140
C	-1.357755	2.583996	-1.448397
H	-0.540045	2.696042	-2.171493
H	-1.266804	3.396577	-0.717214
H	-2.300598	2.724715	-1.989581
I	-3.843464	-0.670953	0.177154
O	1.750071	1.348842	1.417648
C	1.710754	2.448124	0.453744
H	1.219210	3.257802	0.991420
H	2.726591	2.732984	0.171446
H	1.126780	2.166867	-0.423107

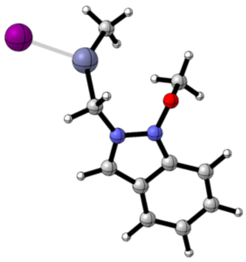

Int3-OMe

C	-0.757528	-1.014573	0.095212
C	-0.424774	0.282755	-0.350899
C	-1.342659	1.304441	-0.503133
C	-2.680774	1.014679	-0.190214
C	-3.045448	-0.270613	0.234732
C	-2.105100	-1.295412	0.376709
C	0.449148	-1.762065	0.150325
H	-1.031332	2.286443	-0.842940
H	-3.433281	1.790713	-0.282625
H	-4.087324	-0.477179	0.462881
H	-2.405616	-2.282857	0.712050
H	0.631354	-2.786873	0.430179
N	0.956371	0.295193	-0.683311
N	1.490267	-0.961920	-0.214465
C	2.805953	-1.149938	-0.240267
H	3.425868	-0.359034	-0.625438
H	3.180981	-2.106782	0.085604
O	1.714736	1.397677	-0.295220
C	1.715015	1.616330	1.139352
H	2.207460	0.785439	1.652408
H	2.289967	2.532668	1.274688
H	0.694569	1.749505	1.508483

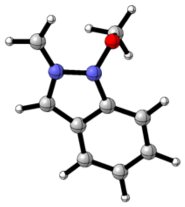

TS2-OMe

C	-0.713726	-1.034304	0.002274
C	-0.400937	0.297590	-0.378079
C	-1.363859	1.309366	-0.374895
C	-2.685099	0.957261	-0.106184
C	-3.025584	-0.375487	0.199617
C	-2.057529	-1.370296	0.261383
C	0.481918	-1.766719	0.223315
H	-1.084239	2.325229	-0.633336
H	-3.461291	1.714381	-0.153948
H	-4.062690	-0.626359	0.401378
H	-2.322269	-2.388405	0.529569
H	0.561046	-2.707333	0.753461
N	0.932739	0.398317	-0.762311
N	1.586673	-1.065033	-0.049852
C	2.860330	-1.316836	-0.157898
H	3.549488	-0.483846	-0.098835
H	3.202008	-2.315658	-0.403656
O	1.615595	1.543630	-0.384248

C	1.604146	1.817006	1.035446
H	2.127448	1.015158	1.567710
H	2.144983	2.757363	1.147172
H	0.586926	1.921959	1.419733

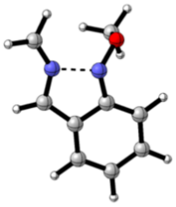

Int4-OMe

C	-0.706131	-0.923658	-0.064598
C	-0.300901	0.493252	0.028640
C	-1.345609	1.484418	0.235497
C	-2.654594	1.136413	0.134915
C	-3.053545	-0.224084	-0.133978
C	-2.117294	-1.203300	-0.232822
C	0.161974	-1.982190	0.052061
H	-1.048541	2.510786	0.404840
H	-3.423693	1.895752	0.244882
H	-4.108661	-0.459367	-0.229105
H	-2.414887	-2.236510	-0.389626
H	-0.243667	-2.989319	-0.069502
N	0.970032	0.769189	-0.135091
N	1.486839	-1.873685	0.408294
C	2.364662	-2.694594	-0.028198
H	3.377880	-2.643242	0.367853
H	2.162292	-3.449956	-0.796798
O	1.239812	2.117159	0.099990
C	2.606831	2.377403	-0.218845
H	2.806632	2.196242	-1.280959
H	2.768502	3.431922	0.013026
H	3.275193	1.755934	0.387017

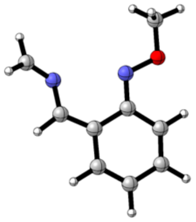

TS3-OMe

C	-0.999597	0.800051	0.189474
C	-0.146412	-0.385714	0.036208
C	-0.800179	-1.636193	-0.283128
C	-2.156291	-1.758172	-0.187934
C	-2.987213	-0.640086	0.141944
C	-2.421274	0.590623	0.300006
C	-0.573156	2.106440	-0.080087
H	-0.176946	-2.492773	-0.501889
H	-2.616428	-2.728451	-0.353942
H	-4.060424	-0.772442	0.230974
H	-3.046701	1.462173	0.475689
H	-1.347482	2.867234	-0.179293
N	1.162275	-0.285605	0.233182
N	0.653638	2.476313	-0.477122
C	1.728174	1.997393	0.072772
H	2.675123	2.091874	-0.456715
H	1.775419	1.743943	1.126076
O	1.853152	-1.373630	-0.317238
C	3.154404	-1.431211	0.262863
H	3.102700	-1.614562	1.342157
H	3.663243	-2.261774	-0.230281
H	3.704170	-0.499934	0.080002

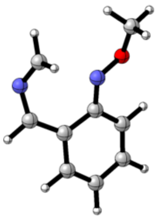

4c

C	-0.936258	0.846575	-0.112275
C	-0.230537	-0.303661	0.284305
C	-0.893535	-1.522811	0.433365
C	-2.268702	-1.588584	0.212832
C	-2.984867	-0.442515	-0.158760
C	-2.319450	0.768488	-0.328383
C	-0.167613	2.071935	-0.349253

H	-0.329040	-2.400614	0.730359
H	-2.788614	-2.533196	0.339321
H	-4.056659	-0.501183	-0.319517
H	-2.862730	1.658311	-0.635555
H	-0.671095	2.884905	-0.878856
N	1.180420	-0.220305	0.490834
N	1.049823	2.248954	0.008502
C	1.644573	1.144440	0.762543
H	2.725191	1.143515	0.606950
H	1.458651	1.335520	1.828760
O	1.806315	-0.681144	-0.745525
C	2.739794	-1.709189	-0.427432
H	2.244893	-2.581409	0.016612
H	3.198103	-1.993980	-1.378571
H	3.518656	-1.351333	0.257694

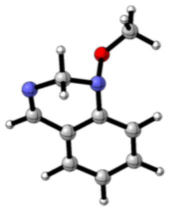

3m

C	2.454276	0.632365	-1.096544
C	1.961224	0.011800	0.072634
C	2.311904	0.450692	1.355334
C	3.170086	1.541946	1.425582
C	3.679941	2.170148	0.267832
C	3.334183	1.722400	-0.998965
C	1.850439	-0.090271	-2.178981
H	1.930846	-0.038378	2.241749
H	3.461311	1.918253	2.401324
H	4.354425	3.013317	0.376598
H	3.726646	2.198333	-1.891692
H	1.971469	0.078522	-3.241349
N	1.169545	-1.049787	-0.375324
N	1.068771	-1.055448	-1.756105
S	-0.144527	-1.770808	0.484043
O	-0.462311	-3.006034	-0.225549

O	0.292351	-1.780532	1.881557
C	-1.464612	-0.599328	0.279467
C	-1.652149	0.388569	1.248750
C	-2.245415	-0.651420	-0.878122
C	-2.653837	1.335201	1.050785
H	-1.032326	0.407533	2.137652
C	-3.239731	0.305927	-1.053936
H	-2.075949	-1.424920	-1.618028
C	-3.460269	1.309451	-0.097038
H	-2.811830	2.105807	1.799327
H	-3.854750	0.275067	-1.948343
C	-4.561954	2.321289	-0.286225
H	-5.503412	1.943533	0.130419
H	-4.332756	3.261897	0.221597
H	-4.734301	2.530524	-1.345453

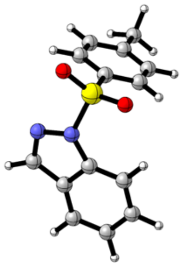

Int1-Ts

C	2.020325	2.513849	1.170977
C	1.900196	2.076463	-0.166832
C	2.705593	2.575777	-1.196388
C	3.646697	3.532526	-0.834028
C	3.776583	3.990972	0.496742
C	2.967623	3.495181	1.508671
C	1.051406	1.749428	1.898651
H	2.593437	2.235097	-2.217663
H	4.297062	3.945127	-1.598916
H	4.521394	4.746599	0.724268
H	3.060741	3.848315	2.530255
H	0.817528	1.784191	2.954514
N	0.857024	1.148584	-0.173256
N	0.398731	0.924314	1.112276
Zn	-1.609605	-0.402759	1.398481

C	-2.893557	0.967032	0.517653
H	-2.439103	1.577400	-0.266505
H	-3.483594	1.624668	1.160563
C	-0.992976	-1.972795	2.572808
H	0.001191	-1.784222	2.998879
H	-0.936349	-2.916670	2.014734
H	-1.676925	-2.145704	3.413727
I	-4.526658	-0.117344	-0.639770
S	0.712988	-0.183199	-1.269961
O	-0.563072	-0.808573	-0.907371
O	0.941576	0.393056	-2.592164
C	3.256280	-1.167590	-1.525948
C	1.885511	-2.114822	0.264640
C	4.314776	-1.978845	-1.125587
H	3.355925	-0.492188	-2.367756
C	2.959657	-2.912634	0.644978
H	0.941856	-2.158877	0.795823
C	4.184581	-2.860163	-0.040971
H	5.256017	-1.927347	-1.664536
H	2.845476	-3.588549	1.487243
C	5.326549	-3.757613	0.362881
H	6.291770	-3.332552	0.075423
H	5.330812	-3.936298	1.441708
H	5.235707	-4.733311	-0.129641

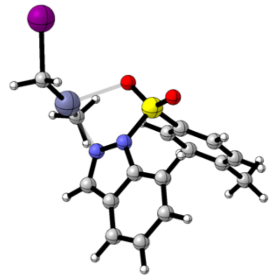

TS1-Ts

C	-2.139785	2.182728	-1.230438
C	-2.292541	1.946275	0.150639
C	-3.505622	2.146857	0.808042
C	-4.570613	2.594417	0.028481
C	-4.432777	2.840958	-1.352884
C	-3.219163	2.646564	-1.998727
C	-0.777915	1.902347	-1.520319
H	-3.608382	1.972750	1.870923
H	-5.530246	2.768343	0.504032
H	-5.289365	3.196984	-1.915332
H	-3.100595	2.847404	-3.057646
H	-0.220061	2.047290	-2.434065
N	-1.050712	1.510739	0.666559
N	-0.154531	1.505076	-0.420310
Zn	2.070868	-0.728174	-0.257888
C	1.255984	1.261499	-0.233871
H	1.535009	1.669615	0.736743
H	1.770638	1.809789	-1.023274
C	1.731212	-2.741682	-0.521706
H	1.221892	-2.955675	-1.469958
H	1.109075	-3.142348	0.287178
H	2.679507	-3.291570	-0.524949
I	4.884837	0.225807	-0.120134
S	-1.141019	0.045276	1.732277
O	0.221446	-0.461241	1.850960
O	-1.865570	0.558743	2.888982
C	-2.174470	-1.062600	0.809077
C	-3.499427	-1.230499	1.223456
C	-1.674927	-1.690350	-0.337400
C	-4.330525	-2.058845	0.475121
H	-3.863776	-0.730211	2.112264
C	-2.529655	-2.506610	-1.070262
H	-0.648174	-1.564322	-0.654663
C	-3.863939	-2.705805	-0.678959
H	-5.359325	-2.200656	0.791498
H	-2.152238	-3.000034	-1.960772
C	-4.765190	-3.619258	-1.468645
H	-5.818174	-3.360477	-1.331008
H	-4.532192	-3.583235	-2.536371
H	-4.633904	-4.657288	-1.140203

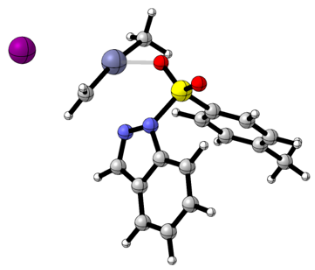

Int2-Ts

C	-2.139785	2.182728	-1.230438
C	-2.292541	1.946275	0.150639
C	-3.505622	2.146857	0.808042
C	-4.570613	2.594417	0.028481
C	-4.432777	2.840958	-1.352884
C	-3.219163	2.646564	-1.998727
C	-0.777915	1.902347	-1.520319
H	-3.608382	1.972750	1.870923
H	-5.530246	2.768343	0.504032
H	-5.289365	3.196984	-1.915332
H	-3.100595	2.847404	-3.057646
H	-0.220061	2.047290	-2.434065
N	-1.050712	1.510739	0.666559
N	-0.154531	1.505076	-0.420310
Zn	2.070868	-0.728174	-0.257888
C	1.255984	1.261499	-0.233871
H	1.535009	1.669615	0.736743
H	1.770638	1.809789	-1.023274
C	1.731212	-2.741682	-0.521706
H	1.221892	-2.955675	-1.469958
H	1.109075	-3.142348	0.287178
H	2.679507	-3.291570	-0.524949
I	4.884837	0.225807	-0.120134
S	-1.141019	0.045276	1.732277
O	0.221446	-0.461241	1.850960
O	-1.865570	0.558743	2.888982
C	-2.174470	-1.062600	0.809077
C	-3.499427	-1.230499	1.223456
C	-1.674927	-1.690350	-0.337400
C	-4.330525	-2.058845	0.475121
H	-3.863776	-0.730211	2.112264
C	-2.529655	-2.506610	-1.070262
H	-0.648174	-1.564322	-0.654663
C	-3.863939	-2.705805	-0.678959
H	-5.359325	-2.200656	0.791498
H	-2.152238	-3.000034	-1.960772
C	-4.765190	-3.619258	-1.468645
H	-5.818174	-3.360477	-1.331008
H	-4.532192	-3.583235	-2.536371
H	-4.633904	-4.657288	-1.140203

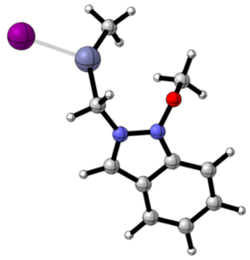

Int3-Ts

C	1.445124	1.062443	-1.166424
C	1.883361	0.281172	-0.072406
C	2.567728	0.806454	1.010482
C	2.797671	2.190027	1.018709
C	2.358225	2.988947	-0.047318
C	1.690116	2.446424	-1.145956
C	0.849671	0.183775	-2.101950
H	2.890710	0.169704	1.824062
H	3.324706	2.639229	1.853626
H	2.550074	4.057948	-0.022572
H	1.367961	3.075767	-1.968995
H	0.511021	0.355739	-3.110516
N	1.510962	-1.087412	-0.302741
N	0.953798	-1.097542	-1.637826
C	0.668425	-2.263853	-2.209779
H	0.906616	-3.171719	-1.687391
H	0.196316	-2.233616	-3.179358
S	0.300330	-1.682287	0.905654
O	-0.011143	-3.061148	0.530284
O	0.939719	-1.378569	2.185879

C	-1.110738	-0.646921	0.643534
C	-1.131441	0.627993	1.219354
C	-2.119872	-1.082653	-0.221164
C	-2.196221	1.472475	0.922735
H	-0.332267	0.945766	1.878084
C	-3.173644	-0.218205	-0.500371
H	-2.070722	-2.071221	-0.661571
C	-3.228937	1.066347	0.063083
H	-2.224862	2.464632	1.362965
H	-3.965239	-0.544084	-1.168426
C	-4.390363	1.982983	-0.225754
H	-4.087345	3.033020	-0.193701
H	-4.828858	1.776631	-1.205686
H	-5.179949	1.846758	0.523039

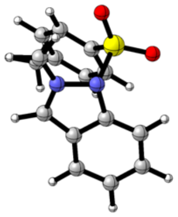

TS2-Ts

C	-1.529931	0.965374	1.124807
C	-1.908188	0.149542	0.029985
C	-2.707311	0.643543	-0.999354
C	-3.027935	2.003251	-0.989696
C	-2.619606	2.831636	0.068530
C	-1.889104	2.321845	1.138557
C	-0.988038	0.137738	2.150042
H	-3.004177	-0.007543	-1.812451
H	-3.592951	2.423309	-1.815524
H	-2.886357	3.883928	0.056584
H	-1.595165	2.957344	1.967582
H	-0.954222	0.376990	3.203952
N	-1.469398	-1.178964	0.185879
N	-0.824555	-1.127567	1.776825
C	-0.217026	-2.167322	2.287951
H	-0.551126	-3.155171	2.013444
H	0.666266	-2.005403	2.891673
S	-0.229713	-1.686537	-0.895709
O	0.151832	-3.035548	-0.469183
O	-0.788346	-1.460676	-2.234901
C	1.138007	-0.578000	-0.640440
C	1.085526	0.705593	-1.194574
C	2.197550	-0.974218	0.179488
C	2.115627	1.598477	-0.916032
H	0.255951	0.994723	-1.828951
C	3.218890	-0.064302	0.441841
H	2.213788	-1.973071	0.597620
C	3.194690	1.230498	-0.097096
H	2.081918	2.597608	-1.340427
H	4.048035	-0.364428	1.075880
C	4.318969	2.199496	0.170056
H	3.953894	3.229218	0.220349
H	4.834201	1.967574	1.105903
H	5.062436	2.156122	-0.634949

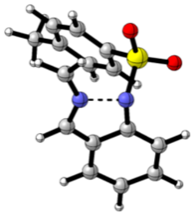

Int4-Ts

C	-2.816132	1.176768	-0.085282
C	-2.135431	-0.116201	-0.034074
C	-2.972225	-1.284734	-0.098224
C	-4.333076	-1.175038	-0.221548
C	-4.992463	0.088462	-0.281217
C	-4.245725	1.229288	-0.210277
C	-2.137925	2.391523	0.003626
H	-2.506645	-2.262015	-0.073870
H	-4.929645	-2.081400	-0.278729
H	-6.071533	0.133342	-0.376573
H	-4.722926	2.204736	-0.246703
H	-2.732930	3.305858	0.001027
N	-0.807853	-0.087186	0.066331
N	-0.847703	2.591823	0.246376

C	0.201126	2.923159	-0.366756
H	1.119462	3.081732	0.194943
H	0.234715	3.027186	-1.454260
S	0.087218	-1.453756	0.265470
O	-0.045795	-1.982336	1.644517
O	-0.085429	-2.431967	-0.836045
C	1.716193	-0.727017	0.099022
C	2.578149	-1.192702	-0.889995
C	2.113332	0.283719	0.980460
C	3.852362	-0.632213	-1.001730
H	2.248719	-1.974569	-1.564925
C	3.384189	0.834722	0.852616
H	1.429433	0.641991	1.742101
C	4.274573	0.385133	-0.137698
H	4.525147	-0.990323	-1.775870
H	3.694334	1.625271	1.530691
C	5.659806	0.974012	-0.244230
H	6.108420	0.772065	-1.220402
H	5.644620	2.057215	-0.089109
H	6.321518	0.547697	0.519269

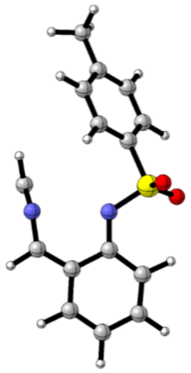

TS3-Ts

C	-2.803743	1.245235	-0.171183
C	-2.117390	-0.038821	-0.086107
C	-2.943683	-1.195628	0.091287
C	-4.313651	-1.110766	0.007040
C	-4.978763	0.126833	-0.191419
C	-4.231369	1.273469	-0.253542
C	-2.170579	2.491822	0.037220
H	-2.474467	-2.161756	0.227150
H	-4.900820	-2.021226	0.088446
H	-6.059887	0.161038	-0.266040
H	-4.717736	2.240811	-0.347054
H	-2.804326	3.378635	0.034721
N	-0.776448	-0.054993	-0.216190
N	-0.940616	2.679977	0.467589
C	0.177425	2.134613	0.157762
H	0.967720	2.082746	0.904910
H	0.444241	1.905269	-0.869146
S	0.096036	-1.436243	0.076885
O	-0.115400	-1.968735	1.442588
O	-0.048903	-2.388813	-1.047684
C	1.740840	-0.735943	0.007523
C	2.313893	-0.456235	-1.234717
C	2.410395	-0.438808	1.193720
C	3.570681	0.141707	-1.280143
H	1.782324	-0.703585	-2.147187
C	3.669792	0.157358	1.129674
H	1.946770	-0.669850	2.146249
C	4.266848	0.458382	-0.102598
H	4.020685	0.365500	-2.243242
H	4.195355	0.392759	2.050739
C	5.640301	1.079365	-0.166539
H	5.733635	1.753879	-1.022558
H	5.869179	1.640952	0.743141
H	6.408632	0.304725	-0.277191

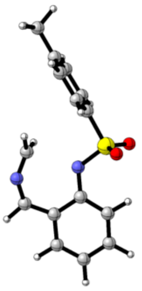

4m

C	-2.950337	1.236419	-0.066451
C	-2.197090	0.050025	0.011403
C	-2.885718	-1.167201	0.104160
C	-4.284558	-1.179935	0.018119

C	-5.021142	-0.007582	-0.148221
C	-4.337949	1.207099	-0.169391
C	-2.211494	2.474798	0.141457
H	-2.364169	-2.105319	0.233745
H	-4.795751	-2.135708	0.081541
H	-6.101981	-0.038338	-0.232393
H	-4.874849	2.149538	-0.237996
H	-2.789340	3.398566	0.230467
N	-0.780163	0.203108	-0.024786
N	-0.953205	2.537315	0.335155
C	-0.172366	1.304538	0.080907
H	0.537532	1.230873	0.904644
H	0.419222	1.507532	-0.821671
S	0.149087	-1.269312	-0.009651
O	-0.096952	-1.984348	1.252432
O	-0.110809	-1.993922	-1.261765
C	1.809252	-0.658841	-0.028986
C	2.369863	-0.261905	-1.245432
C	2.492373	-0.516495	1.179611
C	3.644905	0.293642	-1.239570
H	1.818524	-0.389209	-2.170682
C	3.766047	0.042962	1.161481
H	2.028841	-0.832054	2.107429
C	4.358674	0.456623	-0.041368
H	4.094453	0.606727	-2.176951
H	4.308379	0.161976	2.094400
C	5.749487	1.036484	-0.053591
H	5.859340	1.791974	-0.836436
H	6.002532	1.491458	0.907386
H	6.487854	0.250381	-0.251759

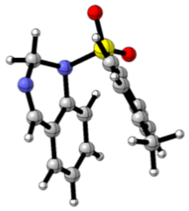

7. References

1. S. Fukuhara, S. Yugandar, S. Fuse, H. Nakamura, *ACS Omega* **2018**, *3*, 16472-16476.
2. Y. Zhang, Z. Long, Y. Le, L. Liu, L. Yan, *Heterocycles* **2022**, *104*, 1098-1110.
3. Y. Sun, A. Abdukader, H. Zhang, W. Yang, C. Liu, *RSC Adv.* **2017**, *7*, 55786-55789.
4. G. R. Kumar, Y. K. Kumar, M. S. Reddy, *Chem. Commun.* **2016**, *52*, 6589-6592.
5. X. Liu, D. Hong, Z. She, W. H. Hersh, B. Yoo, Y. Chen, *Tetrahedron* **2018**, *74*, 6593-6606.
6. L. Cecchi, F. De Sarlo, F. Machetti, *Eur. J. Org. Chem.* **2006**, 21, 4852-4860.

7 Y. Harigae, K. Moriyama, H. Togo, *J. Org. Chem.* **2014***, 79*, 2049-2058.

8. S. Bhosale, S. Kurhade, S. Vyas, V. P. Palle, D. Bhuniya, *Tetrahedron* **2010**, *66*, 9582-9588.
9. C. Spiteri, C. Mason, F. Zhang, D. J. Ritson, P. Sharma, S. Keeling, J. E. Moses, *Org. Biomol. Chem*. **2010**, 8, 2537-2542.
10. J. Li, J. Li, X. Ji, Q. Liu, L. Chen, Y. Huang, Y. Li, *Adv. Synthe. Catal.* **2021**, 363, 1059-1068.
11. D. Liang, Q. Zhu, *Asian J. Org. Chem*. **2015**, *4*, 42-45.
12. F. Lehmann, T. Koolmeister, L. R. Odell, M. Scobie, *Org. Lett*. **2009**, *11*, 5078-5081.
13. Y. Ye, I. Kevlishvili, S. Feng, P. Liu, S. L. Buchwald, *J. Am. Chem. Soc.* **2020**, *142*, 10550-10556.
14. L. Han, B. Zhang, M. Zhu, J. Yan, *Tetrahedron Lett.* **2014**, *55*, 2308-2311.
15. J. M. Pérez, D. J. Ramón, *ACS Sustainable Chem. Eng.* **2015**, *3*, 2343-2349.
16. F. Ono, Y. Ohta, M. Hasegawa, S. Kanemasa, *Tetrahedron Lett*. **2009**, *50*, 2111-2114.
17. (a) C. Yin, W. Wu, Y. Hu, X. Tan, C. You, Y. Liu, Z. Chen, X. -Q. Dong, X. Zhang, *Adv. Synth. Catal.* **2018**, *360*, 2119-2124; (b) D. E. Davies, T. L. Gilchrist, T. G. Roberts, *J. Chem. Soc. Perkin Trans 1* **1983**, 1275-1281.
18. M. Shigenobu, K. Takenaka, H. Sasai, *Angew. Chem. Int. Ed.* **2015**, *54*, 9572.
19. N. Ashwini, M. Garg, C. D. Mohan, J. E. Fuchs, S. Rangappa, S. Anusha, T. R. Swaroop, K. S. Rakesh, D. Kanojia, V. Madan, A. Bender, H. P. Koeffler, Basappa, K. S. Rangappa, *Bioorg. Med. Chem*. **2015**, *23*, 6157-2165.
20. E. LeGoff, *J. Org. Chem.* **1964**, *29*, 2048-2050.
21. A. Ben-Yahia, M. Naas, S. E. Kazzouli, E. M. Essassi, G. Guillaumet, *Eur. J. Org. Chem*. **2012**, 7072-7081.
22. Gaussian 16, Revision C.01, M. J. Frisch, G. W. Trucks, H. B. Schlegel, G. E. Scuseria, M. A. Robb, J. R. Cheeseman, G. Scalmani, V. Barone, G. A. Petersson, H. Nakatsuji, X. Li, M. Caricato, A. V. Marenich, J. Bloino, B. G. Janesko, R. Gomperts, B. Mennucci, H. P. Hratchian, J. V. Ortiz, A. F. Izmaylov, J. L. Sonnenberg, D. Williams-Young, F. Ding, F. Lipparini, F. Egidi, J. Goings, B. Peng, A. Petrone, T. Henderson, D. Ranasinghe, V. G. Zakrzewski, J. Gao, N. Rega, G. Zheng, W. Liang, M. Hada, M. Ehara, K. Toyota, R. Fukuda, J. Hasegawa, M. Ishida, T. Nakajima, Y. Honda, O. Kitao, H. Nakai, T. Vreven, K. Throssell, J. A. Montgomery, Jr., J. E. Peralta, F. Ogliaro, M. J. Bearpark, J. J. Heyd, E. N. Brothers, K. N. Kudin, V. N. Staroverov, T. A. Keith, R. Kobayashi, J. Normand, K. Raghavachari, A. P. Rendell, J. C. Burant, S. S. Iyengar, J. Tomasi, M. Cossi, J. M. Millam, M. Klene, C. Adamo, R. Cammi, J. W. Ochterski, R. L. Martin, K. Morokuma, O. Farkas, J. B. Foresman, D. J. Fox, Gaussian, Inc., Wallingford CT, 2016.
23. (a) A. D. Becke, *J. Chem. Phys.* **1993**, *98*, 5648-5652; (b) P. J. Stephens, F. J. Devlin, C. F. Chabalowski, M.

J. Frisch, *J. Phys. Chem.* **1994,** *98*, 11623-11627; (c) S. Grimme, J. Antony, S. Ehrlich, S. Krieg, *J. Chem. Phys.* **2010**, *132*, 154104.

24. S. Grimme, J. Antony, S. Ehrlich, H. Krieg, *J. Chem. Phys*. **2010**, *132*, 154104.
25. R. Ditchfield, W. J. Hehre, J. A. Pople, *J. Chem. Phys*. **1971**, *54*, 724-728.
26. (a) P. J. Hay, W. R. Wadt, *J. Chem. Phys.* **1985**, *82*, 270-283; (b) W. R. Wadt, P. J. Hay, *J. Chem. Phys.* **1985**, *82*, 284-298; (c) P. J. Hay, W. R. Wadt, *J. Chem. Phys.* **1985**, *82*, 299-310.
27. K. Fukui, *J. Phys. Chem*. **1970**, *74*, 4161–4163.
28. J. Tomasi, B. Mennucci, R. Cammi, *Chem. Rev.* **2005**, *105*, 2999–3093.
29. All molecular graphics were prepared with CYLview20; Legault, C. Y., Université de Sherbrooke, 2020 (http://www.cylview.org)

8. NMR spectra

3-Phenyl-5-(triisopropylsilyl)isoxazole (1q)

^{1}H NMR (500 MHz, $CDCl_3$)

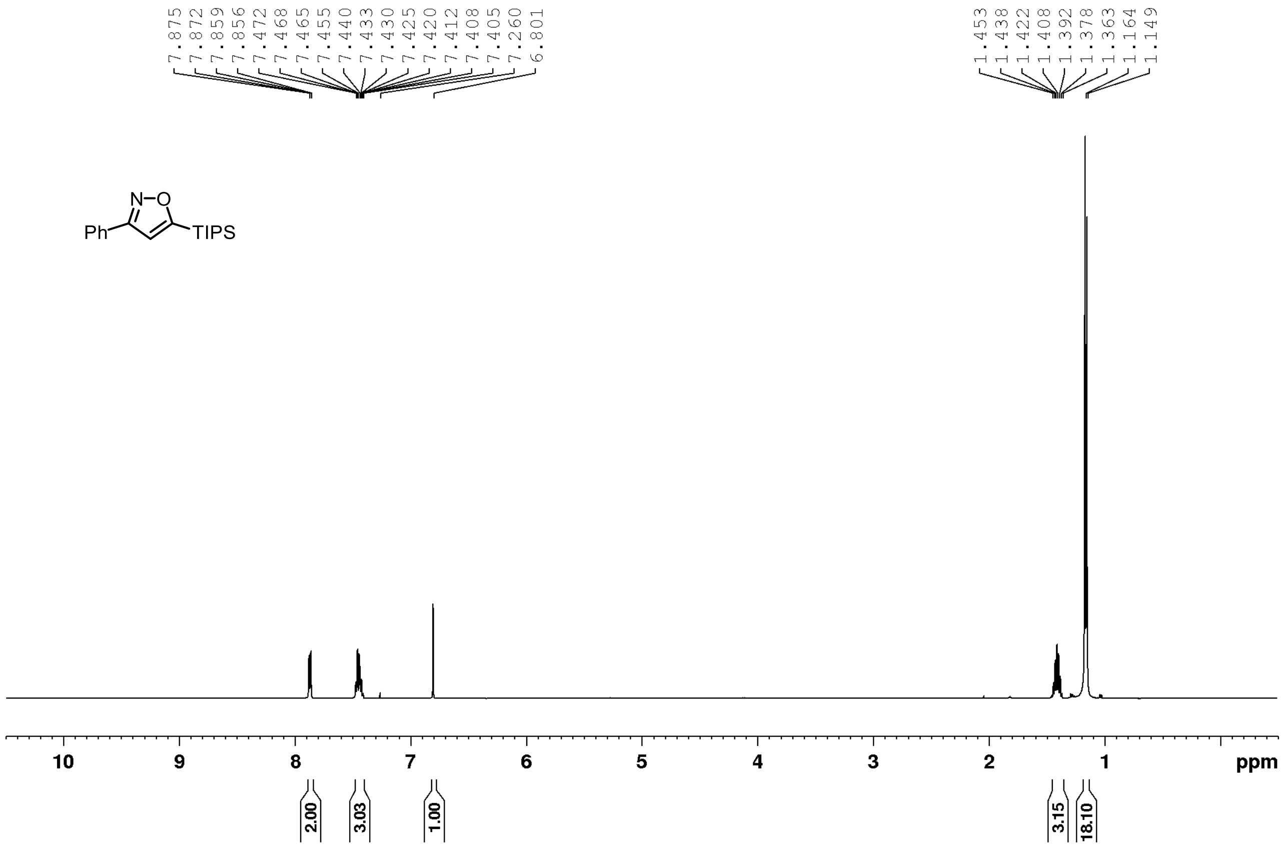

^{13}C NMR (125 MHz, $CDCl_3$)

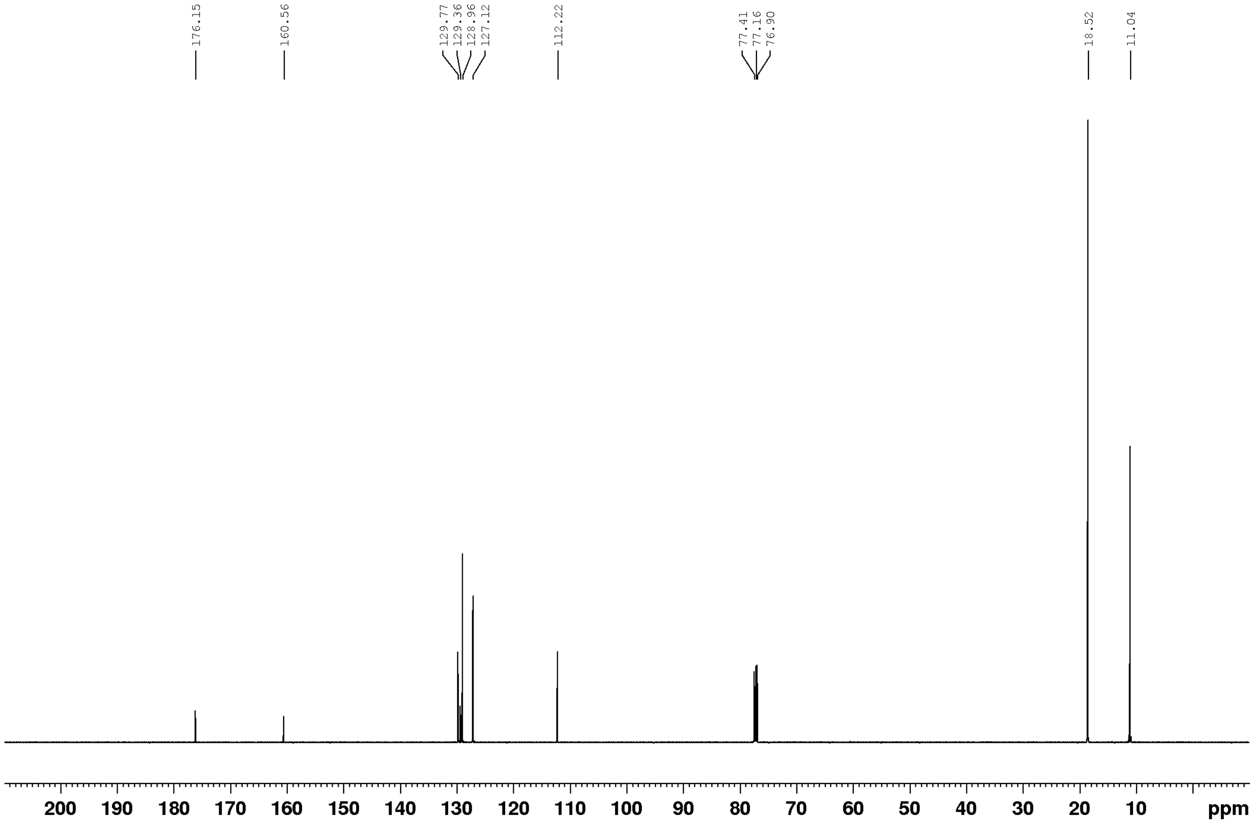

4,6-Diphenyl-2*H*-1,3-oxazine (2a)

^{1}H NMR (400 MHz, CDCl$_3$)

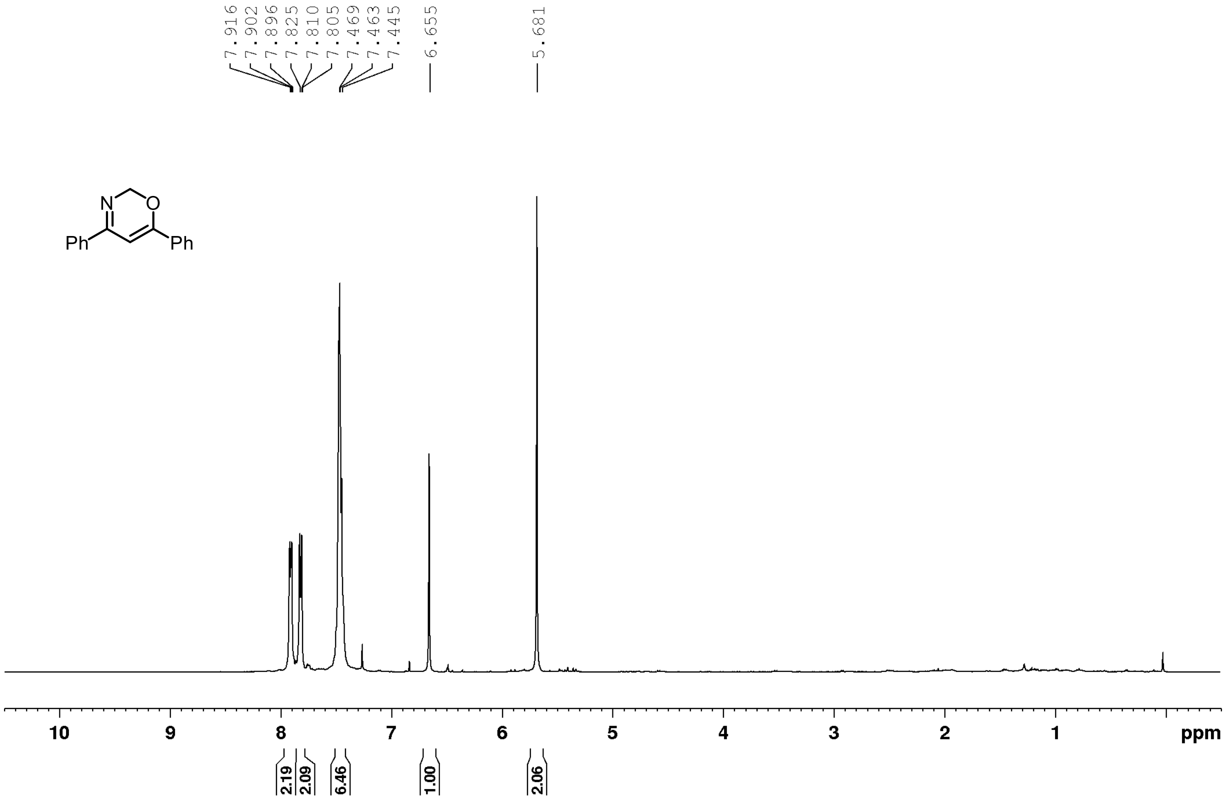

^{13}C NMR (100 MHz, CDCl$_3$)

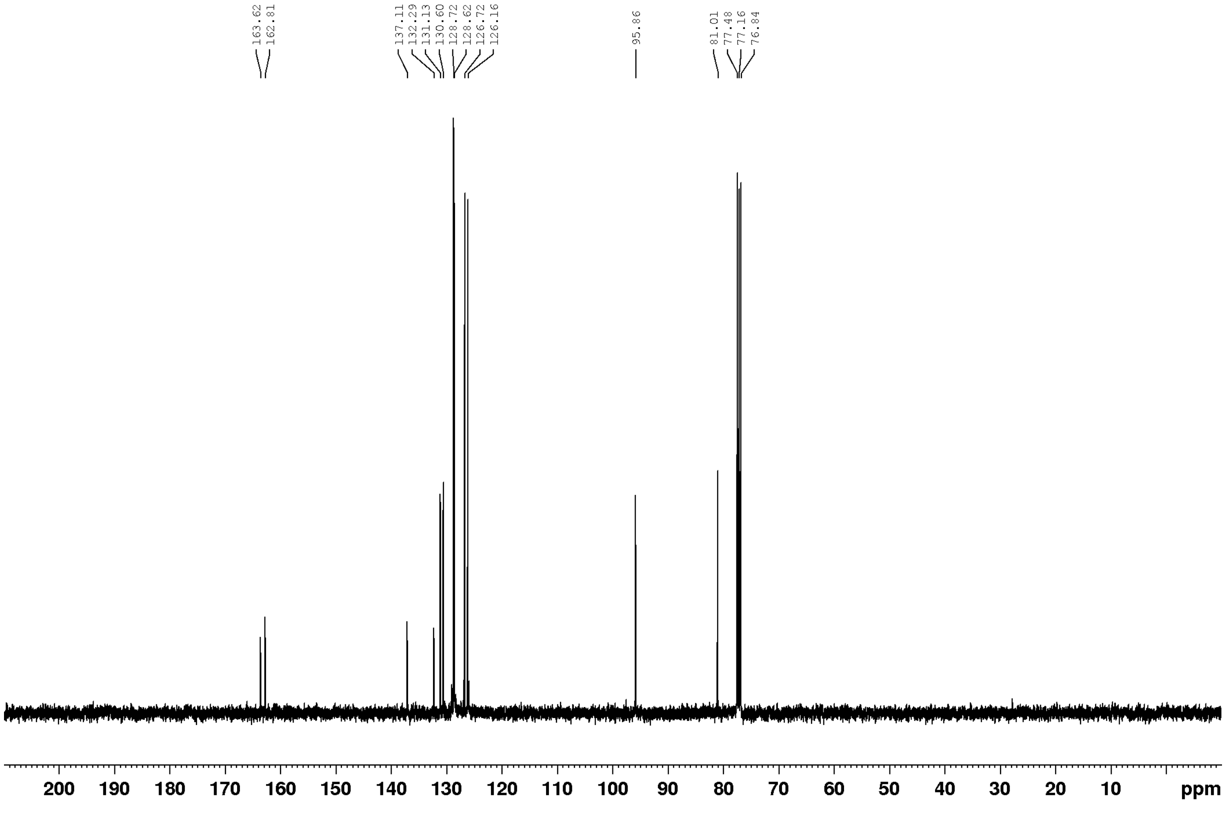

6-Phenyl-4-(*p*-tolyl)-2*H*-1,3-oxazine (2b)

^{1}H NMR (500 MHz, CDCl$_3$)

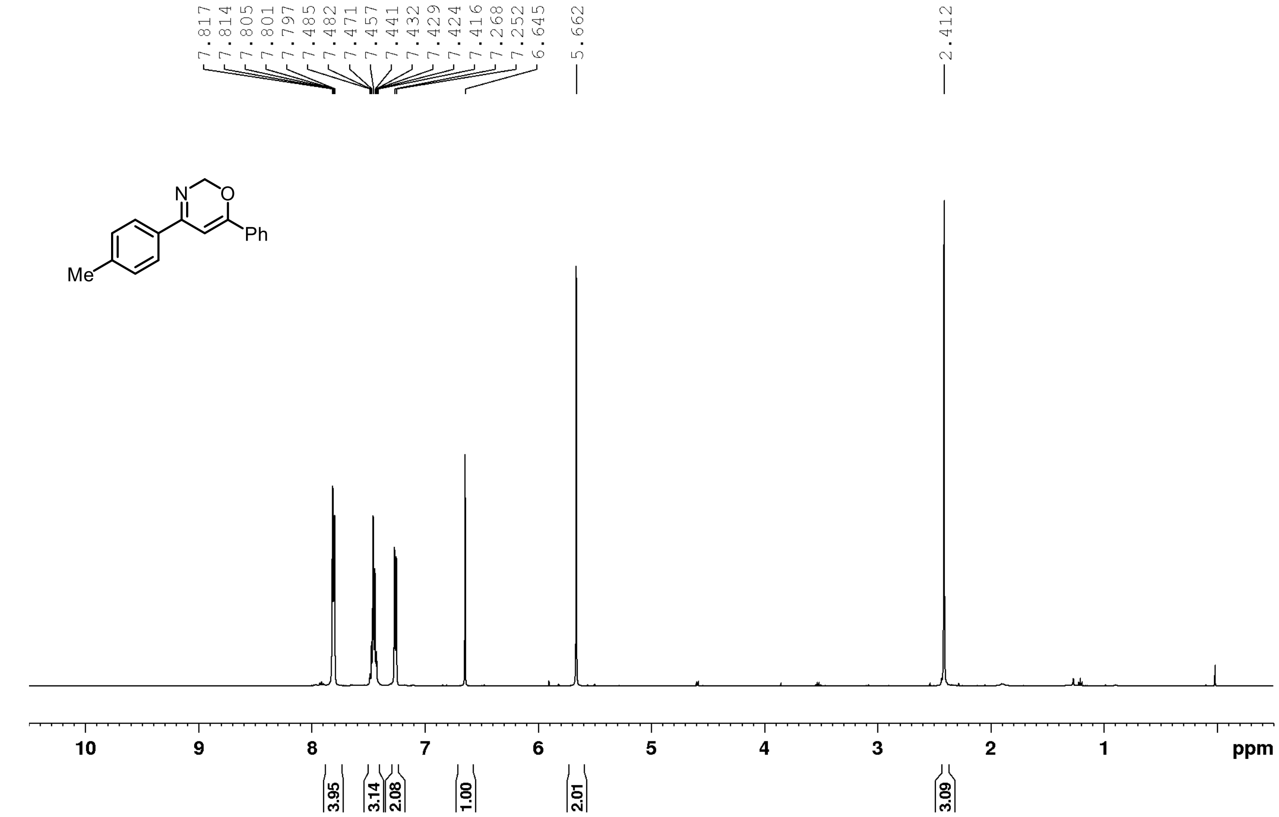

^{13}C NMR (125 MHz, CDCl$_3$)

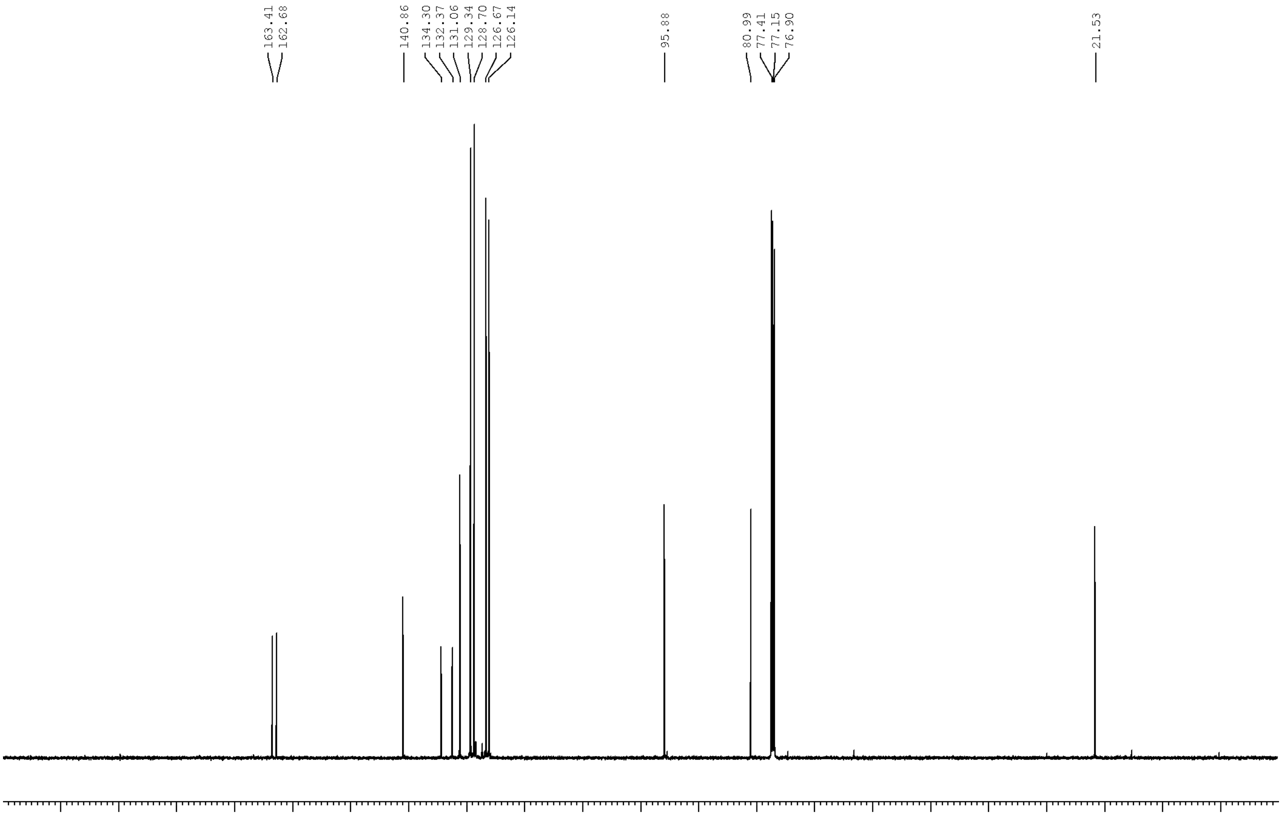

4-(4-Chlorophenyl)-6-phenyl-2*H*-1,3-oxazine (2c)

^{1}H NMR (400 MHz, $CDCl_3$)

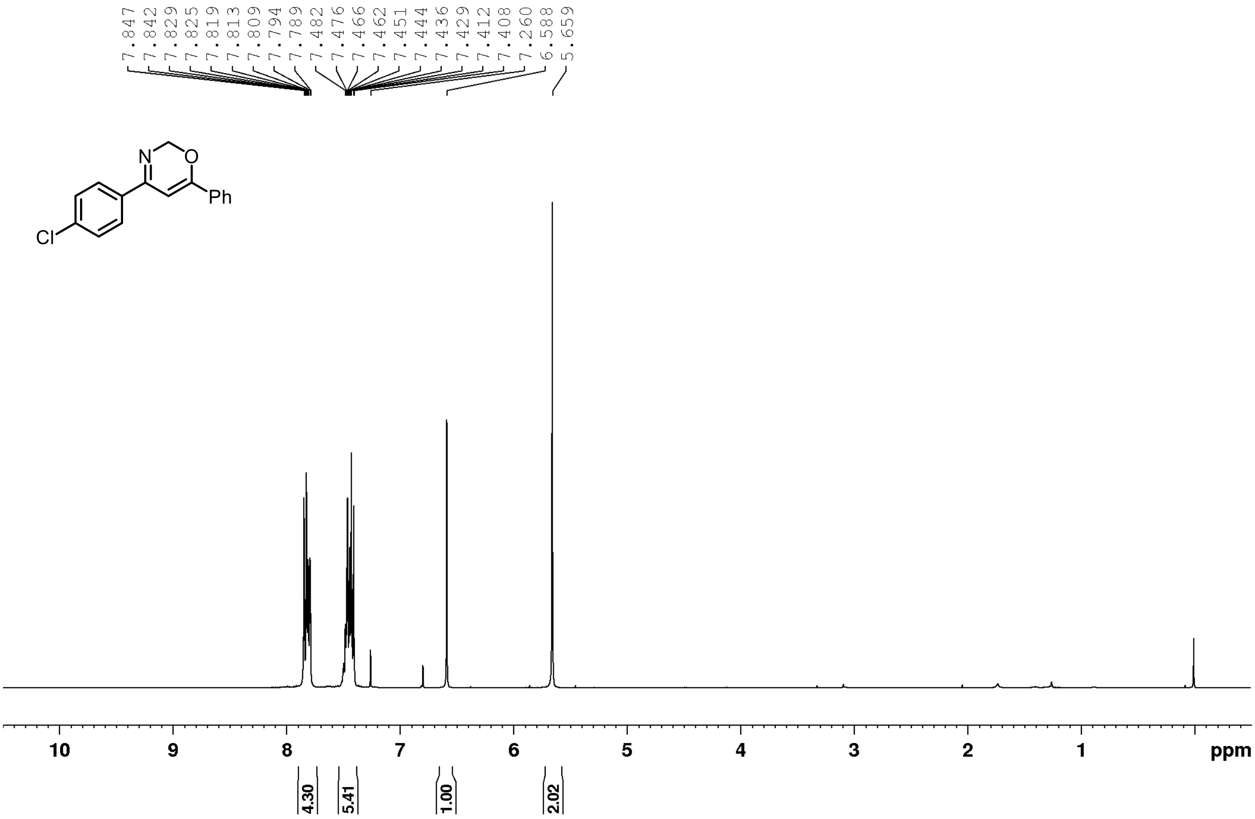

^{13}C NMR (100 MHz, $CDCl_3$)

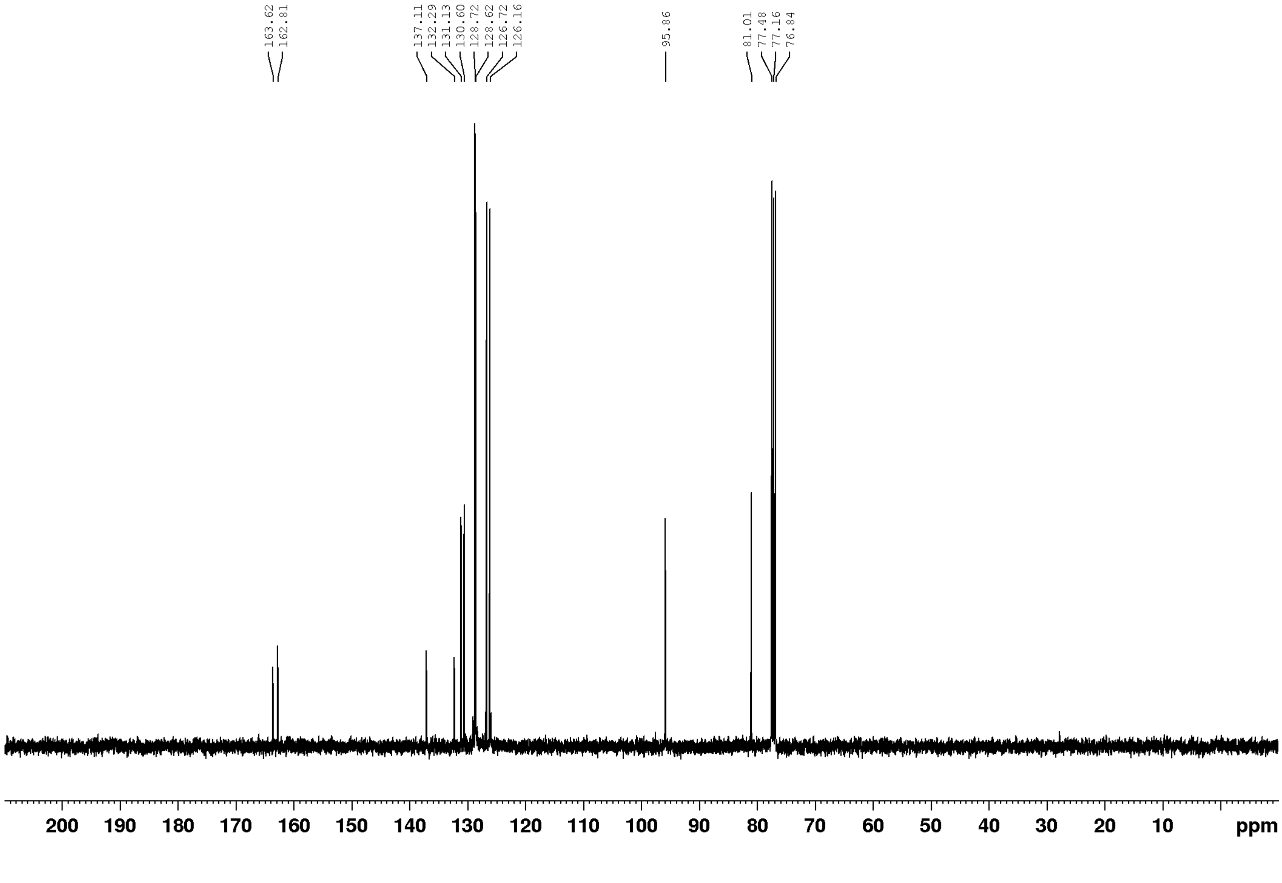

4-(3,4-Dichlorophenyl)-6-phenyl-2*H*-1,3-oxazine (2d)

^{1}H NMR (500 MHz, $CDCl_3$)

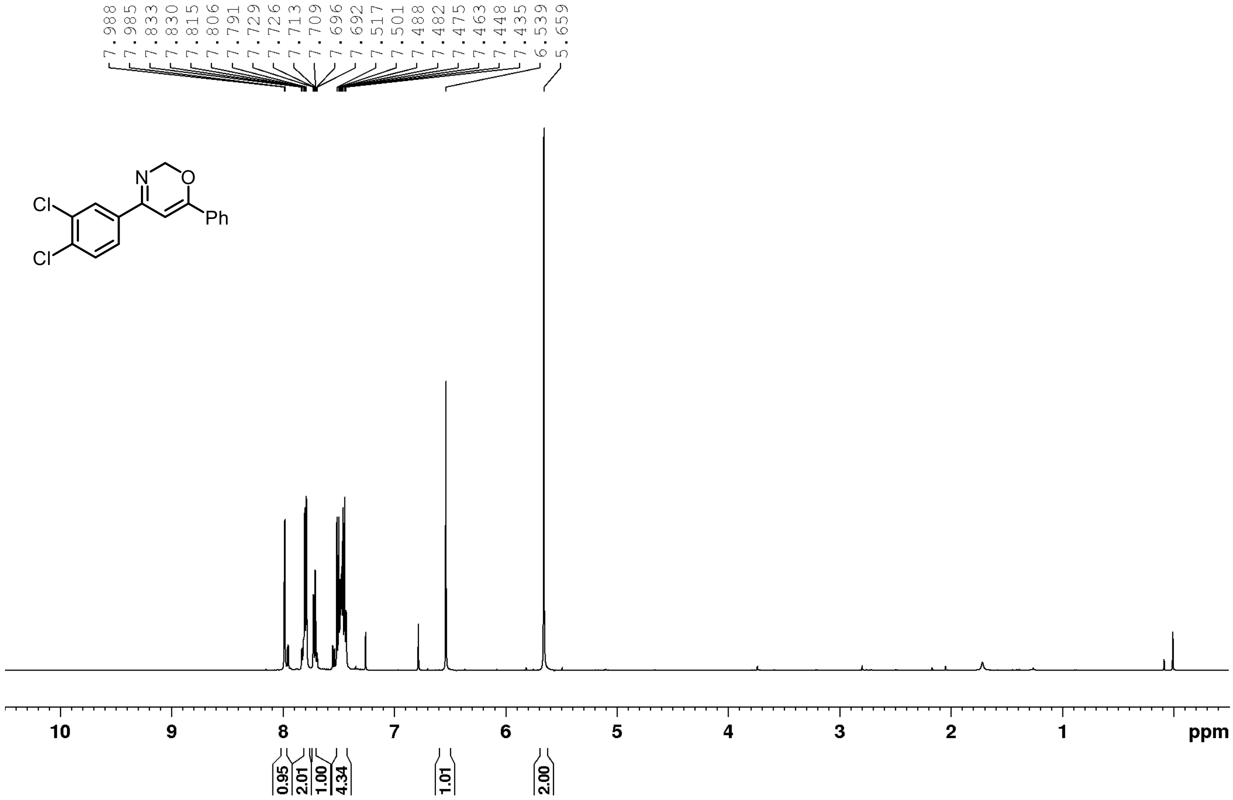

^{13}C NMR (125 MHz, $CDCl_3$)

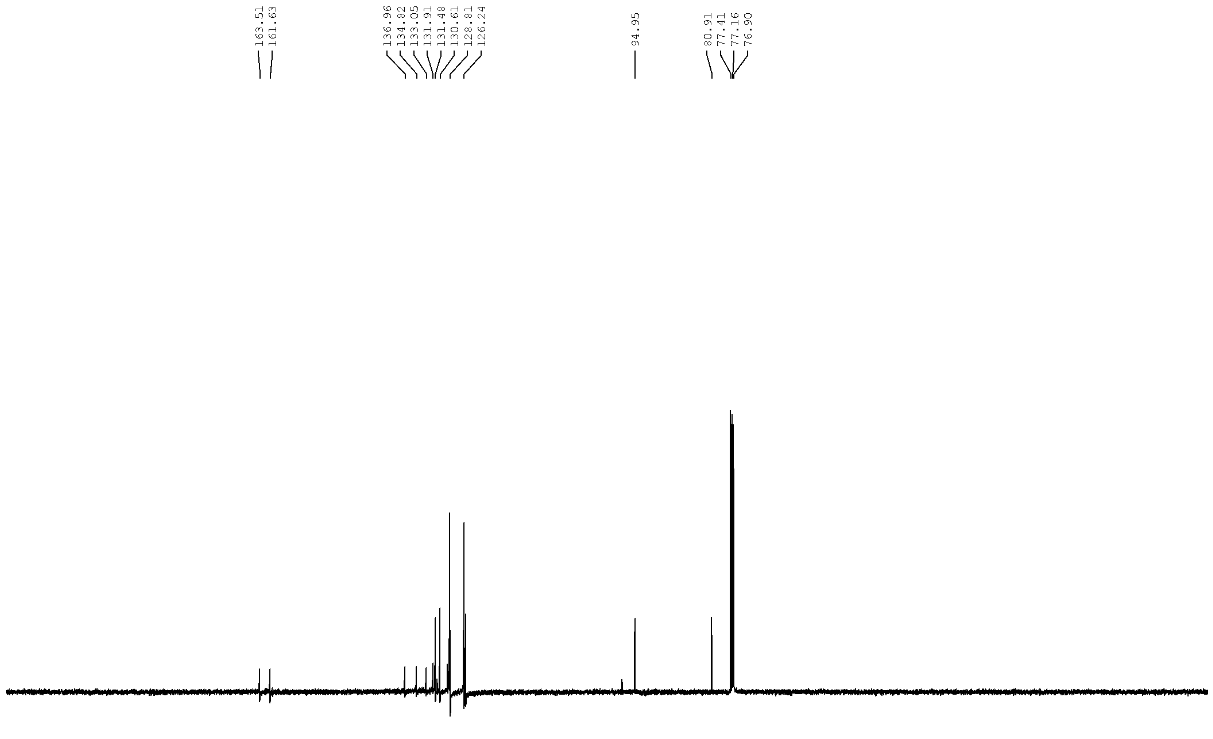

6-**Phenyl-4-(*o*-tolyl)-2*H*-1,3-oxazine (2e)**

^{1}H NMR (400 MHz, CDCl$_3$)

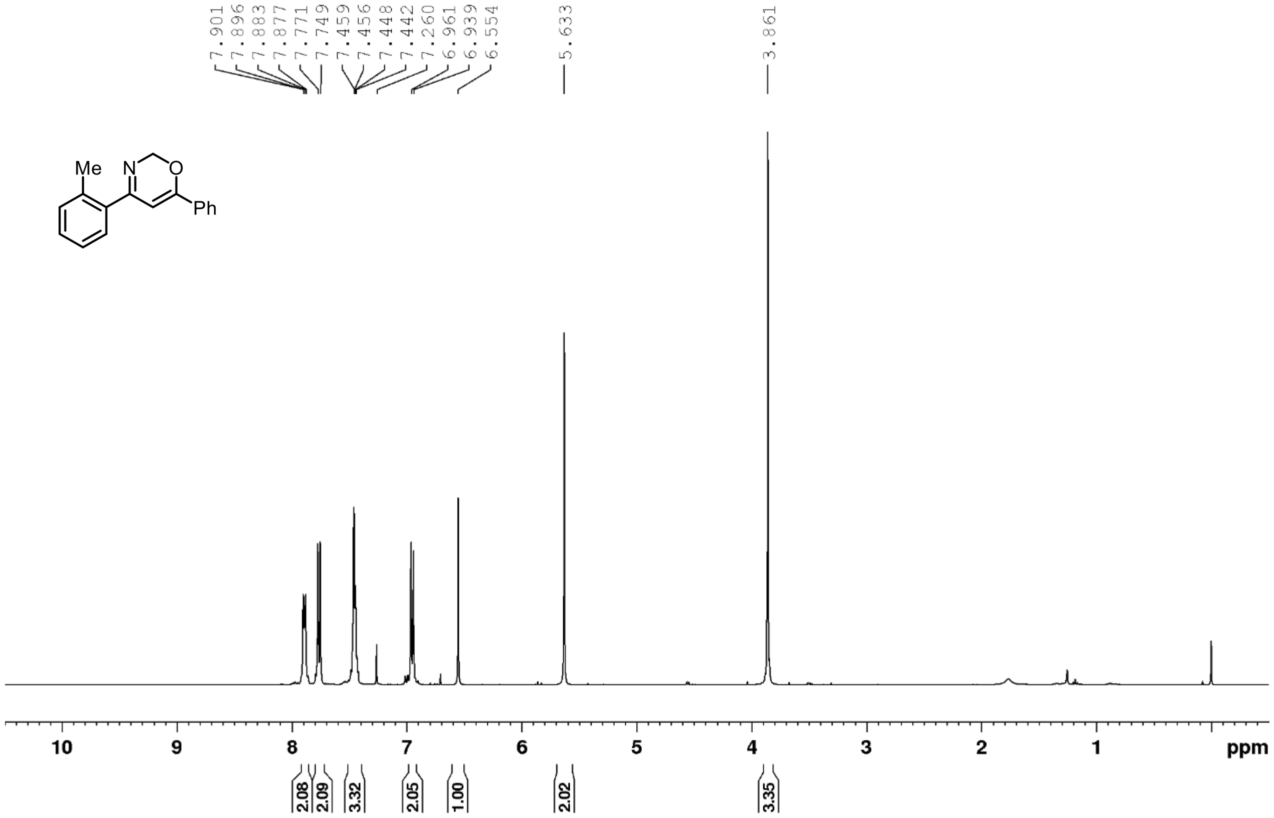

^{13}C NMR (100 MHz, CDCl$_3$)

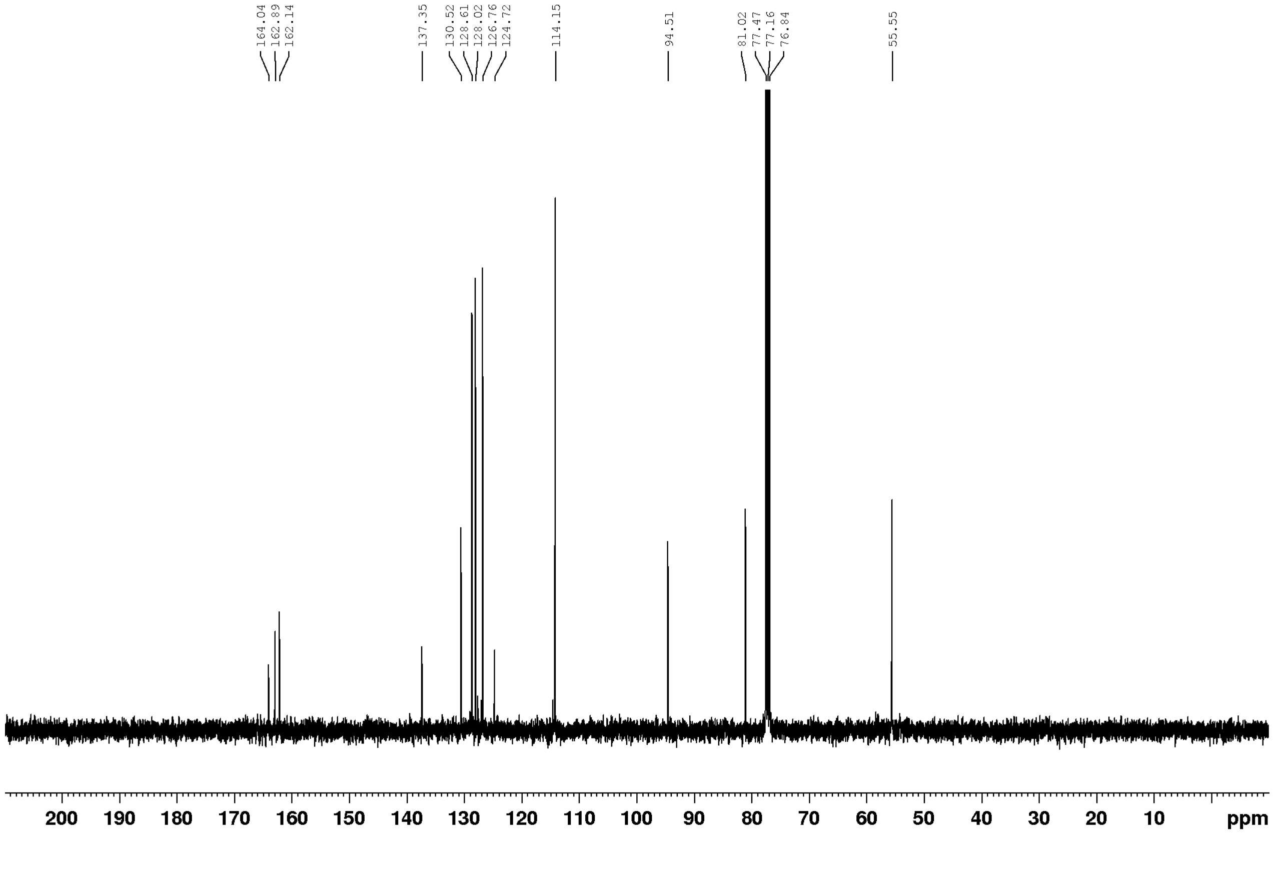

4-Cyclohexyl-6-phenyl-2*H*-1,3-oxazine (2f)

^{1}H NMR (500 MHz, $CDCl_3$)

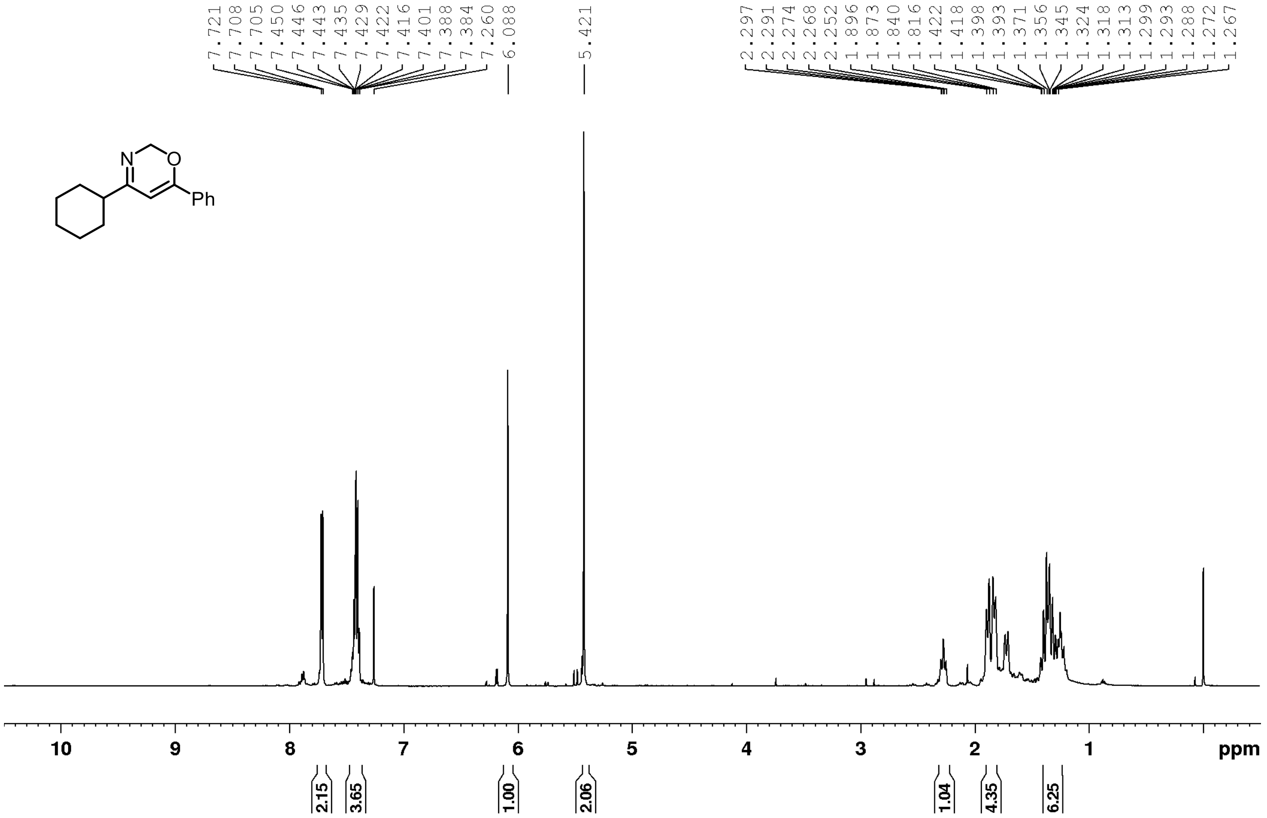

^{13}C NMR (125 MHz, $CDCl_3$)

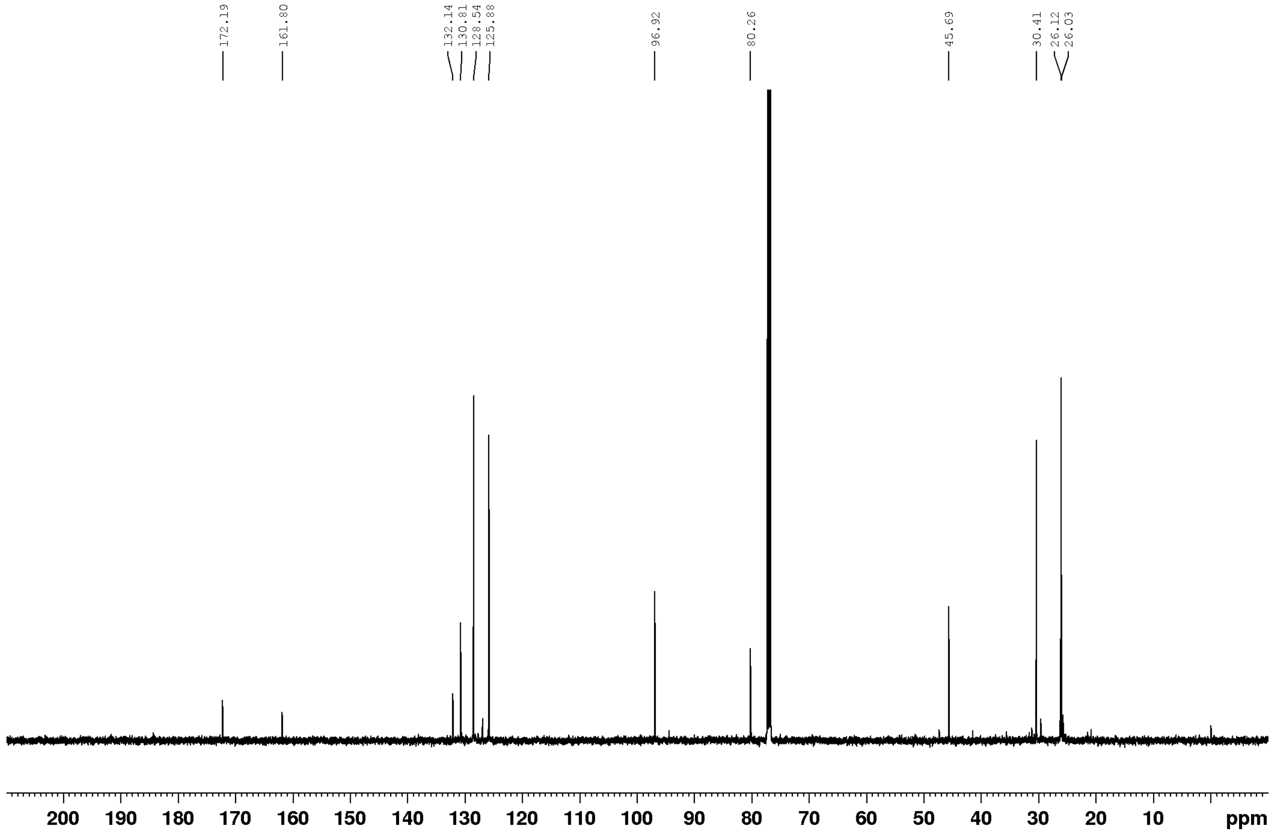

4-(*tert*-Butyl)-6-phenyl-2*H*-1,3-oxazine (2g)

^{1}H NMR (500 MHz, $CDCl_3$)

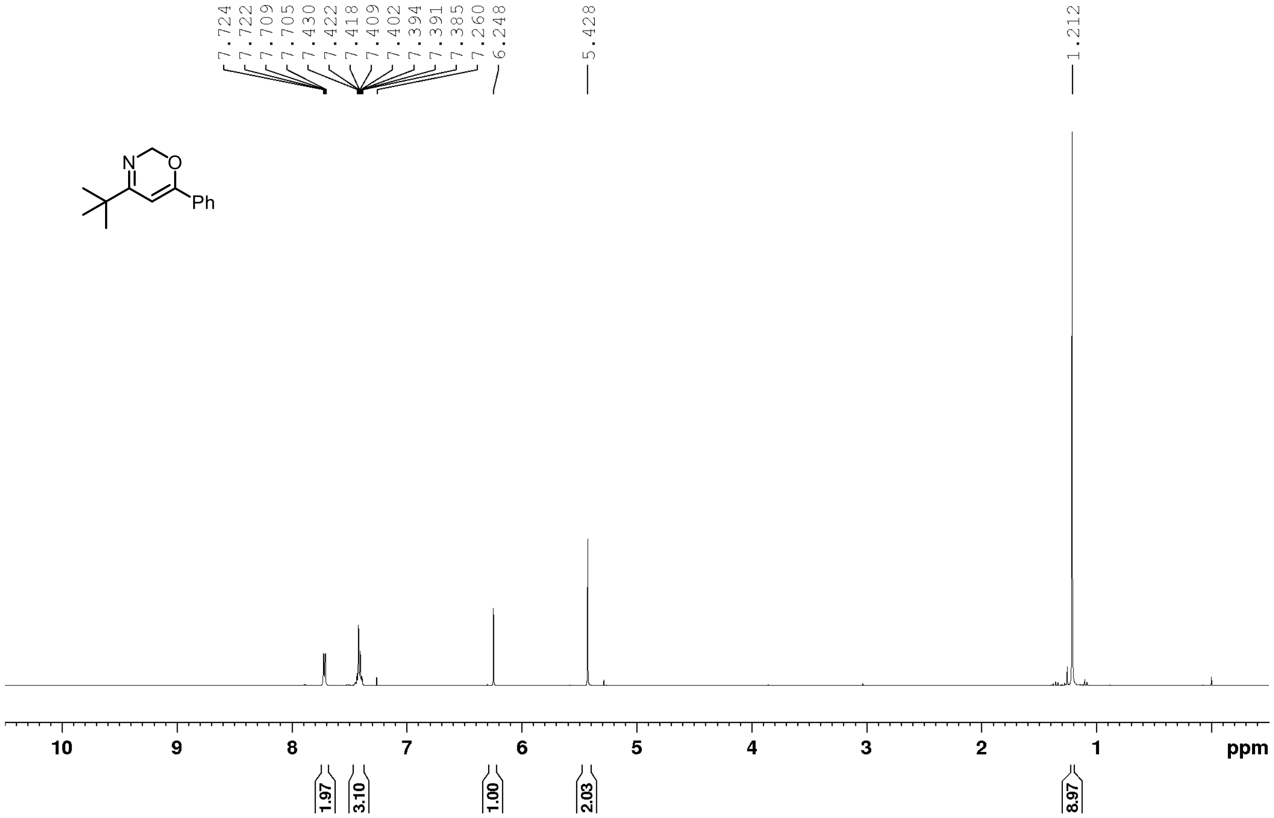

^{13}C NMR (125 MHz, $CDCl_3$)

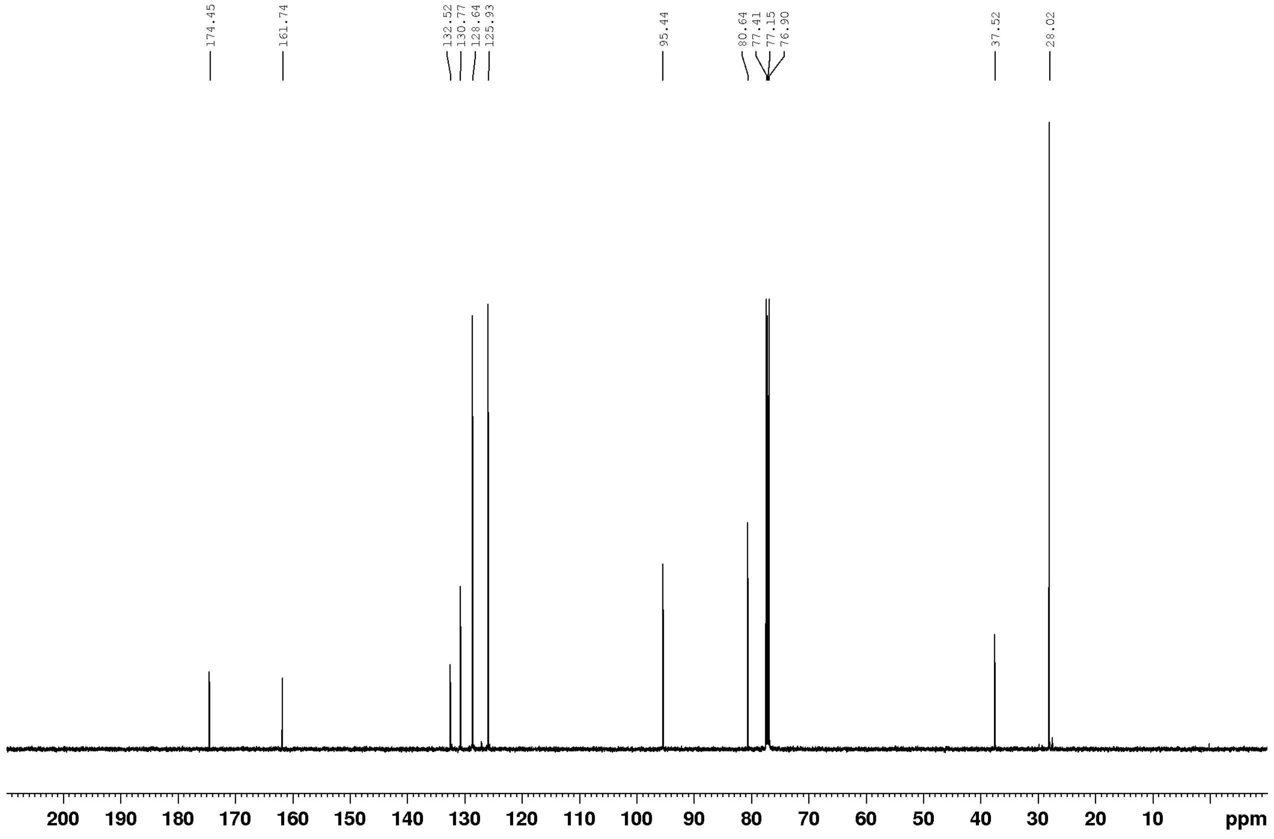

Ethyl 6-phenyl-2*H*-1,3-oxazine-4-carboxylate (2h)

^{1}H NMR (500 MHz, $CDCl_3$)

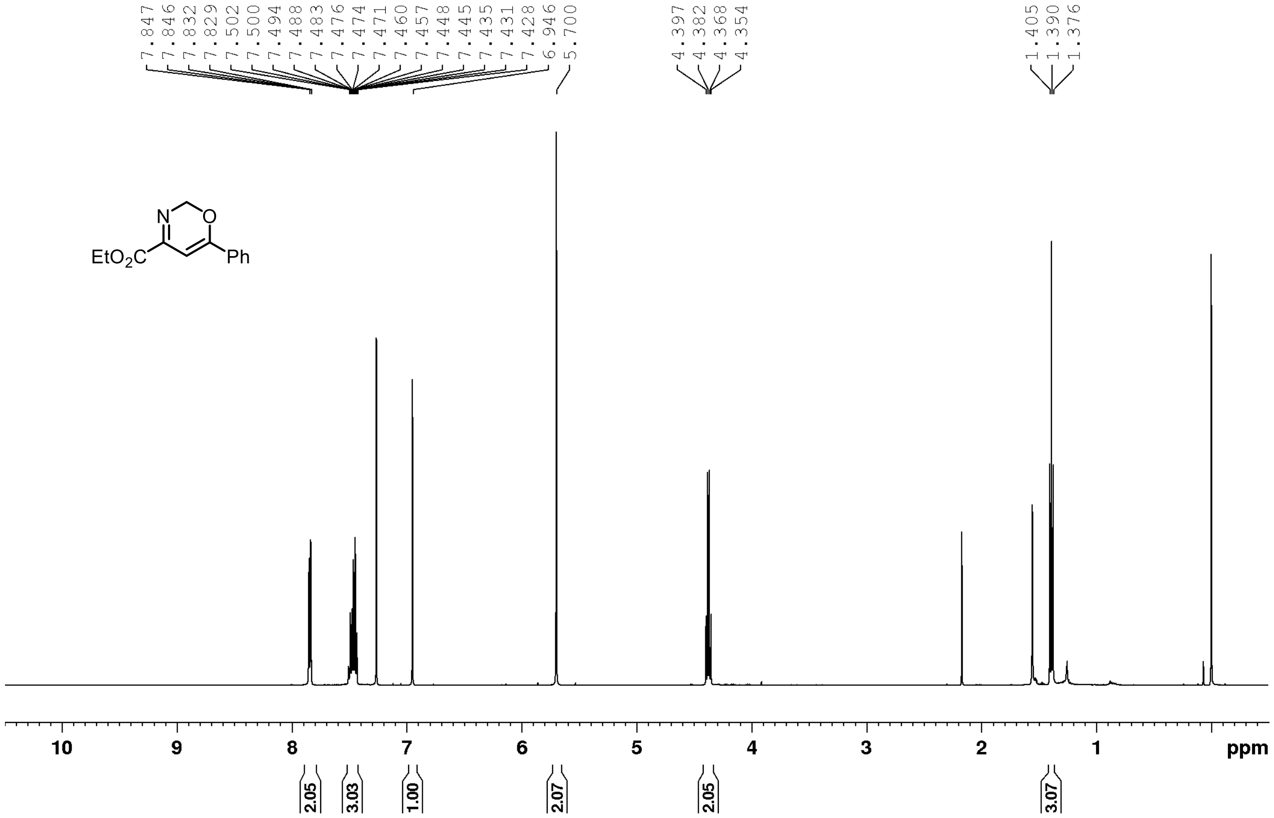

^{13}C NMR (125 MHz, $CDCl_3$)

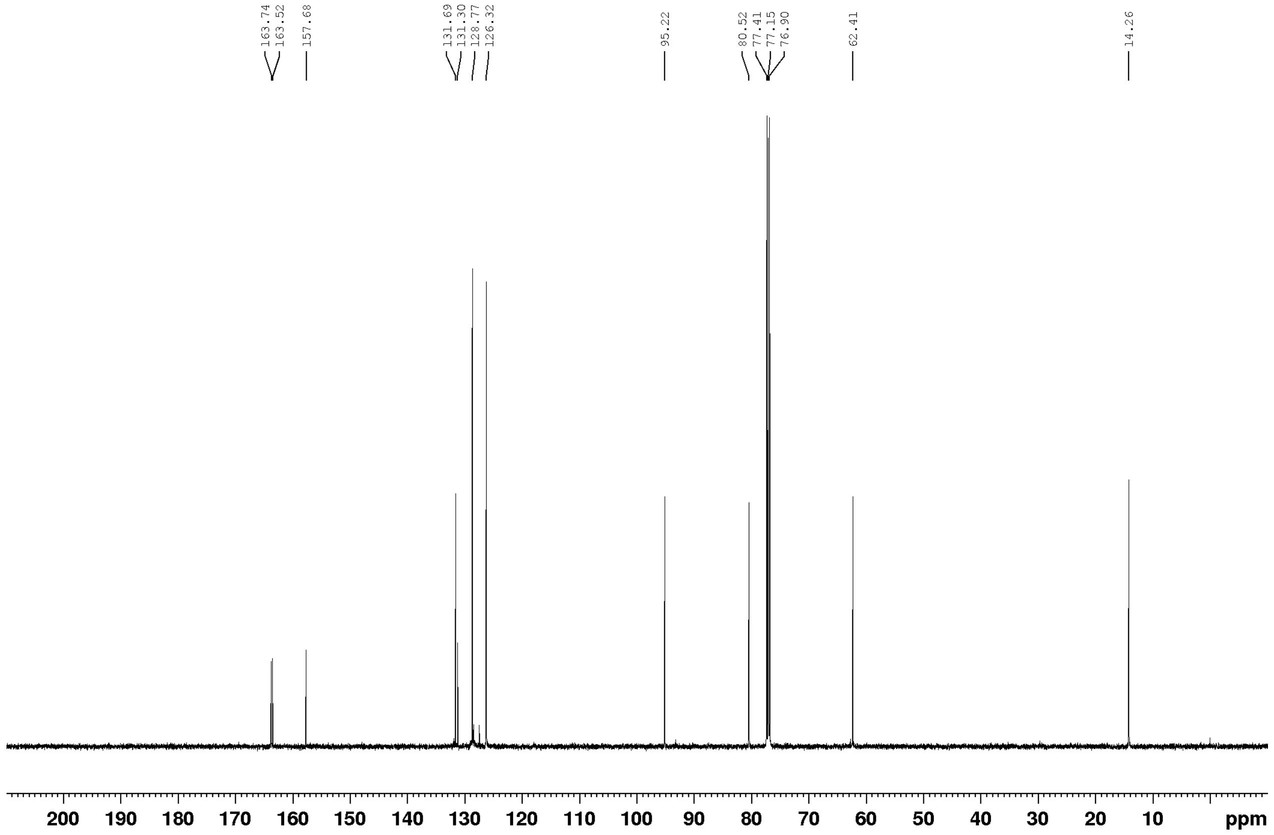

4-Phenyl-6-(*p*-tolyl)-2*H*-1,3-oxazine (2i)

^{1}H NMR (500 MHz, $CDCl_3$)

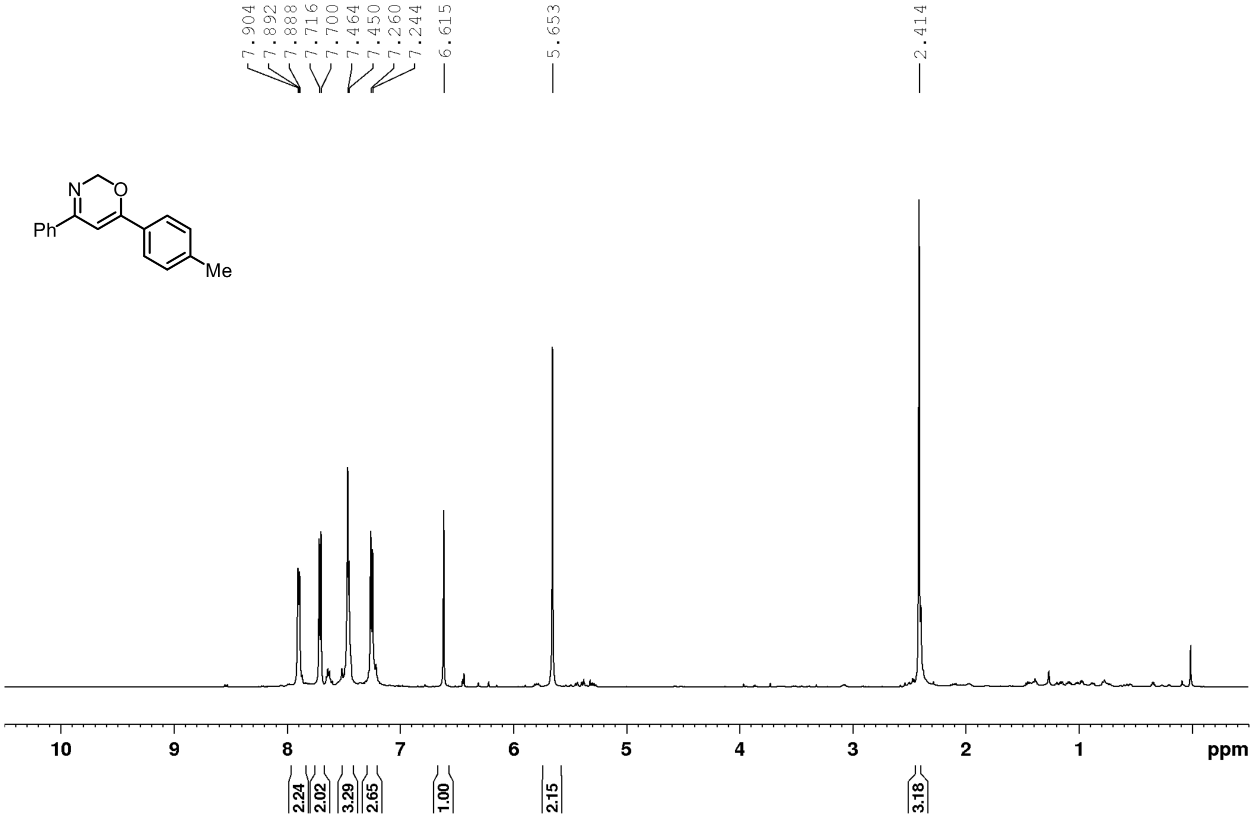

^{13}C NMR (125 MHz, $CDCl_3$)

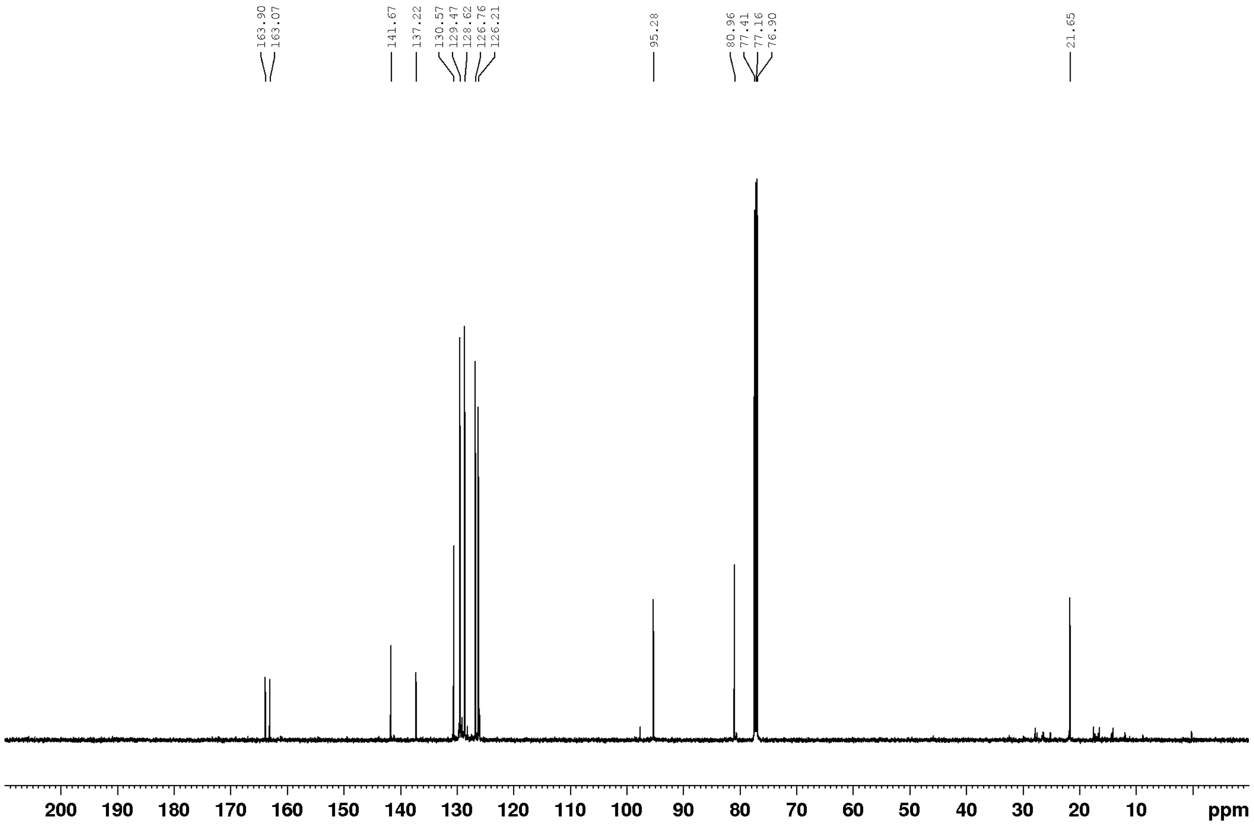

6-(4-Methoxyphenyl)-4-phenyl-2*H*-1,3-oxazine (2j)

^{1}H NMR (400 MHz, $CDCl_3$)

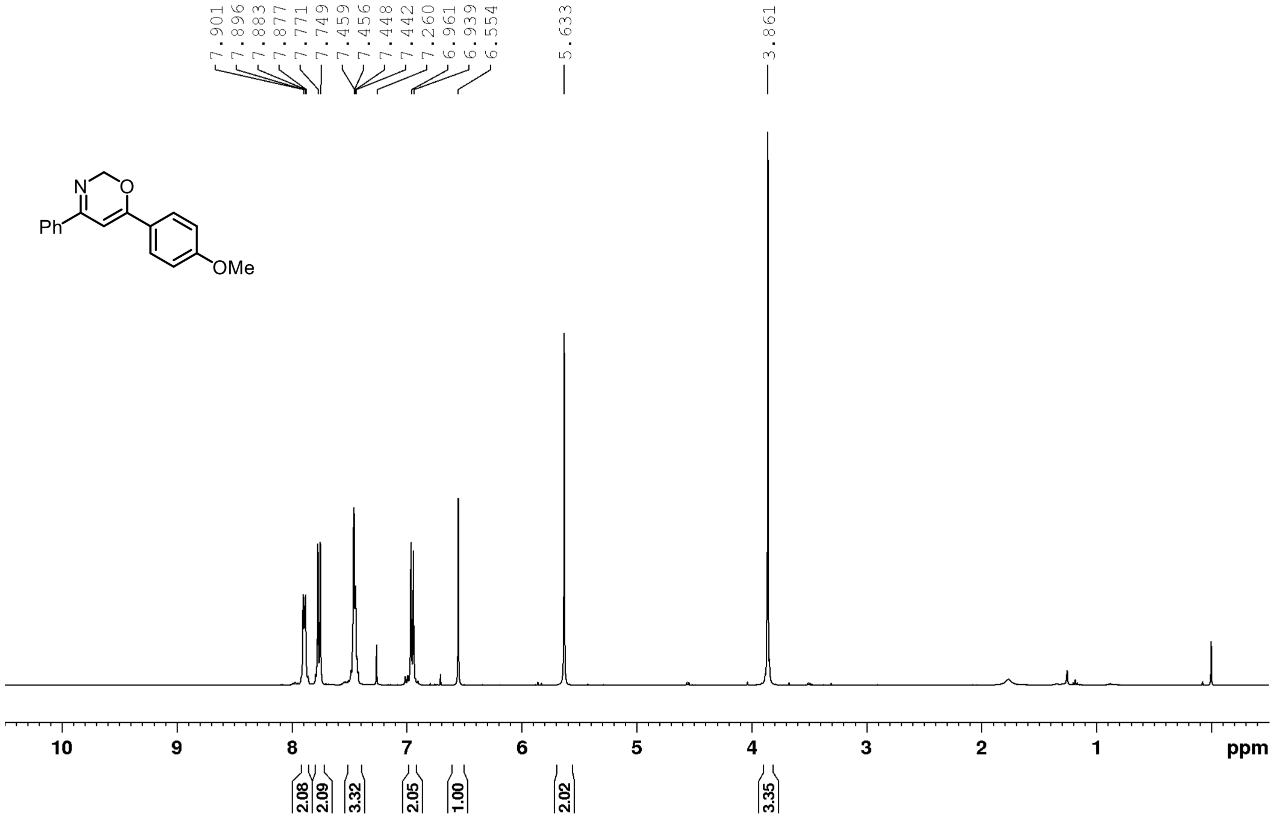

^{13}C NMR (100 MHz, $CDCl_3$)

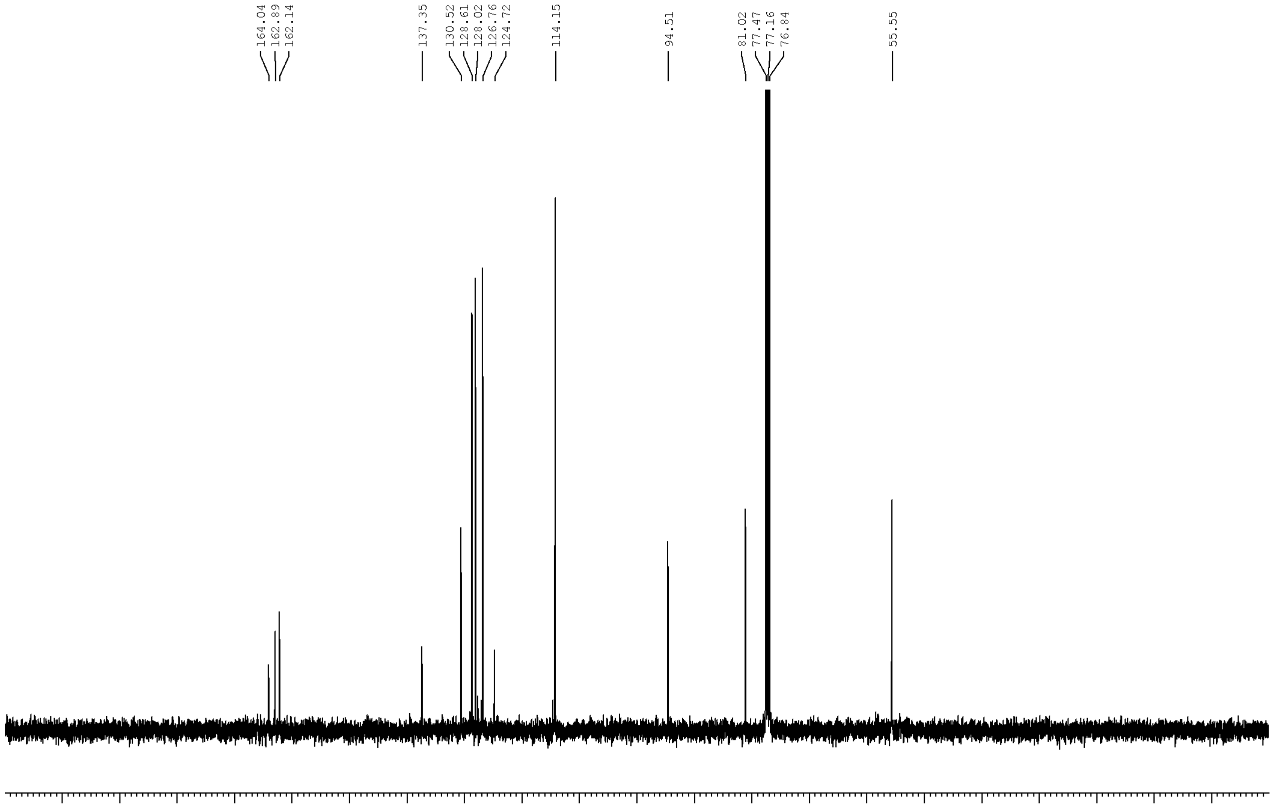

6-(4-Fluorophenyl)-4-phenyl-2*H*-1,3-oxazine (2k)

^{1}H NMR (500 MHz, CDCl$_3$)

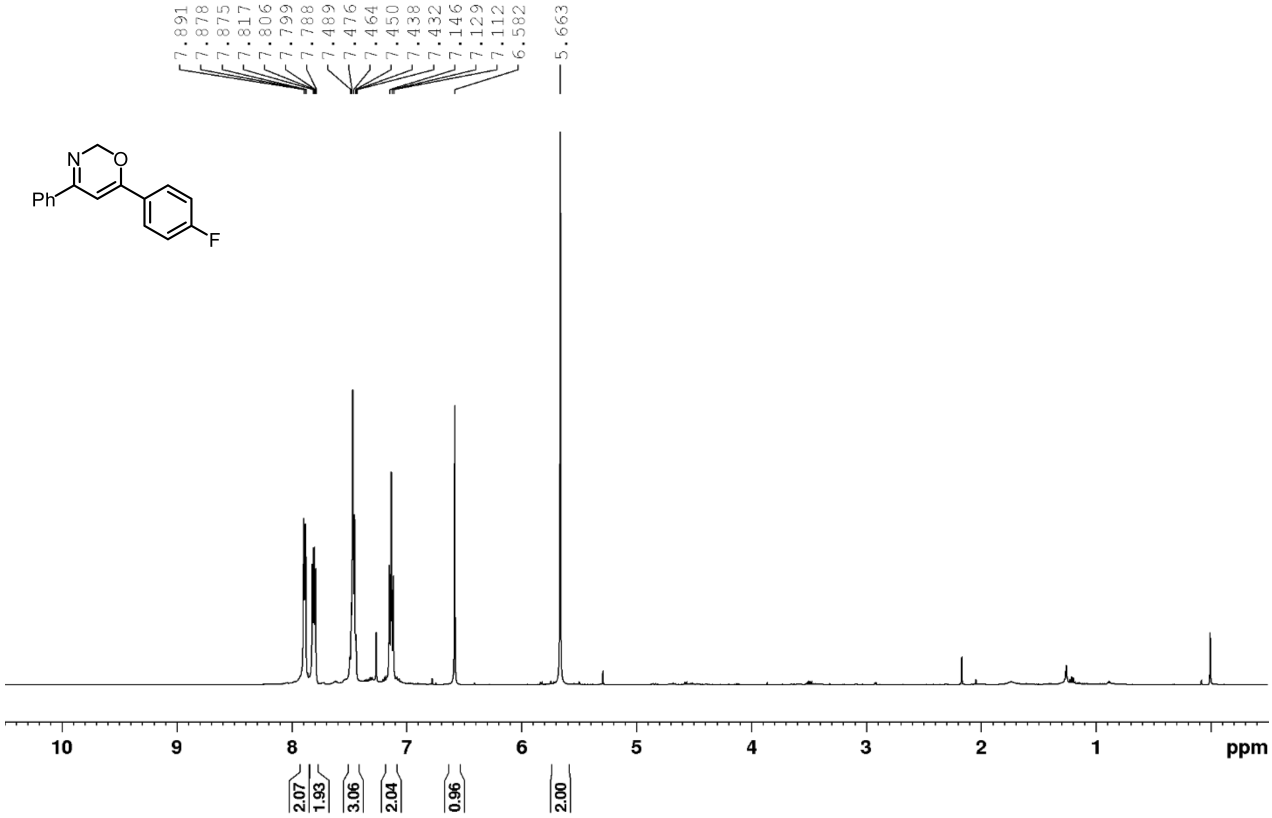

^{13}C NMR (125 MHz, CDCl$_3$)

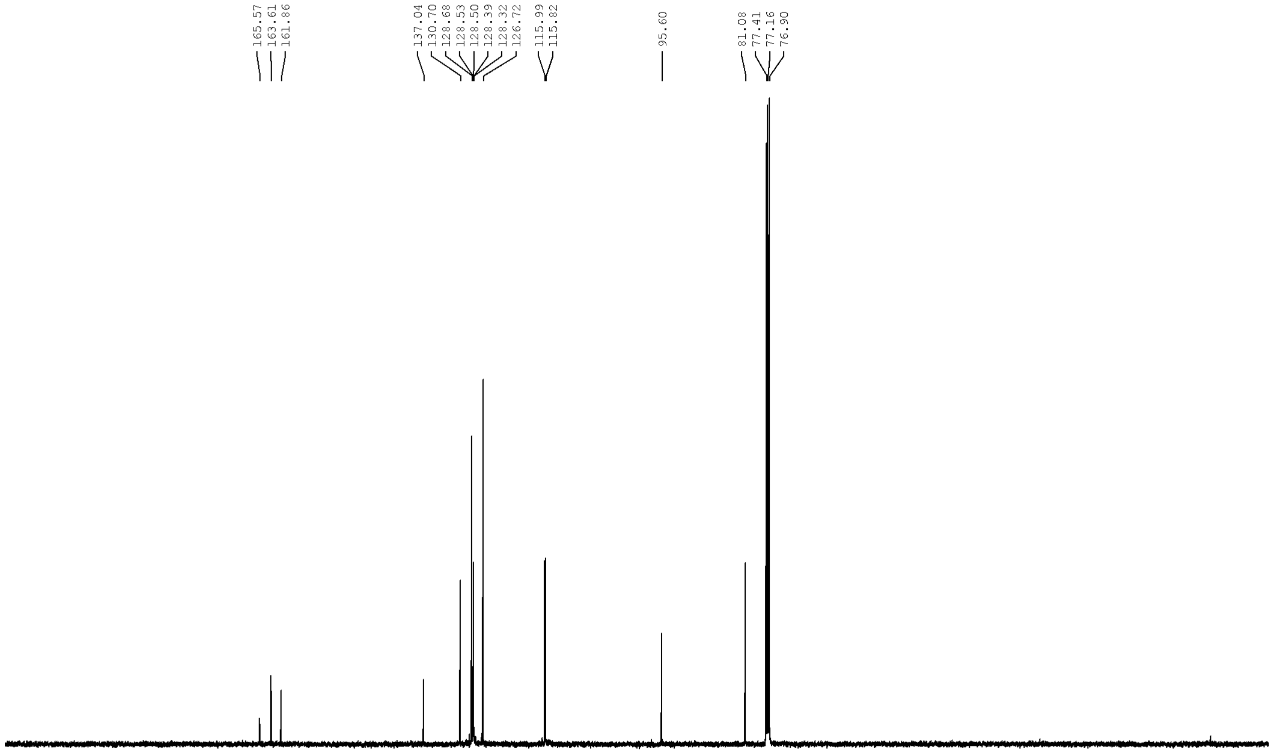

4-Phenyl-6-(*o*-tolyl)-2*H*-1,3-oxazine (2l)

^{1}H NMR (500 MHz, $CDCl_3$)

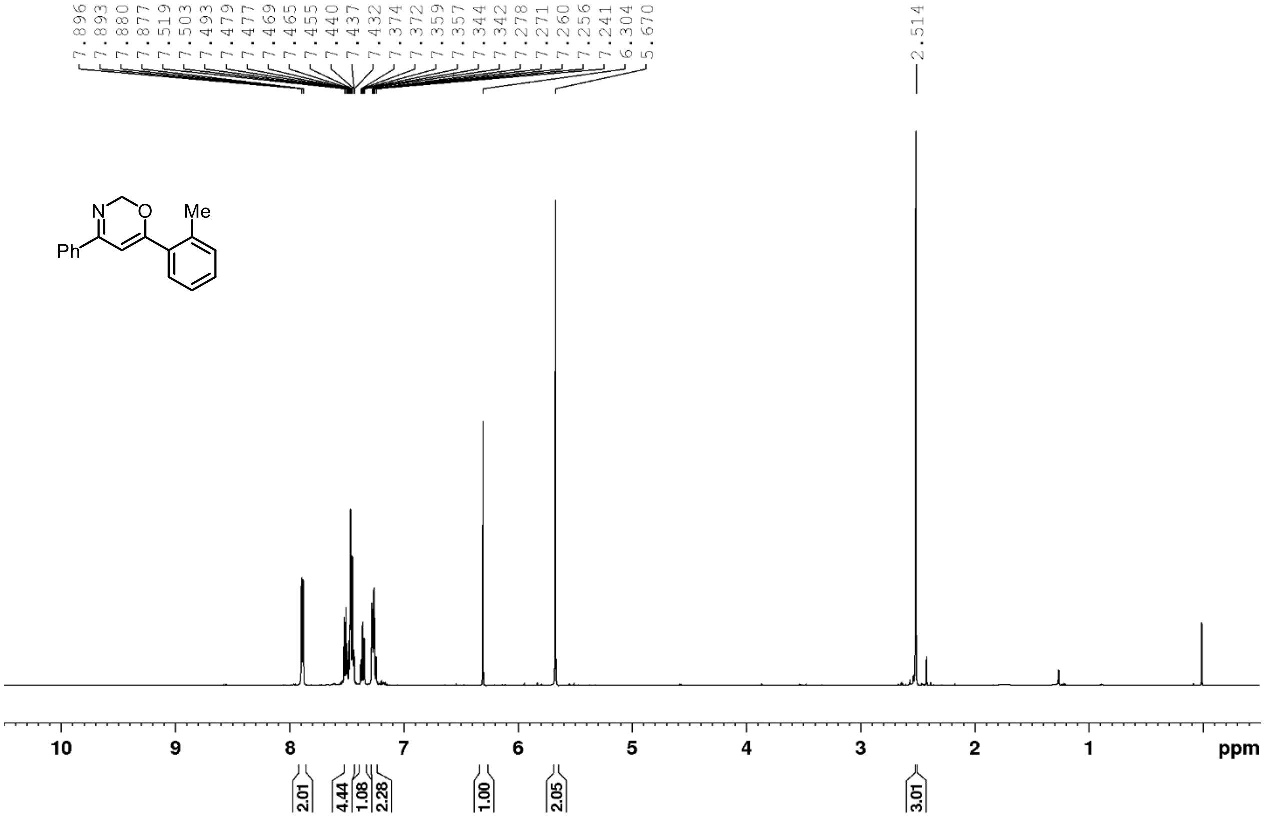

^{13}C NMR (125 MHz, $CDCl_3$)

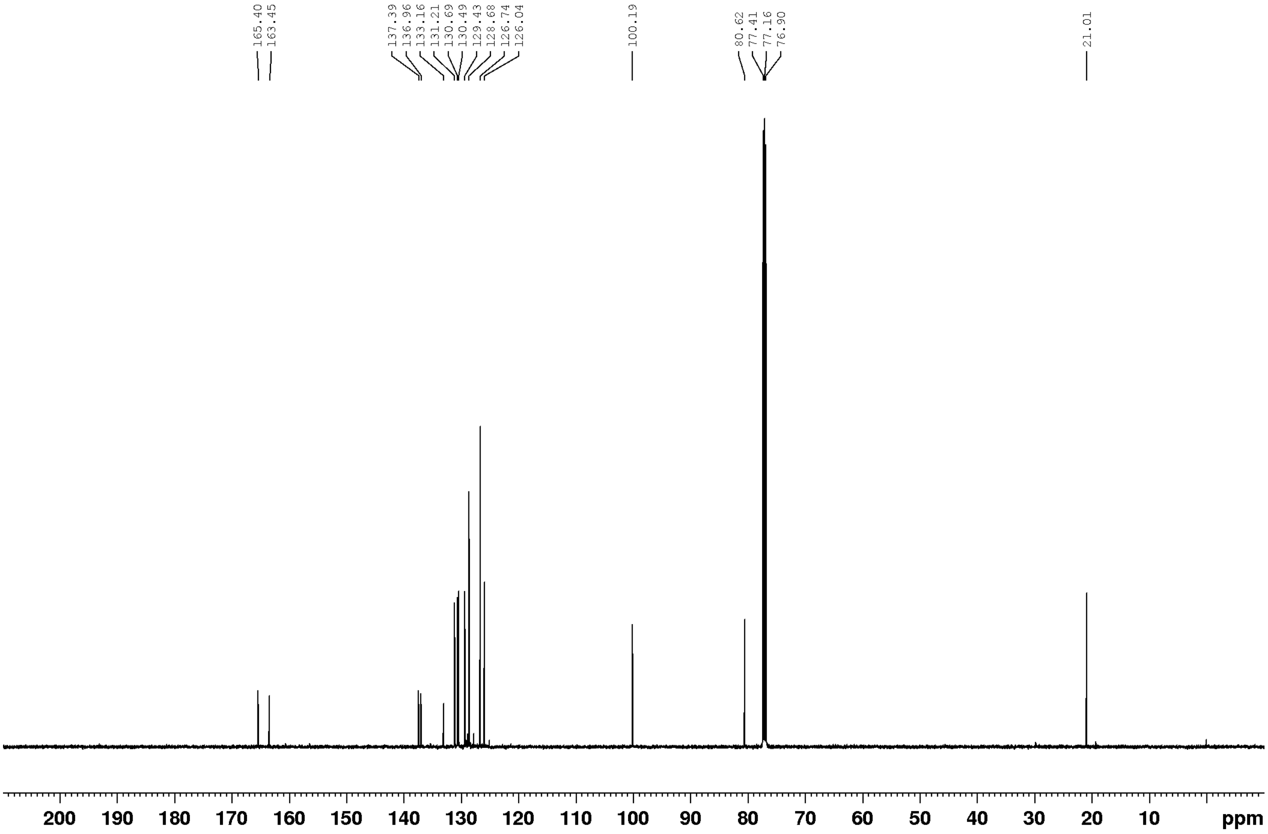

6-(*tert*-Butyl)-4-phenyl-2*H*-1,3-oxazine (2m)

^{1}H NMR (500 MHz, CDCl$_3$)

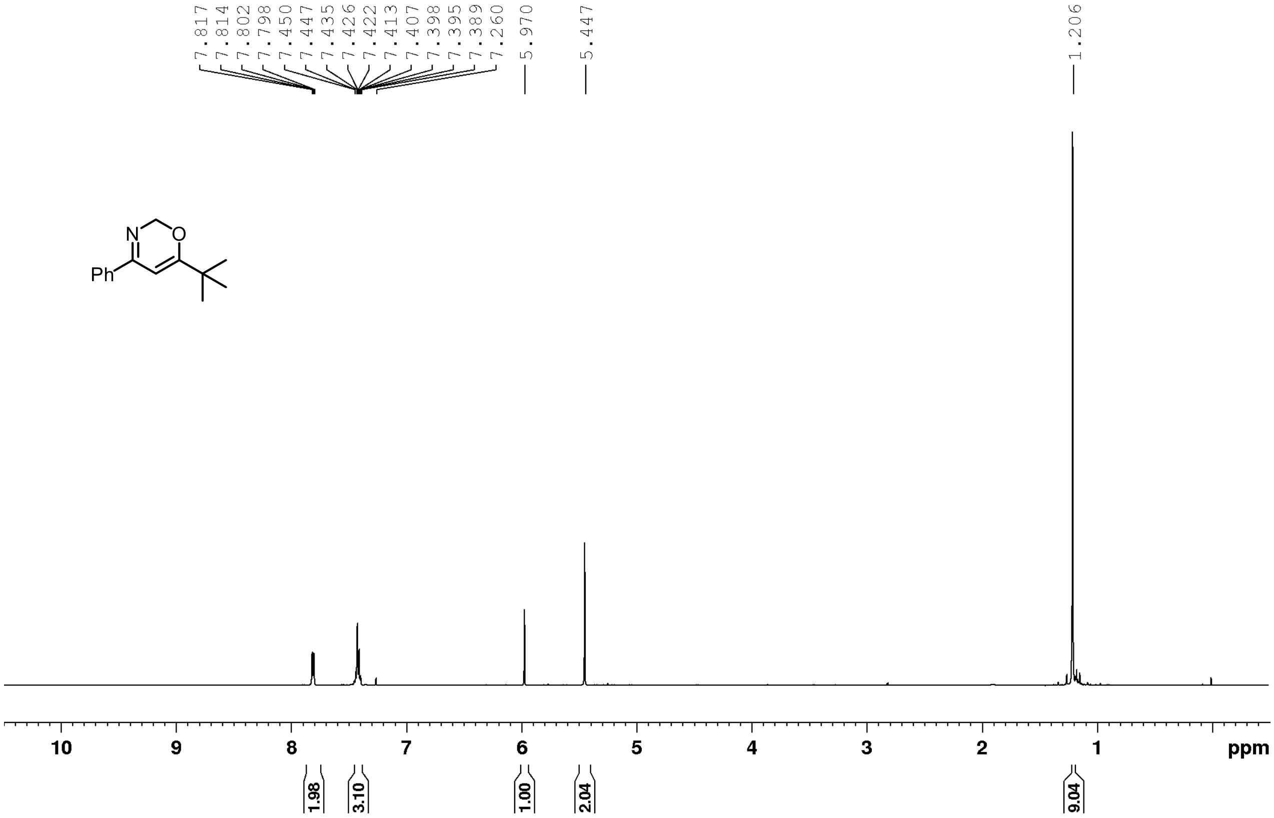

^{13}C NMR (125 MHz, CDCl$_3$)

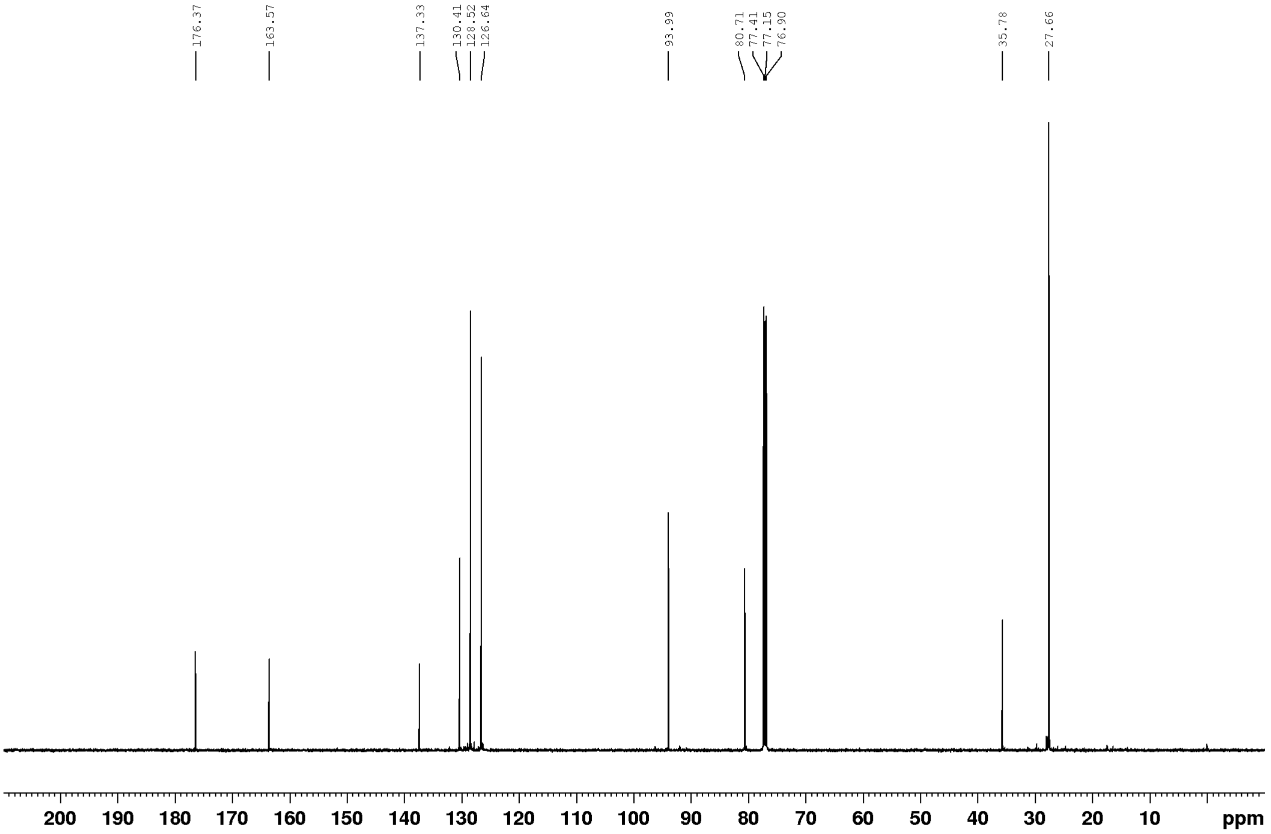

Ethyl 4-phenyl-2*H*-1,3-oxazine-6-carboxylate (2n)

^{1}H NMR (500 MHz, CDCl$_3$)

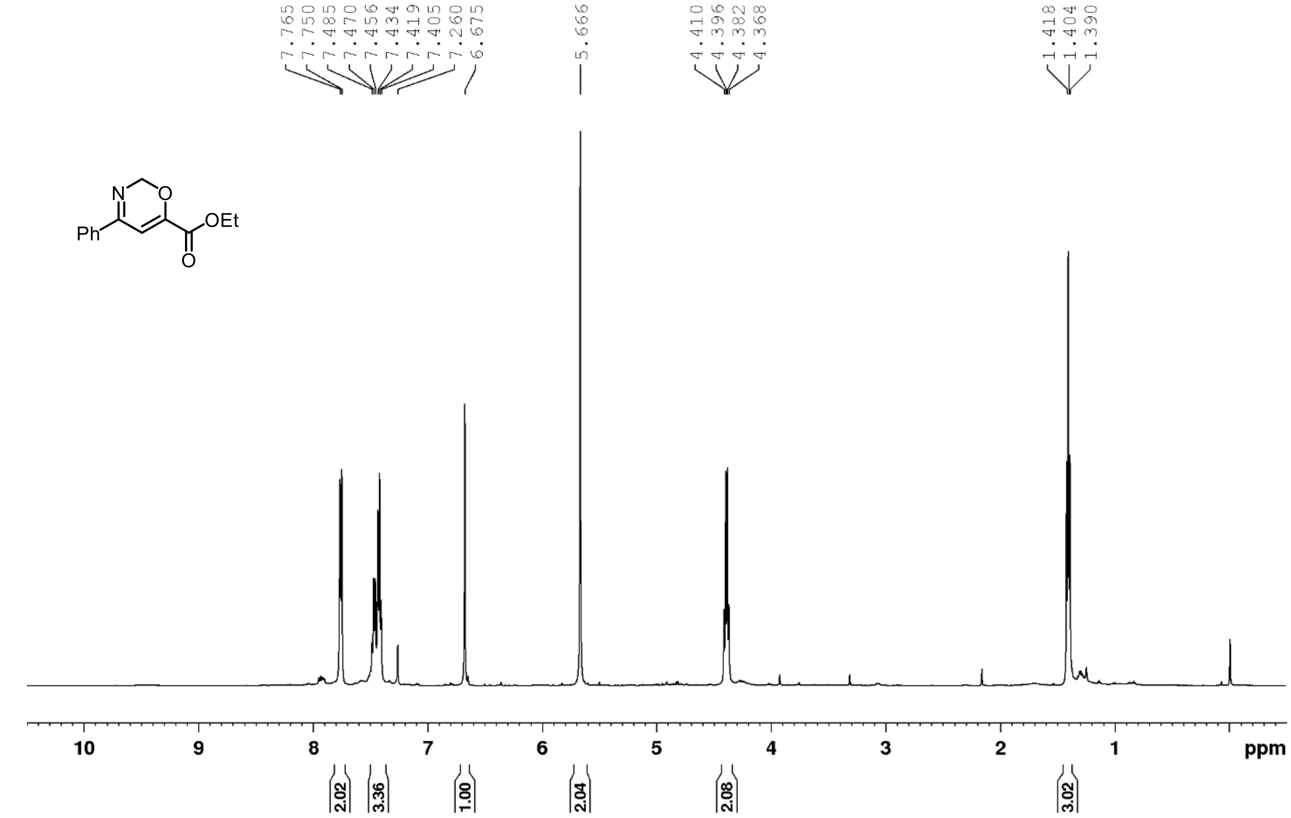

^{13}C NMR (125 MHz, CDCl$_3$)

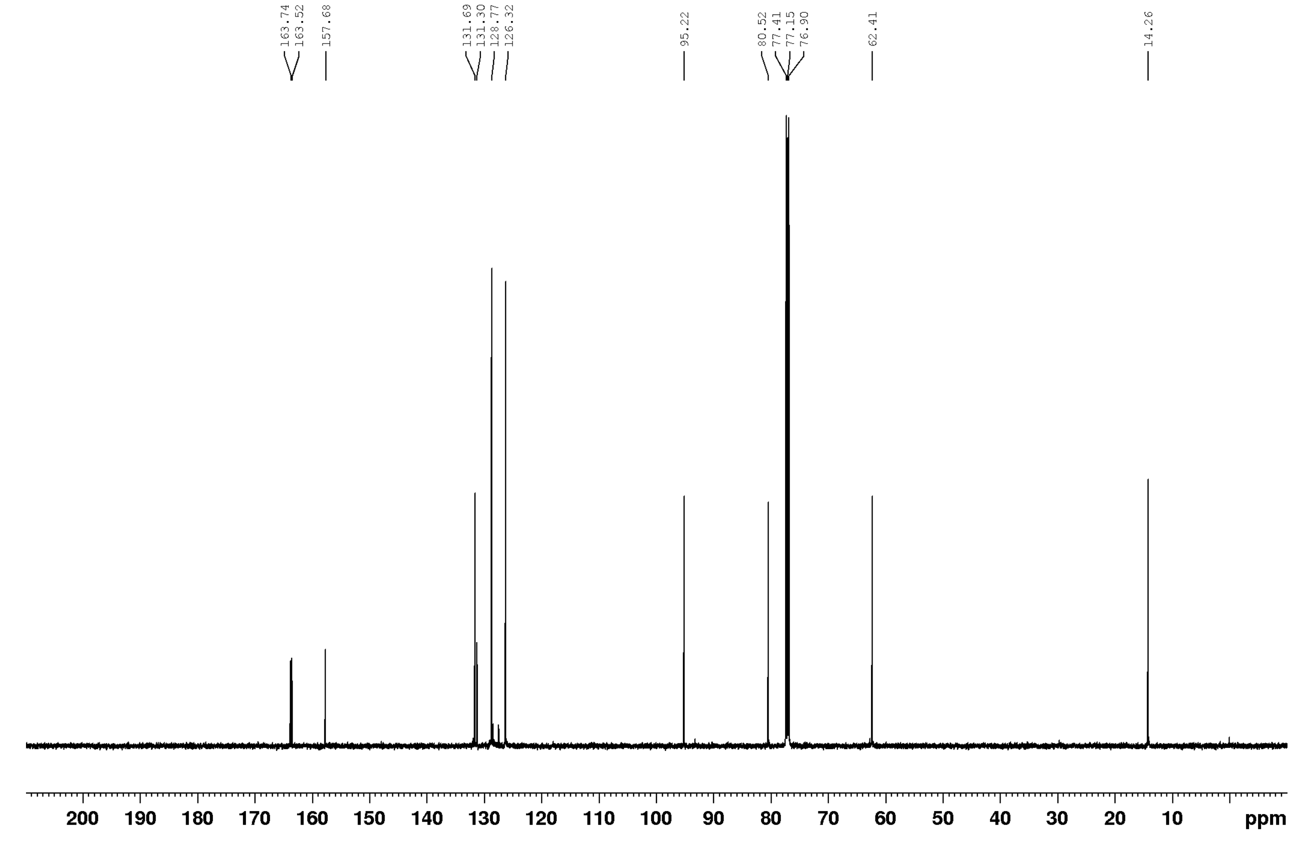

4-Phenyl-6-(trimethylsilyl)-2*H*-1,3-oxazine (2o)

^{1}H NMR (400 MHz, CDCl$_3$)

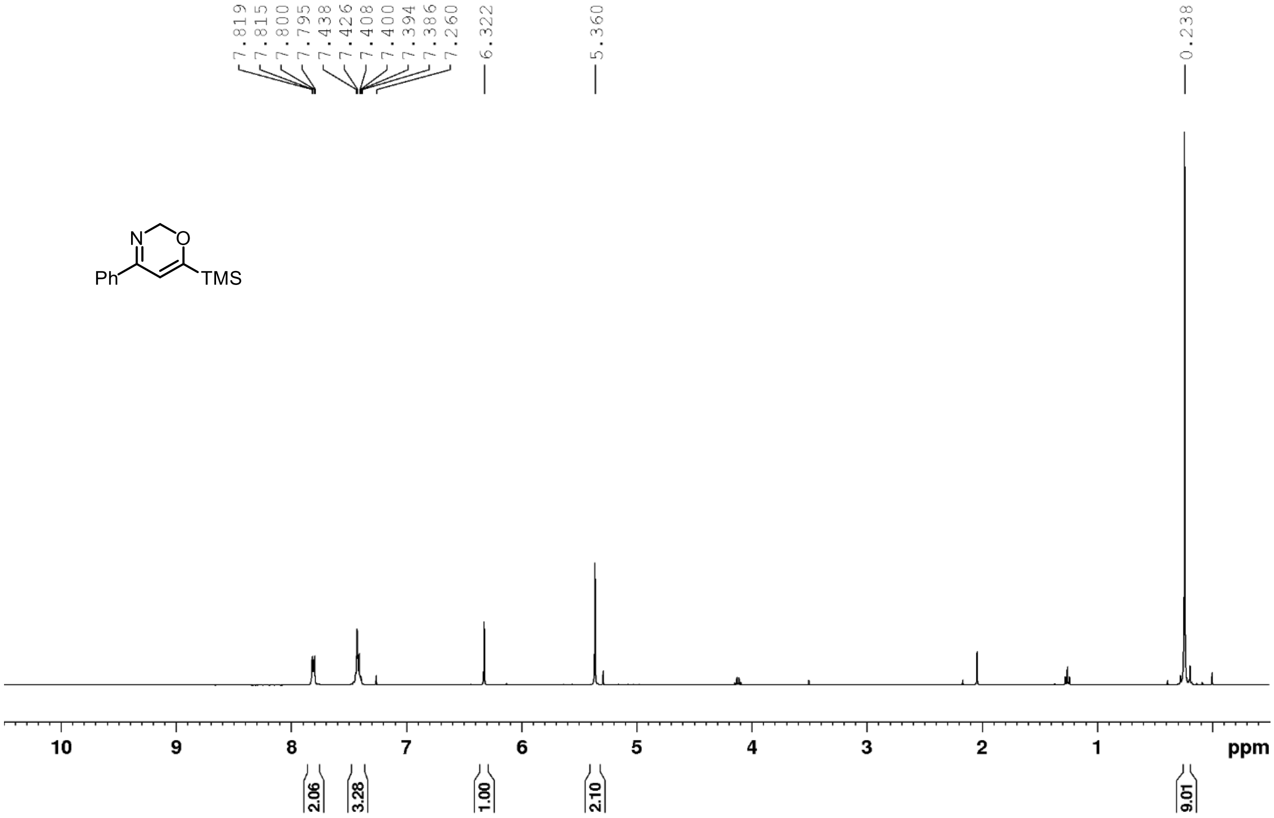

^{13}C NMR (125 MHz, CDCl$_3$)

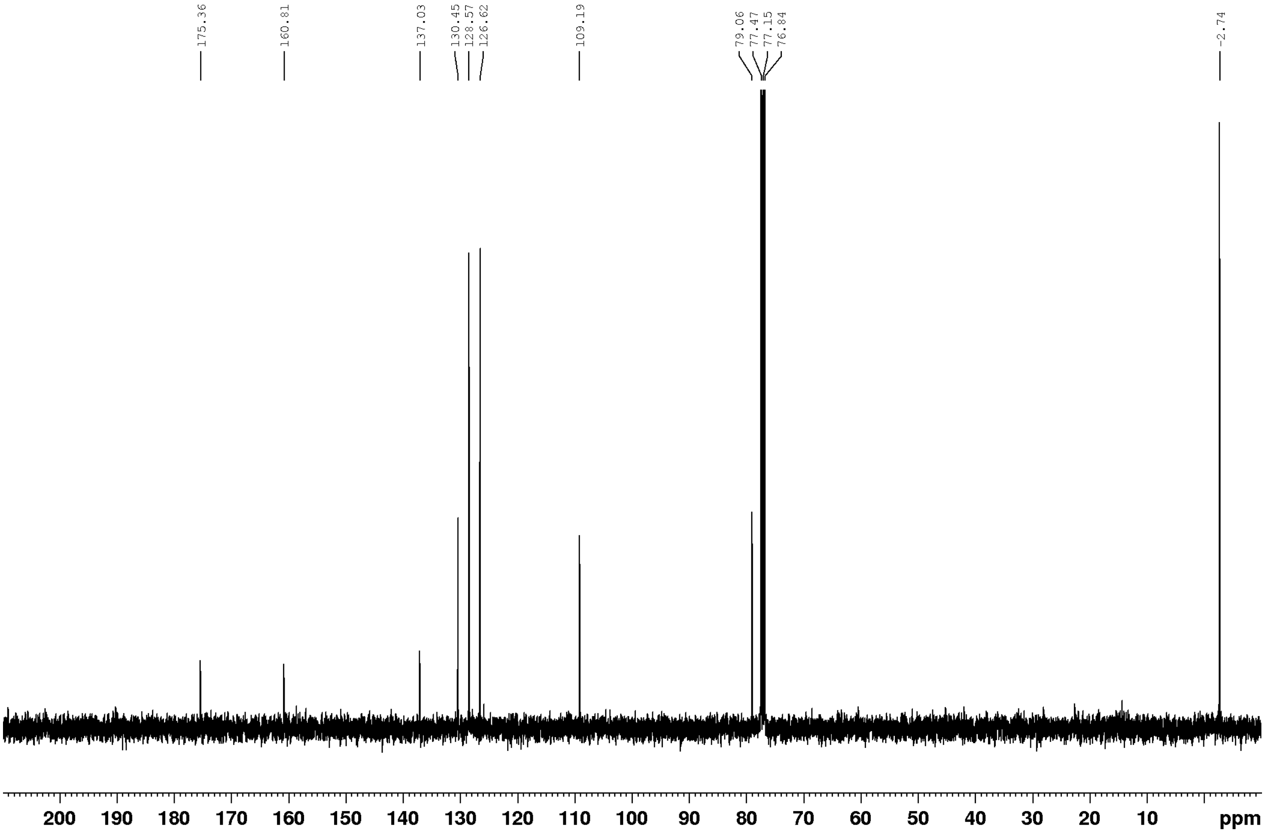

4-Phenyl-6-(triisopropylsilyl)-2*H*-1,3-oxazine (2p)

^{1}H NMR (500 MHz, $CDCl_3$)

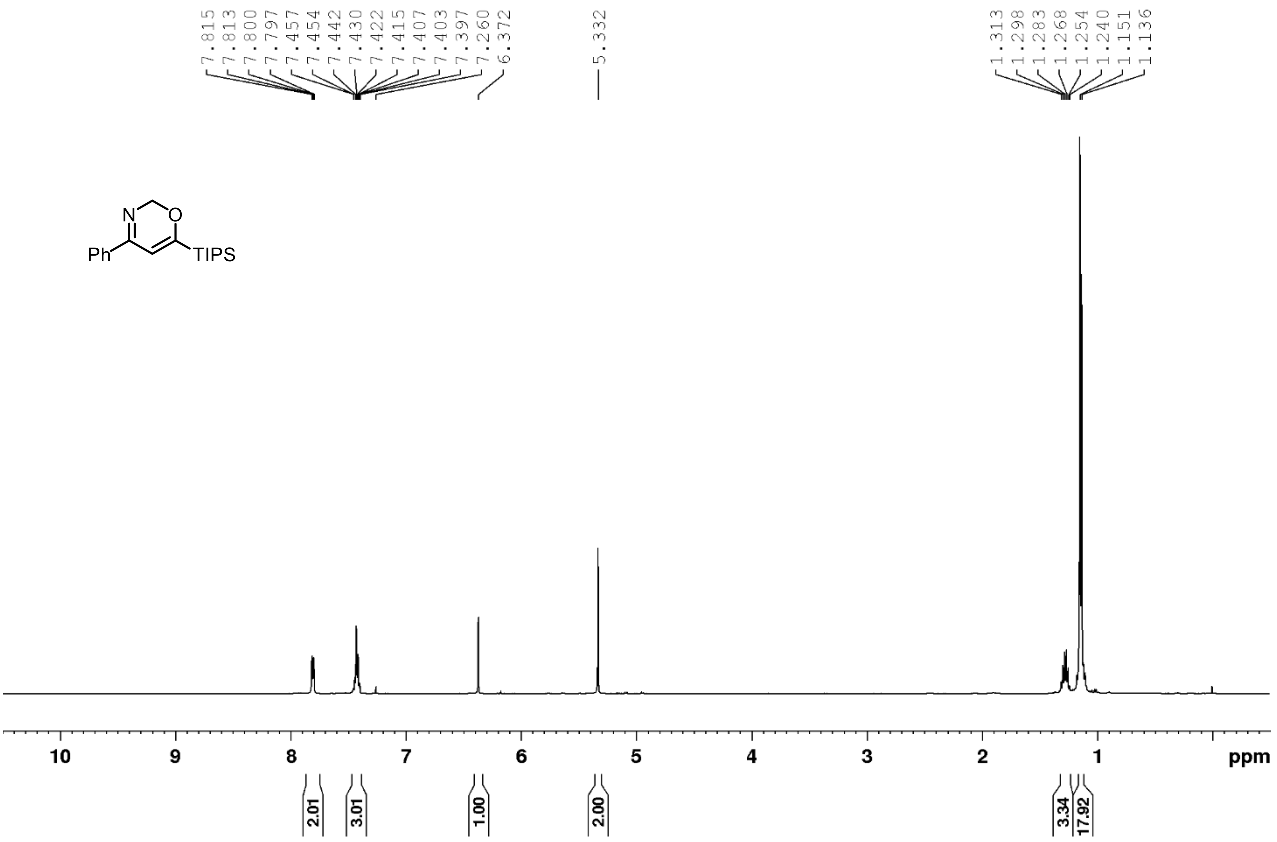

^{13}C NMR (125 MHz, $CDCl_3$)

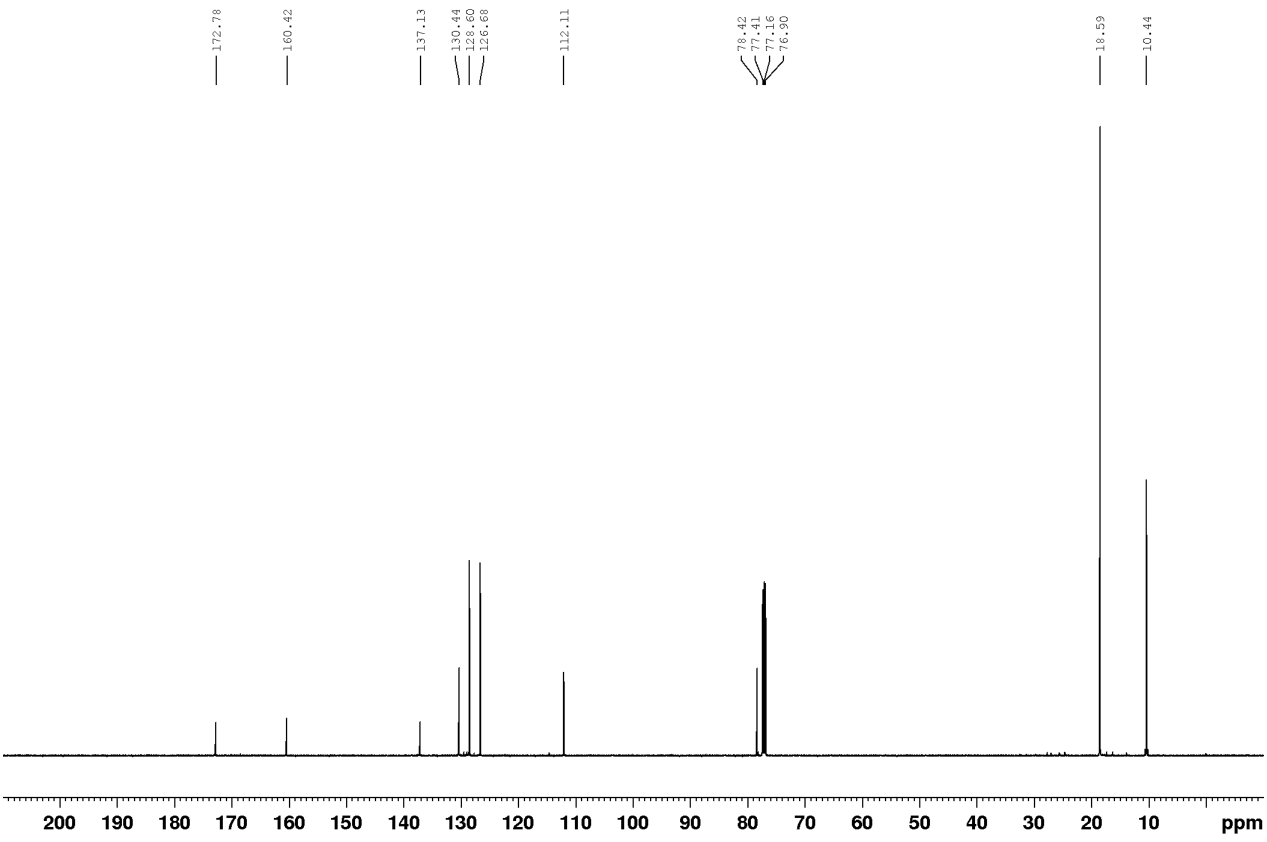

5-Bromo-4,6-diphenyl-2*H*-1,3-oxazine (2q)

^{1}H NMR (500 MHz, CDCl$_3$)

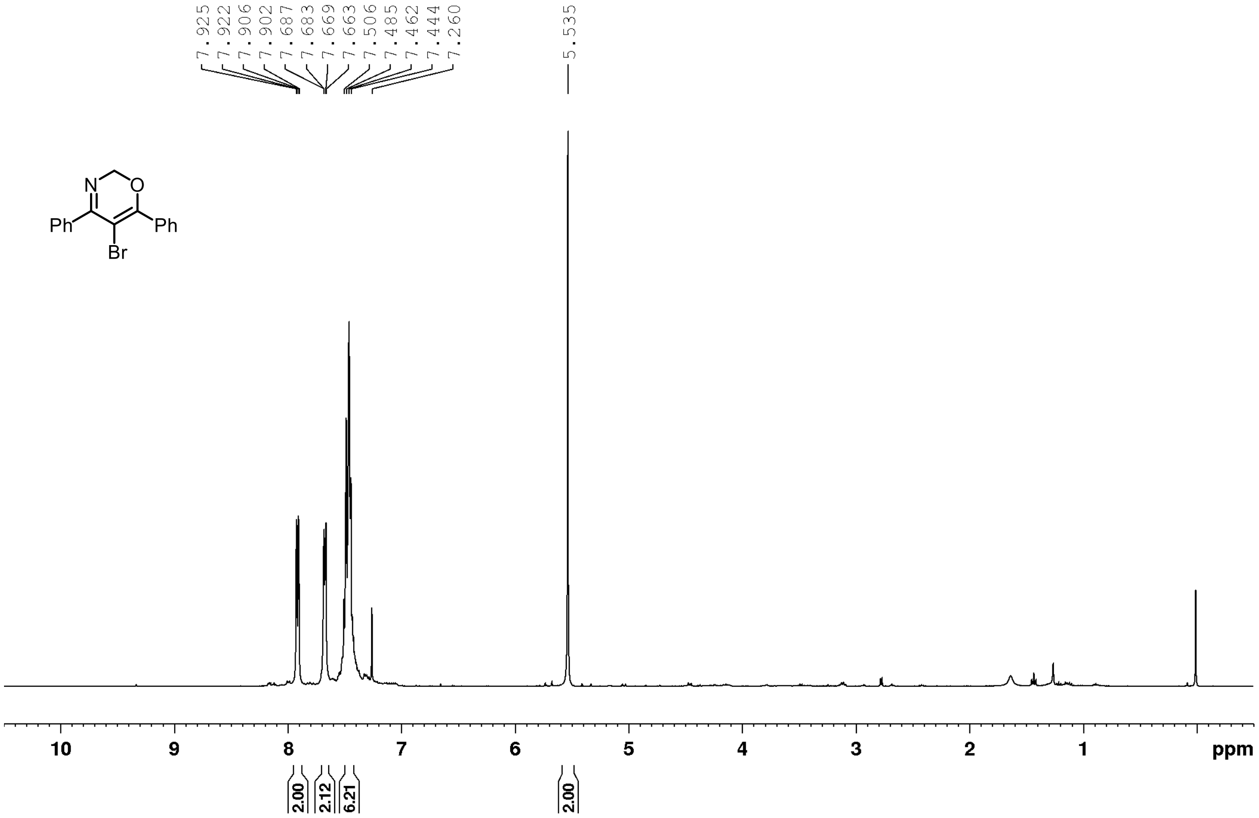

^{13}C NMR (125 MHz, CDCl$_3$)

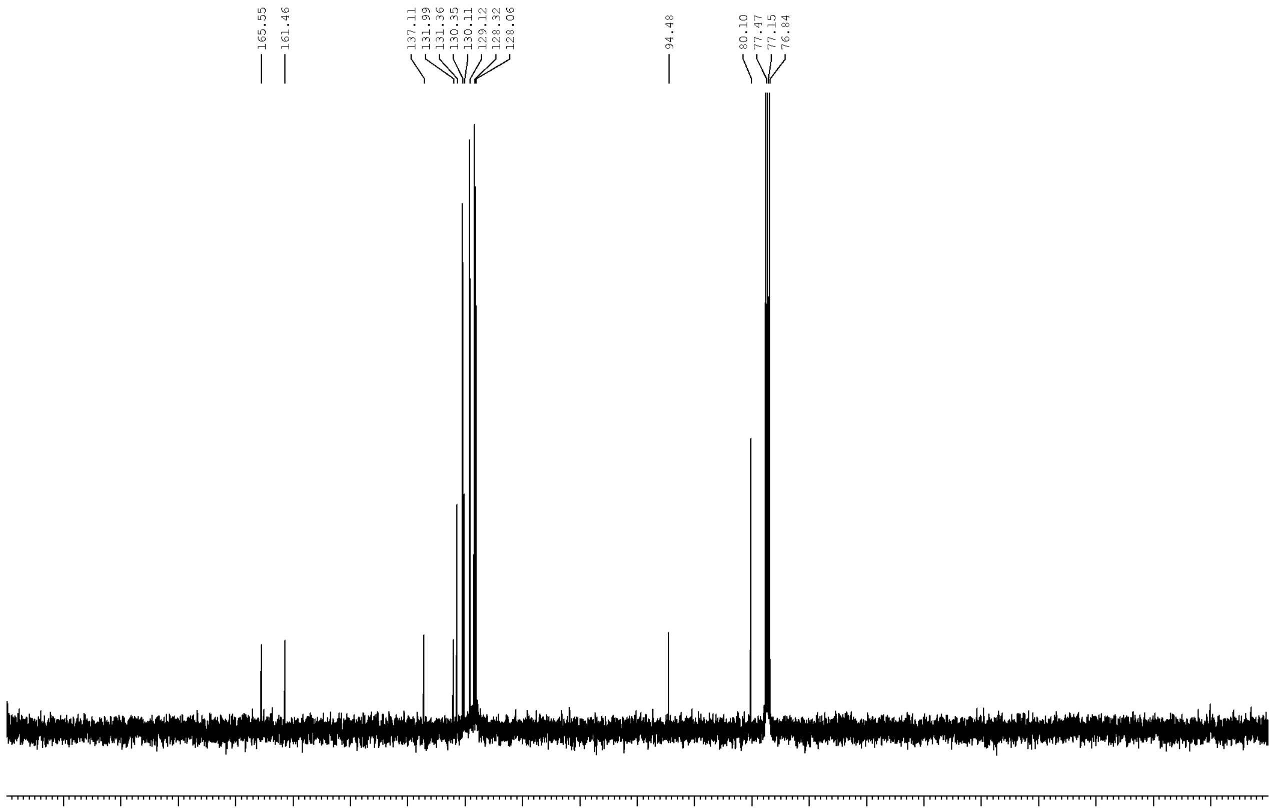

Ethyl 4-(4-chlorophenyl)-6-phenyl-2*H*-1,3-oxazine-5-carboxylate (2r)

^{1}H NMR (400 MHz, $CDCl_3$)

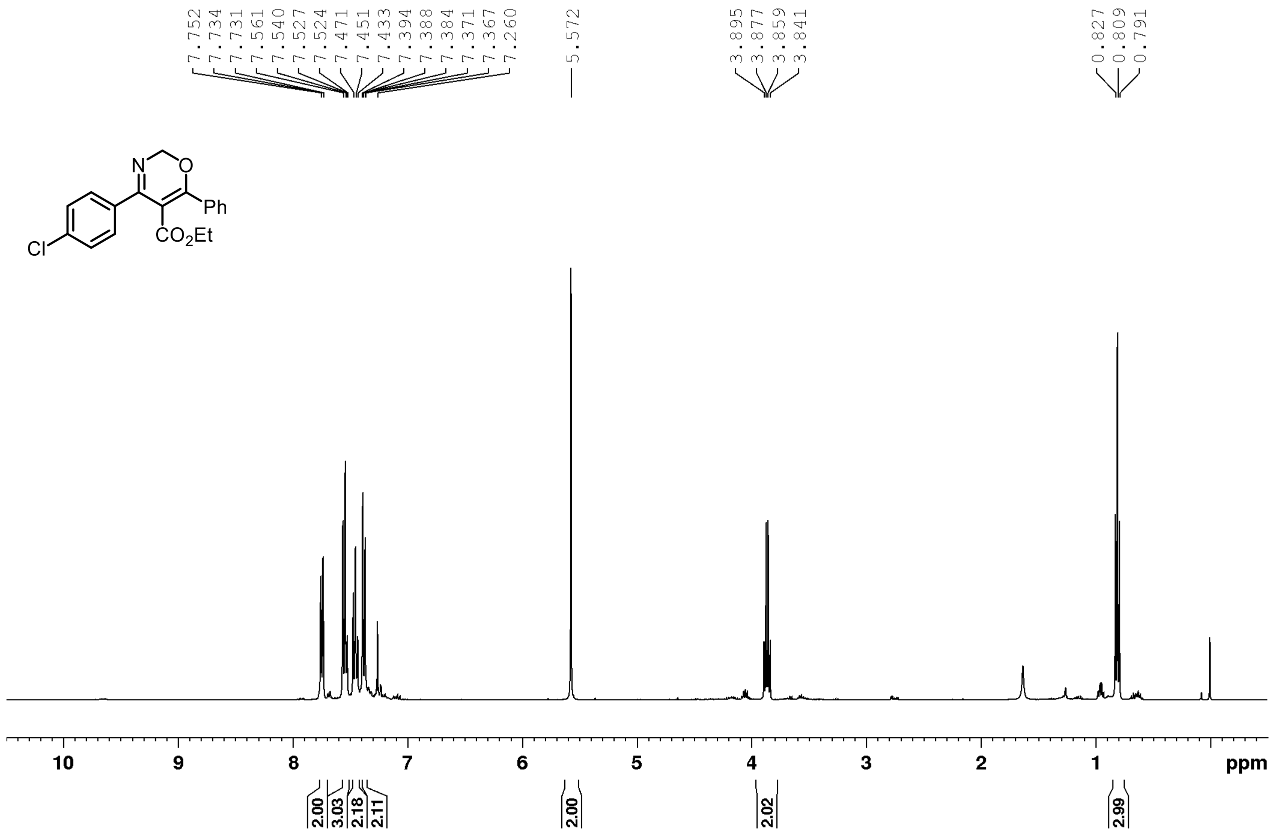

^{13}C NMR (100 MHz, $CDCl_3$)

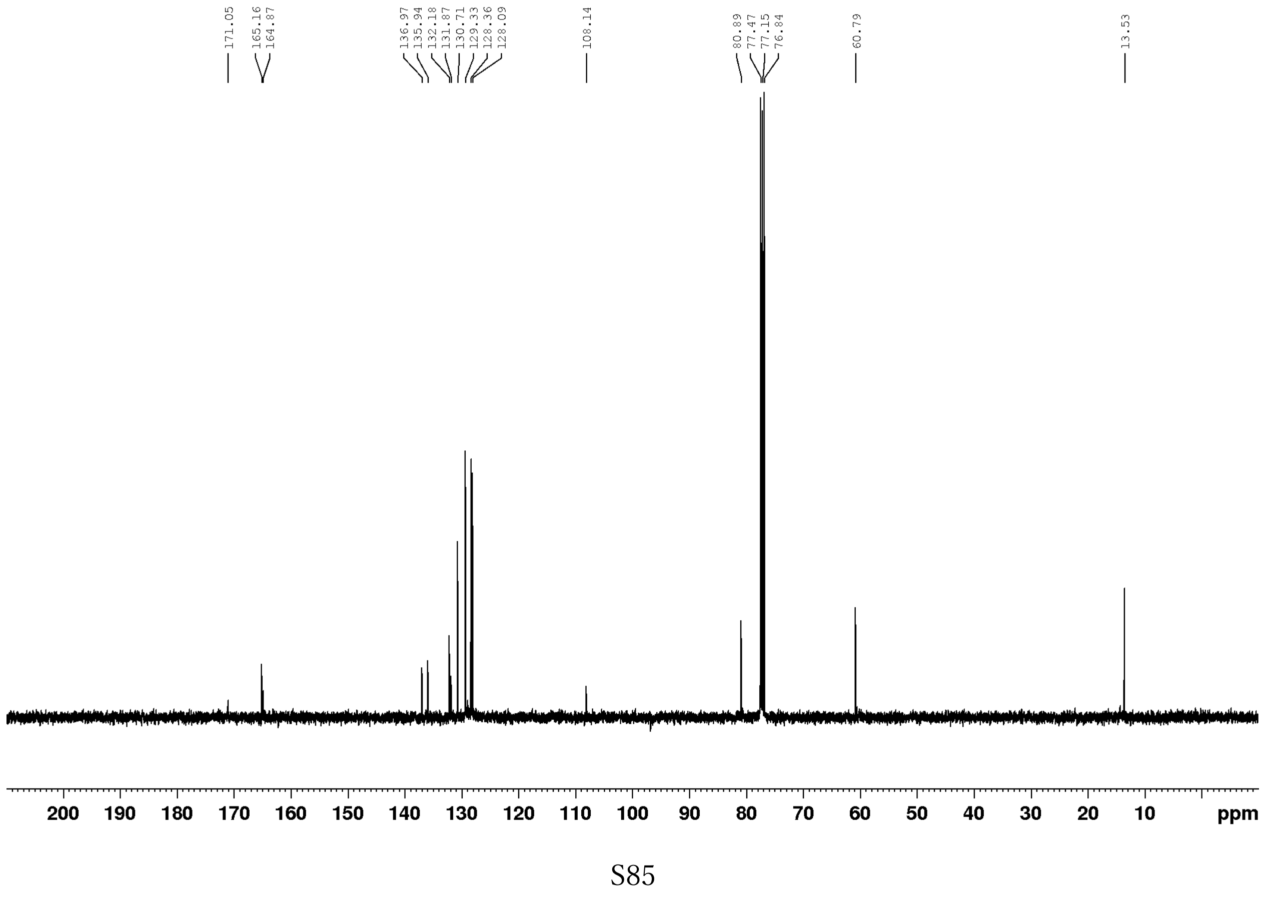

4-Phenyl-2*H*-benzo[*e*][1,3]oxazine (2s)

^{1}H NMR (500 MHz, CDCl$_3$)

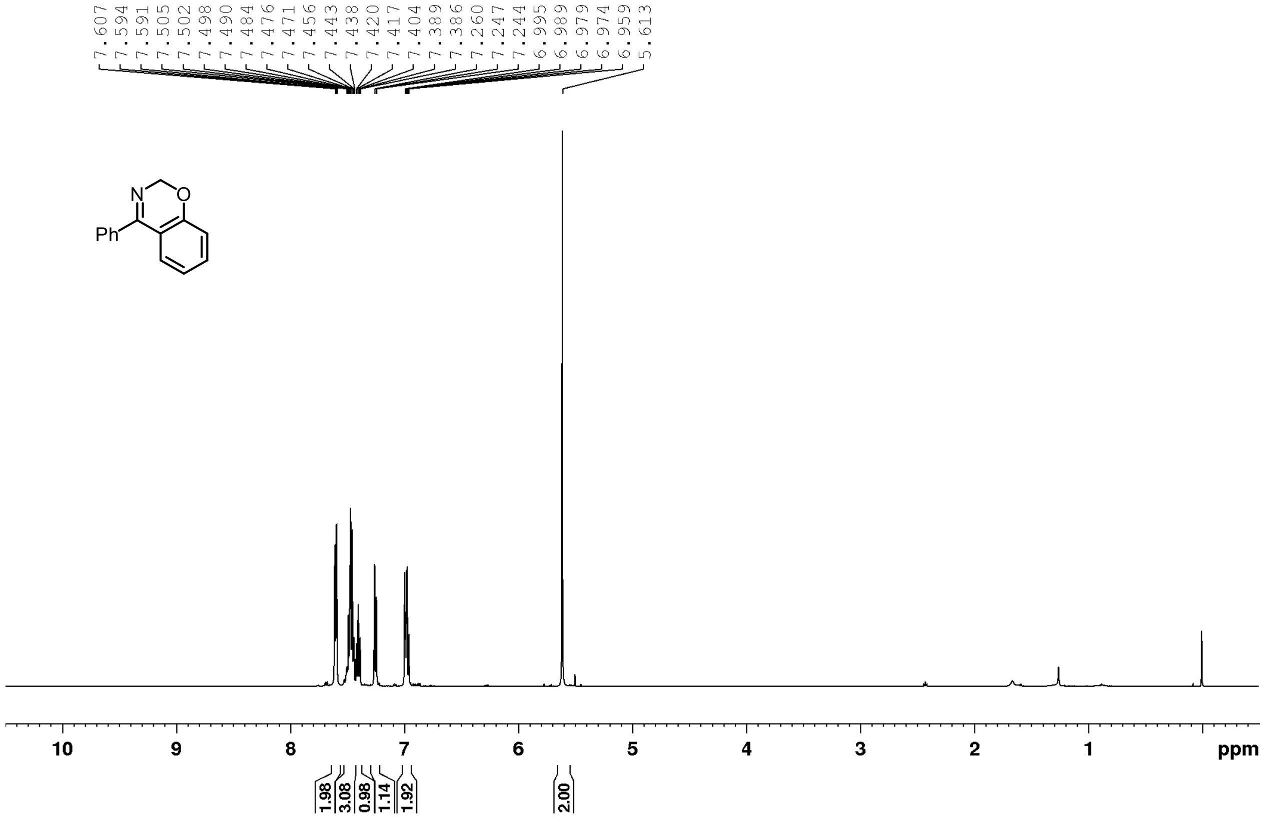

^{13}C NMR (125 MHz, CDCl$_3$)

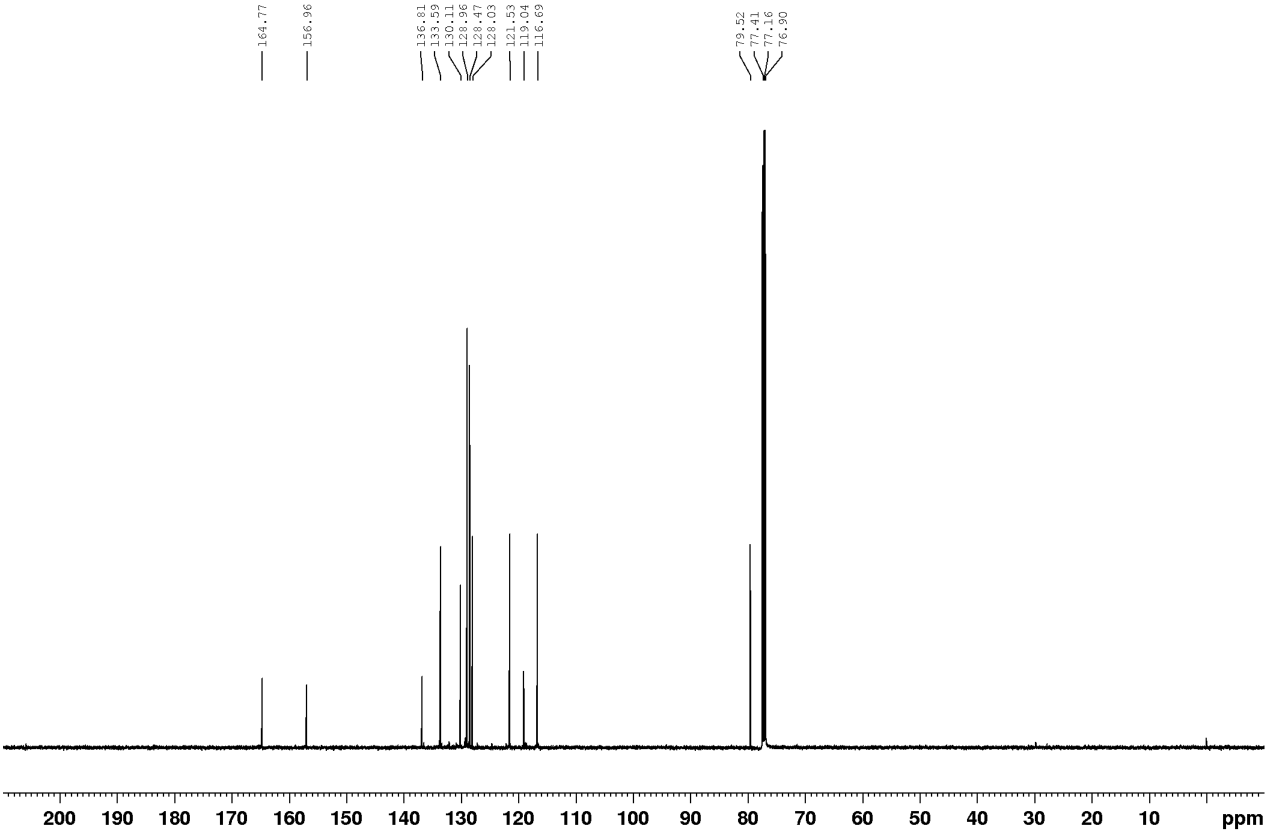

Ethyl 2*H*-benzo[*e*][1,3]oxazine-4-carboxylate (2t)

^{1}H NMR (400 MHz, $CDCl_3$)

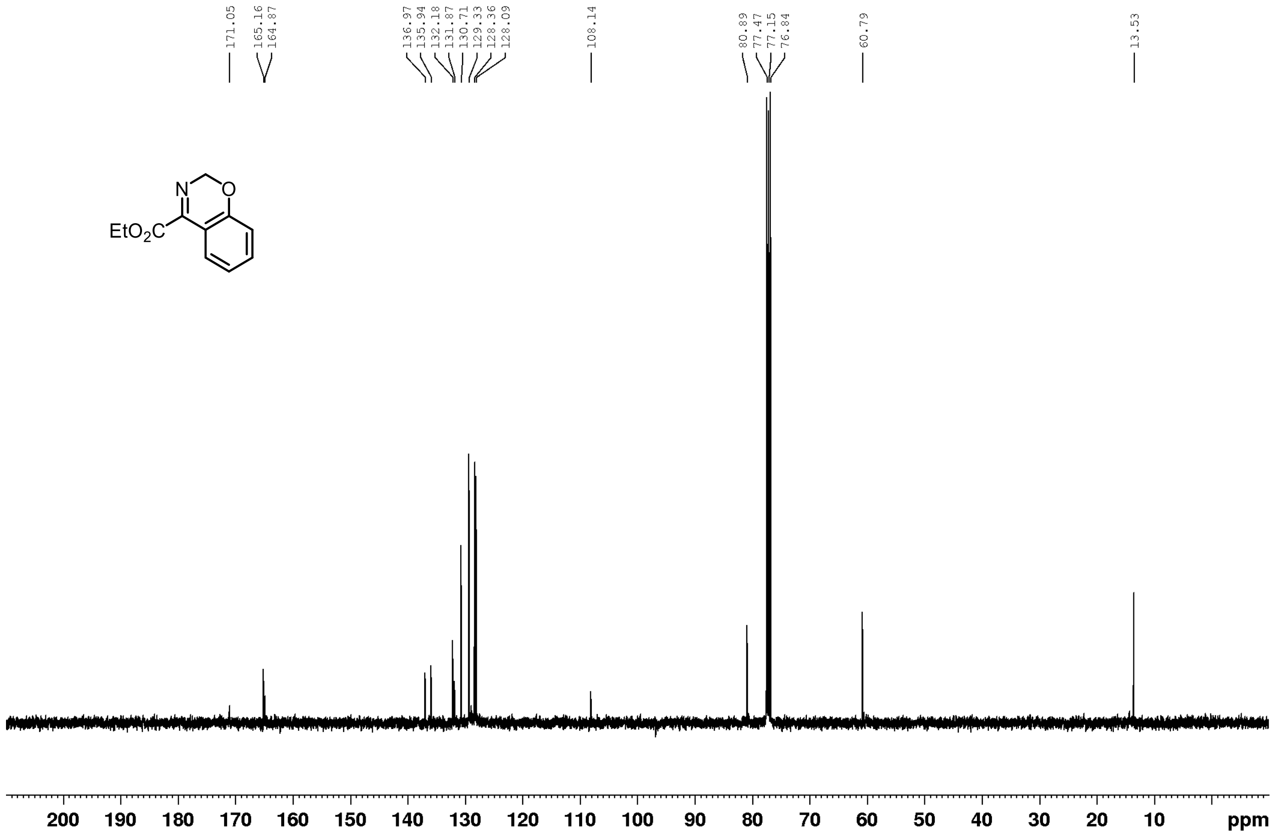

^{13}C NMR (100 MHz, $CDCl_3$)

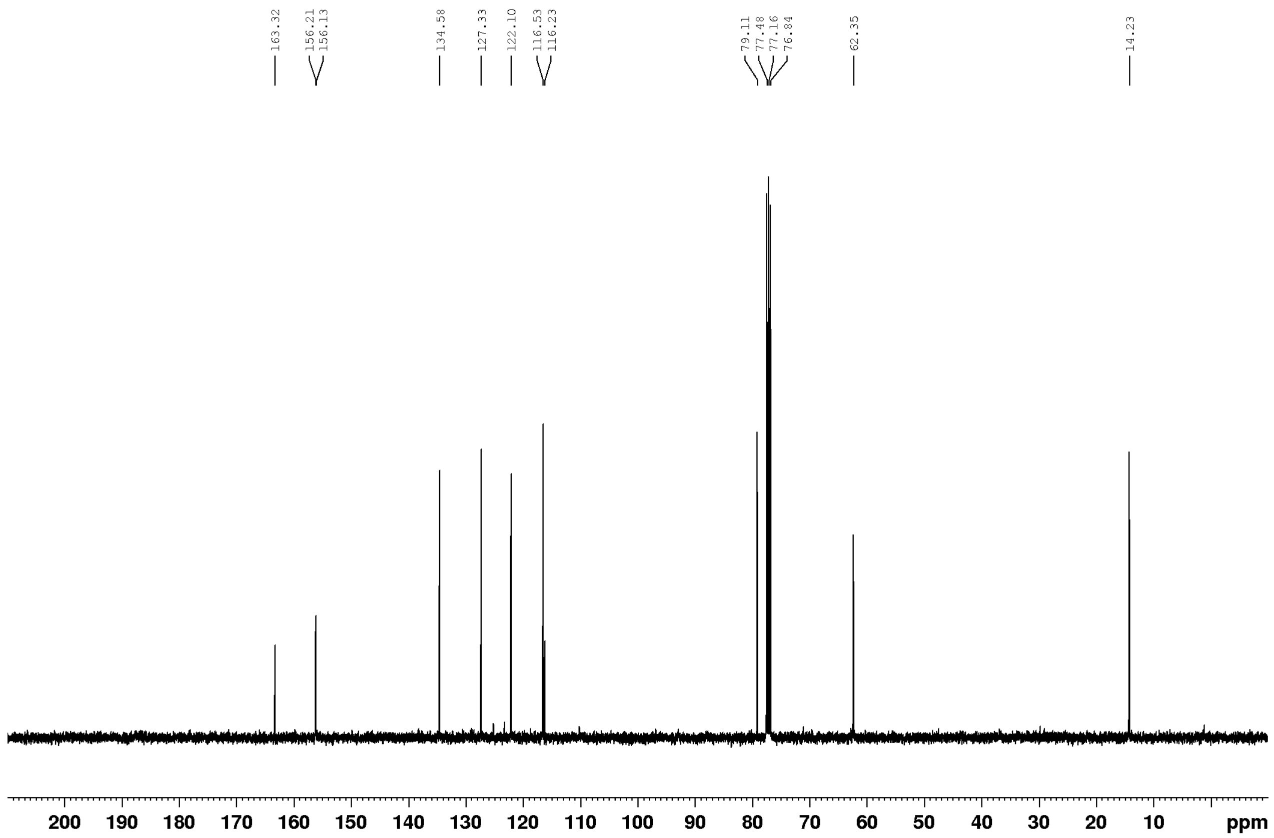

(2*H*-benzo[*e*][1,3]oxazin-4-yl)methanesulfonamide (2u)

^{1}H NMR (500 MHz, CD_3CN)

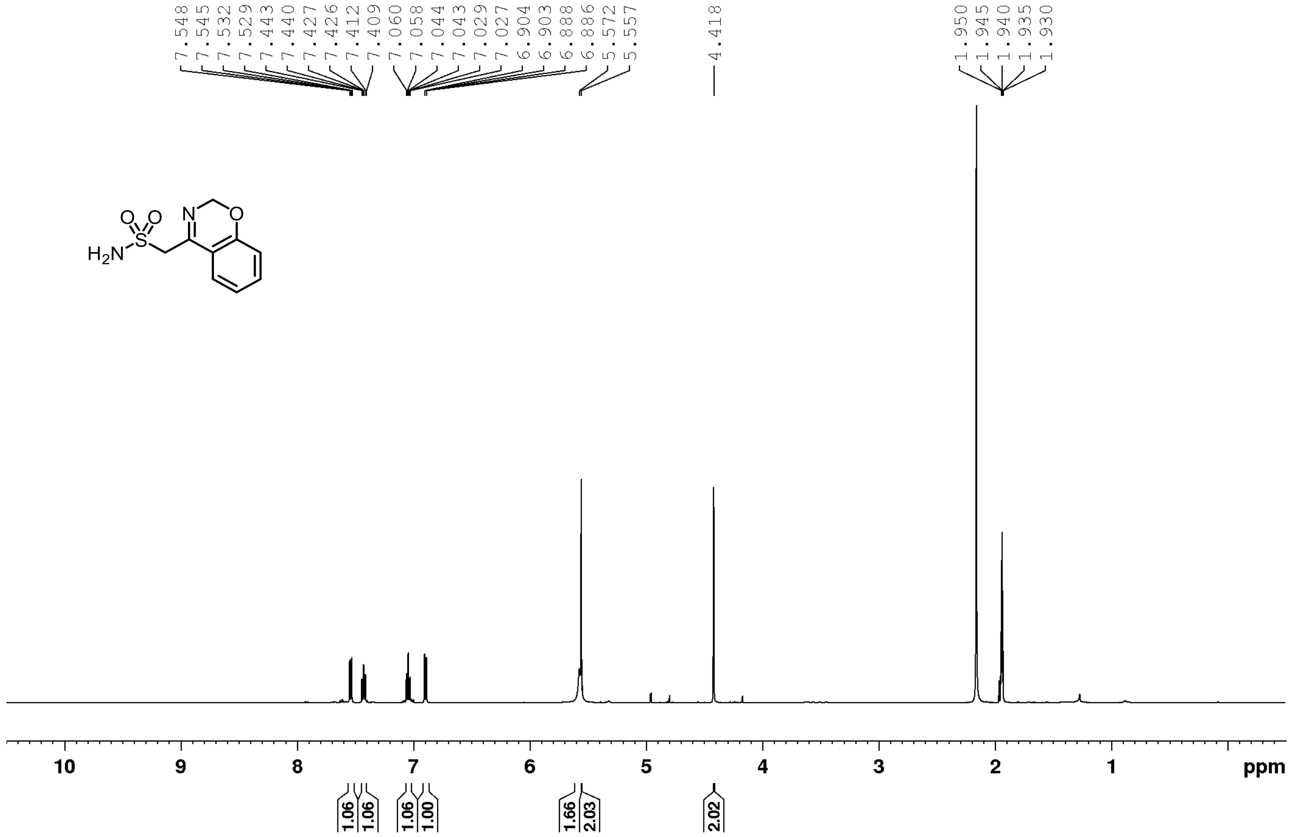

^{13}C NMR (125 MHz, CD_3CN)

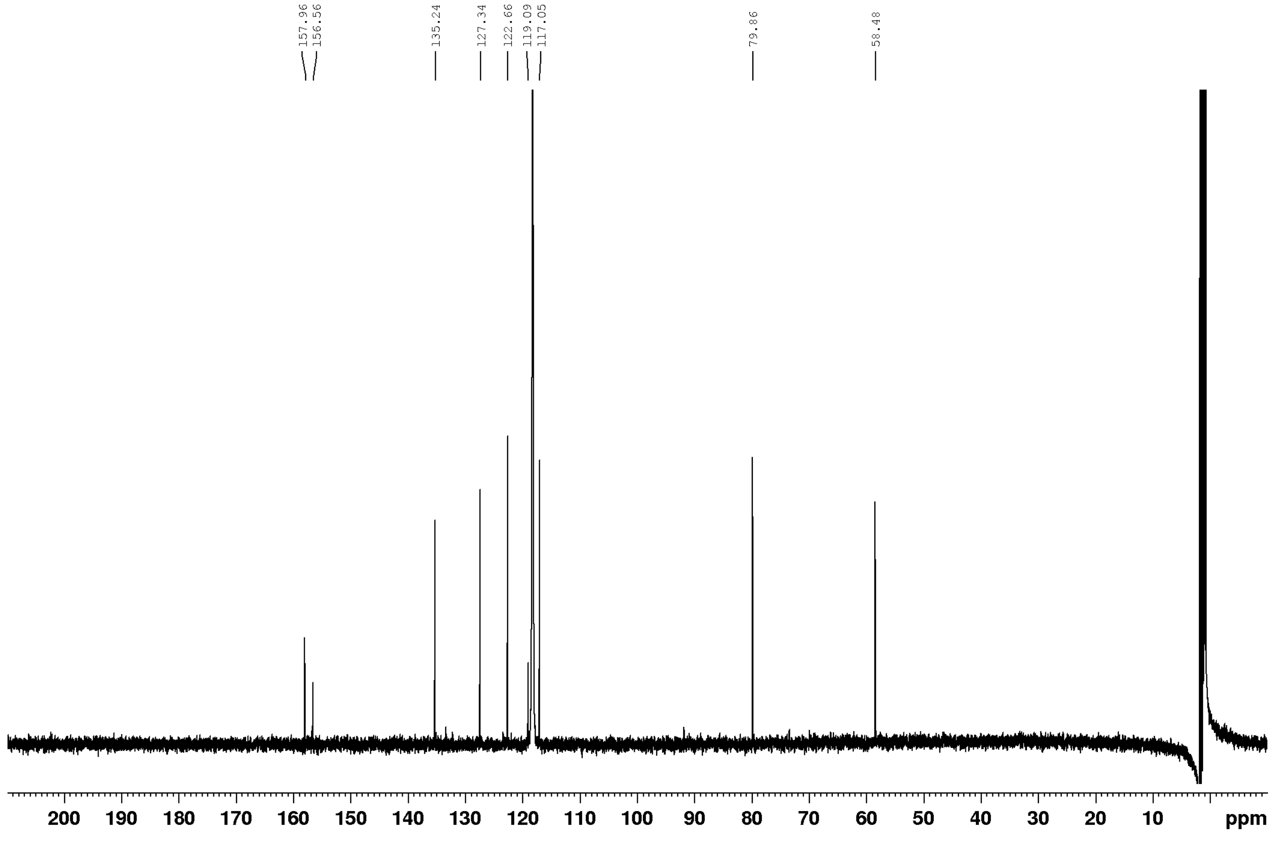

4-(4-(4-phenoxyphenyl)-1*H*-1,2,3-triazol-1-yl)-2*H*-benzo[*e*][1,3]oxazine (2v)

^{1}H NMR (500 MHz, $CDCl_3$)

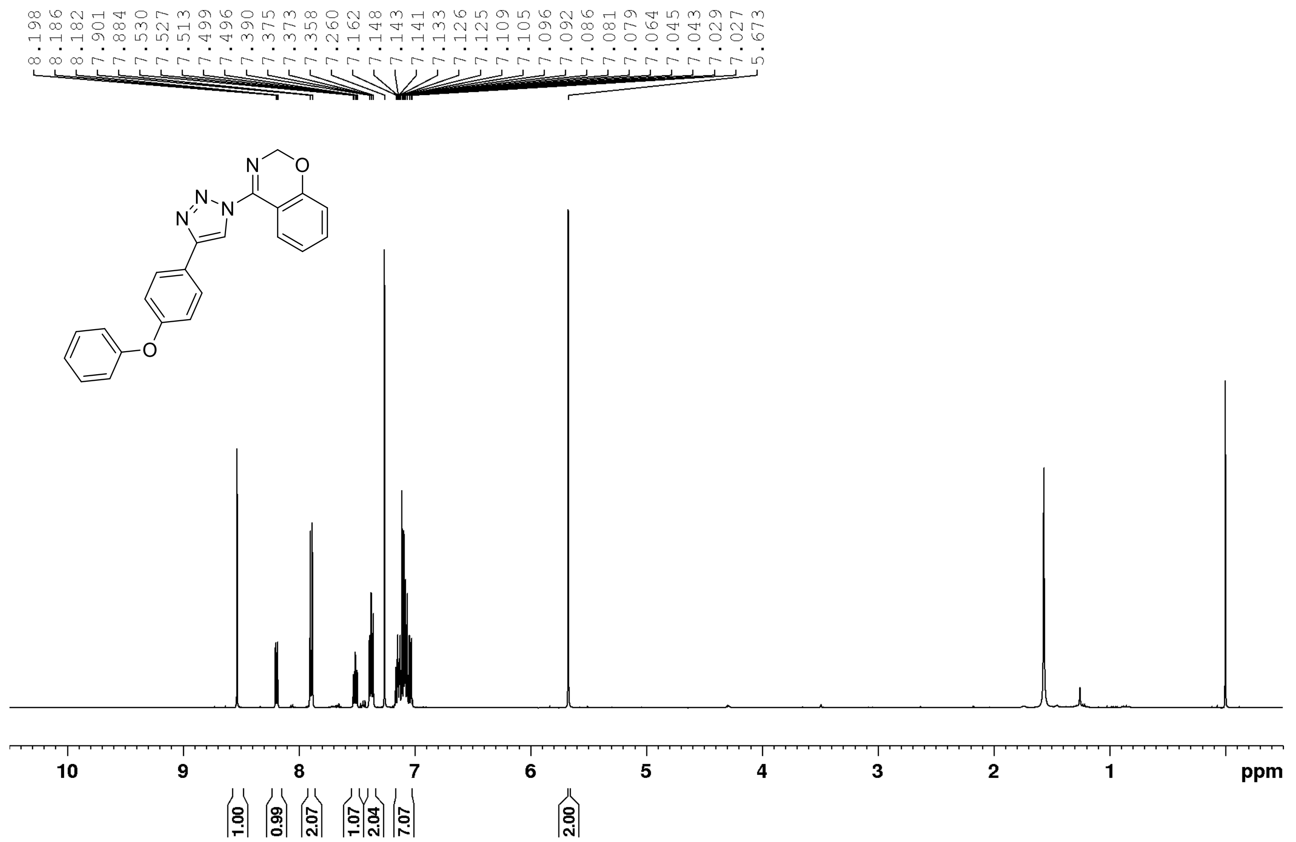

^{13}C NMR (125 MHz, $CDCl_3$)

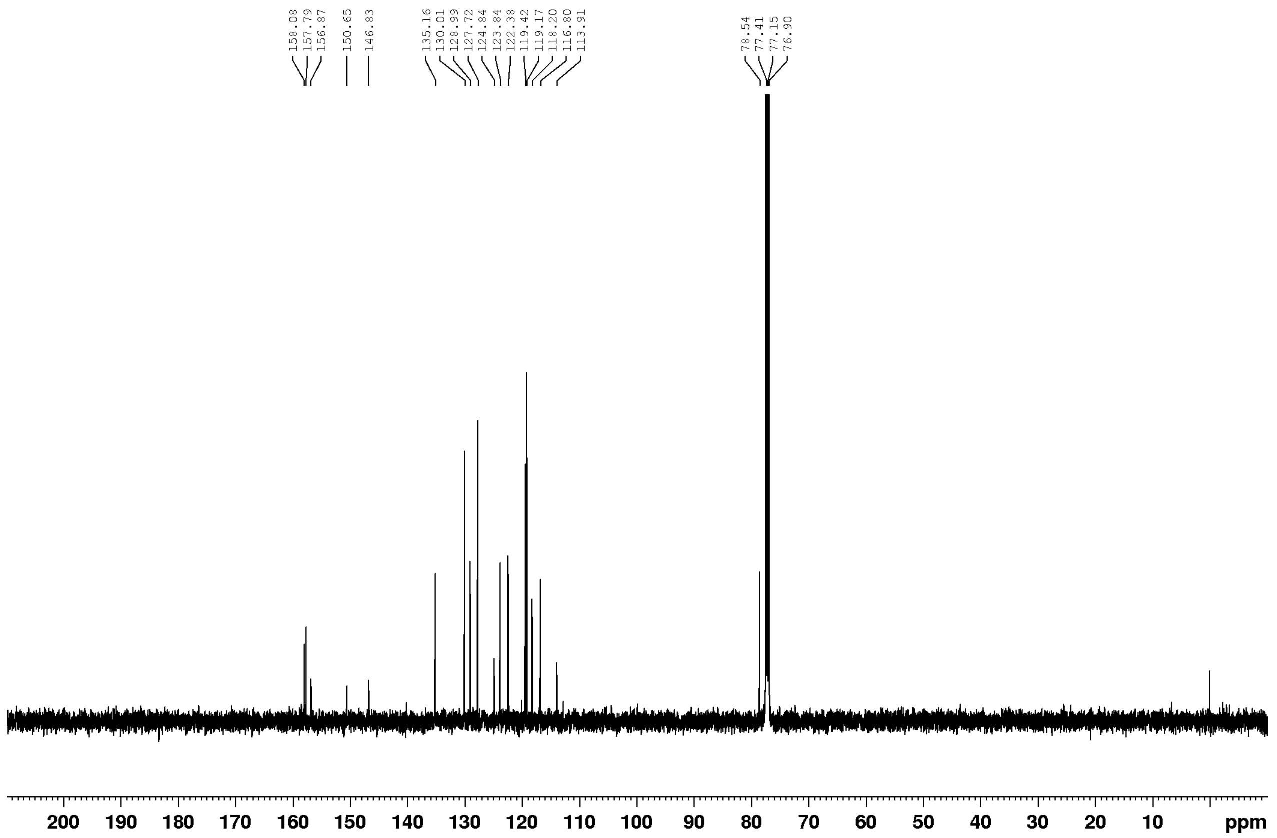

4,6-Diphenyl-2*H*-1,3-thiazine (4a)

^{1}H NMR (500 MHz, $CDCl_3$)

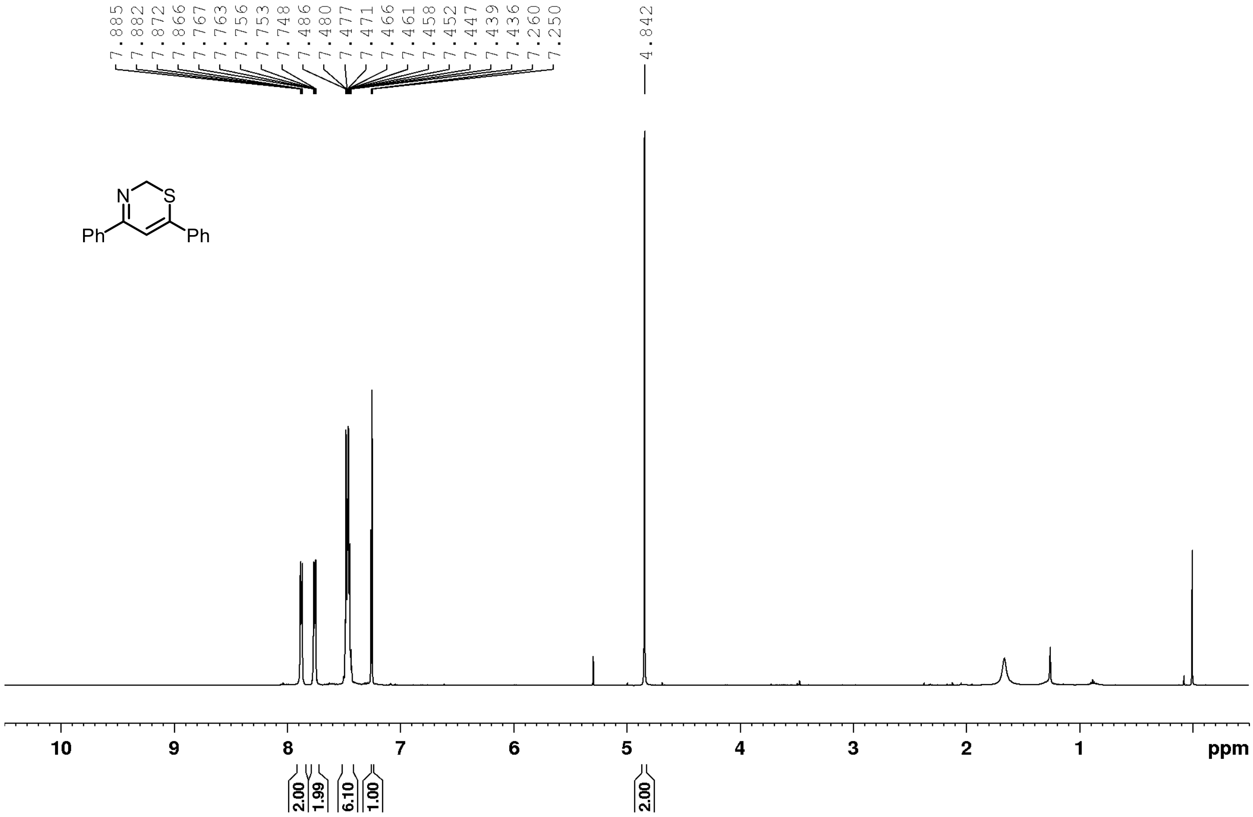

^{13}C NMR (125 MHz, $CDCl_3$)

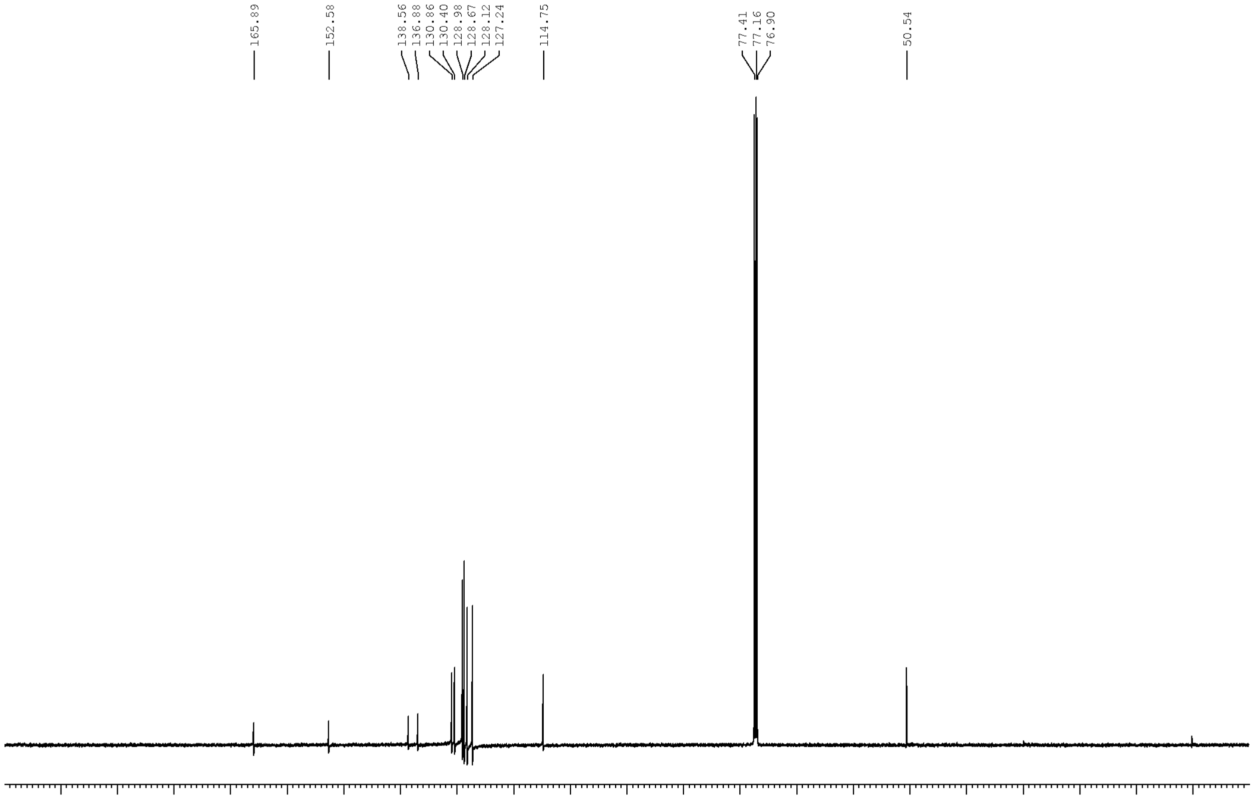

4,6-Diphenyl-1-tosyl-1,2-dihydropyrimidine (4b)

^{1}H NMR (500 MHz, CDCl$_3$)

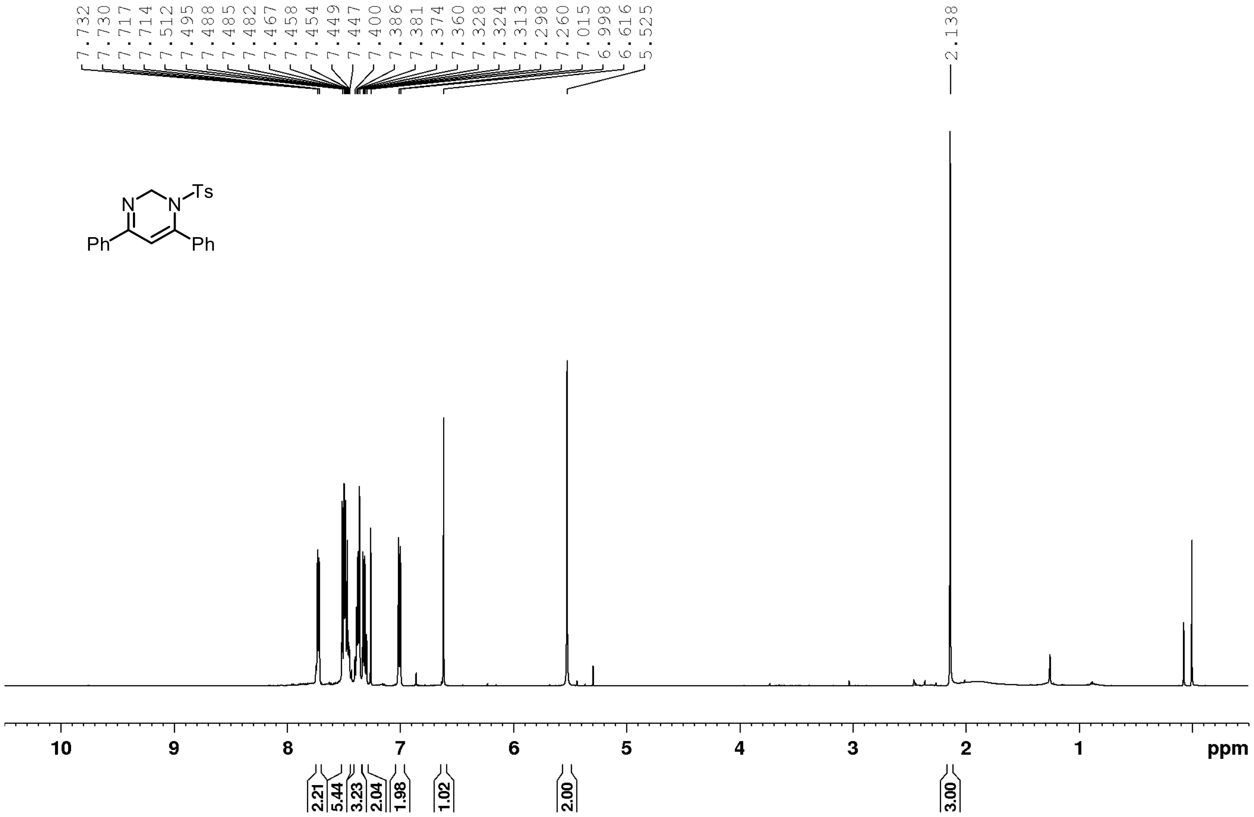

^{13}C NMR (125 MHz, CDCl$_3$)

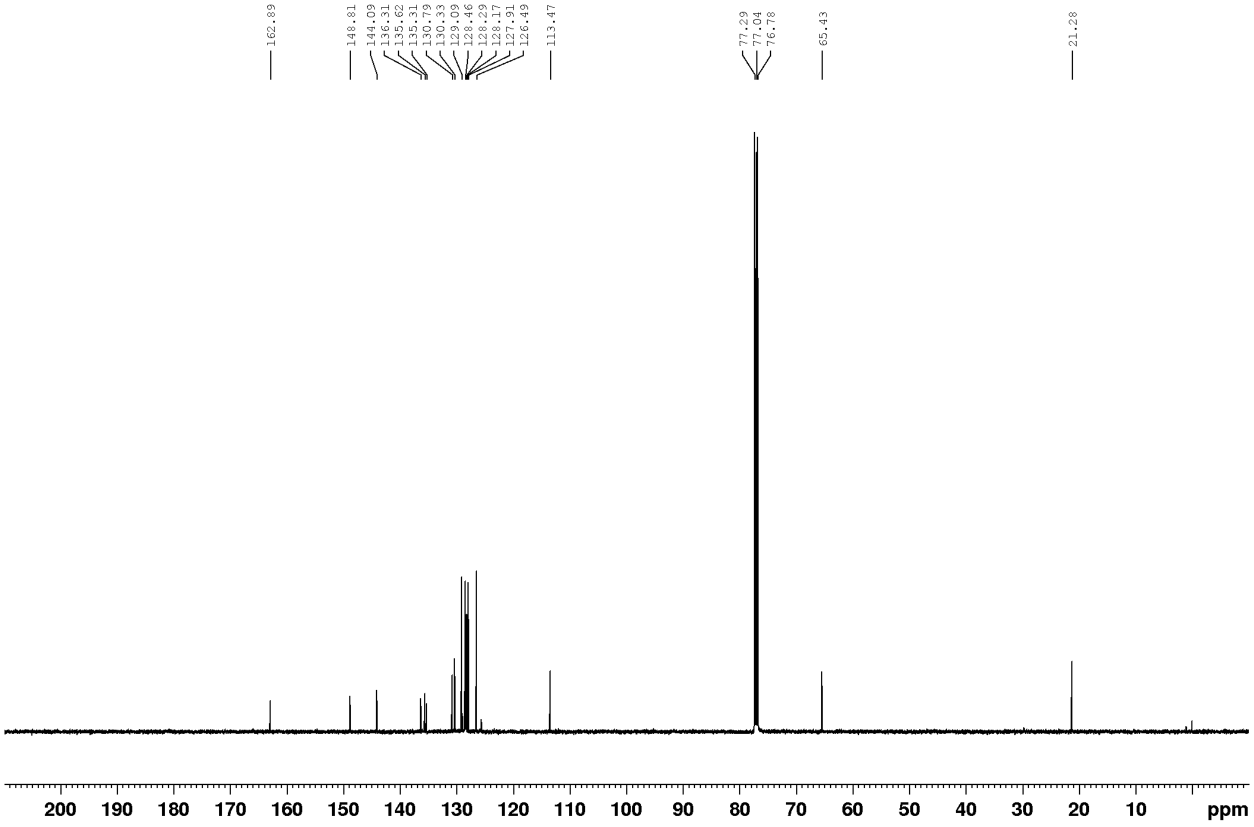

1-Methoxy-1,2-dihydroquinazoline (4c)

^{1}H NMR (500 MHz, $CDCl_3$)

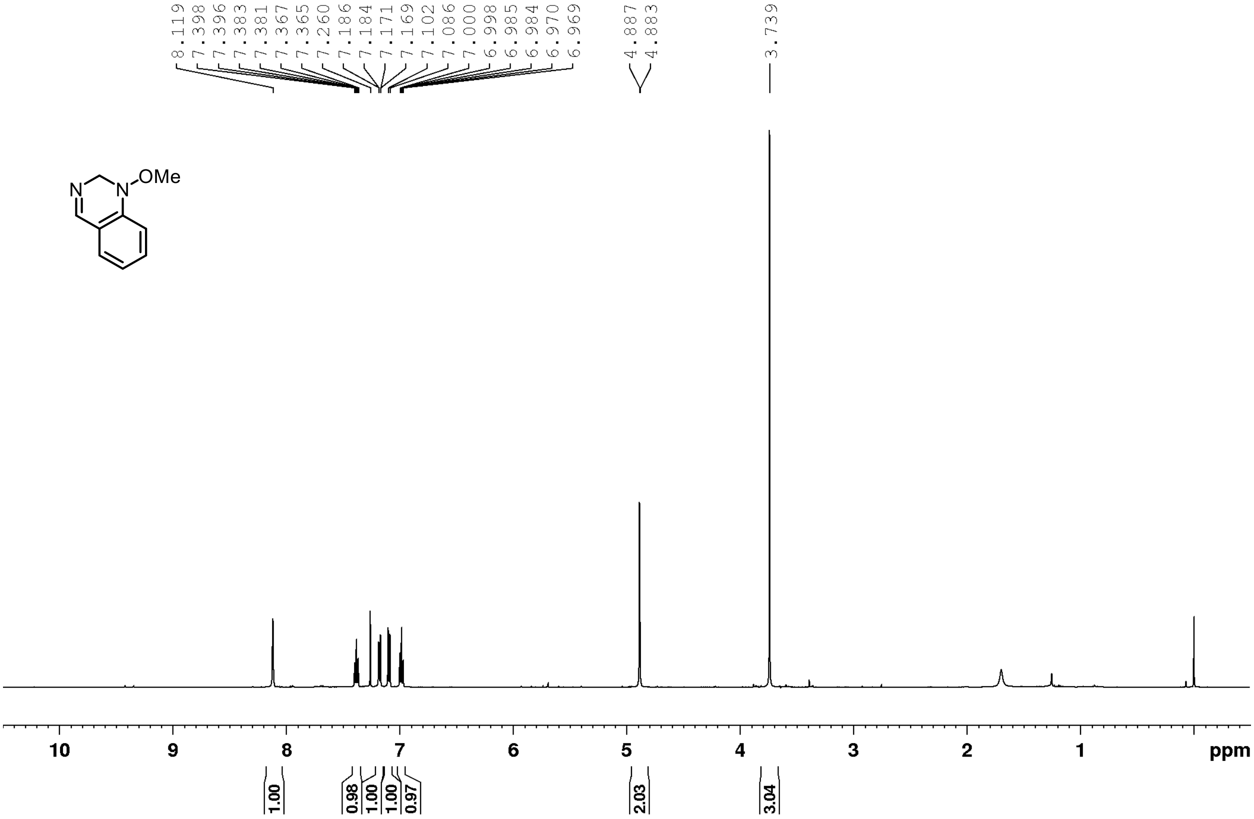

^{13}C NMR (125 MHz, $CDCl_3$)

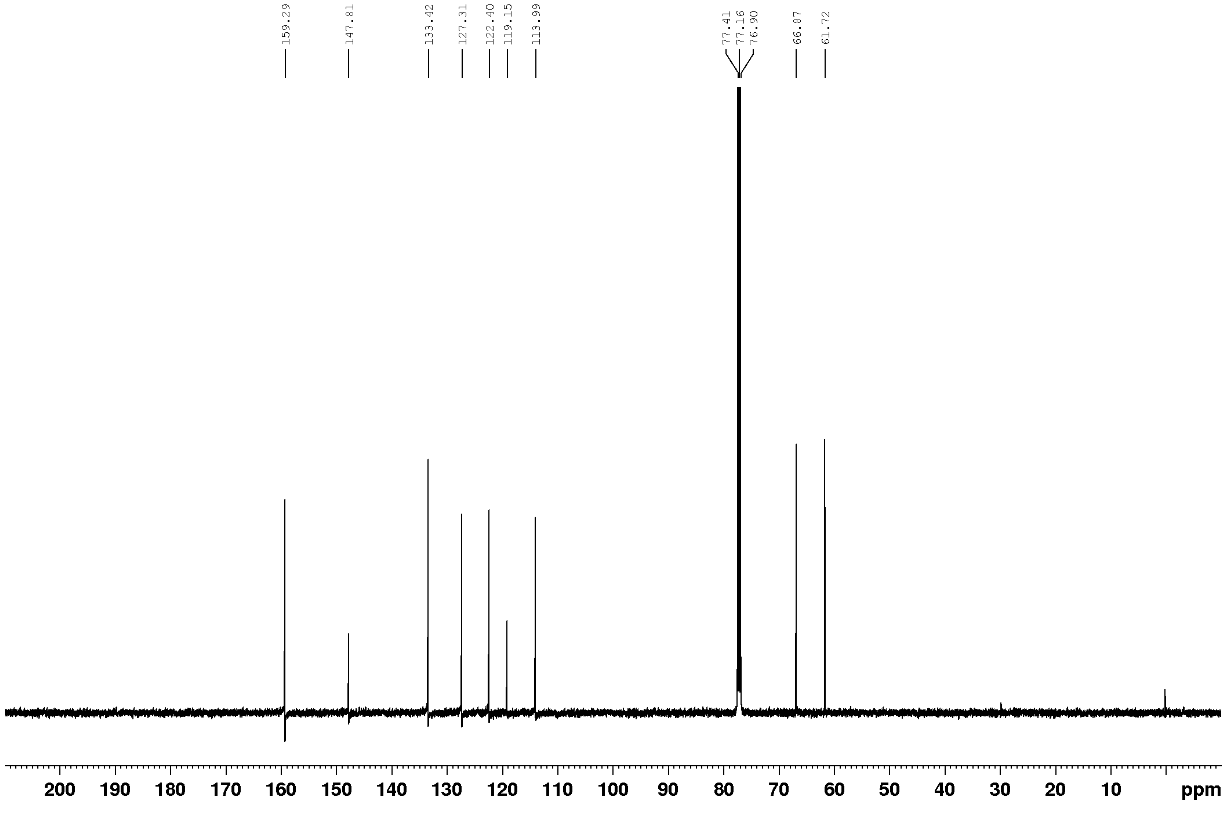

1-(Benzyloxy)-1,2-dihydroquinazoline (4d)

^{1}H NMR (500 MHz, CDCl$_3$)

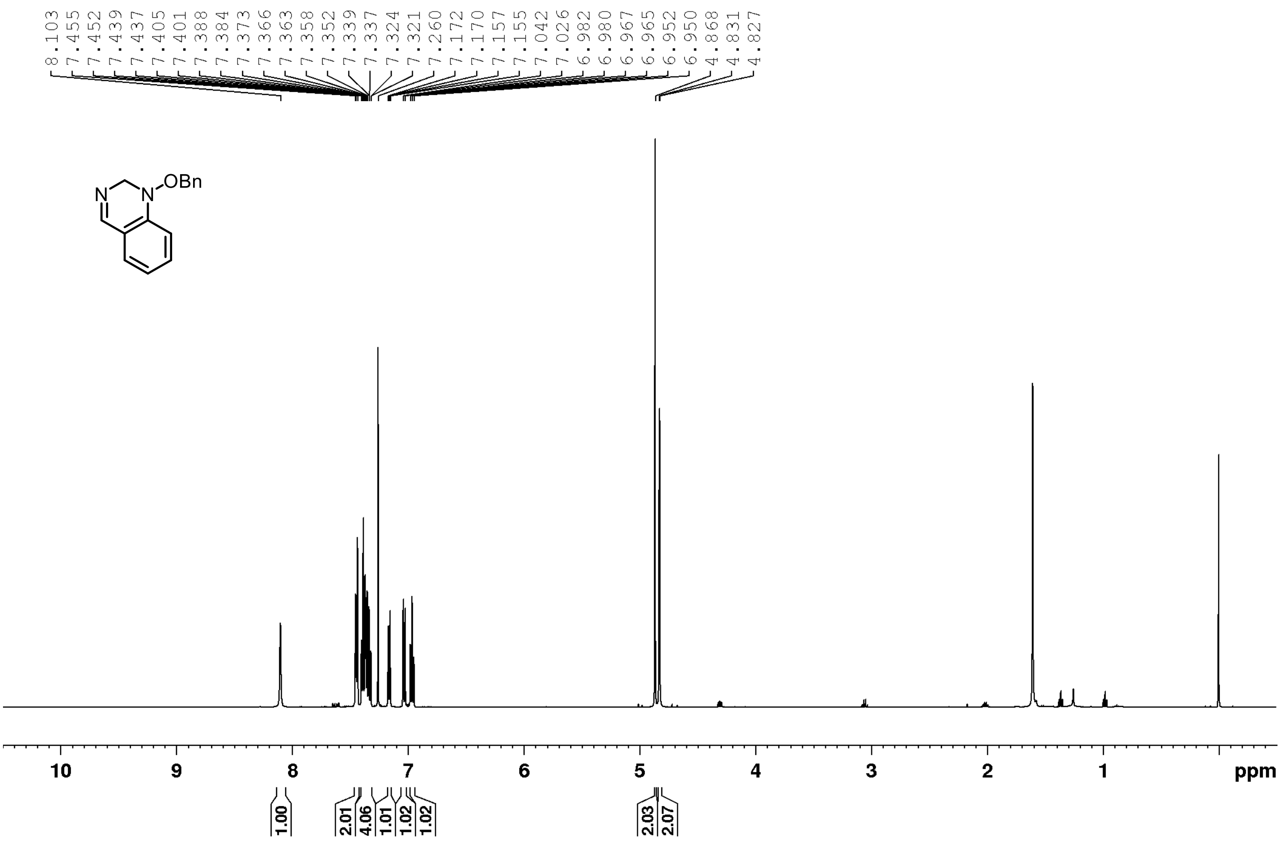

^{13}C NMR (125 MHz, CDCl$_3$)

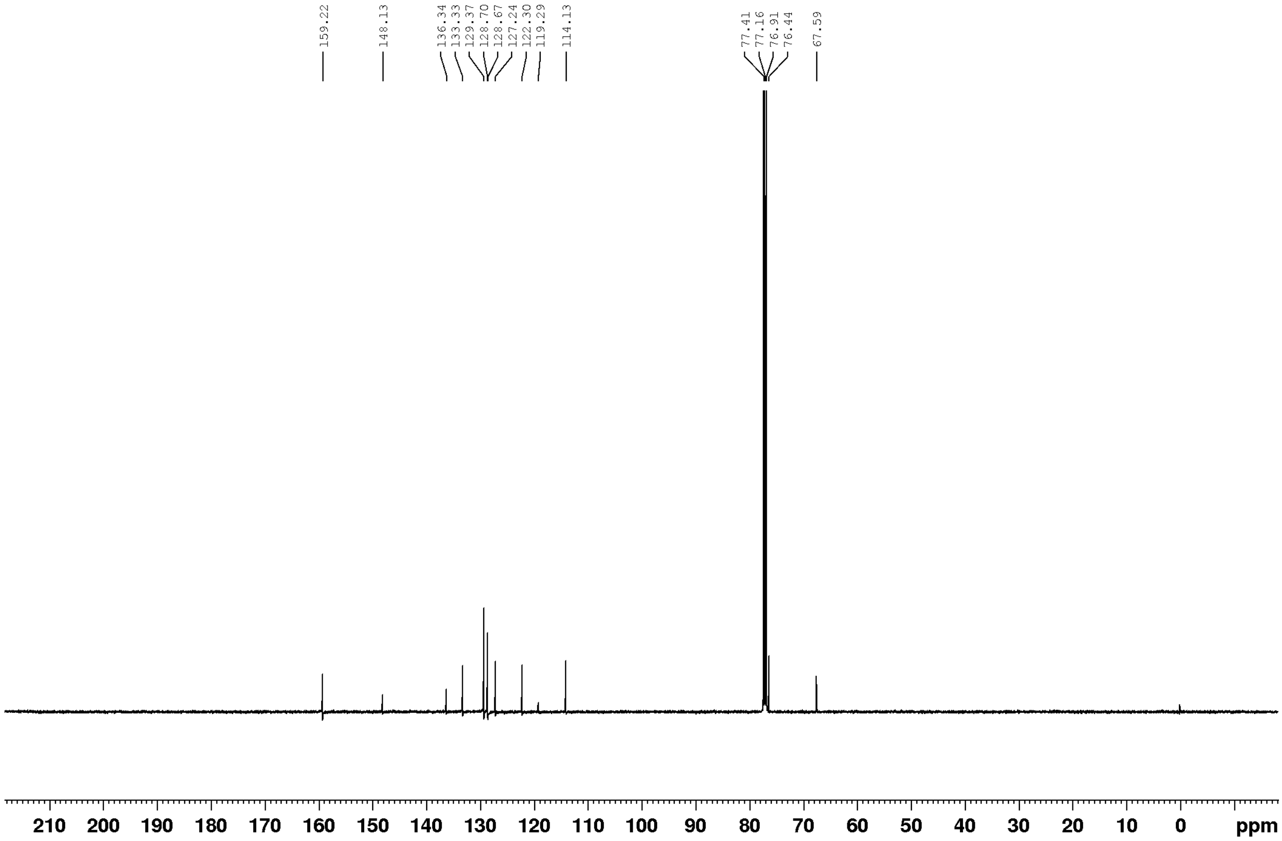

Quinazolin-1(2*H*)-yl 2,4,6-trimethylbenzoate (4e)

^{1}H NMR (500 MHz, CDCl₃)

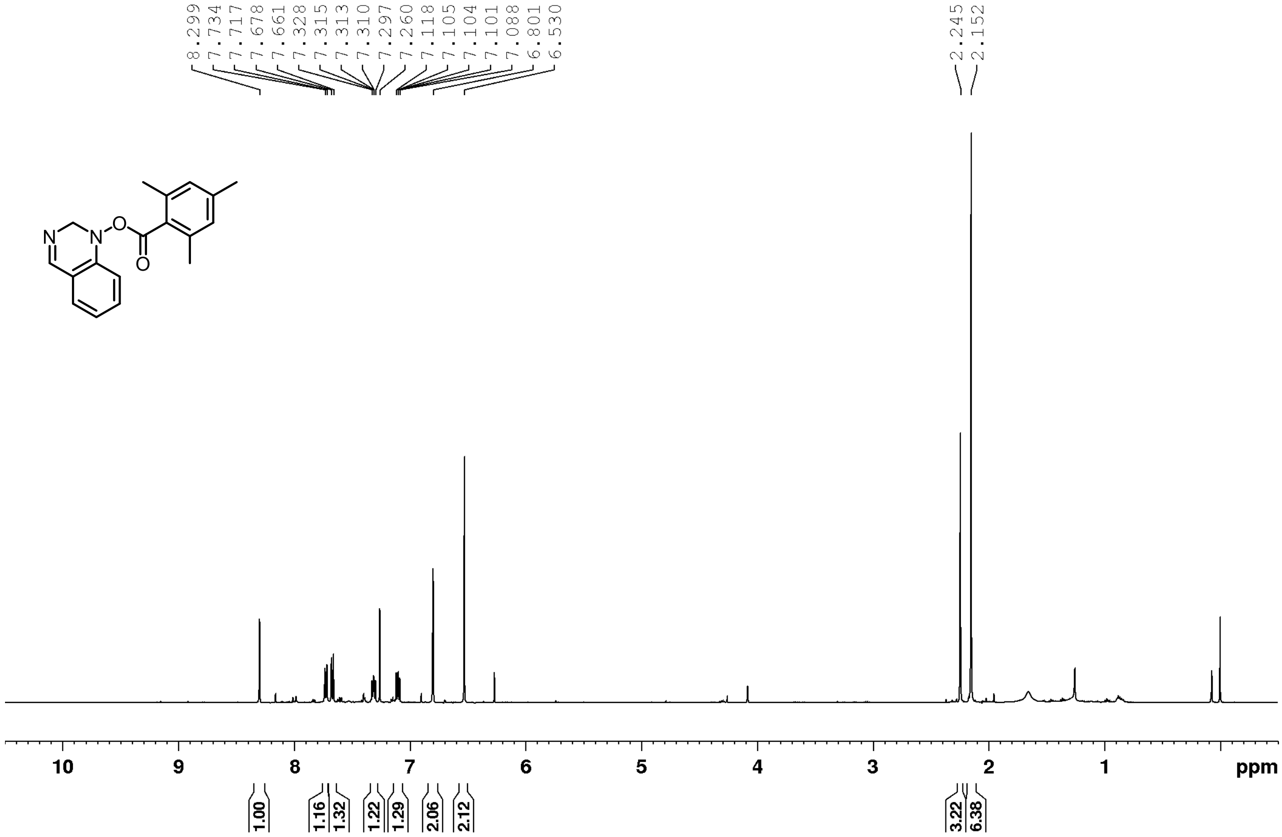

^{13}C NMR (125 MHz, CDCl₃)

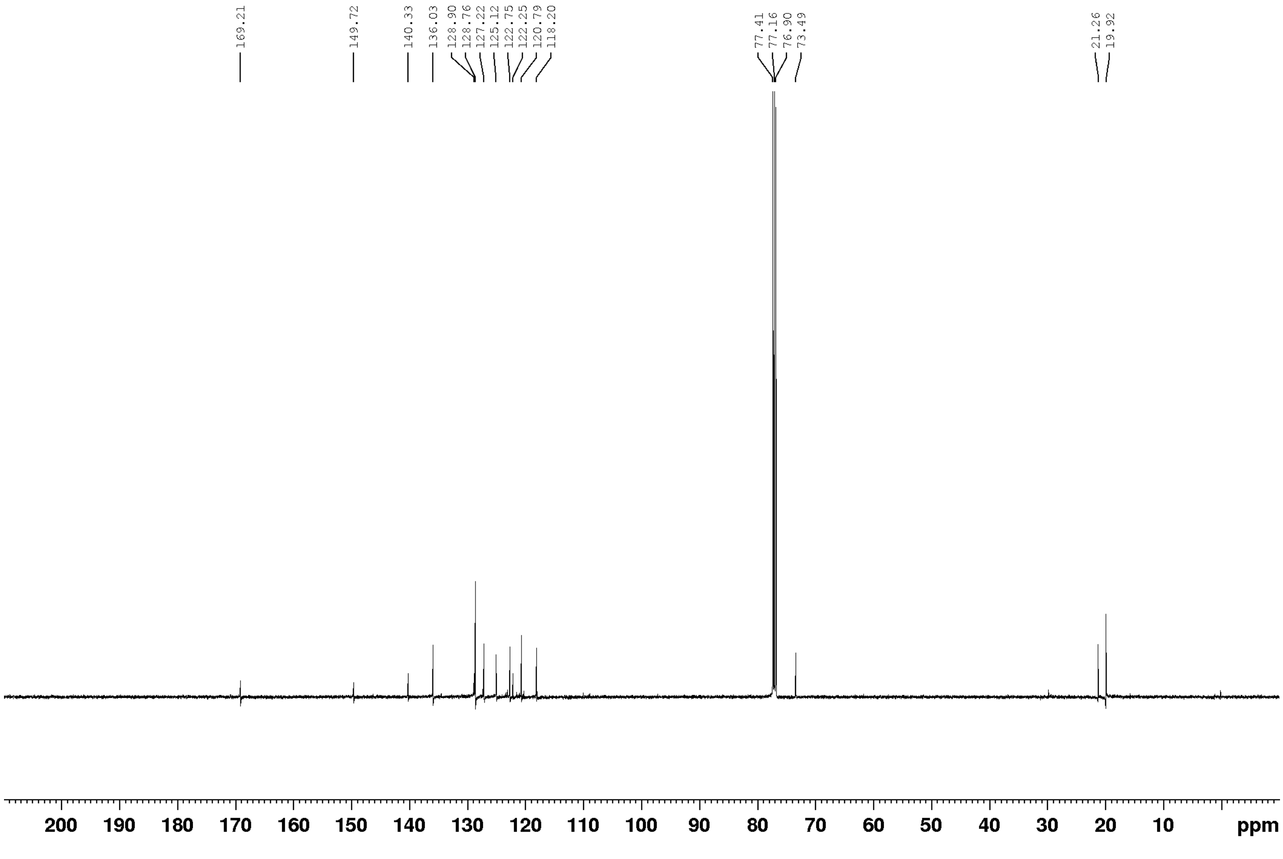

4,6-Diphenyl-5,6-dihydro-2*H*-1,3-oxazine (4f)

^{1}H NMR (400 MHz, CDCl$_3$)

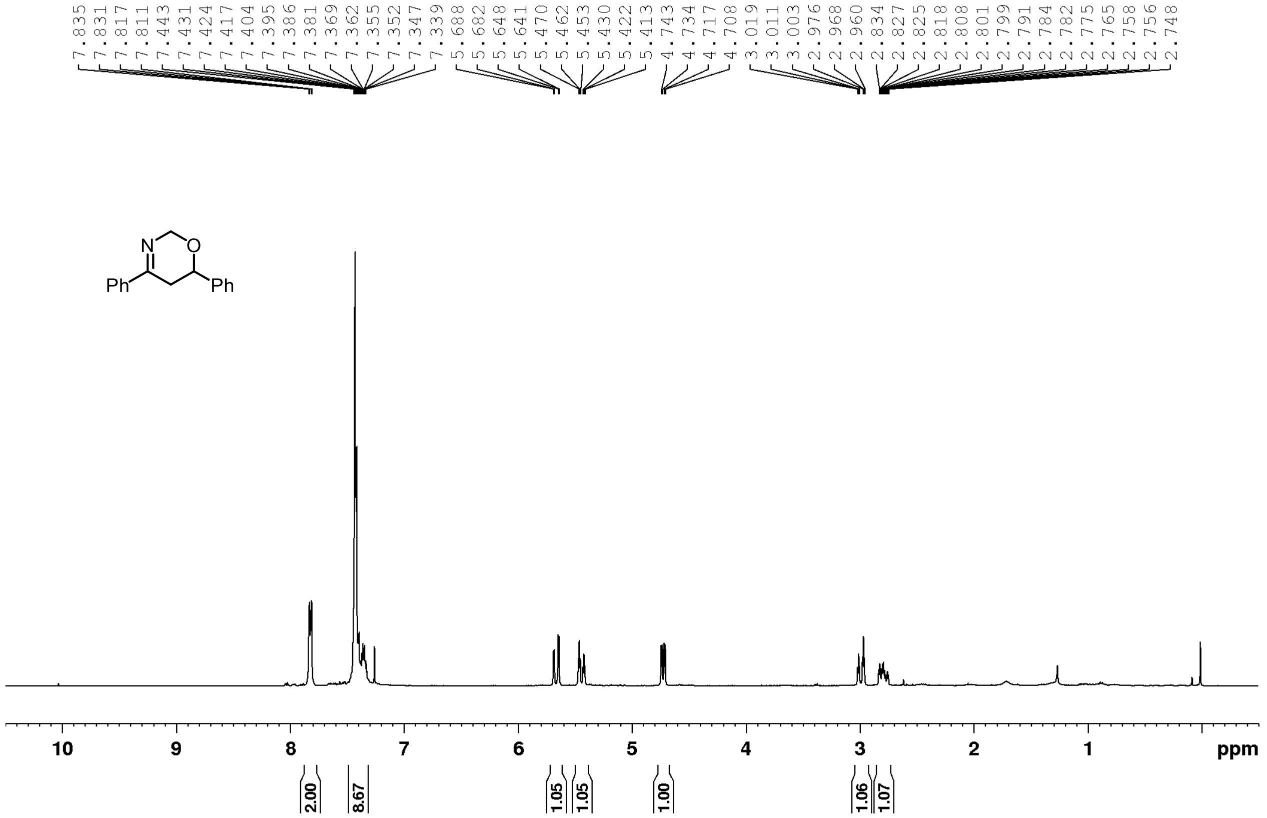

^{13}C NMR (100 MHz, CDCl$_3$)

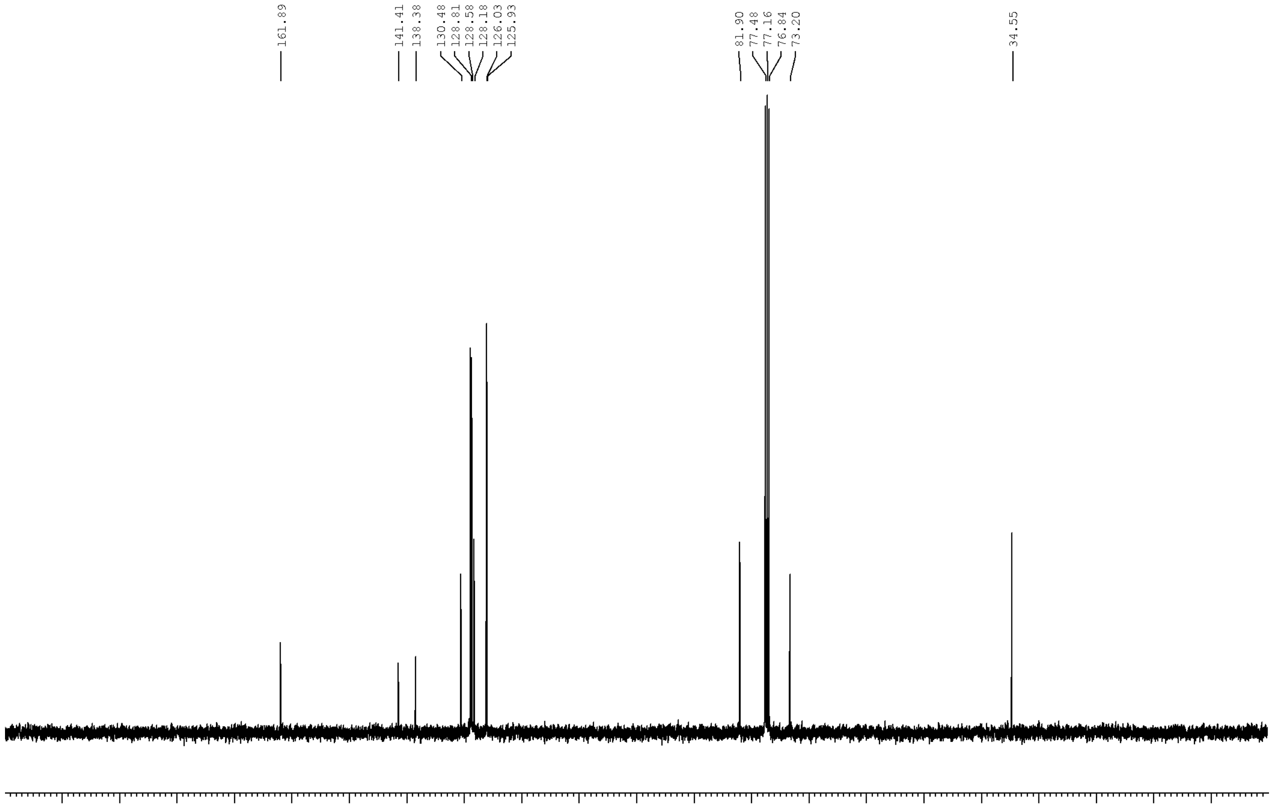

6-Phenyl-4-(*p*-tolyl)-5,6-dihydro-2*H*-1,3-oxazine (4g)

^{1}H NMR (400 MHz, CDCl$_3$)

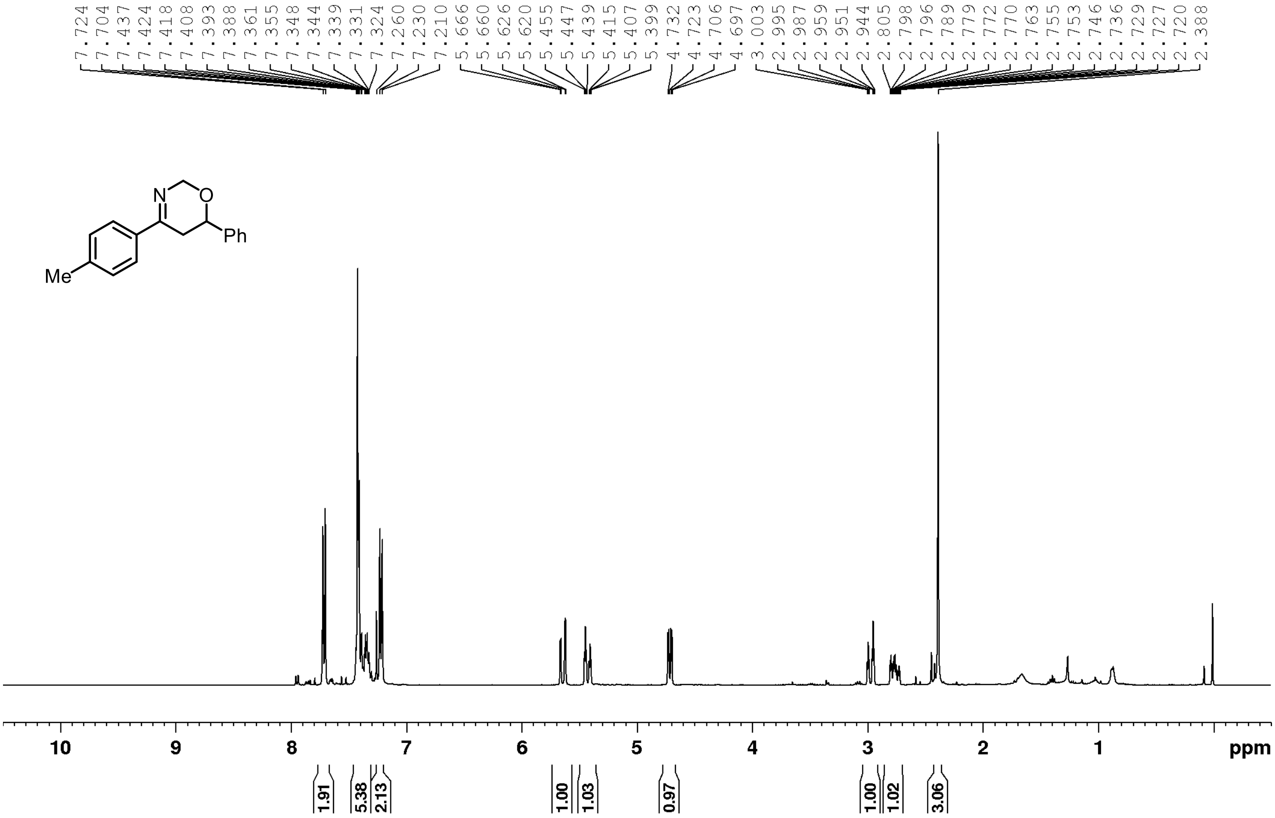

^{13}C NMR (100 MHz, CDCl$_3$)

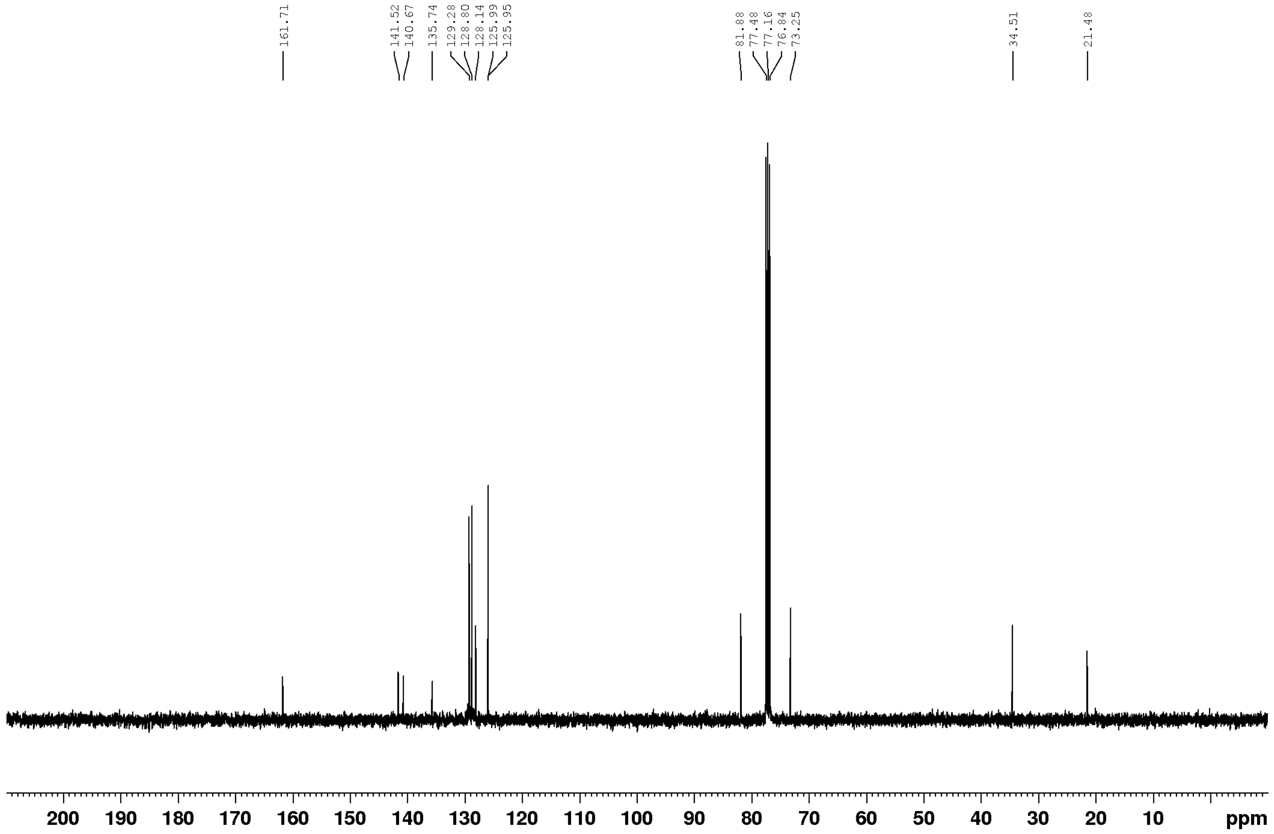

6-Phenyl-4-(*o*-tolyl)-5,6-dihydro-2*H*-1,3-oxazine (4h)

^{1}H NMR (400 MHz, $CDCl_3$)

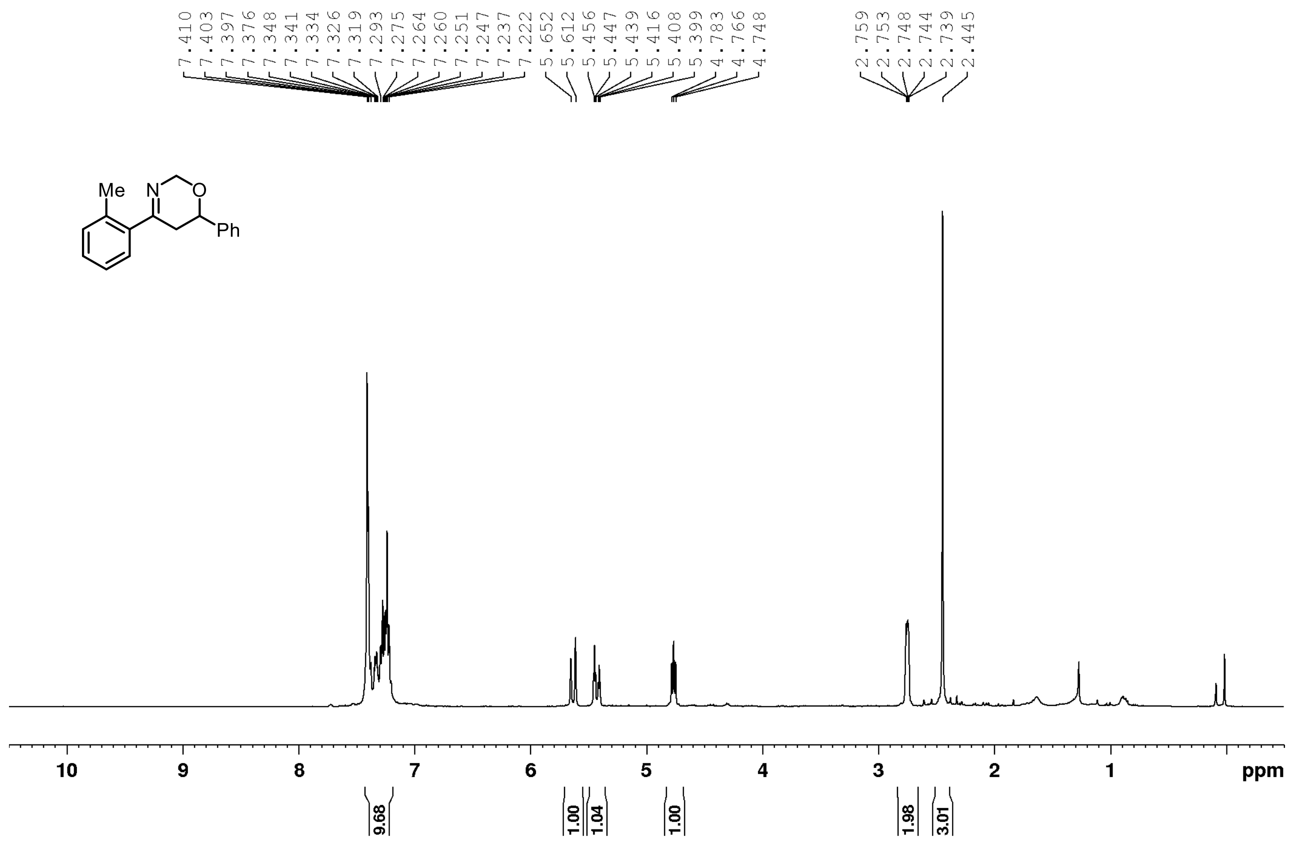

^{13}C NMR (125 MHz, $CDCl_3$)

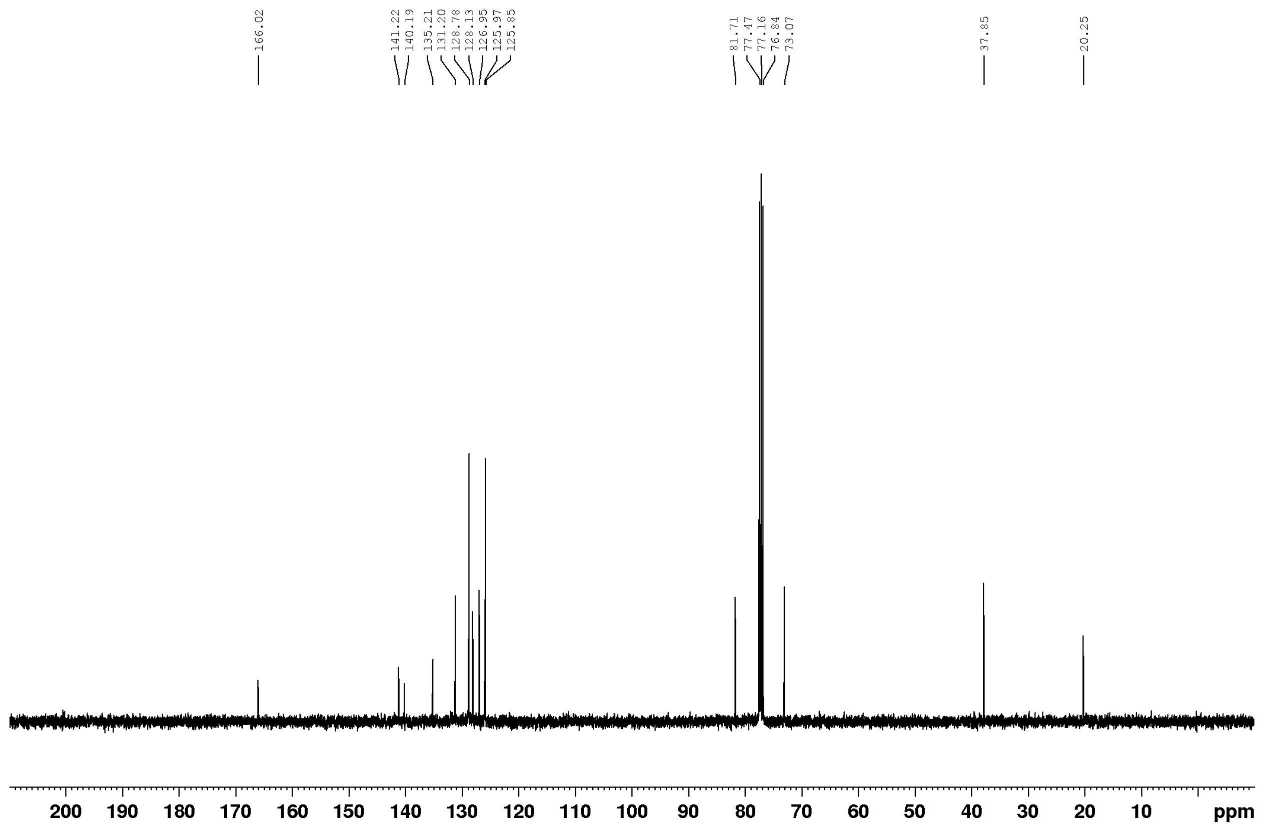

Supplement: Supplementary file 1 — Supporting Information [file ADVS-11-2307563-s001.pdf]
